# Posicionamento sobre a Saúde Cardiovascular nas Mulheres – 2022

**DOI:** 10.36660/abc.20220734

**Published:** 2022-11-07

**Authors:** Glaucia Maria Moraes de Oliveira, Maria Cristina Costa de Almeida, Celi Marques-Santos, Maria Elizabeth Navegantes Caetano Costa, Regina Coeli Marques de Carvalho, Cláudia Maria Vilas Freire, Lucelia Batista Neves Cunha Magalhães, Ludhmila Abrahão Hajjar, Maria Alayde Mendonça Rivera, Marildes Luiza de Castro, Walkiria Samuel Avila, Alexandre Jorge Gomes de Lucena, Andréa Araujo Brandão, Ariane Vieira Scarlatelli Macedo, Carla Janice Baister Lantieri, Carisi Anne Polanczyk, Carlos Japhet da Matta Albuquerque, Daniel Born, Eduardo Belisário Falcheto, Érika Olivier Vilela Bragança, Fabiana Goulart Marcondes Braga, Fernanda M. Consolim Colombo, Ieda Biscegli Jatene, Isabela Bispo Santos da Silva Costa, Ivan Romero Rivera, Jaqueline Ribeiro Scholz, José Xavier de Melo, Magaly Arrais dos Santos, Maria Cristina de Oliveira Izar, Maria Fátima Azevedo, Maria Sanali Moura, Milena dos Santos Barros Campos, Olga Ferreira de Souza, Orlando Otávio de Medeiros, Sheyla Cristina Tonheiro Ferro da Silva, Stéphanie Itala Rizk, Thais de Carvalho Vieira Rodrigues, Thaís Rocha Salim, Viviana de Mello Guzzo Lemke, Elizabeth Regina Giunco Alexandre

**Affiliations:** 1 Universidade Federal do Rio de Janeiro Rio de Janeiro RJ Brasil Universidade Federal do Rio de Janeiro , Rio de Janeiro RJ – Brasil; 2 Centro Universitário de Belo Horizonte Belo Horizonte MG Brasil Centro Universitário de Belo Horizonte , Belo Horizonte MG – Brasil; 3 Universidade Tiradentes Aracaju SE Brasil Universidade Tiradentes (UNIT), Aracaju SE – Brasil; 4 Centro Universitário do Estado Pará Belém PA Brasil Centro Universitário do Estado Pará (CESUPA), Belém PA – Brasil; 5 Secretaria de Saúde do Estado do Ceará Fortaleza CE Brasil Secretaria de Saúde do Estado do Ceará , Fortaleza CE – Brasil; 6 Hospital das Clínicas UFMG Belo Horizonte MG Brasil Hospital das Clínicas UFMG , Belo Horizonte MG – Brasil; 7 Faculdade de Medicina UFBA Salvador BA Brasil Faculdade de Medicina da UFBA , Salvador BA – Brasil; 8 Instituto do Coração Hospital das Clínicas FMUSP São Paulo SP Brasil Instituto do Coração (Incor) do Hospital das Clínicas FMUSP , São Paulo SP – Brasil; 9 Hospital Universitário Professor Alberto Antunes Universidade Federal de Alagoas Maceió AL Brasil Hospital Universitário Professor Alberto Antunes / Universidade Federal de Alagoas , Maceió AL – Brasil; 10 Faculdade IPEMED de Ciências Médicas Belo Horizonte MG Brasil Faculdade IPEMED de Ciências Médicas , Belo Horizonte MG – Brasil; 11 Hospital Agamenom Magalhães Recife PE Brasil Hospital Agamenom Magalhães , Recife PE – Brasil; 12 Universidade do Estado do Rio de Janeiro Rio de Janeiro RJ Brasil Universidade do Estado do Rio de Janeiro , Rio de Janeiro RJ – Brasil; 13 Santa Casa de Misericórdia de São Paulo São Paulo SP Brasil Santa Casa de Misericórdia de São Paulo , São Paulo SP – Brasil; 14 Universidade Municipal de São Caetano do Sul Caetano do Sul SP Brasil Universidade Municipal de São Caetano do Sul , Caetano do Sul SP – Brasil; 15 Hospital de Clínicas UFRS Porto Alegre RS Brasil Hospital de Clínicas da UFRS , Porto Alegre RS – Brasil; 16 Instituto de Medicina Integral Professor Fernando Figueira Recife PE Brasil Instituto de Medicina Integral Professor Fernando Figueira (IMIP), Recife PE – Brasil; 17 Escola Paulista de Medicina São Paulo SP Brasil Escola Paulista de Medicina , São Paulo SP – Brasil; 18 Hospital Felício Rocho Belo Horizonte MG Brasil Hospital Felício Rocho , Belo Horizonte MG – Brasil; 19 Hospital Pio XII São José dos Campos SP Brasil Hospital Pio XII , São José dos Campos SP – Brasil; 20 Hospital do Coração São Paulo SP Brasil Hospital do Coração (HCor), São Paulo SP – Brasil; 21 UDI Hospital Rede D’Or São Luís MA Brasil UDI Hospital , Rede D’Or , São Luís MA – Brasil; 22 Universidade Federal de São Paulo São Paulo SP Brasil Universidade Federal de São Paulo (UNIFESP), São Paulo SP – Brasil; 23 Ministério da Saúde Brasília DF Brasil Ministério da Saúde , Brasília DF – Brasil; 24 Hospital Federal do Rio Grande do Norte Natal RN Brasil Hospital Federal do Rio Grande do Norte , Natal RN – Brasil; 25 Hospital Universitário de Sergipe Aracajú SE Brasil Hospital Universitário de Sergipe , Aracajú , SE – Brasil; 26 Rede D’Or Hospitais Rio de Janeiro RJ Brasil Rede D’Or Hospitais , Rio de Janeiro RJ – Brasil; 27 CEMISE Aracajú SE Brasil CEMISE , Aracajú SE – Brasil; 28 Hospital São Lucas D’Or Aracaju SE Brasil Hospital São Lucas D’Or , Aracaju SE – Brasil; 29 Cardiocare Clínica Cardiológica Curitiba PR Brasil Cardiocare Clínica Cardiológica , Curitiba PR – Brasil

**Table:** 

Posicionamento sobre a Saúde Cardiovascular nas Mulheres – 2022	

O relatório abaixo lista as declarações de interesse conforme relatadas à SBC pelos especialistas durante o período de desenvolvimento deste posicionamento, 2022	

Especialista	Tipo de relacionamento com a indústria
Alexandra Oliveira de Mesquita	Nada a ser declarado
Alexandre Jorge Gomes de Lucena	Nada a ser declarado
Andréa Araujo Brandão	Declaração financeira A - Pagamento de qualquer espécie e desde que economicamente apreciáveis, feitos a (i) você, (ii) ao seu cônjuge/ companheiro ou a qualquer outro membro que resida com você, (iii) a qualquer pessoa jurídica em que qualquer destes seja controlador, sócio, acionista ou participante, de forma direta ou indireta, recebimento por palestras, aulas, atuação como proctor de treinamentos, remunerações, honorários pagos por participações em conselhos consultivos, de investigadores, ou outros comitês, etc. Provenientes da indústria farmacêutica, de órteses, próteses, equipamentos e implantes, brasileiras ou estrangeiras: - Servier: Acertil, Acertalix, Acertanlo, Triplixan. Outros relacionamentos Financiamento de atividades de educação médica continuada, incluindo viagens, hospedagens e inscrições para congressos e cursos, provenientes da indústria farmacêutica, de órteses, próteses, equipamentos e implantes, brasileiras ou estrangeiras: - Servier: Acertil, Acertalix, Acertanlo, Triplixan.
Ariane Vieira Scarlatelli Macedo	Declaração financeira A - Pagamento de qualquer espécie e desde que economicamente apreciáveis, feitos a (i) você, (ii) ao seu cônjuge/ companheiro ou a qualquer outro membro que resida com você, (iii) a qualquer pessoa jurídica em que qualquer destes seja controlador, sócio, acionista ou participante, de forma direta ou indireta, recebimento por palestras, aulas, atuação como proctor de treinamentos, remunerações, honorários pagos por participações em conselhos consultivos, de investigadores, ou outros comitês, etc. Provenientes da indústria farmacêutica, de órteses, próteses, equipamentos e implantes, brasileiras ou estrangeiras: - Bayer: Anticoagulação e insuficiência cardíaca; Pfizer: Anticoagulação e amiloidose; Jannsen: Leucemia. Outros relacionamentos Financiamento de atividades de educação médica continuada, incluindo viagens, hospedagens e inscrições para congressos e cursos, provenientes da indústria farmacêutica, de órteses, próteses, equipamentos e implantes, brasileiras ou estrangeiras: - Bayer: Insuficiência cardíaca.
Carisi Anne Polanczyk	Nada a ser declarado
Carla Janice Baister Lantieri	Nada a ser declarado
Carlos Japhet da Matta Albuquerque	Nada a ser declarado
Celi Marques Santos	Nada a ser declarado
Cláudia Maria Vilas Freire	Nada a ser declarado
Daniel Born	Nada a ser declarado
Eduardo Belisario Falchetto	Nada a ser declarado
Elizabeth Regina Giunco Alexandre	Declaração financeira A - Pagamento de qualquer espécie e desde que economicamente apreciáveis, feitos a (i) você, (ii) ao seu cônjuge/ companheiro ou a qualquer outro membro que resida com você, (iii) a qualquer pessoa jurídica em que qualquer destes seja controlador, sócio, acionista ou participante, de forma direta ou indireta, recebimento por palestras, aulas, atuação como proctor de treinamentos, remunerações, honorários pagos por participações em conselhos consultivos, de investigadores, ou outros comitês, etc. Provenientes da indústria farmacêutica, de órteses, próteses, equipamentos e implantes, brasileiras ou estrangeiras: - Lilly: Trulicity, Jardiance, Glyxambi. Outros relacionamentos Financiamento de atividades de educação médica continuada, incluindo viagens, hospedagens e inscrições para congressos e cursos, provenientes da indústria farmacêutica, de órteses, próteses, equipamentos e implantes, brasileiras ou estrangeiras: - Novo Nordisk: Ozempic.
Érika Olivier Vilela Bragança	Declaração financeira A - Pagamento de qualquer espécie e desde que economicamente apreciáveis, feitos a (i) você, (ii) ao seu cônjuge/ companheiro ou a qualquer outro membro que resida com você, (iii) a qualquer pessoa jurídica em que qualquer destes seja controlador, sócio, acionista ou participante, de forma direta ou indireta, recebimento por palestras, aulas, atuação como proctor de treinamentos, remunerações, honorários pagos por participações em conselhos consultivos, de investigadores, ou outros comitês, etc. Provenientes da indústria farmacêutica, de órteses, próteses, equipamentos e implantes, brasileiras ou estrangeiras: - Bayer: Xarelto; Pfizer: Eliquis; Biocath: dispositivos cardíacos eletrônicos implantáveis e arritmias cardíacas. Outros relacionamentos Financiamento de atividades de educação médica continuada, incluindo viagens, hospedagens e inscrições para congressos e cursos, provenientes da indústria farmacêutica, de órteses, próteses, equipamentos e implantes, brasileiras ou estrangeiras: - Bayer: Xarelto; Pfizer: Eliquis; Daiichi Sankyo: Lixiana; Boehringer Ingelheim: Pradaxa. Participação societária de qualquer natureza e qualquer valor economicamente apreciável de empresas na área de saúde, de ensino ou em empresas concorrentes ou fornecedoras da SBC: - Área de Saúde.
Fabiana Goulart Marcondes Braga	Nada a ser declarado
Fernanda M. Consolim Colombo	Outros relacionamentos Financiamento de atividades de educação médica continuada, incluindo viagens, hospedagens e inscrições para congressos e cursos, provenientes da indústria farmacêutica, de órteses, próteses, equipamentos e implantes, brasileiras ou estrangeiras: - Daiichi; Ache; Servier; AstraZeneca; Merck: Anti-hipertensivos.
Gláucia Maria Moraes de Oliveira	Nada a ser declarado
Ieda Biscegli Jatene	Declaração financeira A - Pagamento de qualquer espécie e desde que economicamente apreciáveis, feitos a (i) você, (ii) ao seu cônjuge/ companheiro ou a qualquer outro membro que resida com você, (iii) a qualquer pessoa jurídica em que qualquer destes seja controlador, sócio, acionista ou participante, de forma direta ou indireta, recebimento por palestras, aulas, atuação como proctor de treinamentos, remunerações, honorários pagos por participações em conselhos consultivos, de investigadores, ou outros comitês, etc. Provenientes da indústria farmacêutica, de órteses, próteses, equipamentos e implantes, brasileiras ou estrangeiras: - AstraZeneca: Palivizumabe. Outros relacionamentos Financiamento de atividades de educação médica continuada, incluindo viagens, hospedagens e inscrições para congressos e cursos, provenientes da indústria farmacêutica, de órteses, próteses, equipamentos e implantes, brasileiras ou estrangeiras: - AstraZeneca: Palivizumabe.
Isabela Bispo Santos da Silva Costa	Nada a ser declarado
Ivan Romero Rivera	Nada a ser declarado
Jaqueline Ribeiro Scholz	Nada a ser declarado
José Xavier de Melo Filho	Nada a ser declarado
Lucelia Batista Neves Cunha Magalhães	Nada a ser declarado
Ludhmila Abrahão Hajjar	Nada a ser declarado
Magaly Arrais dos Santos	Declaração financeira A - Pagamento de qualquer espécie e desde que economicamente apreciáveis, feitos a (i) você, (ii) ao seu cônjuge/ companheiro ou a qualquer outro membro que resida com você, (iii) a qualquer pessoa jurídica em que qualquer destes seja controlador, sócio, acionista ou participante, de forma direta ou indireta, recebimento por palestras, aulas, atuação como proctor de treinamentos, remunerações, honorários pagos por participações em conselhos consultivos, de investigadores, ou outros comitês, etc. Provenientes da indústria farmacêutica, de órteses, próteses, equipamentos e implantes, brasileiras ou estrangeiras: - Edwards: Implante transcateter valvar; Boston: Implante transcateter valvar; Medtronic: Implante.
Marcia de Melo Barbosa	Nada a ser declarado
Maria Alayde Mendonça Rivera	Nada a ser declarado
Maria Cristina Costa de Almeida	Nada a ser declarado
Maria Cristina de Oliveira Izar	Declaração financeira A - Pagamento de qualquer espécie e desde que economicamente apreciáveis, feitos a (i) você, (ii) ao seu cônjuge/ companheiro ou a qualquer outro membro que resida com você, (iii) a qualquer pessoa jurídica em que qualquer destes seja controlador, sócio, acionista ou participante, de forma direta ou indireta, recebimento por palestras, aulas, atuação como proctor de treinamentos, remunerações, honorários pagos por participações em conselhos consultivos, de investigadores, ou outros comitês, etc. Provenientes da indústria farmacêutica, de órteses, próteses, equipamentos e implantes, brasileiras ou estrangeiras: - Bayer/Xarelto; Daiichi Sankyo/Lixiana; Libbs/Propafenona e Amiodarona; Pfizer/Eliquis.
Maria Elizabeth Navegantes Caetano Costa	Declaração financeira A - Pagamento de qualquer espécie e desde que economicamente apreciáveis, feitos a (i) você, (ii) ao seu cônjuge/ companheiro ou a qualquer outro membro que resida com você, (iii) a qualquer pessoa jurídica em que qualquer destes seja controlador, sócio, acionista ou participante, de forma direta ou indireta, recebimento por palestras, aulas, atuação como proctor de treinamentos, remunerações, honorários pagos por participações em conselhos consultivos, de investigadores, ou outros comitês, etc. Provenientes da indústria farmacêutica, de órteses, próteses, equipamentos e implantes, brasileiras ou estrangeiras: - Libbs: Plenance Enze; Servier: Vastarel. Outros relacionamentos Financiamento de atividades de educação médica continuada, incluindo viagens, hospedagens e inscrições para congressos e cursos, provenientes da indústria farmacêutica, de órteses, próteses, equipamentos e implantes, brasileiras ou estrangeiras: - Libbs; Servier: participação em congresso.
Maria Fátima de Azevedo	Outros relacionamentos Financiamento de atividades de educação médica continuada, incluindo viagens, hospedagens e inscrições para congressos e cursos, provenientes da indústria farmacêutica, de órteses, próteses, equipamentos e implantes, brasileiras ou estrangeiras: - Novo Nordisk: Diabetes; Boehinger: Diabetes; Biolab: Hipertensão e anticoagulante. Vínculo empregatício com a indústria farmacêutica, de órteses, próteses, equipamentos e implantes, brasileiras ou estrangeiras, assim como se tem relação vínculo empregatício com operadoras de planos de saúde ou em auditorias médicas (incluindo meio período) durante o ano para o qual você está declarando: - Unimed Natal.
Maria Sanali Moura de Oliveira Paiva	Nada a ser declarado
Marildes Luiza de Castro	Declaração financeira A - Pagamento de qualquer espécie e desde que economicamente apreciáveis, feitos a (i) você, (ii) ao seu cônjuge/ companheiro ou a qualquer outro membro que resida com você, (iii) a qualquer pessoa jurídica em que qualquer destes seja controlador, sócio, acionista ou participante, de forma direta ou indireta, recebimento por palestras, aulas, atuação como proctor de treinamentos, remunerações, honorários pagos por participações em conselhos consultivos, de investigadores, ou outros comitês, etc. Provenientes da indústria farmacêutica, de órteses, próteses, equipamentos e implantes, brasileiras ou estrangeiras: - AstraZeneca: Forxiga/Insuficiência cardíaca; Servier: Acertil/Hipertensão arterial.
Milena dos Santos Barros Campos	Nada a ser declarado
Olga Ferreira de Souza	Nada a ser declarado
Orlando Otávio de Medeiros	Nada a ser declarado
Regina Coeli Marques de Carvalho	Nada a ser declarado
Sheyla Cristina Tonheiro Ferro da Silva	Declaração financeira A - Pagamento de qualquer espécie e desde que economicamente apreciáveis, feitos a (i) você, (ii) ao seu cônjuge/ companheiro ou a qualquer outro membro que resida com você, (iii) a qualquer pessoa jurídica em que qualquer destes seja controlador, sócio, acionista ou participante, de forma direta ou indireta, recebimento por palestras, aulas, atuação como proctor de treinamentos, remunerações, honorários pagos por participações em conselhos consultivos, de investigadores, ou outros comitês, etc. Provenientes da indústria farmacêutica, de órteses, próteses, equipamentos e implantes, brasileiras ou estrangeiras: - Boehringer Ingelheim, Novartis Farmacêutica, AstraZeneca, Servier, Libbs. Outros relacionamentos Financiamento de atividades de educação médica continuada, incluindo viagens, hospedagens e inscrições para congressos e cursos, provenientes da indústria farmacêutica, de órteses, próteses, equipamentos e implantes, brasileiras ou estrangeiras: - Boehringer Ingelheim, Novartis Farmacêutica, Novo Nordisk, AstraZeneca.
Stéphanie Itala Rizk	Nada a ser declarado
Thais de Carvalho Vieira Rodrigues	Outros relacionamentos Financiamento de atividades de educação médica continuada, incluindo viagens, hospedagens e inscrições para congressos e cursos, provenientes da indústria farmacêutica, de órteses, próteses, equipamentos e implantes, brasileiras ou estrangeiras: - AstraZeneca.
Thaís Rocha Salim	Nada a ser declarado
Viviana de Mello Guzzo Lemke	Nada a ser declarado
Walkiria Samuel Avila	Nada a ser declarado

**Table:** 

Lista de Abreviaturas e Siglas	
AAS	Ácido Acetilsalicílico
Angio-TC	Angiotomografia de Coronárias
APS	Atenção Primária a Saúde
AVC	Acidente Vascular Cerebral
CAC	Escore de Cálcio Coronariano
CC	Cardiopatia Congênita
CDI	Cardiodesfibrilador Implantável
CV	Cardiovascular
DA	Doença de Alzheimer
DAC	Doença Arterial Coronariana
DALYs	Anos de vida ajustados por incapacidade (do inglês, *Disability-Adjusted Life Years* ) – 1 DALY representa a perda do equivalente a 1 ano de saúde completa
DAP	Doença Arterial Periférica
DApC	Doenças do Aparelho Circulatório
DCR	Doença Cardíaca Reumática
DCV	Doenças Cardiovasculares
DEAC	Dissecção Espontânea da Artéria Coronária
DG	Diabetes Gestacional
DHG	Doença Hipertensiva da Gravidez
DIC	Doença Isquêmica do Coração
DM	Diabetes mellitus
DMV	Doença Microvascular
DOACs	Anticoagulantes de ação direta
DPP-4	Dipeptidil Peptidase-4
DRC	Doença Renal Crônica
DSS	Determinantes Sociais de Saúde
ECG	Eletrocardiograma
EMI	Espessura do Complexo Médio-Intimal
FA	Fibrilação Atrial
FEVE	Fração de Ejeção do Ventrículo Esquerdo
FPR	Fatores Potencializadores de Risco
FR	Fatores de Risco
FRCV	Fatores de Risco Cardiovasculares
GBD	Do inglês: *Global Burden of Disease*
HAS	Hipertensão Arterial Sistêmica
HG	Hipertensão Gestacional
HVE	Hipertrofia Ventricular Esquerda
IAM	Infarto Agudo do Miocárdio
IAMSSST	Infarto Agudo do Miocárdio Sem Supra do Segmento ST
IC	Insuficiência Cardíaca
ICFEp	Insuficiência Cardíaca com Fração de Ejeção Preservada
ICFEr	Insuficiência Cardíaca com Fração de Ejeção Reduzida
II	Intervalo de Incerteza
IMC	Índice de Massa Corporal
IMCSST	Infarto do Miocárdio Com Supra de ST
INOCA	Isquemia na ausência de obstrução arterial coronariana
INRA	Inibidores da neprilisina/bloqueadores de angiotensina II
IRM	Índice de Resistência Microvascular
LDL-c	Colesterol da lipoproteína de baixa densidade
MAC	Malformações do Aparelho Circulatório
MINOCA	Infarto do miocárdio na ausência de obstrução arterial coronária
MSC	Morte Súbita Cardíaca
NT-proBNP	Fragmento N-terminal do peptídeo natriurético tipo B
OCDE	Organização para Cooperação e Desenvolvimento Econômico
OMS	Organização Mundial da Saúde
PA	Pressão Arterial
PE	Pré-Eclâmpsia
PNS	Pesquisa Nacional em Saúde
QT	Quimioterápico
QV	Qualidade de Vida
RCV	Risco Cardiovascular
RMC	Ressonância Magnética Cardíaca
RVM	Revascularização Miocárdica
SBC	Sociedade Brasileira de Cardiologia
SCA	Síndrome Coronariana Aguda
SGLT2	Do inglês: *Sodium-Glucose Cotransporter-2*
SLG	*Strain* Longitudinal Global
SOP	Síndrome dos Ovários Policísticos
TC	Transplante Cardíaco
TE	Teste Ergométrico
THM	Terapia Hormonal na Menopausa
VPC	Vasoespasmo Coronariano

Legendas para as tabelas de recomendação e nível de evidência:

**Table:** 

Classe de recomendação:	
	**I –** Condições para as quais há evidências conclusivas ou, em sua falta, consenso geral de que o procedimento é seguro e útil/eficaz.
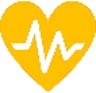	**II –** Condições para as quais há evidências conflitantes e/ou divergência de opinião sobre segurança, e utilidade/eficácia do procedimento.
	**IIA –** Peso ou evidência/opinião a favor do procedimento. A maioria aprova
	**IIB –** Segurança e utilidade/eficácia menos bem estabelecida, não havendo predomínio de opiniões a favor.
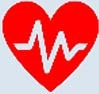	**III –** Condições para as quais há evidências e/ou consenso de que o procedimento não é útil/ eficaz e, em alguns casos, pode ser prejudicial.
**Nível de evidência:**	
**Nível A**	Dados obtidos a partir de múltiplos estudos randomizados de bom porte, concordantes e/ou de metanálise robusta de estudos clínicos randomizados.
**Nível B**	Dados obtidos a partir de metanálise menos robusta, a partir de um único estudo randomizado ou de estudos não randomizados (observacionais).
**Nível C**	Dados obtidos de opiniões consensuais de especialistas.

## Sumário

Introdução 822

1. Destaques deste Posicionamento 823

2. Epidemiologia das Doenças Cardiovasculares nas Mulheres 827

2.1. Doenças do Aparelho Circulatório em Crianças e Adolescentes do Sexo Feminino no Brasil 835

3. Fatores de Risco Cardiovascular 837

3.1. Introdução 837

4. Doenças Cardiovasculares nas Mulheres 844

4.1. Doença Isquêmica do Coração 844

**4.1.1. Doença Arterial Coronariana** 844

**4.1.2. Isquemia na Ausência de Obstrução Arterial Coronariana** 846

**4.1.3. Infarto do Miocárdio na Ausência de Obstrução Arterial Coronariana** 846

**4.1.4. Dissecção Espontânea de Artéria Coronária** 846

**4.1.5. Doença Microvascular** 846

**4.1.6. Vasoespasmo Coronariano** 846

**4.1.7. Trombose/Embolia Coronariana** 846

4.2. Insuficiência Cardíaca 846

**4.2.1. Tratamento Farmacológico e Não Farmacológico da ICFEr e ICFEp** 847

4.3. Arritmias 850

**4.3.1. Taquicardia Ventricular e Morte Súbita Cardíaca** 850

**4.3.2. Fibrilação Atrial** 850

4.4. Doença Cardiovascular e Câncer 851

4.5. Acidente Vascular Cerebral 853

4.6. Doença Arterial Periférica 853

4.7. Demência 854

4.8. Doenças Valvares 856

**4.8.1. Estenose Aórtica** 856

**4.8.2. Doença Valvar Mitral** 857

**4.8.3. Doença Reumática** 858

4.9. Diabetes Mellitus, Pré-eclâmpsia e Doenças Hipertensivas na Gravidez 858

**4.9.1. Diabetes Mellitus** 858

**4.9.2. Doenças Hipertensivas na Gravidez** 859

4.10. Gravidez na Adolescência 859

5. Peculiaridades dos Métodos Propedêuticos nas Mulheres 863

5.1. Eletrocardiograma 864

5.2. Teste Ergométrico 864

5.3. Ultrassonografia de Carótidas 864

5.4. Ecocardiografia 864

5.5. Cintilografia Miocárdica 864

5.6. Escore de Cálcio e Angiotomografia de Coronárias 865

5.7. Ressonância Magnética Cardíaca 865

5.8. Coronariografia 865

6. Representação de Mulheres nos Estudos Clínicos sobre Fatores de Risco e Doença Cardiovascular 867

7. Medidas de Prevenção Primária nas Mulheres 869

8. Burnout, Qualidade de Vida e Espiritualidade nas Mulheres 869

8.1. Burnout 869

8.2. Qualidade de Vida 870

8.3 Espiritualidade 871

9. Implicações Cardiovasculares da COVID-19 na Gestação 872

10. Perspectivas Futuras para a Melhoria do Cuidado

Cardiovascular das Mulheres 874

Referências 875

## Introdução

Entre as doenças crônicas não transmissíveis, as DCV constituem a principal causa de morte no mundo e no Brasil, que apresenta uma das mais altas taxas de mortalidade da América do Sul. ^1^ As DCV respondem por um terço das mortes por todas as causas e acometem homens e mulheres em todas as faixas etárias, representando mais do que o dobro das mortes por todas as neoplasias associadas. ^1^ Nas mulheres, observa-se aumento da prevalência de DCV e de morte por DCV após a menopausa, o que agrava as perspectivas em futuro próximo pelo envelhecimento e adoecimento da população feminina no Brasil.

Atualmente a DIC é responsável pela maioria das mortes em todas as unidades da federação, seguida pelas doenças cerebrovasculares. ^1^ Um aspecto particular é a desigualdade de acometimento entre as regiões, no acesso tanto ao diagnóstico como ao tratamento, de acordo com as particularidades determinadas pelos indicadores sociais e econômicos, nas macrorregiões, estados e cidades de diferentes portes no Brasil. Cerca de metade da mortalidade por DCV antes dos 65 anos pode ser atribuída à pobreza e às desigualdades sociais. ^2^ Alimentação inadequada, baixa atividade física, consumo de álcool e tabagismo são outros importantes FR para as DCV em mulheres, mais prevalentes nas classes sociais menos favorecidas da população, incluindo as crianças e as adolescentes brasileiras. ^3^ Assim, os programas de prevenção primária e secundária, bem como o maior acesso ao diagnóstico, nessa camada da população poderão ter impacto ainda maior na morbimortalidade por DCV.

Na maioria das vezes, as DCV podem ser prevenidas por ações de saúde pública que envolvem o controle de FR e o manejo clínico otimizado dos pacientes. A redução das DCV em mulheres no Brasil e no mundo é uma tarefa complexa, que depende de inúmeros agentes e de um esforço continuado.

A SBC, que reúne a maioria dos cardiologistas brasileiros e tem em seus quadros um terço de cardiologistas mulheres, vem desenvolvendo ações continuadas para a diminuição da morbimortalidade por DCV através do Departamento de Cardiologia da Mulher.

Desse modo, foi publicada, nos *Arquivos Brasileiros de Cardiologia* , a “Carta das Mulheres”, ^4^ que avançou em estabelecer deliberações de ações concretas para diminuir a morbimortalidade por DCV em mulheres. Dentre elas destacam-se: trabalhar coletivamente em defesa das metas globais para prevenção e controle de doenças crônicas não transmissíveis, especialmente as DCV nas brasileiras; estabelecer campanhas de prevenção cardiovascular, promovendo esforços consistentes para obter a meta de redução de 30% da taxa de mortalidade até 2030; elaborar e sugerir políticas governamentais para promover ambientes adequados para a redução da exposição ao risco, facilitando a adoção de hábitos saudáveis em ambientes escolares, de trabalho e de lazer, voltadas ao combate às DCV na mulher; atuar junto aos governos para o desenvolvimento e a aplicação de programa de prevenção cardiovascular, além da incorporação de tecnologias custo-efetivas para a redução da morbimortalidade por DCV nas mulheres; mobilizar os meios de comunicação para levar informações continuadas sobre a importância das DCV nas mulheres, seus principais FR e formas de prevenção, ampliando a divulgação para a população sobre a importância do diagnóstico precoce; fornecer o mais alto nível de educação médica continuada; promover o intercâmbio técnico-científico, cultural e social entre as cardiologistas do Brasil e do mundo; e fomentar o conhecimento científico necessário para aumentar a participação das mulheres nas ciências e nos eventos científicos das áreas de saúde e ciências afins.

Mesmo tendo em vista os enormes avanços científicos e tecnológicos já alcançados ou em perspectiva na cardiologia, precisamos modificar o paradigma de saúde e doença, com o objetivo de abordagem populacional, que viabilize o benefício de tais conquistas a toda a população. Para tanto, faz-se necessário um grande pacto entre a sociedade civil, as sociedades de especialidades, o governo e a comunidade para que seja implementada uma reforma na educação médica e na educação dos demais profissionais da saúde, paralelamente a uma ampla discussão na sociedade, contribuindo para conscientização, prevenção, diagnóstico e tratamento das doenças que mais causam mortes em homens e mulheres no Brasil.

O Brasil, ao estabelecer na Constituição Federal o compromisso inalienável com a preservação da dignidade da pessoa humana, definiu a saúde como direito social, assegurando o acesso universal, integral e gratuito a todos os brasileiros.

Mesmo com as garantias constitucionais, as iniquidades em relação às mulheres persistem e ainda espreitam a sociedade brasileira. Por isso, necessário se faz, por intermédio de ações afirmativas, remarcar a necessidade de assegurar a igualdade imprescindível entre homens e mulheres, particularmente em relação à conscientização das DCV na mulher, que lamentavelmente ainda são negligenciadas no Brasil. ^2^

Desse modo, foi proposto o “Dia Nacional de Conscientização das Doenças Cardiovasculares nas Mulheres” pelos autores da “Carta das Mulheres”, aprovado pela Lei 14.320 de 2022, sancionada pelo presidente da República e publicada no Diário Oficial da União de 1º de abril. Esse dia teve origem no Projeto de Lei 1.136/2019, de autoria da deputada federal e cardiologista Mariana Carvalho. Será comemorado no dia 14 de maio em homenagem ao nascimento da médica Bettina Ferro de Souza, que foi a primeira presidente mulher da SBC.

É fundamental promover iniciativas para aumentar o conhecimento sobre a importância da saúde cardiovascular ao longo da vida da mulher. Além disso, é fundamental compreender melhor as disparidades locais na saúde cardiovascular das mulheres para definir políticas públicas e assistência à saúde, reduzir lacunas e promover a equidade de sexo na atenção à saúde brasileira.

Nesse sentido, o Departamento de Cardiologia da Mulher da SBC apresenta seu **Posicionamento sobre a Saúde Cardiovascular nas Mulheres** com foco na prevenção primária. Com esse documento, pretendemos contribuir para atingir o objetivo que traçamos na “Carta das Mulheres” de exercer um papel de liderança nas políticas brasileiras para a saúde, fornecendo aos gestores uma visão geral da relevância das DCV nas mulheres. Tal visão vai permitir a eles traçar ações estratégicas para reduzir a prevalência de FR, melhorar o diagnóstico e a abordagem terapêutica, reduzindo assim os desfechos cardiovasculares, com impacto na saúde física, mental e espiritual das mulheres brasileiras.

## 1. Destaques deste Posicionamento

Neste capítulo 1, apresentamos os principais destaques dos capítulos 2 a 10 deste documento.

### Epidemiologia das doenças cardiovasculares nas mulheres

- Houve aumento da prevalência de DCV nos últimos 30 anos nos jovens de 15-49 anos, de ambos os sexos, bem como maior prevalência porcentual das DCV nas mulheres em relação aos homens até o ano de 2011, a partir do qual, a prevalência de DCV nos homens foi proporcionalmente maior. ^1^
- As DCV são a principal causa de morte em adultos de ambos os sexos, representadas nesse segmento etário principalmente pela DIC e pela doença cerebrovascular. A DIC foi responsável por porcentual de óbitos similares em mulheres e homens, enquanto o porcentual dos óbitos por AVC foi maior em mulheres do que em homens. ^5^
- Nas demais faixas etárias, do nascimento ao início da vida adulta, as malformações cardíacas, as complicações cardíacas da febre reumática e as cardiomiopatias, dentre outras, têm papel importante na mortalidade da população em ambos os sexos. ^6^
- Entre os FR tradicionais, o excesso de peso, a obesidade e o diabetes mellitus foram mais frequentes nas mulheres. Cabe ressaltar também que a prevalência de HAS autorreferida no Brasil foi maior no sexo feminino do que no masculino.

### Fatores de risco cardiovascular nas mulheres

- O reconhecimento dos FR da mulher, quer específicos, mais prevalentes ou similares aos dos homens, assim como o conhecimento do risco de 10 anos para DCV aterosclerótica, é um passo fundamental na estratificação de risco das DCV no sexo feminino. ^2^
- Nas mulheres, os FR tradicionais para DCV mais impactantes incluem: diabetes mellitus, HAS, dislipidemia, tabagismo, obesidade e sedentarismo.
- A prevalência dos FR tradicionais citados vem aumentando mesmo em mulheres mais jovens e, quando associados a FR específicos do sexo, contribuem para o aumento da morbimortalidade; em geral, porém, não são considerados na estratificação de RCV. ^7 , 8^
- Fatores de risco específicos do sexo, como síndrome dos ovários policísticos, uso de contraceptivo hormonal, doença hipertensiva da gravidez, eventos adversos da gravidez, THM, riscos agregados às doenças inflamatórias e autoimunes (artrite reumatoide, lúpus eritematoso sistêmico) e distúrbios depressivos são considerados FPR. ^8^
- Na avaliação de RCV nas mulheres, devem-se considerar, além dos estratificadores de risco, os agravantes de risco associados com diabetes mellitus, os FPR e os FR inerentes ao sexo, a fim de instituir mudança do estilo de vida e recomendar medidas de prevenção primária das DCV, com o objetivo de identificar e tratar mais precocemente um maior número de mulheres em risco. ^7 , 8^

### Doenças cardiovasculares nas mulheres

#### 1 – Doença isquêmica do coração

- Atualmente são bem definidos mecanismos adicionais de isquemia coronariana, sendo mais apropriado falar em DIC por se referir às diversas afecções coronárias geradoras de isquemia, como o infarto do miocárdio na ausência de obstrução arterial coronária, isquemia coronária não obstrutiva, dissecção espontânea da artéria coronária, doença microvascular, vasoespasmo coronariano e embolia/trombose coronariana. Nas mulheres, muitos dos mecanismos fisiopatológicos de DIC estão relacionados a uma ou mais dessas afecções, que podem estar presentes mesmo nas mais jovens e gestantes.

#### 2 – Insuficiência cardíaca

- Em relação à fisiopatologia da IC, devido às respostas hormonais, as mulheres mostram diferenças quanto a epidemiologia, apresentação clínica, desfechos e tratamento da doença. ^9 , 10^ Existem fenótipos de IC mais prevalentes em mulheres, como a síndrome de Takotsubo, e específicos, como a cardiomiopatia periparto. Porém, tanto na IC crônica quanto na aguda, ensaios clínicos/registros voltados especialmente para mulheres são escassos e as evidências são provenientes de subanálise de grandes estudos em que as mulheres estão sub-representadas. Registros prospectivos multicêntricos poderiam trazer evidências mais precisas na população feminina, com o envolvimento de maior número de mulheres tanto na IC com fração de ejeção reduzida, quanto na IC com fração de ejeção preservada.

#### 3 – Arritmias

- As mulheres têm mais taquicardia sinusal e taquicardia por reentrada nodal, sendo que a gravidez aumenta o risco de taquicardias supraventriculares. ^11^
- Na síndrome do QT longo tipo 2, é conhecido um maior risco de morte súbita entre as mulheres e um maior risco de pró-arritmia. No puerpério, o QT longo aumenta o risco de *torsades de pointes.* ^12^
- Nas mulheres, a morte súbita de etiologia cardíaca ocorre mais por causas não isquêmicas, sendo os homens mais reanimados e tratados com desfibrilador. O implante de cardiodesfibrilador implantável, como prevenção tanto primária quanto secundária, é mais comum nos homens, porém o risco de complicações relacionadas ao procedimento é maior nas mulheres. ^13^
- Embora a incidência de FA seja maior nos homens, as mulheres mais idosas têm mais FA, mais sintomas e pior qualidade de vida. A HAS e a obesidade são fortes preditores de risco de FA nas mulheres. Elas mais frequentemente apresentam FA paroxística, AVC, tromboembolismo, IC e hospitalizações, além de maior CHA _2_ DS _2_ -VASc e maior risco de mortalidade cardiovascular e por todas as causas. ^14 , 15^

#### 4 – Doença cardiovascular e câncer

- A cardiotoxicidade é um desafio no tratamento dos cânceres na mulher, especialmente o de mama, por ser um dos mais frequentes e por levar à incidência aumentada de eventos cardiovasculares e de mortalidade cardiovascular e por todas as causas em comparação a mulheres sem neoplasia. Os riscos variam de acordo com o tratamento do câncer. Esse pode agredir o sistema cardiovascular levando a disfunção ventricular, desde assintomática e reversível até IC sintomática e irreversível, síndrome coronariana aguda, pericardite, miocardite, arritmias ventriculares, HAS, doença vascular periférica, entre outras. Variantes genéticas são fatores relacionados ao risco de cardiomiopatia, explicando porque pacientes com o mesmo perfil desenvolvem cardiotoxicidade e outras não.
- A análise dos FR cardiovasculares antes, durante e após o diagnóstico e o tratamento oncológico, a intervenção nos FR modificáveis de forma efetiva, além do diagnóstico e do tratamento precoces de cardiotoxicidade dos quimioterápicos irão impactar o prognóstico dessas pacientes.

#### 5 – AVC, doença arterial periférica e demência

- Pesquisas futuras voltadas para o reconhecimento dos FR de AVC específicos do sexo feminino e uma pontuação para identificação desse grupo de maior risco são urgentemente necessárias para elaborar estratégias de prevenção do AVC nas mulheres.
- A doença arterial periférica tem correlação com DIC e, na sua vigência, o tratamento dos FR tradicionais é medida preventiva em ambos os sexos. O reconhecimento de FR específicos da mulher, como gravidez e suas complicações, ou de fatores predominantemente femininos pode permitir uma adequada estratificação de risco e a adoção de medidas de prevenção precoces.
- As mulheres parecem ter maior risco de desenvolver demência e alterações de memória relacionadas à idade do que os homens. Apesar de existirem estudos mostrando que o estrogênio tem papel importante na função cognitiva em mulheres, a THM não demonstrou efeito benéfico em reduzir essas alterações, mesmo em mulheres mais jovens. ^16^
- Em pacientes com menos de 55 anos, independentemente do sexo, as principais causas de alterações cognitivas e demência são doença de Alzheimer, demência vascular, demência frontoparietal e demência relacionada ao alcoolismo.
- Existem FR vasculares relacionados a demência precoce, como AVC, ataque isquêmico transitório, doença renal, DCV, HAS, alcoolismo crônico e intoxicação por drogas. O controle de FR cardiovascular tem importante papel na prevenção de doenças demenciais e cognitivas, principalmente em mulheres mais jovens.

#### 6 – Doenças valvares

- O seguimento das mulheres portadoras de estenose aórtica ou doença valvar mitral exige cuidadoso e contínuo julgamento sobre o melhor modelo de tratamento. As decisões quanto à conduta clínica ou intervencionista dependem do diagnóstico anatômico e funcional da doença e da cuidadosa avaliação da paciente.
- Quando comparadas aos homens, considerando o mesmo grau de calcificação valvar, as mulheres apresentam uma tendência a maior gravidade da estenose aórtica em razão da fibrose do aparelho valvar, que é mais pronunciada do que a calcificação. Apresentam também perfis distintos relacionados à apresentação clínica, à resposta ao tratamento e aos resultados após intervenção valvar. ^17^
- Nas substituições valvares aórticas transcateter, mulheres são mais idosas e apresentam melhor função ventricular esquerda e menor prevalência de doença arterial coronariana; contudo, apresentam comorbidades, como diabetes mellitus e FA. Características anatômicas do sexo feminino, tais como menor distância entre os óstios coronarianos e o anel valvar e maior prevalência de calcificação valvar e da aorta, são responsáveis pela maior incidência de obstrução coronariana durante o procedimento. O menor diâmetro dos vasos periféricos também causa maiores complicações vasculares e sangramento. ^18^
- Dentre as medidas preventivas nas doenças valvares, destacam-se a prevenção primária e secundária da doença reumática e a profilaxia antibiótica da endocardite infecciosa, notadamente no parto. A doença valvar nas mulheres tem características peculiares com impacto significativo nos resultados do tratamento e prognóstico da doença.
- As indicações de profilaxia de endocardite infecciosa na gravidez são: mulheres portadoras de valvopatia reumática, próteses valvares, cardiopatia congênita cianogênica e passado de endocardite infecciosa.

#### 7 – Diabetes mellitus, pré-eclâmpsia e doenças hipertensivas na gravidez

- Diabetes gestacional está associada a complicações materno-fetais, tais como PE, prematuridade e morte perinatal. Mudança de estilo de vida, como atividade física regular na gravidez, na ausência de contraindicações obstétricas ou cardiovasculares, reduz os riscos dessas complicações de forma substancial. ^19^
- A hipertensão na gravidez como FR para DCV no futuro está bem embasada na literatura; mulheres primigestas que desenvolveram HG tiveram maior risco de DCV no futuro, notadamente após a menopausa. Portanto, a melhor recomendação é iniciar a prevenção da PE e outras formas de HG antes da gravidez, incluindo peso corporal adequado, dieta saudável e orientada, além de exercícios físicos regulares. Nas mulheres de alto risco, o uso do ácido acetilsalicílico no primeiro trimestre e a reposição de cálcio nas gestantes com baixa ingesta diária devem ser indicados. ^20^

#### 8 – Gravidez na adolescência

- A gravidez na adolescência aumenta complicações maternas, fetais e neonatais, além de agravar problemas socioeconômicos previamente existentes e influenciar o futuro de gerações, devendo assim, ser abordada de forma eficiente e contínua em todos os níveis socioeconômicos de nossa sociedade.
- Um dos mais importantes fatores de prevenção da gravidez na adolescência é a educação sobre a sexualidade e a saúde reprodutiva, apoiada em evidências científicas e em programas de promoção à saúde. ^21^ A instrução deve ser direcionada a aspectos biológicos, respeito recíproco, atividades sexuais com responsabilidade e uso de métodos contraceptivos seguros e eficazes na prevenção da gravidez e na proteção contra infecções sexualmente transmissíveis. ^22^
- Adolescentes que são orientados por provedores determinados a enfrentar temas difíceis, como prevenção da gravidez e contracepção, estão mais engajados em seu bem-estar. A orientação qualificada e bem feita sobre a sexualidade na adolescência é um investimento para um futuro com mais saúde, proveito e autoestima.

## Peculiaridades dos métodos propedêuticos nas mulheres

- O eletrocardiograma nas mulheres difere em relação à magnitude dos sinais elétricos, com menor amplitude do QRS, do ponto J e da onda T e maior intervalo QT corrigido. A inversão da onda T anterior, isso é, além de V1, ocorre em cerca de 2,3% da população e é mais comum em mulheres, independentemente do *status* de atividade física. É um padrão benigno desde que ocorra em assintomáticas e de baixo risco, pois estudos com RMC sugerem que possa refletir um deslocamento lateral do ventrículo direito. ^23^ Alterações eletrocardiográficas são observadas em 45% das portadoras de implantes mamários: inversão de ondas T, depressão do segmento ST inferolateral, baixa progressão de R de V1-V4, intervalo QT prolongado e hipertrofia ventricular esquerda, podendo assim levar a interpretações enganosas. ^24^
- No TE, as mulheres mostram mais depressão do segmento ST de caráter falso-positivo e a acurácia varia com a probabilidade pré-teste de DIC. Nas mulheres, a associação de cintilografia miocárdica de estresse ao TE tem melhor acurácia diagnóstica do que o TE isoladamente. Essa associação tem excelente valor preditivo negativo em mulheres de risco pré-teste intermediário/alto. ^25^
- A medida da espessura médio-intimal para a reclassificação de risco pode ser utilizada em mulheres com pelo menos dois FR cardiovascular. ^3 , 4^ A presença de placa como fator agravante para estratificação de risco pode ser usada em mulheres com risco intermediário. ^26^
- Mulheres com escore de cálcio maior que zero e calcificação arterial mamária têm risco de eventos isquêmicos maior do que homens. A angiotomografia coronariana evidencia o padrão não obstrutivo de coronárias, que é mais prevalente em mulheres. ^26 , 27^
- Coronárias normais na coronariografia são mais comuns em mulheres. O risco de complicações vasculares da coronariografia é maior entre as mulheres, que apresentam maior tendência para desenvolver lesão renal aguda após contraste. ^28^
- A RMC é excelente opção para as mulheres, especialmente em idade fértil, em gestantes e naquelas em tratamento de câncer de mama. Além disso, pode evidenciar alterações perfusionais e/ou miocárdicas, auxiliando no diagnóstico diferencial da dor torácica e sendo particularmente útil na abordagem da DIC em mulheres.

## Representação das mulheres nos estudos clínicos sobre fatores de risco e doença cardiovascular

- A DCV é a maior causa de morte no mundo e cada vez mais reconhecida como tendo características específicas em relação ao sexo quanto aos processos de adoecer, manifestações clínicas e resultados dos tratamentos. A identificação de diferenças na expressão da DCV em homens e mulheres determina, portanto, que as mulheres devam ser igualmente representadas em ensaios clínicos cardiovasculares.
- Apesar do aumento da representação das mulheres nos estudos clínicos mais recentes, isso não ocorreu em todas as áreas de investigação da DCV. As mulheres permanecem em minoria nos estudos de arritmias, doença arterial coronariana aguda e crônica e IC, em especial naqueles que envolvem intervenções com o uso de dispositivos implantáveis e procedimentos de alta complexidade.
- A identificação das barreiras a serem transpostas, para que se obtenha equidade no cenário da representação dos sujeitos nos estudos clínicos, oferece inúmeras oportunidades para que a sua resolução permita a equidade na seleção e manutenção das mulheres como sujeitos de estudos clínicos sobre DCV e seus FR.
- Essa equidade é necessária para o acurado conhecimento da expressão da DCV e seus FR nas mulheres, bem como do impacto do tratamento no prognóstico cardiovascular das mulheres.

## Medidas de prevenção primária nas mulheres

- A APS é geralmente o primeiro contato da mulher com o setor saúde e ocorre de forma eletiva ou por busca ativa dos agentes comunitários de saúde. As equipes de APS devem contemplar todo o curso de vida das mulheres, com ações voltadas para a promoção da saúde integral, com ênfase no bem-estar físico, mental e espiritual.
- Ao longo do tempo, observou-se uma descentralização dos serviços públicos iniciada pelo Programa de Assistência Integral à Saúde da Mulher e consolidada pela Política de Atenção à Saúde da Mulher. A garantia do acesso das mulheres a todos os níveis de atenção à saúde e a integração das ações e dos serviços permanecem como desafio, assim como o planejamento local e o monitoramento dos indicadores para promover a redução de agravos e de óbitos evitáveis.
- Faz-se necessária uma mudança de paradigma nas políticas públicas voltadas para a saúde integral das mulheres, especialmente quando consideramos que as DCV são a principal causa de morte das mulheres, na maior parte de seu ciclo de vida.

## *Burnout* , qualidade de vida e espiritualidade nas mulheres

- *Burnout* associa-se positivamente com o consumo de bebidas alcoólicas, distúrbios do sono, depressão, sedentarismo, obesidade e dores musculoesqueléticas, sendo um preditor significativo de hipercolesterolemia e diabetes tipo 2, relacionando-se com maior incidência de DIC e hospitalizações por DCV. ^29^
- As condições de trabalho têm impacto conhecido na saúde dos trabalhadores e as mulheres, por estarem mais inseridas no mercado de trabalho e sobrecarregadas com atividade laboral dupla, apresentam altas taxas de *burnout* .
- O curso de vida das mulheres é permeado por experiências de perda, estresse, ansiedade e medo, que aumentam a vulnerabilidade psicológica e facilitam o aparecimento de sintomas de ansiedade-depressão. Entretanto, resiliência, espiritualidade e crenças pessoais parecem desempenhar um papel mediador em algumas dessas variáveis psicológicas, associando-se com melhor qualidade de vida e menor frequência de DCV nas mulheres.

## Implicações cardiovasculares da COVID-19 na gestação

- A gravidade da infecção por SARS-CoV-2 é maior em mulheres grávidas em comparação com não grávidas, com admissão em unidades de terapia intensiva, uso de ventilação mecânica e aumento da mortalidade e morbidade, incluindo infarto do miocárdio, eventos tromboembólicos venosos e outros eventos trombóticos, PE, trabalho de parto e parto prematuros. ^30^ Além disso, a COVID-19 foi associada a uma taxa mais alta (e proporções combinadas) de parto prematuro, PE, cesariana e morte perinatal. ^31^
- O manejo das complicações cardíacas na gravidez deve envolver o acompanhamento multidisciplinar com cardiologista, obstetra e neonatologista.
- A infecção por SARS-CoV-2 na gravidez é importante no diagnóstico diferencial com outras complicações, como dissecção coronariana e cardiomiopatia periparto. ^32^
- O início e a duração da anticoagulação profilática na gravidez associada com infecção por SARS-CoV-2 devem considerar a gravidade da doença, a necessidade de internação, a relação temporal entre a ocorrência da doença e o momento do parto e o risco pró-trombótico conferido pelas comorbidades adicionais. ^31^
- Nessa população, deve-se reforçar a vacinação, principal forma de prevenção de complicações relacionadas à COVID-19. As medidas de prevenção, como uso de máscara, higiene das mãos e evitar aglomeração, devem ser mantidas.

## Perspectivas futuras para a melhoria do cuidado cardiovascular das mulheres

- As taxas de mortalidade por DCV nas mulheres permanecem elevadas e estagnadas na maioria das regiões do mundo, com pequena ou nenhuma redução nos últimos anos. O RCV na mulher ainda é subestimado pela população em geral e, em especial, pelas próprias mulheres e pelos profissionais de saúde. A não implementação das diretrizes de prevenção de DCV em mulheres retarda o diagnóstico de várias DCV, em especial da cardiopatia isquêmica, que é frequentemente negligenciada nas mulheres. ^33^
- A compreensão clara das disparidades de sexo e de gênero na mortalidade prematura por DCV é essencial para o desenvolvimento de ações preventivas e de controle dessas doenças. A falta de estudos clínicos robustos e a sub-representação do sexo feminino nos ensaios clínicos contribuem para o escasso conhecimento sobre as DCV nas mulheres. ^34^ É mandatório investir em mais pesquisas sobre o papel dos hormônios sexuais no RCV global feminino.
- Reduzir a carga de DCV em mulheres até 2030 é uma meta ambiciosa, porém um imperativo, especialmente porque, em grande parte, os FR cardiovasculares podem ser modificados e mitigados.

## 2. Epidemiologia das Doenças Cardiovasculares nas Mulheres

**Figura 2.1:**
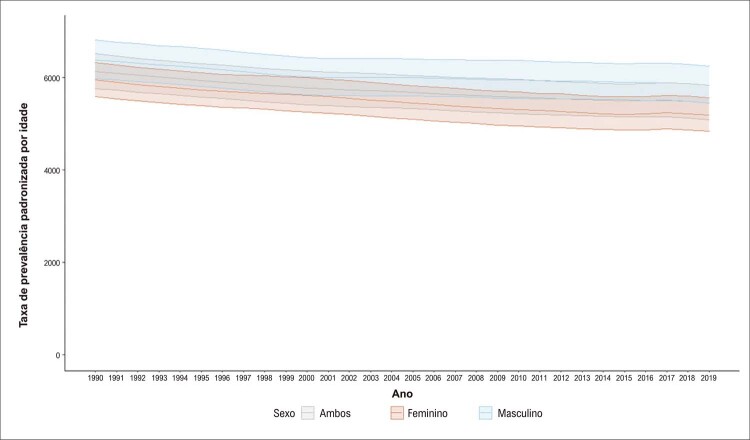
Taxas de prevalência de doença cardiovascular padronizadas por idade, por 100 mil habitantes, por sexo, Brasil, 1990-2019.

**Figura 2.2:**
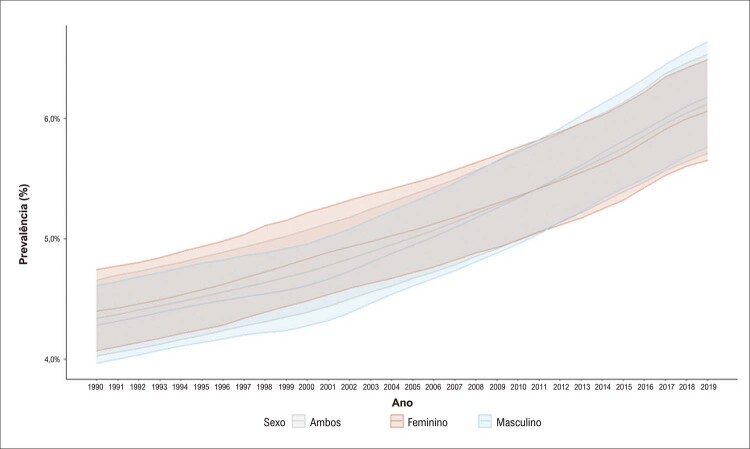
Prevalência de doença cardiovascular, por sexo, no Brasil, 1990-2019.

A prevalência de DCV, segundo o Estudo GBD 2019, foi de 6,1% da população em 2019, 12.946.932 (II 95%, 11.899.752 – 13.617.524) indivíduos, sendo 51% do sexo masculino. Os homens apresentaram maior taxa de prevalência padronizada por idade do que as mulheres em 2019 ( Figura 2.1 ). Entre 1990 e 2019, houve redução da taxa de prevalência de 8,7% nos homens, que foi menor do que a das mulheres, 12,8%. Houve aumento da prevalência de DCV nesse período nos jovens de 15-49 anos de ambos os sexos, bem como maior prevalência das DCV nas mulheres até o ano de 2011, a partir do qual a prevalência das DCV nos homens foi maior ( Tabela 2.1 e Figura 2.2 ). ^1 , 5^

Fonte: Estudo Global Burden of Disease (GBD) 2019. ^1^

**Tabela 2.1:** Número de casos e taxas de prevalência, incidência, mortes e DALYs de doença cardiovascular padronizadas por idade nas mulheres, por 100 mil habitantes, e variação percentual das taxas, por grupo etário, no Brasil, em 1990 e 2019.

Grupos Etários	1990		2019		Variação percentual (II 95%)

	Número (II 95%)	Taxa (II 95%)	Número (II 95%)	Taxa (II 95%)	
**PREVALÊNCIA**					
15-49 anos	81840.1 (71524.9;92783.6)	210.2 (183.7;238.3)	105700 (92430.3;120336.2)	180.7 (158.1;205.8)	-14 (-16.8;-10.9)
50-69 anos	102496.1 (91526.7;114514.7)	1255.7 (1121.3;1402.9)	208399.3 (186607.1;232143.7)	973.2 (871.4;1084)	-22.5 (-25;-19.8)
5-14 anos	26514.1 (17557.2;37666.8)	151.7 (100.4;215.5)	24476.1 (16014.6;34617.6)	154.6 (101.1;218.6)	1.9 (-1.4;5.7)
70+ anos	77895.9 (69702.8;86822.5)	3321.7 (2972.3;3702.4)	200343.1 (180509.1;222408)	2653.3 (2390.6;2945.5)	-20.1 (-22.9;-17.2)
Padronizada por idade	294962.9 (275518.3;317426.8)	557.5 (523.8;597.3)	544515.2 (512491.4;581529.1)	437.4 (411;468.6)	-21.5 (-23.3;-20)
Todas as idades	294962.9 (275518.3;317426.8)	391.9 (366;421.7)	544515.2 (512491.4;581529.1)	491.1 (462.3;524.5)	25.3 (21.7;29.1)
Abaixo de 5	6216.7 (4434;8521.4)	74.5 (53.1;102.1)	5596.8 (3974;7644)	73.8 (52.4;100.8)	-1 (-4;2.2)
**INCIDÊNCIA**					
15-49 anos	81840.1 (71524.9;92783.6)	210.2 (183.7;238.3)	105700 (92430.3;120336.2)	180.7 (158.1;205.8)	-14 (-16.8;-10.9)
50-69 anos	102496.1 (91526.7;114514.7)	1255.7 (1121.3;1402.9)	208399.3 (186607.1;232143.7)	973.2 (871.4;1084)	-22.5 (-25;-19.8)
5-14 anos	26514.1 (17557.2;37666.8)	151.7 (100.4;215.5)	24476.1 (16014.6;34617.6)	154.6 (101.1;218.6)	1.9 (-1.4;5.7)
70+ anos	77895.9 (69702.8;86822.5)	3321.7 (2972.3;3702.4)	200343.1 (180509.1;222408)	2653.3 (2390.6;2945.5)	-20.1 (-22.9;-17.2)
Padronizada por idade	294962.9 (275518.3;317426.8)	557.5 (523.8;597.3)	544515.2 (512491.4;581529.1)	437.4 (411;468.6)	-21.5 (-23.3;-20)
Todas as idades	294962.9 (275518.3;317426.8)	391.9 (366;421.7)	544515.2 (512491.4;581529.1)	491.1 (462.3;524.5)	25.3 (21.7;29.1)
Abaixo de 5	6216.7 (4434;8521.4)	74.5 (53.1;102.1)	5596.8 (3974;7644)	73.8 (52.4;100.8)	-1 (-4;2.2)
**MORTES**					
15-49 anos	769476.9 (739810.4;801490.1)	1976.3 (1900.1;2058.5)	679263.9 (631272.6;728617.3)	1161.5 (1079.5;1245.9)	-41.2 (-44.5;-37.8)
50-69 anos	1154563.6 (1117881.8;1194713.4)	14144.8 (13695.4;14636.7)	1485239.2 (1407973.4;1568445)	6935.6 (6574.8;7324.2)	-51 (-53.4;-48.4)
5-14 anos	38226.4 (34169.5;42955.7)	218.7 (195.5;245.7)	22398 (18744.9;26627.2)	141.4 (118.4;168.2)	-35.3 (-42.9;-28.7)
70+ anos	976778.1 (898018.1;1019807.9)	41652.6 (38294.1;43487.5)	1661643.2 (1472104.5;1785160.2)	22006.3 (19496.1;23642.2)	-47.2 (-50.2;-44.7)
Padronizada por idade	3017512.3 (2897630.7;3117428)	6191.2 (5895.3;6408.3)	3875201.6 (3604407.9;4099252.4)	3019.5 (2810.8;3195.6)	-51.2 (-53.4;-49.1)
Todas as idades	3017512.3 (2897630.7;3117428)	4008.9 (3849.7;4141.7)	3875201.6 (3604407.9;4099252.4)	3495.4 (3251.1;3697.5)	-12.8 (-17;-8.6)
Abaixo de 5	78467.3 (65203.6;97481.4)	940.3 (781.4;1168.2)	26657.3 (21657.1;32912.1)	351.4 (285.5;433.9)	-62.6 (-73.6;-49.5)
**DALYs**					
15-49 anos	769476.9 (739810.4;801490.1)	1976.3 (1900.1;2058.5)	679263.9 (631272.6;728617.3)	1161.5 (1079.5;1245.9)	-41.2 (-44.5;-37.8)
50-69 anos	1154563.6 (1117881.8;1194713.4)	14144.8 (13695.4;14636.7)	1485239.2 (1407973.4;1568445)	6935.6 (6574.8;7324.2)	-51 (-53.4;-48.4)
5-14 anos	38226.4 (34169.5;42955.7)	218.7 (195.5;245.7)	22398 (18744.9;26627.2)	141.4 (118.4;168.2)	-35.3 (-42.9;-28.7)
70+ anos	976778.1 (898018.1;1019807.9)	41652.6 (38294.1;43487.5)	1661643.2 (1472104.5;1785160.2)	22006.3 (19496.1;23642.2)	-47.2 (-50.2;-44.7)
Padronizada por idade	3017512.3 (2897630.7;3117428)	6191.2 (5895.3;6408.3)	3875201.6 (3604407.9;4099252.4)	3019.5 (2810.8;3195.6)	-51.2 (-53.4;-49.1)
Todas as idades	3017512.3 (2897630.7;3117428)	4008.9 (3849.7;4141.7)	3875201.6 (3604407.9;4099252.4)	3495.4 (3251.1;3697.5)	-12.8 (-17;-8.6)
Abaixo de 5	78467.3 (65203.6;97481.4)	940.3 (781.4;1168.2)	26657.3 (21657.1;32912.1)	351.4 (285.5;433.9)	-62.6 (-73.6;-49.5)

Fonte: Estudo Global Burden of Disease (GBD) 2019. Taxa/100 mil habitantes. ^1^ II: intervalo de incerteza.

Fonte: Estudo Global Burden of Disease (GBD) 2019. ^1^

Em 2019, no Brasil, as taxas de incidência de DIC, principalmente infarto do miocárdio, padronizadas por idade foram 78 e 148 por 100 mil habitantes em mulheres e homens, respectivamente. Em relação à DIC crônica (infarto do miocárdio prévio, angina estável ou IC isquêmica), as taxas de prevalência padronizadas por idade foram 1.046 e 2.534 por 100 mil em mulheres e homens, respectivamente ( Tabela 2.1 ).

**Figura 2.3:**
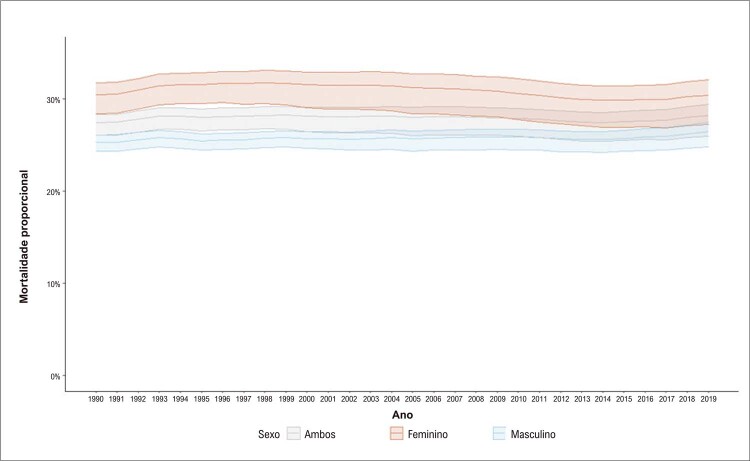
Mortalidade proporcional por doença cardiovascular, por sexo, Brasil, 1990-2019.

Na análise dos dados do GBD 2019, observa-se redução na taxa de mortalidade por DCV padronizada por idade para mulheres. No início do período, em 1990, existia acentuada diferença entre as regiões geográficas brasileiras, com redução da diferença das taxas de mortalidade ao final do período. Tal fato pode ser explicado por uma redução mais pronunciada no Sudeste e Sul, regiões que concentram as maiores populações e renda, e mais modesta no Norte e Nordeste ( Figuras 2.3 e 2.4 ). ^1 , 5^

Fonte: Estudo Global Burden of Disease (GBD) 2019. ^1^

**Figura 2.4:**
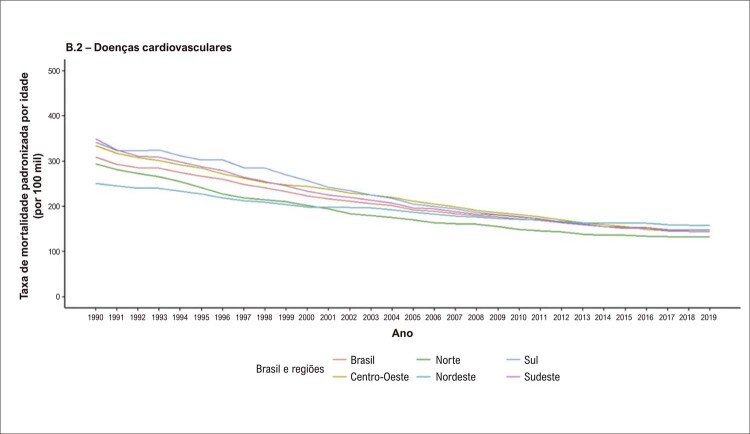
Taxas de mortalidade por doença cardiovascular padronizadas por idade, por 100 mil habitantes, nas mulheres, no Brasil e suas regiões, 1990-2019.

Fonte: Estudo Global Burden of Disease (GBD) 2019. ^1^

As DCV são a principal causa de morte no Brasil, em mulheres e homens, tendo diminuído 50,6% entre 1990 e 2019 ( Tabela 2.1 ). Embora as taxas de mortalidade padronizadas por idade fossem maiores nos homens em todo o período, a redução porcentual foi similar para ambos os sexos, 48% para homens e 52% para mulheres. A mortalidade proporcional por DCV foi maior nas mulheres durante todo o período de 1990 a 2019 ( Figura 2.3 ). ^1 , 5^

**Figura 2.5:**
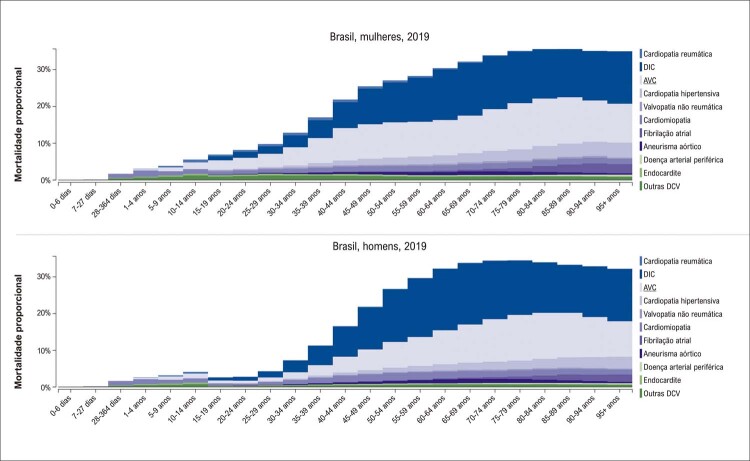
Mortalidade proporcional por doença cardiovascular nas mulheres (A) e nos homens (B), por faixa etária, no Brasil, 2019.

De acordo com as estimativas do Estudo GBD 2019, entre as DCV, a DIC foi a primeira causa de morte no Brasil, seguida pelo AVC. A DIC foi responsável por 12,03% e 12,2% dos óbitos em mulheres e homens, respectivamente, e o porcentual dos óbitos por AVC foi maior em mulheres do que em homens, 10,39% e 8,41%, respectivamente. Os DALYs estimados por DIC foram 1.276,6 (1.165,2;1.359) e 2.179,4 (2.054,4;2.296,3) e por AVC foram 1.235,6 (1.133,8;1.322,5) e 1.410,1 (1.323,5;1.487,9) em mulheres e homens, respectivamente ( Figuras 2.5 e 2.6 ). ^1 , 5^

Fonte: Estudo Global Burden of Disease (GBD) 2019. ^1^

**Figura 2.6:**
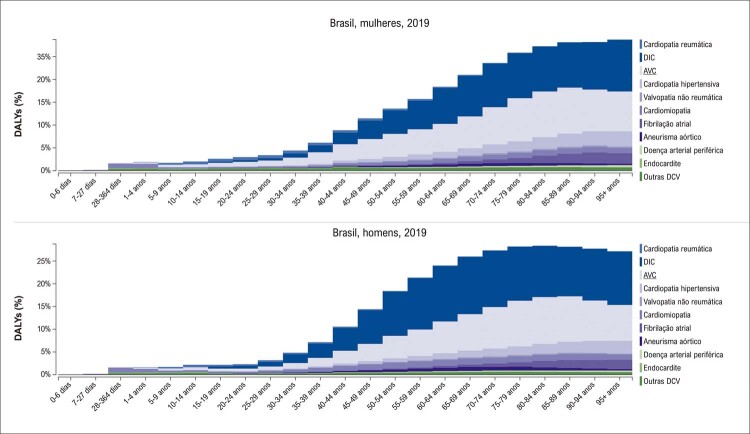
DALYs (%) por doença cardiovascular nas mulheres (A) e nos homens (B), por faixa etária, no Brasil, 2019.

Fonte: Estudo Global Burden of Disease (GBD) 2019. ^1^

A prevalência de FA e *flutter* atrial foi maior nos homens em 2019 do que nas mulheres, mas as mulheres apresentaram maior taxa de mortalidade e DALYs padronizada por idade em 2019. A FA ocorre associada com a DCR avançada, especialmente a estenose mitral, mais frequente em mulheres, na razão de 3 para 2.

A prevalência padronizada por idade de DCR apresentou discreto aumento de 2,1%, sendo mais alta nas mulheres (3,5%). No entanto, houve redução das taxas de mortalidade padronizadas por idade atribuíveis à DCR e a redução porcentual foi similar em ambos os sexos nos últimos 30 anos ( Figuras 2.5 e 2.6 ). Também as mulheres apresentaram maior mortalidade proporcional por estenose aórtica no Brasil em 2019. Observam-se ainda taxas decrescentes de DALYs entre 1990 e 2019, que foram similares para homens e mulheres ( Figuras 2.5 e 2.6 ). ^1 , 5^

Segundo dados do Sistema de Informação sobre Mortalidade do Ministério da Saúde, 1.185.120 óbitos ocorridos entre 1980 e 2018 tiveram a IC listada como sua causa básica (49,3% [584.155] em homens). ^35^ As razões entre as taxas brutas de mortalidade por IC em ambos os sexos, por grupo etário e região geográfica são mostradas na Tabela 2.2 . Observamos razões maiores ou iguais a 1 em quase todo o período, faixas etárias e regiões geográficas, mostrando maior mortalidade nos homens, exceto nas regiões Norte (1985-1989), Nordeste (1980-1984) e Sul na faixa etária de 60 anos e mais, onde a mortalidade das mulheres foi maior. ^35 , 36^

**Tabela 2.2:** Razão entre as taxas brutas de mortalidade nos sexos masculino e feminino, em grupos etários, em períodos de 5 anos, por região geográfica.

Faixa etária	Região / Período	1980-1984	1985-1989	1990-1994	1995-1999	2000-2004	2005-2009	2010-2014	2015-2018
**0-29**	Norte	1,0	0,9	1,2	1,0	1,2	1,3	1,3	1,8
	Nordeste	0,9	1,0	1,0	1,1	1,1	1,4	1,4	1,5
	Sudeste	1,1	1,2	1,3	1,3	1,2	1,6	1,5	1,8
	Sul	1,1	1,2	1,2	1,6	1,5	1,3	1,5	1,1
	Centro-Oeste	1,1	1,1	1,1	1,1	1,9	1,5	2,7	1,0
**30-59**	Norte	1,5	1,4	1,5	1,4	1,6	1,9	1,8	1,5
	Nordeste	1,2	1,3	1,4	1,3	1,3	1,4	1,5	1,6
	Sudeste	1,4	1,5	1,6	1,5	1,6	1,7	1,6	1,5
	Sul	1,4	1,5	1,5	1,4	1,4	1,5	1,4	1,2
	Centro-Oeste	1,2	1,5	1,6	1,6	1,8	1,9	1,7	1,7
**60+**	Norte	1,1	1,1	1,1	1,1	1,2	1,3	1,3	1,2
	Nordeste	1,2	1,2	1,2	1,1	1,2	1,2	1,2	1,2
	Sudeste	1,1	1,1	1,0	1,0	1,0	1,0	1,0	1,0
	Sul	1,1	1,0	1,0	0,9	0,9	0,9	0,9	0,9
	Centro-Oeste	1,1	1,1	1,0	1,0	1,1	1,1	1,1	1,2

Fonte: Sistema de Informação em Saúde-Datasus. ^35,36^

**Figura 2.7:**
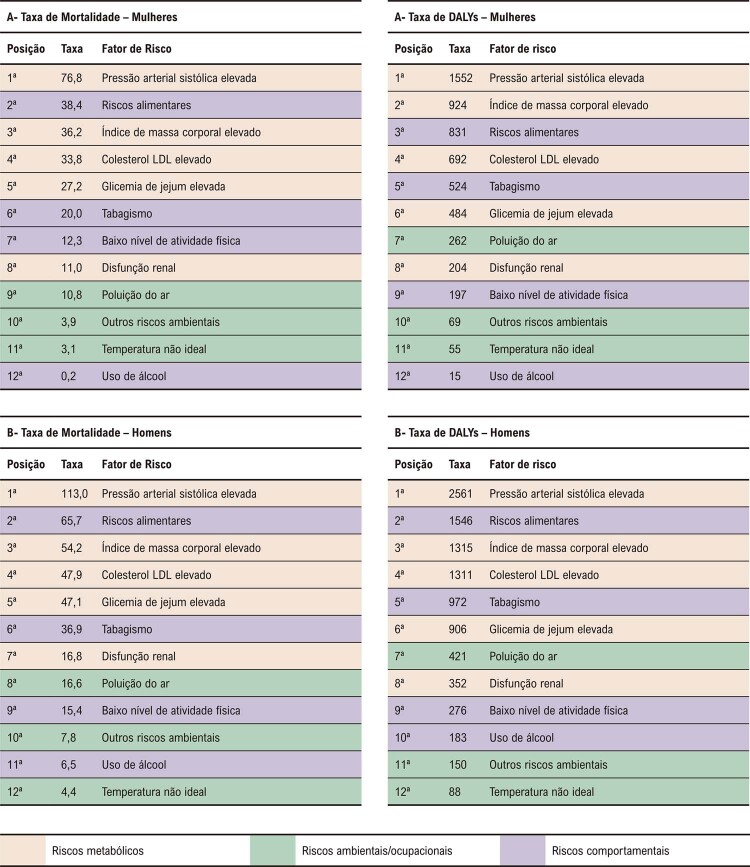
Ranking de taxas de mortalidade e de DALYs por doenças cardiovasculares atribuíveis a fatores de risco padronizadas por idade, em 2019, no Brasil, para mulheres (A) e homens (B). DALYs: anos de vida ajustados por incapacidade; DCV: doença cardiovascular; GBD: Global Burden of Disease; LDL: lipoproteína de baixa densidade.

Entre os FR para DCV em brasileiras, destacam-se HAS, riscos dietéticos, obesidade, aumento do colesterol sérico e glicemia de jejum elevada ( Figura 2.7 ). ^7^ O FR que mais aumentou no Brasil, de 1990 a 2019, foi o IMC elevado. ^1 , 7^ Os FR específicos nas mulheres com AVC incluem gravidez, pré-eclâmpsia, diabetes gestacional, uso de contracepção oral, uso de hormônios na menopausa e alterações no estado hormonal. ^7^

Fonte: Estudo Global Burden of Disease (GBD) 2019. ^1,5^

A prevalência de HAS autorreferida no Brasil em 2019 foi de 23,9%, maior no sexo feminino do que no masculino (26,4% *versus* 21,1%, respectivamente). ^37^ A mortalidade cardiovascular atribuída à HAS foi maior nas mulheres de 65 a 79 anos e nos homens de 50 a 79 anos. ^5^ Os riscos alimentares foram o segundo FR mais importante para DCV em 2019, respondendo por 5,0% e 5,7% das mortes por DIC e 2,6% e 2,4% das mortes por AVC em mulheres e homens, respectivamente. ^2^ A inatividade física, outro FR comportamental, aumentou de 1990 a 2019 no Brasil, com predomínio de mulheres, 4,7%, em relação aos homens, 3,1%. ^2^ Segundo dados do IBGE, no Brasil, em 2019, os porcentuais de adultos (idade ≥18 anos) com excesso de peso e obesidade foram 62,6% e 29,5% para mulheres e 57,5% e 21,8% para homens, respectivamente. Observou-se aumento progressivo da obesidade com o aumento da idade, com maior prevalência de excesso de peso e obesidade nas mulheres em todas as faixas etárias. ^5^ A prevalência de diabetes aumenta com o aumento da prevalência de obesidade. ^5^ Dados da PNS de 2014 a 2015, no Brasil, mostraram que a prevalência de diabetes foi maior em mulheres, indivíduos com idade superior a 30 anos e entre aqueles com sobrepeso ou obesidade. ^37^ O diabetes é um FR para DAC mais importante para as mulheres do que para os homens, mesmo entre mulheres na pré-menopausa. ^7 , 37^

Ansiedade, depressão e vitimização por violência foram estudadas em 31.847 mulheres provenientes da PNS de 2013. Os episódios depressivos maiores e a ideação suicida foram avaliados com o *Patient Health Questionnaire* e a vitimização por violência foi autorreferida. As mulheres apresentaram maiores prevalências de episódio depressivo (OR = 2,36; IC 95% 2,03-2,74), ideação suicida (OR = 2,02; IC 95% 1,73-2,36) e vitimização por violência (OR = 1,73; IC 95% 1,45-2,06). ^38^

**Figura 2.8:**
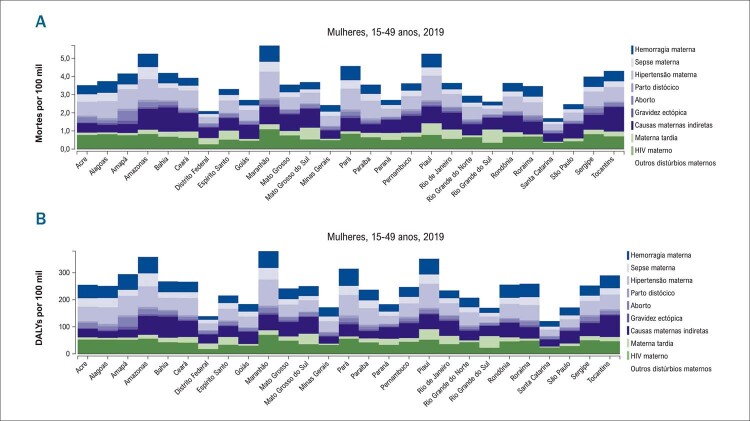
Taxas de mortalidade e DALYs por causas maternas nas unidades federativas, por 100 mil habitantes, nas mulheres, Brasil, 2019. Fonte: Estudo Global Burden of Disease (GBD) 2019. 1

Os fatores inerentes ao sexo são de fundamental importância e irão afetar a ocorrência de DCV ao longo da vida das mulheres. Estudo transversal multicêntrico com 27 maternidades de referência de todas as regiões do Brasil e 82.388 parturientes, de julho de 2009 a junho de 2010, identificou 9.555 casos de morbidade materna grave, 140 mortes e 770 casos de *near miss* materno. A principal causa determinante de complicação materna foi a doença hipertensiva. ^39^ Pré-eclâmpsia, diabetes gestacional, hipertensão induzida pela gravidez, parto prematuro e recém-nascido pequeno para a idade gestacional são considerados indicadores precoces de risco cardiovascular materno. Segundo dados do GBD 2019, as doenças hipertensivas da gravidez foram a segunda maior causa de mortalidade e DALYs nas mulheres em idade fértil ( Figura 2.8 ). ^1^

### 2.1. Doenças do Aparelho Circulatório em Crianças e Adolescentes do Sexo Feminino no Brasil

As DApC nos menores de 20 anos, no Brasil, possuem etiologias que variam de acordo com a faixa etária, o sexo e o local de residência. As MAC são a principal causa de óbito até 4 anos, sendo as cardiomiopatias a principal causa de 5 a 19 anos. ^40^

No Brasil, os óbitos por MAC no período de 2000 a 2015 apresentaram maior ocorrência entre os menores de 4 anos, principalmente no primeiro ano de vida e no sexo masculino ( Tabela 2.3 ). ^6^ No sexo feminino, as MAC com maior incidência foram persistência do canal arterial, anomalia de Ebstein da valva tricúspide e defeito do septo atrial tipo *ostium secundum* . As DApC ocorreram mais entre 5 anos e 19 anos, com predomínio no sexo feminino, provavelmente por competição das causas externas de óbito, mais prevalentes no sexo masculino. ^6^

**Tabela 2.3:** Mortalidade proporcional e taxa de mortalidade por grupo de causas em crianças, segundo sexo e grupo etário, Brasil, de 2000 a 2015. **6**

Causas de óbitos		<20 anos	Masculino						Feminino					

		Total	Total	<1ano	1-4 anos	5-9 anos	10-14 anos	15-19 anos	Total	<1ano	1-4 anos	5-9 anos	10-14 anos	15-19 anos
**MAC**	Óbitos	57.892	31.077	26.144	2.901	766	625	641	26.815	22.016	2.972	728	593	506
	MP(%)	4,2	3,7	6,2	4,1	1,8	1,1	0,3	5,15	6,6	5,0	2,4	1,7	0,8
	Mort100mil	5,3	5,62	107,0 _(1)_	2,7 _(2)_	0,6	0,4	0,5	5,0	94,7 _(1)_	2,9 _(2)_	0,5	0,4	0,4
**Outras MC**	Óbitos	86.165	45.237	39.715	3.133	956	725	708	40.928	35.729	3.009	870	720	600
	MP(%)	6,3	5,4	9,3	4,4	2,3	1,3	0,3	7,78	10,8	5,1	2,9	2,0	0,9
	Mort100mil	7,9	8,2	162,6 _(1)_	2,9 _(2)_	0,7	0,5	0,5	7,7	153,7 _(1)_	2,9 _(2)_	0,7	0,5	0,4
**DApC**	Óbitos	29.904	16.706	3.735	2.084	1.493	2.749	6.645	13.198	3.280	2.045	1.376	2.197	4.300
	MP(%)	2,2	2,0	0,9	2,9	3,6	4,8	2,6	2,54	1,0	3,4	4,5	6,2	6,6
	Mort100mil	2,8	3,0	15,3 _(1)_	1,9 _(2)_	1,1	2,0	4,7	2,5	14,1 _(1)_	2,0 _(2)_	1,0	1,6	3,1
**Mal definidas**	Óbitos	85.458	49.940	25.907	7.692	3.019	3.679	9.643	35.518	19.414	6.431	2.408	2.700	4.565
	MP(%)	6,2	5,9	6,1	10,9	7,2	6,5	3,8	6,82	5,9	10,8	7,9	7,7	7,0
	Mort100mil	7,9	9,0	106,0 _(1)_	7,0 _(2)_	2,2	2,6	6,8	6,7	83,5 _(1)_	6,2 _(2)_	1,8	2,0	3,3
**Externas**	Óbitos	340.974	274.627	10.816	16.304	16.384	29.287	201.836	66.347	7.431	10.328	8.801	11.992	27.795
	MP(%)	24,9	32,5	2,5	23,0	39,1	51,6	80,4	12,75	2,2	17,4	28,8	34,0	42,5
	Mort100mil	31,4	49,7	44,3 _(1)_	15,0 _(2)_	11,9	20,9	142,5	12,5	32,0 _(1)_	9,9 _(2)_	6,6	8,9	20,2
**Todas as causas**	Óbitos	1.367.355	845.481	424.932	70.854	41.904	56.775	251.016	521.874	331.269	59.430	30.518	35.293	65.364
	MP(%)	100,0	100,0	100,0	100,0	100,0	100,0	100,0	100,0	100,0	100,0	100,0	100,0	100,0
	Mort100mil	126,0	153,0	1.739,3 _(1)_	65,2 _(2)_	30,4	40,5	177,3	98,0	1424,7 _(1)_	57,0 _(2)_	23,0	26,1	47,6

MAC: malformações do aparelho circulatório; Outras MC: outras malformações congênitas excluindo as MAC; DApC: doenças do aparelho circulatório; MP(%): mortalidade proporcional em percentual; Mort100mil: taxa de mortalidade por 100mil (1) Mortalidade por 100 mil nascidos vivos (2) Mortalidade por 100 mil na população de 0 a 4 excluídos os nascidos vivos.

**Figura 2.9:**
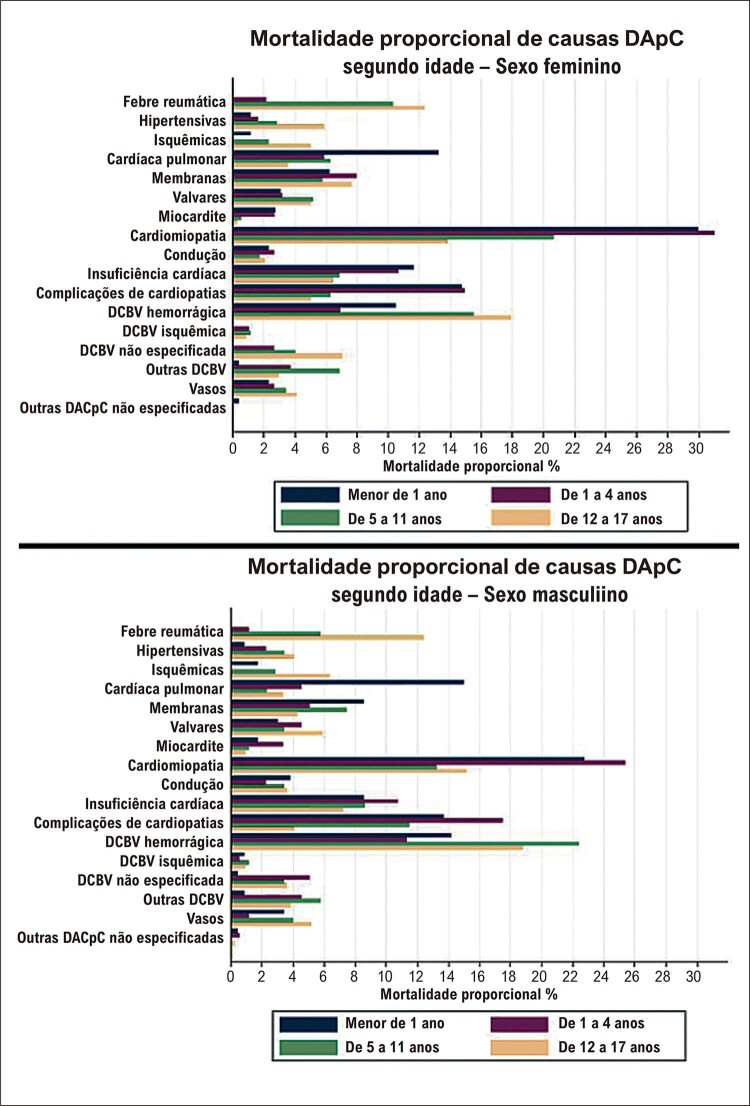
Mortalidade proporcional anual por causas específicas do aparelho circulatório em crianças e adolescentes, por sexo e grupo etário, no estado do Rio de Janeiro, de 1995 a 2012. 40 DApC: Doenças do Aparelho Circulatório, DCBV: Doenças Cerebrovasculares

Há aumento em importância dos óbitos por DApC com a progressão da idade ( Figura 2.9 ). ^6 , 40^ Devemos considerar que crianças que apresentam MAC, até mesmo corrigidas, e que não morreram no primeiro ano de vida poderão apresentar complicações e sequelas, como IC, arritmias, endocardite, com óbito na adolescência. Outra possível explicação é a maior negligência diagnóstica das MAC e DApC no sexo feminino em todas as faixas etárias no Brasil, desencadeada por menor acesso ao sistema de saúde e a recursos diagnósticos em comparação ao sexo masculino. ^3 , 6 , 40^

A Tabela 2.4 mostra a mortalidade proporcional atribuída a DApC e MAC nos menores de 1 ano por causas específicas e macrorregião brasileira. ^13^ Mais de 83% das mortes por DApC foram devidas a MAC em todas as regiões, com ênfase na região Sul, onde aquela porcentagem foi de

**Tabela 2.4:** Mortalidade proporcional por doenças do aparelho circulatório e malformações do aparelho circulatório, com subdivisão por causas específicas, em menores de 1 ano, sexo feminino, por macrorregião do Brasil, de 2000 a 2015. **41**

CAUSAS DE ÓBITOS		Norte	Nordeste	Sudeste	Sul	Centro-Oeste	Total
DOENÇA CARDÍACA PULMONAR E DA CIRCULAÇÃO PULMONAR	Óbitos	77	131	244	39	41	532
	MP (%)	**3,09**	**1,99**	**2,33**	**1,15**	**1,75**	**2,10**
PERICARDITE E ENDOCARDITE	Óbitos	13	32	69	9	14	137
	MP (%)	**0,52**	**0,48**	**0,66**	**0,27**	**0,60**	**0,54**
MIOCARDITES	Óbitos	3	14	49	23	4	93
	MP (%)	**0,12**	**0,21**	**0,47**	**0,68**	**0,17**	**0,37**
CARDIOMIOPATIAS	Óbitos	130	360	469	61	71	1091
	MP (%)	**5,22**	**5,46**	**4,47**	**1,80**	**3,02**	**4,31**
INSUFICIÊNCIA CARDÍACA	Óbitos	74	170	140	33	25	442
	MP (%)	**2,97**	**2,58**	**1,33**	**0,98**	**1,06**	**1,75**
DOENÇAS CEREBROVASCULARES E OUTROS VASOS	Óbitos	46	91	187	31	34	389
	MP (%)	**1,84**	**1,38**	**1,78**	**0,92**	**1,45**	**1,64**
OUTRAS DOENÇAS DO APARELHO CIRCULATÓRIO	Óbitos	83	188	243	37	45	596
	MP (%)	**3,33**	**2,85**	**2,32**	**1,09**	**1,92**	**2,36**
**SUBTOTAL DApC**	Óbitos	426	986	1401	233	234	3280
	MP (%)	**17,11**	**14,97**	**13,36**	**6,89**	**9,96**	**12,97**
CÂMARAS E SEPTOS	Óbitos	300	715	1943	630	370	3985
	MP (%)	**12,05**	**10,75**	**18,52**	**18,64**	**15,75**	**15,75**
VALVAS	Óbitos	87	196	691	293	138	1405
	MP (%)	**3,49**	**2,97**	**6,59**	**8,67**	**5,87**	**5,55**
NÃO ESPECIFICADAS	Óbitos	1284	3749	4666	1733	1324	12756
	MP (%)	**51,57**	**56,90**	**44,48**	**51,27**	**56,36**	**50,43**
OUTRAS	Óbitos	235	547	712	166	126	1786
	MP (%)	**9,57**	**8,30**	**6,79**	**4,91**	**5,36**	**7,06**
VASOS	Óbitos	158	395	1049	325	157	1727
	MP (%)	**6,34**	**5,99**	**10,00**	**9,62**	**6,68**	**8,24**
SUBTOTAL MAC	Óbitos	2064	5602	7859	3147	2115	22016
	MP (%)	**82,89**	**85,04**	**74,93**	**93,11**	**90,04**	**87,03**
**TOTAL DApC + MAC**	Óbitos	2490	6588	10489	3380	2349	25296
	MP (%)	**100,00**	**100,00**	**100,00**	**100,00**	**100,00**	**100,00**

DApC: doenças do aparelho circulatório; MAC: malformações do aparelho circulatório; MP (%): mortalidade proporcional em percentual.

93%. As MAC não especificadas corresponderam à metade das mortes dos menores de 1 ano no Brasil, sem distinção entre os sexos, com predomínio na região Sudeste (44%). A mortalidade proporcional por DApC foi 2,5 vezes maior na região Norte do que na região Sul, com predomínio no sexo feminino. A cardiomiopatia representou 32% das mortes por DApC, emergindo como a principal causa de morte, em todas as regiões, em ambos os sexos. ^41^

Na última década o aumento da obesidade, resistência insulínica e HAS em crianças e adolescentes foi um fator que contribuiu para o aumento do risco cardiovascular em jovens. Estima-se que nos próximos anos ocorra um incremento da prevalência da DApC nessa população. ^26 , 42^

As meninas e adolescentes do sexo feminino morreram mais de DApC do que aqueles do sexo masculino, o que demanda estratégias de saúde pública voltadas para elas, como a equidade de acesso aos recursos de saúde, o diagnóstico precoce e a instituição de medidas terapêuticas específicas para o sexo feminino.

## 3. Fatores de Risco Cardiovascular

### 3.1. Introdução

Nas últimas décadas, segundo dados do GBD 2019, houve uma redução global da taxa de mortalidade por DCV, com tendência a estagnação nos últimos anos. ^1^ Nas mulheres, na faixa etária de 35-54 anos, houve crescente aumento na taxa de mortalidade por DIC nos Estados Unidos da América, ^8 , 43^ atribuído à maior prevalência dos FRCV tradicionais.

Segundo dados do Estudo GBD 2019, para a mulher brasileira ( Figura 2.7 ), no *ranking* de taxas de mortalidade e de DALYs por DCV atribuíveis a fatores de risco, padronizadas por idade, destacaram-se em ordem decrescente os seguintes fatores de risco: elevação da pressão arterial sistólica, IMC elevado, riscos alimentares, colesterol LDL elevado, tabagismo e glicemia de jejum elevada. Importante ressaltar o aumento da prevalência de DCV nas mulheres jovens de 15-49 anos, entre 1990 e 2019. ^7 , 44^

**Figura 3.1:**
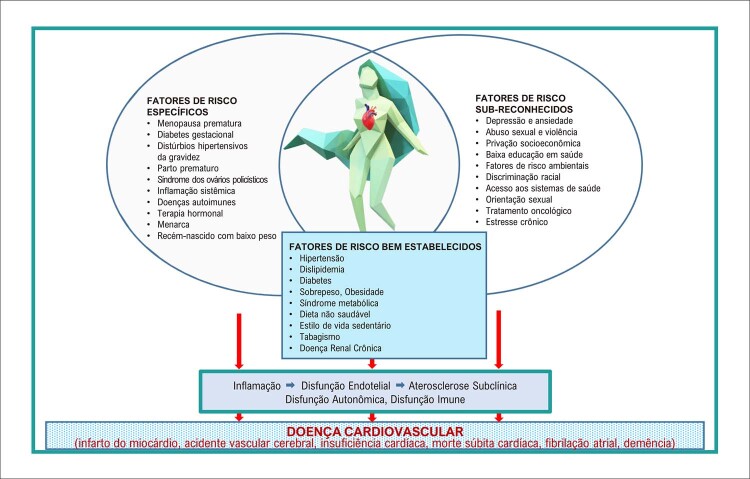
Fatores de risco para doença cardiovascular nas mulheres. Os fatores de risco bem estabelecidos, comuns a ambos os sexos, são incorporados nos escores de risco da doença cardiovascular aterosclerótica. Porém, os fatores de risco específicos do sexo e os fatores de risco sub-reconhecidos interagem com os tradicionais, agregando risco especialmente nas mulheres.

**Figura 3.2:**
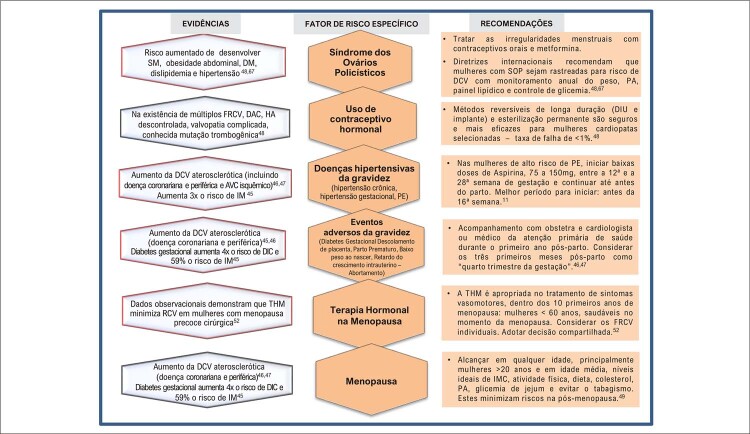
Fatores de risco específicos do sexo feminino para doenças cardiovasculares, com evidências e recomendações. 11,45-49 AVC: acidente vascular cerebral; DAC: doença arterial coronariana; DCV: doença cardiovascular; DIC: doença isquêmica do coração; DIU: dispositivo intrauterino; DM: diabetes mellitus; FRCV: fatores de risco cardiovascular; HA: hipertensão arterial; IM: infarto do miocárdio; IMC: índice de massa corporal; PA: pressão arterial; PE: pré-eclâmpsia; RCV: risco cardiovascular; SM: síndrome metabólica; SOP: síndrome de ovários policísticos; THM: terapia hormonal da menopausa.

Com relação ao risco de DCV aterosclerótica, as mulheres compartilham alguns FRCV tradicionais com os homens, enquanto outros FRCV são sub-reconhecidos nas mulheres ( Quadro 3.1 e Figura 3.1 ) ^2^ e outros ainda são específicos do sexo/gênero feminino ( Figura 3.2 ).

**Quadro 3.1:** Agravantes do risco cardiovascular do diabetes mellitus na mulher. **50**

• Idade > 56 anos nas mulheres e tempo de diagnóstico do diabetes >10 anos
• História familiar: parente de primeiro grau com DCV prematura (homens <55 anos e mulheres <65 anos)
• Tabagismo
• Hipertensão arterial sistêmica
• Síndrome metabólica
• Albuminúria >30 mg/g de creatinina
• Taxa de filtração glomerular <60 ml/min
• Retinopatia não proliferativa
• Neuropatia autonômica cardiovascular incipiente

Nos ensaios clínicos, apesar da importância da relação dos FRCV com DCV, as mulheres são pouco representadas e os escores para estratificação de risco cardiovascular utilizados, Framingham, SCORE ( *Systematic COronary Risk Evaluation* ), SBC/SBD/SBEM, não contemplam os FRCV específicos do sexo feminino, tais como ( Figura 3.2 ): SOP, uso de contraceptivo hormonal, DHG, eventos adversos da gravidez, terapia hormonal na menopausa, riscos agregados ao *status* socioeconômico, psicossocial e ambiental. Além disso, os escores não contemplam a associação com doenças inflamatórias (HIV) e autoimunes (artrite reumatoide, lúpus eritematoso sistêmico, psoríase), ^8^ distúrbios ansiosos-depressivos e DSS, considerados FPR nas mulheres.

**Figura 3.3:**
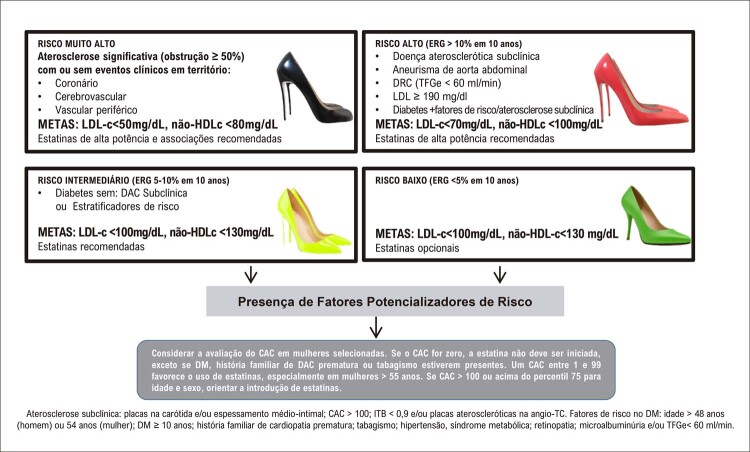
Algoritmo para avaliação do risco cardiovascular nas mulheres e recomendação do uso de estatinas. 53 angio-TC: angiotomografia coronariana; CAC: escore de cálcio coronariano; DAC: doença arterial coronariana; DM: diabetes mellitus; DRC: doença renal crônica; ERG: Escore de Risco Global; ITB: índice tornozelo-braquial; TFGe: taxa de filtração glomerular estimada.

Torna-se fundamental considerar na avaliação do risco cardiovascular, além dos estratificadores de risco ( Figura 3.3 ), os agravantes de risco associados com DM ( Quadro 3.2 ), os FPR e os fatores de risco inerentes ao sexo/gênero. Desse modo, mudanças do estilo de vida podem ser instituídas, além da recomendação de medidas de prevenção primária das DCV, com o objetivo de identificar e tratar mais precocemente um maior número de mulheres em risco. ^7 , 8 , 44^

**Quadro 3.2:** Fatores de risco para doença cardiovascular mais prevalentes nas mulheres, além dos fatores de risco semelhantes em homens e mulheres, com evidências. **11,43,45,53,55–58,65–67**

Fatores de risco e doenças	> risco para DCV em mulheres	Evidências
**Diabetes mellitus**	**X**	Hipertensão e dieta inadequada estão associadas a alto risco de pré-diabetes. Mulheres com DM têm 45% maior risco de DIC. ^45,67^ DM2 mais comum em mulheres < 40 anos com maior mortalidade ao longo da vida. Risco relativo de mortalidade por DCV maior em mulheres do que em homens, como também o excesso de risco de mortalidade por DCV é maior em mulheres. ^45,67^ Eventos ateroscleróticos acarretam mais IC congestiva nas mulheres. ^67^
**Hipertensão arterial sistêmica**	**X**	Maior risco de infarto do miocárdio (INTERHEART). ^67^ Maior prevalência de hipertensão secundária na pré-menopausa. Mulheres na pós-menopausa têm menos descenso noturno e mais eventos. Mulheres desenvolvem mais hipertrofia ventricular, ICFEp, rigidez arterial, DRC e DM. Mulheres apresentam mais efeitos colaterais com os anti-hipertensivos. Ocorre em 80% das mulheres > 75 anos. Apenas 29% têm controle adequado da HAS. ^45^
Dislipidemia (*)		No SWAN, colesterol total, LDL-c e triglicérides tiveram pico na transição da menopausa para a fase precoce após a menopausa. ^65^ Recente meta-análise não demonstrou diferenças significativas de HDL-C entre a pré- e pós-menopausa. ^61^
Obesidade (*)		No Framingham Heart Study, houve aumento de risco de DAC: 64% nas mulheres versus 46% nos homens. ^45^ Mais prevalente nas mulheres brasileiras do que nos homens. FR mais importante para HAS nas mulheres. Associada com desfechos adversos na gravidez.
Sedentarismo (*)		Maior prevalência de inatividade nas mulheres. 25% das mulheres não praticam atividade física regular. ^43,45^
Tabagismo (*)		As mulheres têm 25% maior risco de DAC em comparação aos homens, exceto na faixa etária mais jovem (30-44 anos). ^43,67^
**Fibrilação Atrial**	**X**	Em comparação aos homens com FA, as mulheres têm 20% a 30% maior risco de AVC, maior gravidade e pior resultado a longo prazo em termos de incapacidade permanente. O sexo feminino foi associado à idade >65 anos ou >2 fatores de risco no CHA2DS2-VASc em FA não valvar. ^67^
Síndrome Metabólica (*)		No estudo SWAN, os riscos de SM em mulheres na pré-menopausa e menopausa são 1,45x e 1,25x maiores, respectivamente. ^65^
**Artrite Reumatoide**		Aumento de 50% no risco de mortalidade por DCV em comparação à população geral. ^67^
**Lúpus Eritematoso Sistêmico**		Três vezes maior o risco de DIC. ^45^ Prevalente em asiáticas, afro-americanas, afro-caribenhas e hispânicas em comparação com caucasianas. As mulheres negras são 2-4x mais propensas a ter LES do que as mulheres brancas. A DIC é a causa principal de mortalidade de LES. ^67^
Depressão (**)	**X**	Comum em portadoras de DIC. Fator de risco independente para piores desfechos. ^56^ Mulheres são mais suscetíveis, com aumento do risco de DIC em 2x. ^45^
**Estresse Mental (ansiedade, estresse psicossocial) Exaustão vital**	**X**	Estudos demonstram associação entre estresse mental, disfunção microvascular e vasoespasmo coronarianos em pacientes com INOCA, ^58^ principalmente nas mulheres.
**Determinantes Sociais de Saúde**		DSS geram desfechos negativos que, somados as FRCV clássicos e específicos do sexo/gênero, promovem inflamação vascular e disfunção endotelial, culminando em DCV. ^59^

(*) fatores de risco semelhantes em ambos os sexos; (**) Questões básicas: Como você acha que as coisas estarão com sua saúde no futuro? Com que frequência você sente prazer ou felicidade em sua vida? Você já se sentiu grato por sua saúde, já se sentiu grato por outras coisas em sua vida? - analisam otimismo, afeto positivo, gratidão, respectivamente, ^56^ e podem auxiliar o reconhecimento e a abordagem específica. AVC: acidente vascular cerebral; BRA: bloqueador de receptor de angiotensina; CT: colesterol total; DAC: doença arterial coronariana; DIC: doença isquêmica do coração; DM: diabetes mellitus; DM2: diabetes mellitus tipo 2; DCV: doença cardiovascular; DOAC: anticoagulantes de ação direta; DRC: doença renal crônica; DSS: determinantes sociais de saúde; FA: fibrilação atrial; FR: fator de risco; FRCV: fatores de risco cardiovascular; HAS: hipertensão arterial sistêmica; HDL-c: colesterol da lipoproteína de alta densidade; IC: insuficiência cardíaca; ICFEp insuficiência cardíaca com fração de ejeção preservada; IECA: inibidor da enzima de conversão da angiotensina; IMC: índice de massa corporal; INOCA: isquemia na ausência de obstrução arterial coronariana; LES: lúpus eritematoso sistêmico; LDL-c: colesterol da lipoproteína de baixa densidade; SM: síndrome metabólica.

A estimativa do risco de eventos cardiovasculares na mulher para prevenção primária é limitada. A diretriz do ACC/AHA identifica situações particulares em mulheres aparentemente saudáveis com FPR, nas quais a decisão sobre a utilização de estatina de média potência para risco baixo ou intermediário deve ser compartilhada entre cardiologista, gineco-obstetra e paciente. Não há evidência claramente estabelecida sobre o uso de estatinas e será necessário considerar a avaliação do CAC em mulheres selecionadas. Se o CAC for zero, a estatina não deve ser iniciada, exceto na presença de: DM, história familiar de DAC prematura ou tabagismo. O CAC entre 1 e 99 favorece o uso de estatinas, especialmente em mulheres > 55 anos. Se o CAC > 100 ou acima do percentil 75 para idade e sexo, deve-se orientar a introdução de estatinas ( Figura 3.3 ). ^51 , 52^

A Diretriz Brasileira de Dislipidemias e Prevenção da Aterosclerose - 2017 ^53^ e a Atualização da Diretriz de Prevenção Cardiovascular da SBC - 2019 ^26^ recomendam o Escore de Risco Global, ^54^ que estima o risco em 10 anos de infarto do miocárdio e AVC fatais ou não fatais, insuficiência cardíaca e insuficiência vascular periférica. A Figura 3.3 sugere algoritmo para a avaliação do risco cardiovascular nas mulheres e a recomendação do uso de estatinas.

Os distúrbios ansiosos-depressivos, sejam alterações de humor, comportamentos, afeição, acompanhados ou não de alterações somáticas ou déficit de cognição, são causas comuns de incapacidade em países desenvolvidos e são considerados FPR pela AHA/ACC e a Sociedade Europeia de Cardiologia. Mulheres são mais suscetíveis aos distúrbios ansiosos-depressivos, que aumentam duas vezes o risco de DIC. Alterações comportamentais, hormonais, genéticas e psicossociais se sobrepõem e, do ponto de vista fisiopatológico, promovem disfunção endotelial, aterotrombose, disfunção do sistema imunológico e da hemostasia a partir de alterações no eixo hipotálamo-hipófise-adrenal e do sistema nervoso autônomo ( Figura 3.1 ). Ademais, os estrogênios estimulam os linfócitos T e B, propiciando uma maior resposta imune e inflamatória. Essas sobreposições justificam a relação entre a depressão e as DCV, principalmente a DIC. Nesse contexto, o exercício físico tem sido reconhecido como eficaz no tratamento da depressão, à semelhança da psicoterapia e dos inibidores da serotonina. Os exercícios aeróbicos parecem promover melhor benefício. ^55^

Em relação aos DSS, a diversidade de raça e etnicidade, nível de escolaridade, educação, racismo e discriminação, inacessibilidade aos sistemas assistenciais de saúde, ausência de suporte social, instabilidade econômica, orientação e violência sexual são fatores que conduzem às disparidades, atingem a mulher e geram desfechos negativos, que somados aos FRCV clássicos e específicos do sexo/gênero, promovem inflamação vascular e disfunção endotelial culminando em DCV ( Figura 3.1 ). ^26 , 59^

Estudo transversal para calcular o risco cardiovascular, utilizando dados laboratoriais de uma subamostra da PNS com 3.584 mulheres, encontrou 58,4% com baixo risco cardiovascular, 32,9% com risco médio e 8,7% com risco alto. O risco aumentou com a idade e foi elevado na população com baixa escolaridade. A proporção dos componentes do modelo de Framingham, por grupos de risco e sexo, mostra que, no risco elevado entre mulheres, os indicadores que mais contribuíram para o risco cardiovascular foram: pressão arterial sistólica, colesterol total, HDL, DM e tabagismo. ^64^

Os Quadros 3.3 , 3.4 , 3.5 , 3.6 , 3.7 e 3.8 mostram as recomendações para o manejo de dislipidemia, diabetes, síndrome metabólica, tabagismo, sobrepeso e obesidade, hipertensão arterial e atividade física nas mulheres.

**Quadro 3.3:** Recomendações para o manejo da dislipidemia nas mulheres.

Recomendações para o manejo da dislipidemia nas mulheres			

Recomendação	CR	NE	Referências
A terapia nutricional, a perda de peso e a prática de atividade física devem ser recomendadas a todos os pacientes		**A**	^26,43,51,67^
Reduzir o consumo de sódio e manter adequado consumo de frutas frescas, hortaliças e produtos lácteos com baixo teor de gordura. Manter peso corpóreo e a medida da cintura dentro da normalidade		**A**	^26,51,53,67^
Pelo menos 30 minutos de exercício aeróbico dinâmico de intensidade moderada (caminhada, corrida, ciclismo ou natação) em 5 a 7 dias por semana. Aumento gradual da atividade física aeróbica para 300 minutos por semana de intensidade moderada ou 150 minutos por semana de atividade física aeróbica de intensidade vigorosa, ou uma combinação equivalente dos mesmos, idealmente com exercício diário supervisionado		**A**	^26,43,51,67^
Mulheres de muito alto risco e alto risco cardiovascular: o LDL-c deve ser reduzido para < 50 mg/dL e < 70 mg/dL e o não HDL-c para < 80 mg/dL, e < 100mg/dl, respectivamente		**B**	^51,52,53,^
Mulheres de risco cardiovascular intermediário: o LDL-c deve ser reduzido para < 100 mg/dL e o não HDL-c para < 130 mg/dL		**A**	^51,52,53^
Mulheres de baixo risco cardiovascular: a meta de LDL-c deve ser < 130 mg/dL e o não HDL-c < 160 mg/dL		**A**	^51,52,53^
Não é recomendado tratamento medicamentoso nas gestantes	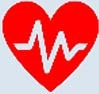	**A**	^11,43,47,67^
Os sequestrantes de ácidos biliares são considerados seguros para uso durante a amamentação	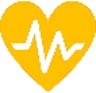	**B**	^11,43,47,67^
Rosuvastatina e pravastatina em baixas doses foram estudadas e podem ser consideradas durante a amamentação se os benefícios superarem os riscos potenciais	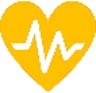	**B**	^11,43,47,67^

CR: classe de recomendação; NE: nível de evidência; HDL-c: colesterol da lipoproteína de alta densidade; LDL-c: colesterol da lipoproteína de baixa densidade. O tempo de reavaliação após o tratamento medicamentoso deve ser de pelo menos um mês. Adaptado da Atualização da Diretriz de Dislipidemias e Prevenção da Aterosclerose. ^53^ Semelhantemente ao uso de medicamentos durante a gravidez, os autores deste artigo recomendam a tomada de decisão compartilhada entre paciente e clínico para determinar o curso clínico ideal para pacientes individuais.

**Quadro 3.4:** Recomendações para o manejo de diabetes e síndrome metabólica nas mulheres.

Recomendações para o manejo de diabetes e síndrome metabólica nas mulheres			

Recomendação	CR	NE	Referências
As estratégias de controle de peso, atividade física, orientação dietética e cessação do tabagismo devem ser oferecidas a todas as mulheres com intolerância a glicose, SM ou DM, de maneira a reduzir o risco CV		**A**	^26,43,50,51,52,67^
Não é recomendável estratificar o risco de eventos coronários por meio de métodos anatômicos ou funcionais em mulheres assintomáticas com SM ou DM	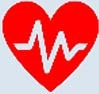	**A**	^26,43,50,51,52,67^
Recomenda-se o uso de CAC em mulheres com DM ou SM e risco CV intermediário (ERG 5-10% em 10 anos). Com CAC = 0, em geral, recomenda-se não iniciar estatina		**B**	^26,43,50,51,52,53,67^
Em prevenção primária, mulheres diabéticas ou com SM cuja terapia com estatina está indicada devem receber doses de alta potência dessas e/ou de ezetimiba, com alvo de LDL-c < 70 mg/dL		**A **	^26,43,50, 51,52,67^
Alternativamente, em mulheres com DM ou SM de risco elevado ou muito elevado, uma meta de LDL-c < 50 mg/dL deve ser buscada		**B**	
O uso de AAS não é recomendado como estratégia de prevenção primária em mulheres com SM ou DM, independentemente do risco CV	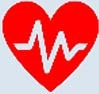	**A**	^26,43,50,51,53,67^
O uso de AAS é recomendado como estratégia de prevenção em mulheres com SM ou DM, com alto e muito alto risco, na ausência de contraindicação ou risco de sangramento	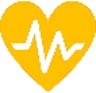 a	**B**	^26,43,50,51,52,67^
Em relação ao tratamento farmacológico, não há diferença nas recomendações quanto ao gênero, exceto os relacionados com gravidez		**A**	^11,26,43,50,51,52,67^

CR: classe de recomendação; NE: nível de evidência; AAS: ácido acetilsalicílico; CAC: escore de cálcio coronariano; CV: cardiovascular; DM: diabetes mellitus; ERG: escore de risco global; LDL-c: colesterol da lipoproteína de baixa densidade; SM: síndrome metabólica.

**Quadro 3.5:** Recomendações para o manejo do tabagismo nas mulheres.

Recomendações para o manejo do tabagismo nas mulheres			

Recomendação	CR	NE	Referências
Avaliação rotineira do tabagismo e exposição ao tabaco para mulheres em todas as consultas dos profissionais de saúde, com registro no prontuário médico		**A**	^26,43,51,52,67^
Aconselhamento sistemático de todas as mulheres para a cessação do tabagismo		**A**	^26,43,51,52,67^
Recomenda-se para todas as mulheres uma combinação de intervenções cognitivo-comportamentais e farmacológicas para maximizar as taxas de abandono		**A**	^26,43,51,52,67^
Recomenda-se a abstinência do fumo para todas as mulheres com o objetivo de reduzir os riscos cardiovasculares		**A**	^26,43,51,52,67^
Deve-se alocar equipe multidisciplinar para facilitar a cessação do fumo, em todos os sistemas de saúde	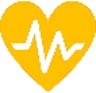 a	**B**	^26,43,51,52,67^

CR: classe de recomendação; NE: nível de evidência.

**Quadro 3.6:** Recomendações para o manejo de sobrepeso e obesidade nas mulheres

Recomendações para o manejo do sobrepeso e obesidade nas mulheres			

Recomendação	CR	NE	Referências
Em mulheres com sobrepeso e obesidade, a perda de peso é recomendada para melhorar o perfil de risco cardiovascular		**B**	^26,43,51,52,67^
Aconselhamento e intervenções abrangentes no estilo de vida, incluindo restrição calórica, são recomendados para alcançar e manter a perda de peso nas mulheres com sobrepeso e obesidade Enfatizar a relação de sobrepeso e obesidade com aumento de risco cardiovascular		**B**	^26,43,51,52,67^
Calcular o índice de massa corporal e realizar medidas antropométricas em consultas médicas para identificar mulheres com sobrepeso e obesidade com objetivo de intervenção		**C**	^26,43,51,52,67^
Para o tratamento, sugere-se equipe multidisciplinar, considerando-se tratamento farmacológico e cirurgia bariátrica quando não se consegue a redução adequada. Empregar medicamentos se IMC > 30 ou >27 na presença de comorbidades		**B**	^26,43,51,52,67^
Avaliar a circunferência da cintura para identificar as mulheres com maior risco cardiometabólico	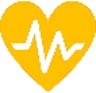 a	**B**	^26,43,51,52,67^
Não se recomenda o uso de fármacos para diminuição de peso na gestação e durante a amamentação. Deve-se dar especial atenção a mulheres no período fértil	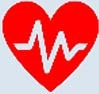	**B**	^26,43,51,52,67^

CR: classe de recomendação; NE: nível de evidência; IMC: índice de massa corporal.

**Quadro 3.7:** Recomendações para o manejo da hipertensão arterial nas mulheres.

Recomendações para o manejo da hipertensão arterial nas mulheres			

Recomendação	CR	NE	Referências
Para todas as mulheres com PA elevada ou hipertensão, medidas não farmacológicas são indicadas para reduzir a PA: perda de peso, padrão alimentar saudável, redução de sódio, suplementação dietética de potássio, aumento da atividade física com um programa estruturado de exercícios e ingesta limitada de álcool		**A**	^26,43,51,52,67^
Recomenda-se medicação anti-hipertensiva para mulheres com risco estimado ≥ 5% em 10 anos e PA sistólica média ≥ 130 mmHg ou PA diastólica média ≥ 80 mmHg, para prevenção primária de DCV		**A**	^26,43,51,52,67^
Recomenda-se, para mulheres com hipertensão confirmada e risco CV ≥ 10%, PA alvo < 130/80 mmHg		**B**	^26,43,51,52,67^
Recomenda-se, para mulheres com hipertensão arterial e doença renal crônica, meta pressórica < 130/80 mmHg		**B**	^26,43,51,52,67^
Recomenda-se, para mulheres com hipertensão arterial e diabetes tipo 2, meta pressórica < 130/80 mmHg, devendo medicação anti-hipertensiva ser iniciada se PA ≥ 130/80 mmHg		**B**	^26,43,51,52,67^
Recomenda-se medicação anti-hipertensiva para mulheres com risco estimado < 10% em 10 anos e PA ≥ 140/90 mmHg, para prevenção primária de DCV		**C**	^26,43,51,52,67^
Em mulheres com hipertensão confirmada sem marcadores adicionais de aumento do risco CV, a meta pressórica < 130/80 mmHg é recomendada	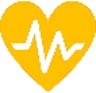 b	**B**	^26,43,51,52,67^
Não utilizar IECA ou BRA em mulheres com idade fértil e perspectivas de engravidar por seus possíveis efeitos teratogênicos	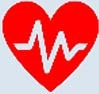	**A**	^26,43,51,52,67^
A hipertensão secundária é comum em mulheres jovens e idosas, devendo-se rastrear adolescentes e adultos jovens com hipertensão, para prevenir complicações CV a longo prazo e iniciar tratamento específico		**A**	^26,43,51,52,67^

CR: classe de recomendação; NE: nível de evidência; BRA: bloqueador de receptor de angiotensina; CV: cardiovascular; DCV: doença cardiovascular; IECA: inibidor da enzima de conversão da angiotensina; PA: pressão arterial.

**Quadro 3.8:** Recomendações para o manejo da atividade física nas mulheres.

Recomendações para atividade física nas mulheres			

Recomendação	CR	NE	Referências
Durante as consultas, médicos devem aconselhar seus pacientes para as práticas de atividade física		**B**	^26,43,51,52,67^
Atividade física semanal ≥ 150 minutos de exercício em intensidade moderada ou 75 minutos de exercícios mais intensos reduz o risco cardiovascular		**A**	^26,43,51,52,67^
Atividade física semanal < 150 minutos de exercício de intensidade moderada ou < 75 minutos de exercícios mais intensos reduz o risco cardiovascular	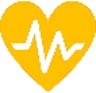 a	**B**	^26,43,51,52,67^
Atividade física semanal > 150 minutos de exercício de intensidade moderada durante toda a gravidez sem complicações		**A**	^26,43,51,52,67^

CR: classe de recomendação; NE: nível de evidência.

## 4. Doenças Cardiovasculares nas Mulheres

### 4.1. Doença Isquêmica do Coração

Com relação às mortes por DCV em mulheres, a DIC corresponde a 47% e o AVC, a 36%. ^1^ As particularidades da DIC em relação a fisiopatologia, apresentação clínica, diagnóstico, tratamento e prevenção estão condensadas na Tabela 4.1 .

**Tabela 4.1:** Características gerais da doença isquêmica do coração.

	DAC ^**70**^	INOCA ^**69**^	MINOCA ^**71**^	DEAC	Vasoespasmo ^**72**^	Doença Microvascular ^**70**^	Trombose/ Embolia Coronária ^**73**^
**Fisio patologia**	- Placa aterosclerótica menos volumosa - Erosão da placa com embolização distal - Aterosclerose difusa associada a disfunção microvascular e endotelial	Disfunção microvascular e/ou vasoespasmo epicárdico e microvascular	Vasoespasmo coronariano, disfunção microvascular, trombose coronariana espontânea e êmbolos com recanalização, rotura da placa e dissecção espontânea da artéria coronária	- Separação não traumática, não iatrogênica e não aterosclerótica da parede da artéria coronária, seja por ruptura espontânea da íntima ou por ruptura dos vasa vasorum dentro da parede do vaso - Formação de hematoma intramural na falsa luz, compressão da luz verdadeira, causando isquemia ou infarto do miocárdio - Estresse de cisalhamento aumentado - Gatilhos emocionais/físicos	- Vasoconstrição epicárdica ou microvascular relacionada à disfunção endotelial - Múltiplos mecanismos: sistema nervoso autônomo (variação circadiana com predomínio matinal), disfunção endotelial, inflamação crônica, estresse oxidativo e hipercontratilidade do músculo liso	- Remodelação estrutural - Espasmo microvascular - Compressão externa (hipertrofia miocárdica) - Condutância microcirculatória reduzida e/ou obstrução arteriolar dinâmica	Trombofilia, fibrilação atrial, doença valvar, forame oval patente, endocardite infecciosa e endocardite trombótica não bacteriana
**Apresentação Clínica**	- Idosa - Dor torácica (precordial/retroesternal); dor na mandíbula, pescoço - Fadiga, náusea - Angina estável/instável IAM	Amplo espectro: desde manifestações clínicas como desconforto torácico até angina e equivalentes anginosos	IAM 2/3 dos casos de IAMSSST (descartar miocardite, Takotsubo, CMPP e EP) Afastar: sepse, EP, causas não cardíacas de elevação da troponina	- Amplo espectro de manifestações clínicas: desde dor torácica leve, SCA, IAM até morte súbita cardíaca - Causa mais comum de IAM na gravidez e no puerpério	Amplo espectro de manifestações clínicas, desde doença silenciosa até morte súbita cardíaca, angina/IAM	Angina Dispneia aos esforços	IAM
**Diagnóstico clínico/ exames complementares**	Semelhante entre mulheres e homens	- Testes não invasivos: RMC associada a adenosina; PET - Testes invasivos: acetilcolina intracoronária e hiperemia com adenosina intracoronária (RFC e IRM)	- Transitório até se estabelecer a causa - Quadro clínico de IAM - Troponina - Angiografia coronariana com ausência de DAC obstrutiva, TCO, USIC RMC	- Ausência de FRCV tradicionais - Conhecimento dos FR predisponentes: displasia fibromuscular (50-86%), distúrbios do tecido conjuntivo (5%), doenças inflamatórias sistêmicas (5-12%), uso de terapia hormonal (estrogênio, progesterona, gonadotrofina, clomifeno ou tratamento de infertilidade) - Múltiplas gestações anteriores - Angiotomografia coronariana - Angiografia coronariana	- Angiografia coronariana associada a teste de provocação farmacológica com acetilcolina	- Associação com marcadores inflamatórios (lúpus eritematoso sistêmico e artrite reumatoide) - Testes não invasivos: defeitos reversíveis - Testes invasivos: FFR > 0.8; RFC < 2.0; IRM ≥ 25	- Angiografia coronariana/trombectomia por aspiração
**Prevenção**	- Conhecimento das diferenças no quadro clínico das SCA/SCC - Aperfeiçoar diagnóstico e tratamento - Identificar e tratar FRCV clássicos e FR específicos da mulher	- Identificar e tratar agressivamente FRCV clássicos e FR específicos da mulher - Identificar os fatores potencializadores de risco (fatores psicossociais e determinantes sociais de saúde)	- Identificar e tratar FRCV clássicos e FR específicos da mulher - Identificar os fatores potencializadores de risco (fatores psicossociais e determinantes sociais de saúde)	- Minimizar os gatilhos emocionais, evitar terapia hormonal (isto é, estrogênio, progesterona e gonadotrofina coriônica β-humana) - Evitar gravidez futura - Reabilitação cardíaca, preferencialmente com protocolo modificado, evitando exercícios isométricos pesados e atividades aeróbicas intensas	- Evitar fatores agravantes: uso de drogas ilícitas, anfetaminas, gás butano, álcool e medicamentos para enxaqueca	- Modificação dos FRCV (perda de peso e controle do estresse)	- Diagnóstico precoce das causas subjacentes e profilaxia da trombose/infecção
**Desfechos**	Mulher: maior mortalidade hospitalar e em 30 dias - Maior mortalidade em mulheres abaixo de 50 anos	- Angina recorrente - Hospitalizações frequentes - Coronariografias repetidas - Altas taxas de eventos CV maiores	- Prognóstico depende da causa subjacente - Mortalidade: semelhante à DAC - Incapacidade: semelhante à DAC	- Recorrência frequente principalmente no período pós-parto e em portadoras de doenças do tecido conjuntivo	- Taxa de recorrência entre 3,9% e 18,6% - Arritmia e morte súbita	- Angina recorrente - Hospitalizações frequentes - Coronariografias repetidas - Altas taxas de eventos CV maiores	- Prognóstico bom na maioria dos casos com trombectomia precoce

CMPP: cardiomiopatia periparto; CV: cardiovascular; DAC: doença arterial coronariana; EP: embolia pulmonar; FFR: reserva de fluxo fracionado; FR: fator de risco; FRCV: fatores de risco cardiovascular; IAM: infarto agudo do miocárdio; IAMSSST: infarto do miocárdio sem supra do segmento ST; IRM: índice de resistência microvascular; PET: PET scan; RFC: reserva de fluxo coronariano; RMC: ressonância magnética cardíaca; SCA: síndrome coronariana aguda; SCC: síndrome coronariana crônica; TCO: tomografia de coronária; USIC: ultrassom intracoronariano.

Atualmente o termo DIC é muito mais adequado para se referir às diversas afecções coronarianas geradoras de isquemia, antes referidas como DAC. A DIC reúne DAC, INOCA, MINOCA, DEAC, DMV, VPC e embolia/trombose coronariana.

#### 4.1.1. Doença Arterial Coronariana

A DAC é a principal causa de infarto na mulher. As diferenças relacionadas a sexo e gênero são especialmente pronunciadas e as peculiaridades estão apresentadas na Tabela 4.1 .

#### 4.1.2. Isquemia na Ausência de Obstrução Arterial Coronariana

A INOCA é mais comum em mulheres do que em homens, com prevalência especialmente alta entre as mulheres com idade de 45-65 anos. Não é uma condição benigna, estando associada a aumento do risco de eventos cardiovasculares adversos. ^69^ As estratégias de abordagem da INOCA não são bem definidas, principalmente porque não há evidências suficientes sobre o tratamento da disfunção microvascular associada a ela. ^70^

#### 4.1.3. Infarto do Miocárdio na Ausência de Obstrução Arterial Coronariana

O MINOCA está relacionado a vários mecanismos fisiopatológicos que acometem coronárias, conforme descrito na Tabela 4.1 . Sua prevalência é de 5-10%, considerando-se todos os IAM, sendo que cerca de dois terços dos pacientes apresentam IAMSSST. ^69^

É mais comum em mulheres mais jovens do que em homens (10,5% vs 3,4%; p<0,0001). Os FRCV podem estar presentes, porém são menos prevalentes do que em pacientes com DAC. ^69^

O diagnóstico de MINOCA é transitório e requer confirmação das várias causas. Primeiramente, deve-se excluir IAM por obstrução de coronária epicárdica. Posteriormente, é de grande importância buscar a causa subjacente, pois a falha na identificação da causa básica pode resultar em tratamento e informações inadequados. ^70^

#### 4.1.4. Dissecção Espontânea de Artéria Coronária

A DEAC é causa rara de infarto do miocárdio, representando 1-4% de todas as SCA. Corresponde à ruptura espontânea da íntima ou dos *vasa vasorum* na parede arterial, resultando no acúmulo de hematoma intramural na falsa luz, que pode comprimir a luz verdadeira, causando isquemia ou infarto do miocárdio.

A DEAC é cada vez mais reconhecida como causa importante de infarto do miocárdio em mulheres com menos de 50 anos. Segundo alguns estudos, 25-35% dos casos de DEAC ocorrem em mulheres antes dos 50 anos e 25% em mulheres com mais de 60 anos. É a causa mais comum de infarto do miocárdio associado a gravidez (até 43% dos infartos do miocárdios na gravidez), ocorrendo principalmente no terceiro trimestre ou no pós-parto. O risco de eventos recorrentes é substancial. ^70^

#### 4.1.5. Doença Microvascular

A sua fisiopatologia resulta do remodelamento estrutural com consequente redução da condutância ou de distúrbios vasomotores afetando as arteríolas, ou de ambos. A confirmação diagnóstica de DMV deve preencher os seguintes critérios: presença de sintomas (angina e/ou dispneia de esforço); ausência de doença obstrutiva; evidência objetiva de isquemia e de alteração da função microvascular (defeitos reversíveis, anormalidades nos testes funcionais invasivos – IRM > 25). ^69^

A DMV associa-se a marcadores pró-inflamatórios em mulheres com INOCA, havendo, portanto, maior risco em doenças como lúpus eritematoso sistêmico e artrite reumatoide. Após a menopausa, essas doenças são mais frequentes nas mulheres, o que pode contribuir para as diferenças sexuais da DMV. ^69^

#### 4.1.6. Vasoespasmo Coronariano

O VPC é definido por vasoconstrição difusa ou focal reversível coronariana, sendo comum entre pacientes com DIC e presente nos mecanismos de INOCA, MINOCA e DMV, independentemente das variações raciais, genéticas e geográficas. É mais prevalente em mulheres de 40-70 anos. ^69^

O teste provocativo com acetilcolina intracoronariana continua a ser a ferramenta de diagnóstico fundamental para o VPC, sendo que as mulheres têm maior resposta a menores doses. Esse teste permite reproduzir o VPC e avaliar a reatividade aos nitratos.

#### 4.1.7. Trombose/Embolia Coronariana

A embolia de artéria coronária é uma causa subdiagnosticada de SCA, sendo dividida em três tipos: direta, paradoxal (trombo oriundo de trombose venosa profunda que transpõe o forame oval) e iatrogênica. Nessa última, a intervenção coronariana percutânea é a causa mais comum de embolia e o risco é aumentado com técnicas de rotação, valvoplastia e anticoagulação inadequada do procedimento.

## 4.2. Insuficiência Cardíaca

**Figura 4.1:**
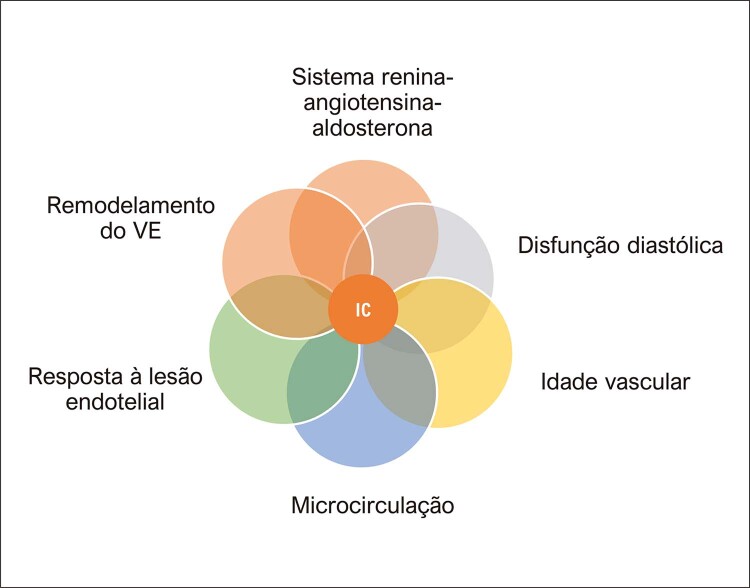
Mecanismos fisiopatológicos relacionados à insuficiência cardíaca na mulher. VE: ventrículo esquerdo; IC: insuficiência cardíaca.

Estudos epidemiológicos revelam incidência de IC similar em homens e mulheres. No entanto, alterações hormonais especialmente após a menopausa são responsáveis por características peculiares da IC em mulheres, o que contribui para a maior prevalência de ICFEp (FE > 50%) em relação a ICFEr (FE < 40%). ^9 , 74 , 75^ A Figura 4.1 mostra os mecanismos fisiopatológicos relacionados à IC na mulher decorrentes do ciclo hormonal. ^10^

**Figura 4.2:**
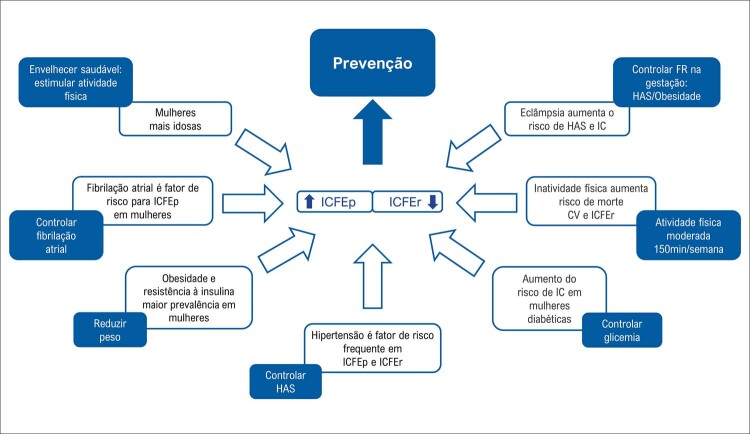
Fatores de risco, características, prognóstico e medidas para prevenir ICFEr e ICFEp em mulheres. CV: cardiovascular; FR: fator de risco; HAS: hipertensão arterial sistêmica; IC: insuficiência cardíaca; ICFEp: insuficiência cardíaca com fração de ejeção preservada; ICFEr: insuficiência cardíaca com fração de ejeção reduzida.

Existem diferenças relacionadas ao sexo não só em relação à epidemiologia da IC, mas também quanto à apresentação clínica, aos desfechos e ao tratamento da IC ( Figura 4.2 ). ^74 , 75^ Essas diferenças são ainda mais evidentes quando os diferentes fenótipos da IC são analisados isoladamente: ICFEr (FE ≤ 40%) e ICFEp (FE ≥ 50%). ^9^

Duas condições clínicas que podem cursar com IC na mulher merecem destaque: Takotsubo, que se refere a disfunção ventricular esquerda aguda reversível, em que 90% dos pacientes são mulheres, especialmente pós-menopausa, ^10^ e a cardiomiopatia periparto, definida como disfunção ventricular esquerda no final da gravidez ou nos primeiros meses pós-parto, sem outra causa evidente. ^76^ A Tabela 4.2 descreve as principais características dessas patologias.

**Tabela 4.2:** Características da síndrome de Takotsubo e cardiomiopatia periparto.

Condição clínica	Síndrome de Takotsubo	Cardiomiopatia Periparto
**Mulheres (%)**	Cerca de 90%	100%
**Média de idade**	Mais idosas – frequentemente após menopausa (90% entre 58 anos e 75 anos)	Idade fértil
**Definição**	Disfunção ventricular esquerda aguda reversível	Disfunção ventricular esquerda no final da gravidez ou primeiros meses após parto
**Fatores de risco**	*Trigger* emocional ou físico é típico, mas não está presente sempre Mais comum em mulheres após a menopausa	✓ Raça afro-americana ✓ Idade materna mais elevada ✓ HAS ou pré-eclâmpsia ✓ Múltiplas gestações
**Características**	✓ Dor torácica aguda sugestiva de SCA ✓ Alterações eletrocardiográficas sugestivas de SCA ✓ Dispneia ✓ Palpitação ✓ Pré-síncope / síncope por arritmia ventricular ou ✓ Choque cardiogênico	✓ Dispneia, ortopneia, edema de membros, palpitação ✓ Sinais de congestão ao final da gravidez ✓ Eventos tromboembólicos ✓ Arritmias ventriculares ✓ Choque cardiogênico
**Diagnóstico**	✓ ECG: elevação ST ou inversão T + QTc alargado ✓ ECO: alteração segmentar que se estende além de território arterial único ✓ Peptídeos natriuréticos elevados ✓ Elevação discreta de troponina (ocorre em mais de 90%) ✓ Disparidade entre o grau de elevação de troponina e a gravidade da disfunção VE ✓ RNM: disfunção VE; avaliação VD; trombo; realce tardio ausente (diferencial com miocardite) ✓ Coronariografia: ausência de lesões coronarianas com hipocinesia apical e médio-apical na ventriculografia	✓ Diagnóstico pode ser atrasado por confusão com sintomas habituais da gestação ✓ Diagnóstico diferencial com miocardite, Takotsubo, cardiomiopatia hipertrófica e outras ✓ ECO: disfunção ventricular esquerda; presença de trombo ✓ Peptídeos natriuréticos elevados ✓ Holter: pode revelar arritmias ventriculares
**Prevenção**	✓ Está relacionada a menopausa (deficiência de estrogênio, porém a reposição hormonal não tem impacto em redução do risco de IC)	✓ Controlar fatores de risco que predispõem à CMPP: hipertensão, diabetes, obesidade, eclâmpsia.
**Tratamento**	✓ Betabloqueador se FEVE < 45%, FA, arritmia ventricular ✓IECA se FEVE < 45% ✓ Anticoagulação se trombo apical ✓ SCM se choque cardiogênico	BOARD ✓ B: Bromocriptina está indicada (doses crescentes de acordo com gravidade da CMPP) ✓ O: Tratamento oral para IC guiado por diretrizes ✓ A: Anticoagulação (pelo menos profilática) ✓ R: Vasodilatadores (relaxantes) EV e inotrópicos para CMPP grave ✓ D: Diuréticos se congestão Para casos mais graves: ✓ BIA, ECMO para casos de rápida deterioração a despeito de inotrópicos ✓ Transplante ou DAV diante da persistência de sintomas a despeito da terapia otimizada
**Prognóstico**	Em geral bom com recuperação precoce da função de VE, porém pode apresentar complicações graves antes da recuperação Choque cardiogênico (6-20%) Mortalidade intra-hospitalar (<5%)	Em geral, bom prognóstico (40% melhoram a FEVE para > 50%) Podem ter disfunção persistente ou rápida deterioração FEVE < 35% na apresentação confere pior prognóstico
**Seguimento**	Exame de imagem para confirmar recuperação da função de VE: ECO ou RNM Recorrência em até 22%	Melhora entre 6 meses e 5 anos em geral Nova gravidez é desaconselhável pelo risco de recorrência

BIA: balão intra-aórtico; CMPP: cardiomiopatia periparto; DAV: dispositivos de assistência ventricular; ECG: eletrocardiograma; ECMO: membrana de oxigenação extracorpórea; ECO: ecocardiograma; EV: endovenoso; FA: fibrilação atrial; FEVE: fração de ejeção ventricular esquerda; HAS: hipertensão arterial sistêmica; IC: insuficiência cardíaca; IECA: inibidor da enzima de conversão da angiotensina; RNM: ressonância nuclear magnética; SCA: síndrome coronariana aguda; SCM: suporte circulatório mecânico; VD: ventrículo direito; VE: ventrículo esquerdo.

Dados de registros de IC aguda demonstram que mulheres e homens são igualmente afetados (cerca de 50%), não havendo evidências de diferença de mortalidade entre homens e mulheres ( Tabela 4.3 ), ^77^ embora mulheres tenham perfil de maior risco (maior idade e maior número de comorbidades). No entanto, em estudos clínicos/registros de choque cardiogênico/suporte circulatório mecânico, as mulheres estão sub-representadas.

**Tabela 4.3:** Diferenças nas características e nos desfechos entre homens e mulheres com insuficiência cardíaca descompensada e/ou choque cardiogênico.

ESTUDO CLÍNICO	ADHERE IC descompensada	IABP-SHOCK II Choque cardiogênico - BIA	cVAD Registry Choque cardiogênico - IMPELLA
**Mulheres (%)**	52%	31%	27%
**Ano**	2001-2004	2009-2012	2007-2013
**Características**	Mais idosas (75 x 70 anos)	Mais idosas (74 x 68 anos)	Mais idosas (71 x 64 anos)
	Maior % comorbidades: HAS Doença tireoidiana Menor % etiologia isquêmica (19% vs 32%)	Maior % comorbidades: HAS (76% vs 66%) DM (40% vs 29%) Menor % etiologia isquêmica (16% vs 25%)	Maior % comorbidades: HAS (80% vs 70%) DM (54% vs 41%) Similar % etiologia isquêmica (33% vs 35%)
	Maior FEVE (42% vs 33%)	Maior FEVE (40% vs 35%)	Maior FEVE (30% vs 24%)
**Mortalidade**	Sem diferença mortalidade ajustada intra-hospitalar	Sem diferença mortalidade ajustada em 30 dias, 6 meses e 1 ano	Sem diferença mortalidade na alta hospitalar

BIA: balão intra-aórtico; DM: diabetes mellitus; FEVE: fração de ejeção ventricular esquerda; HAS: hipertensão arterial sistêmica; IC: insuficiência cardíaca.

A melhor forma de prevenir a progressão para IC avançada é a adequada instituição do tratamento da IC. Porém, quando não suficiente, o tratamento padrão-ouro é o TC para ambos os sexos. A sobrevida média em longo prazo é significativamente maior no sexo feminino e a presença de *mismatch* de sexo (doador feminino/receptor masculino) tem pior prognóstico. ^70^ Quanto ao uso de dispositivos de assistência ventricular de longa permanência, os resultados têm sido cada vez melhores. Entre as suas indicações estão pacientes com contraindicação para o TC, como hipersensibilização, o que é frequente em multíparas. Mulheres tendem a maior risco de AVC e disfunção de ventrículo direito e são sub-representadas na maioria dos registros. ^77^

### 4.2.1. Tratamento Farmacológico e Não Farmacológico da ICFEr e ICFEp

Considerando que a cardiomiopatia isquêmica é a etiologia mais frequente da ICFEr em mulheres, instituir medidas que previnam DAC, como controle de dislipidemia e hipertensão, cessação de tabagismo e estímulo à atividade física, é fundamental ( Figura 4.2 ). O tratamento farmacológico da ICFEr com inibidores da enzima de conversão de angiotensina II, betabloqueadores, antagonistas mineralocorticoides, INRA e inibidores de SGLT2 tem demonstrado impacto inequívoco em redução de morte geral ou mortalidade cardiovascular e hospitalização por IC. ^9^ Tais benefícios ocorreram de forma semelhante em homens e mulheres e, portanto, a instituição do tratamento farmacológico previne morte súbita e progressão da doença para IC avançada que requer terapias específicas. No entanto, as mulheres estão sub-representadas na grande maioria dos estudos de ICFEr (20-30%) e, portanto, estudos envolvendo maior número de mulheres com ICFEr seriam muito relevantes ( Tabela 4.4 ). ^10^

**Tabela 4.4:** Diferenças nos desfechos entre homens e mulheres em estudos de ICFEr.

ESTUDO CLÍNICO	% Mulheres	DESFECHO	RESULTADO GERAL	HOMENS / MULHERES	P interação
**SOLVD (Enalapril)**	20%	Morte geral	HR 0,84 (0,74 – 0,95)	-----	-----
**CIBIS II (Bisoprolol)**	20%	Morte geral	HR 0,66 (0,54 – 0,81)	H: HR 0,53 (0,42 – 0,67) M: HR 0,37 (0,19 – 0,89)	NS
**RALES (Espironolactona)**	27%	Morte geral	HR 0,69 (0,58 – 0,82)	H: HR 0,71 (0,60 – 0,82) M: HR 0,72 (0,57 – 0,97)	NS
**PARADIGM-HF (Sacubitril-Valsartana)**	21%	Morte CV ou hospitalização IC	HR 0,80 (0,73 – 0,87)	H: HR 0,80 (0,73 – 0,87) M: HR 0,79 (0,66 – 0,94)	NS
**DAPA-HF (Dapagliflozina)**	23%	Morte CV ou hospitalização IC	HR 0,75 (0,65-0,85)	H: HR 0,73 (0,63 – 0,85) M: HR 0,79 (0,59 – 1,06)	NS
**EMPEROR-Reduced (Empagliflozina)**	24%	Morte CV ou hospitalização IC	HR 0,75 (0,65– 0,86)	H: HR 0,80 (0,68 – 0,93) M: HR 0,59 (0,44 – 0,80)	NS

CV: cardiovascular; H: homens; HR: hazard ratio; IC: insuficiência cardíaca; M: mulheres; NS: não significativo.

Na ICFEp, a fisiopatologia está diretamente ligada a obesidade, resistência a insulina aumentada e síndrome metabólica. Assim, medidas como redução de peso e estímulo à atividade física são essenciais para a prevenção de ICFEp, especialmente em mulheres ( Figura 4.2 ). Quanto ao tratamento farmacológico, as evidências não são tão robustas e, até recentemente, não havia terapia medicamentosa recomendada. ^9^ Estudos randomizados revelam efeito neutro dos principais fármacos para IC. ^77^ Subanálises de dois deles, um envolvendo espironolactona (TOPCAT) e outro INRA (PARAGON-HF), sugerem resposta diferente à terapêutica de acordo com o sexo. Além disso, os inibidores SGLT2 demonstraram benefícios na ICFEp com resultado similar em homens e mulheres ( Tabela 4.5 ). ^10^ Dessa forma, as evidências atuais apontam para benefício de inibidores SGLT2 em ambos os sexos e potencial benefício com espironolactona e sacubitril-valsartana em mulheres. ^76^ Estudos prospectivos para avaliar diferenças entre os sexos também são necessários.

**Tabela 4.5:** Diferenças nos desfechos entre homens e mulheres em estudos de ICFEp.

ESTUDO CLÍNICO	% Mulheres	DESFECHO	RESULTADO GERAL	HOMENS / MULHERES	DIFERENÇAS entre sexos	P interação
TOPCAT (Espironolactona)	52%	Morte CV ou hospitalização por IC ou PCR	HR 0,89 (0,77 – 1,04)	Morte geral H: p=0,68 M: HR 0,66 (0,48 – 0,90)	Redução significativa de morte geral apenas em mulheres	0,02
PARAGON-HF (Sacubitril-Valsartana)	52%	Morte CV ou hospitalização por IC	HR 0,87 (0,75 – 1,01)	H: HR 1,03 (0,85 – 1,25) M: HR 0,73 (0,59 – 0,90)	Redução significativa morte CV ou hospitalização por IC e hospitalizações totais apenas em mulheres	0,017
EMPEROR-Preserved (Empagliflozina)	45%	Morte CV ou hospitalização por IC	HR 0,79 (0,69 – 0,90)	H: HR 0,81 (0,69 – 0,96) M: HR 0,75 (0,61 – 0,92)	Redução significativa de morte CV ou hospitalização por IC em homens e mulheres	NS
DELIVER (Dapagliflozina)	44%	Morte CV ou hospitalização por IC ou visita à emergência	Em andamento			

CV: cardiovascular; H: homens; HR: hazard ratio; IC: insuficiência cardíaca; M: mulheres; NS: não significativo; PCR: parada cardiorrespiratória.

De maneira geral, tanto na IC crônica quanto na IC aguda, ensaios clínicos voltados especialmente para mulheres são escassos e as evidências são provenientes de subanálise de grandes estudos em que as mulheres estão sub-representadas. No entanto, a identificação de potenciais fatores de risco para o desenvolvimento de IC em mulheres e a instituição de medidas para controlá-los, ou até revertê-los, podem ter impacto prognóstico. Registros prospectivos multicêntricos poderiam trazer evidências mais precisas na população feminina.

## 4.3. Arritmias

Existem algumas diferenças eletrofisiológicas entre os sexos em relação à ocorrência, aos sintomas clínicos e ao prognóstico das arritmias, podendo afetar tanto a despolarização quanto a repolarização. As mulheres têm mais taquicardia sinusal e taquicardia por reentrada nodal, enquanto arritmias ventriculares são menos comuns. Na síndrome do QT longo tipo 2, é conhecido um maior risco de morte súbita entre as mulheres e um maior risco de pró-arritmia, seja por drogas cardiovasculares seja por drogas não cardiovasculares. A gravidez aumenta o risco de taquicardias supraventriculares e diminui a ocorrência de *torsades de pointes,* que tem seu risco aumentado na síndrome de QT longo durante o puerpério. ^70^

### 4.3.1. Taquicardia Ventricular e Morte Súbita Cardíaca

A MSC é um importante problema de saúde pública, com ampla incidência global. Nos Estados Unidos, em 2016, ocorreram 366.494 casos, sendo 178.823 (48,8%) em mulheres. A DIC é a causa mais comum de MSC; no entanto, nas mulheres, a MSC ocorre mais por causas não isquêmicas. As mulheres têm menos taquicardia/fibrilação ventricular documentada (19,4% mulheres *vs* homens 26,7%, p<0,001), o que reduz a probabilidade de sobrevivência se comparadas aos homens, que são reanimados e tratados com desfibrilador. ^70^

O CDI é a terapia de escolha na profilaxia primária ou secundária de MSC. No entanto, as mulheres são sub-representadas nos estudos clínicos (inclusão de mulheres varia entre 16% e 29%) e as análises de subgrupos baseadas no sexo são limitadas. ^78^ Um registro de 236.084 beneficiários do Medicare de 1991 a 2015 evidenciou que homens receberam mais CDI que mulheres, em prevenção tanto primária (3,2 vezes mais) quanto secundária (2,4 vezes mais). Ademais, mulheres têm maior risco de complicações relacionadas ao procedimento de implante do CDI (7,2% *vs* 4,8%, p<0,001), particularmente pneumotórax com necessidade de intervenção, tamponamento cardíaco e complicações mecânicas do dispositivo que requeiram revisão. ^13^

### 4.3.2. Fibrilação Atrial

**Figura 4.3:**
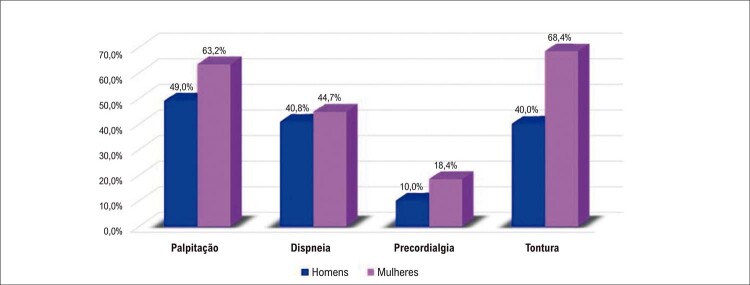
Diferença de sintomas entre mulheres e homens com fibrilação atrial. 72

Estima-se que 29,4 milhões de mulheres tenham FA em todo o mundo. Embora a incidência seja maior entre os homens, as mais idosas têm mais FA, visto que a expectativa de vida é maior nas mulheres. ^1^ As mulheres com FA são mais sintomáticas e relatam pior qualidade de vida em relação aos homens ( Figura 4.3 ), ^79^ além de apresentarem preditores peculiares para FA. Um estudo com 34.221 mulheres observou que HAS foi um preditor de risco, sendo a obesidade outro marcador de risco importante e as mudanças dinâmicas no peso também deletérias. Mulheres que praticam atividade física vigorosa têm redução de incidência de FA em 28%. Entre as saudáveis de meia-idade, foi demonstrada uma incidência aumentada de FA nas que consumiam dois ou mais drinques por dia, sendo o consumo excessivo de álcool um preditor de FA entre elas. Um estudo prospectivo de larga escala demonstrou que a multiparidade associou-se a maior risco. Outro estudo prospectivo avaliou 30.034 mulheres na menopausa, demonstrando que não houve aumento do risco além daquele associado à idade, enquanto a monoterapia com estrogênio associou-se a aumento do risco da arritmia. ^80^

As mulheres têm mais FA paroxística, maior pontuação de CHA _2_ DS _2_ -VASc, são menos submetidas a cardioversão e a ablação de *flutter* e FA, são mais submetidas a ablação do nó atrioventricular e implante de marca-passo, têm mais AVC e tromboembolismo e são mais hospitalizadas por todas as causas. ^14^

Poucos estudos sobre o controle de frequência cardíaca e de ritmo foram avaliados em relação a diferenças entre os sexos. As mulheres são menos submetidas a ablação por cateter com isolamento das veias pulmonares e, em geral, os resultados são piores, seja por indicação mais tardia, por mais comorbidades ou por maior presença de fibrose atrial e focos extra veias pulmonares. ^15^ Um estudo com 2.789 pacientes com FA de início recente com grande representatividade de mulheres (46%) mostrou que o controle precoce do ritmo foi associado a menor risco de eventos adversos cardiovasculares, sem afetar o tempo de internação. ^81^

As mulheres com FA têm maior risco de AVC, sendo esse mais severo e ainda mais extenso quando em idade acima de 65 anos. ^15 , 70^ Os estudos com DOACs (dabigatrana, apixabana, rivaroxabana e edoxabana) demonstraram menor risco de sangramento maior em mulheres, porém maior risco de AVC/embolia sistêmica com o uso de varfarina, apesar de esses estudos avaliarem apenas 35% a 40% de mulheres. ^15^ Houve redução de mortalidade por todas as causas e redução significativa do risco de hemorragia intracraniana com uso de DOACs em mulheres, comparados à varfarina. Em estudos que avaliaram a oclusão do apêndice atrial esquerdo como opção à anticoagulação na prevenção do AVC em FA, as mulheres foram sub-representadas e o desfecho primário de eficácia composto por AVC, embolia sistêmica e morte cardiovascular não variou entre os sexos. ^15 , 70^ As mulheres com FA têm maior risco de mortalidade cardiovascular e por todas as causas, além de mais eventos cardíacos, maior risco de AVC e IC. ^15^ As principais diferenças de sintomas entre os sexos na FA são sumarizadas na Quadro 4.1 .

**Quadro 4.1:** Resumo das diferenças entre os sexos nos portadores de fibrilação atrial. 15

MULHER		HOMEM
**EPIDEMIOLOGIA E RISCO**		
Maior que na população total Maior risco de óbito e AVC pela FA	Aumento da incidência e prevalência com o tempo	Prevalência ajustada pela idade
**SINTOMAS E QUALIDADE DE VIDA**		
Maior duração dos sintomas Pior qualidade de vida nos escores		
**PREVENÇÃO E RISCO DE AVC**		
AVC da FA mais severo e incapacitante		Recebe mais anticoagulação para prevenção Maior tempo na faixa terapêutica com varfarina
**CONTROLE DE FREQUÊNCIA E RITMO CARDÍACO**		
Maior risco de eventos cardiovasculares epró-arritmia com DAA Maior risco de eventos adversos com ablação por cateter		Maior indicação de cardioversão e ablação de FA

AVC: acidente vascular cerebral; DAA: drogas antiarrítmicas; FA: fibrilação atrial.

## 4.4. Doença Cardiovascular e Câncer

As DCV e o câncer são as principais causas de morte no mundo, compartilhando FR, tais como idade, obesidade, tabagismo, história familiar e dieta.

Os cânceres mais comuns em mulheres são os de mama, pulmão e colorretal, responsáveis por 50% dos novos diagnósticos, dos quais o da mama representa 30%. ^82^

Avanços no diagnóstico precoce e no tratamento do câncer propiciaram importante redução na mortalidade, principalmente pela percepção de efeitos adversos cardiovasculares, tanto agudos quanto crônicos, decorrentes do tratamento oncológico. Esses efeitos afetam a qualidade e a expectativa de vida das sobreviventes, que necessitam de acompanhamento por toda a sua existência.

Entre as diferentes formas de tratamento, tanto a quimioterapia quanto a radioterapia e outras terapias podem levar a cardiotoxicidade por diferentes mecanismos, sendo o risco diretamente relacionado à detecção de doença cardíaca de base, de fatores de risco cardiovascular e tratamento prévio oncológico cardiotóxico.

Apesar de até o momento não existir nenhum escore de risco validado, sugerem-se dois fortes preditores de risco, a idade e a FEVE previamente ao tratamento. ^83^

O diagnóstico da cardiotoxicidade pode ser realizado pela confirmação de alteração cardiovascular nova durante ou mesmo anos após o tratamento, seja de natureza clínica e/ou alteração em biomarcadores e/ou imagem cardiovascular, sendo o diagnóstico feito por exclusão após pesquisa de outras etiologias.

Segundo a Diretriz Brasileira de Cardio-Oncologia, disfunção ventricular relacionada à terapia do câncer é definida como uma redução ≥ 10% na FEVE para um valor abaixo do limite inferior da normalidade (FEVE < 50%), devendo ser repetida a imagem cardiovascular em 2 a 3 semanas. ^84^

A disfunção ventricular pode ocorrer durante a evolução do tratamento bem como anos após o seu fim, motivo pelo qual a vigilância a longo prazo é importante, principalmente naqueles que desenvolvem cardiotoxicidade durante o tratamento ou são de alto risco.

O ecocardiograma é o método de escolha para detecção de disfunção miocárdica antes, durante e após o tratamento do câncer. O ecocardiograma tridimensional tem uma melhor acurácia na avaliação da função ventricular. Em sua ausência, o bidimensional Simpson é recomendado para avaliação dos volumes e da FEVE. O SLG é uma ferramenta que prediz com alta sensibilidade a redução posterior da FEVE. Redução ≥ 15% no SLG em relação ao basal é considerada anormal, sendo um marcador precoce de disfunção ventricular. A ressonância nuclear magnética é o padrão-ouro para avaliação da função cardíaca, sendo indicada em casos com limitação da ecocardiografia. ^84^

É fundamental detectar anormalidades cardíacas subclínicas que possam influenciar as decisões clínicas quanto à escolha do tratamento, indicação de cardioproteção ou aumento da vigilância (por exemplo, disfunção ventricular assintomática).

A antraciclina é o QT mais comumente utilizado no tratamento do câncer de mama, porém tem efeito cardiotóxico por necrose de cardiomiócitos histologicamente comprovada, levando a lesões irreversíveis. A associação entre doses cumulativas de antraciclina e risco de IC é exponencial, com incidência de 5% de IC com a dose cumulativa de 400 mg/m ^2^ e incidência de 48% com a dose cumulativa de 700 mg/m ^2^ . ^85^

Outro QT usado no tratamento do câncer de mama, o trastuzumabe, pode causar cardiotoxicidade, porém não se associa a necrose de cardiomiócitos, sendo as lesões causadas parcial e totalmente reversíveis após a interrupção do tratamento.

Fluoropirimidina, droga usada no tratamento do câncer colorretal, pode levar a vasoespasmo e consequente isquemia miocárdica, com ou sem alterações eletrocardiográficas. Os sintomas podem aparecer a qualquer momento durante o tratamento, porém injeção em *bolus* pode ser menos cardiotóxica, uma vez que o vasoespasmo pode estar relacionado a metabólitos acumulados mais do que ao efeito do pico das doses. ^86^

Caso os pacientes desenvolvam IC e FEVE < 40% durante o tratamento, o QT deve ser suspenso temporariamente, conforme discussão entre o cardiologista e o oncologista, e a terapia para IC deve ser iniciada de acordo com as diretrizes e consensos.

**Figura 4.4:**
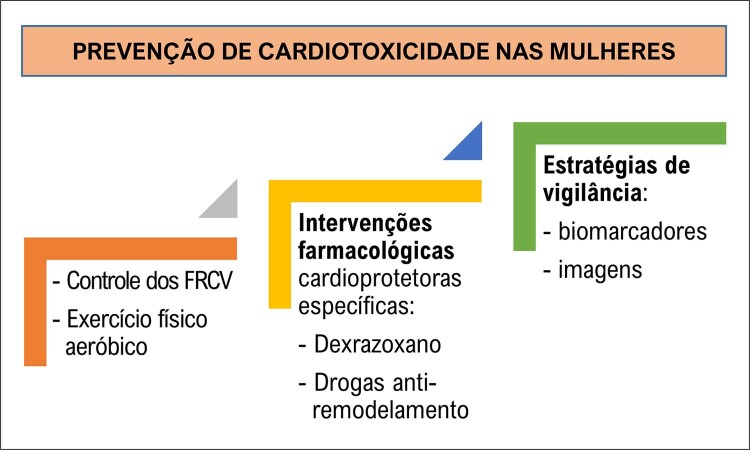
Medidas para a prevenção de cardiotoxicidade nas mulheres.

A prevenção da cardiotoxicidade deve ser realizada em todas as pacientes que serão submetidas ao tratamento do câncer. Medidas gerais, como o controle adequado dos fatores de risco, intervenções farmacológicas cardioprotetoras específicas, assim como estratégias de vigilância baseadas em imagens e biomarcadores têm efeito benéfico geral, mas os resultados são heterogêneos, não havendo consenso sobre as recomendações para farmacoterapia cardioprotetora ( Figura 4.4 ). ^85^

FRCV: fatores de risco cardiovascular

Apesar de as recomendações serem controversas, drogas cardioprotetoras têm sido testadas, como dexrazoxano, um quelante de ferro com comprovada ação cardioprotetora, e outras drogas com efeito bloqueador da resposta neuro-hormonal e consequente ação anti-remodelamento cardíaco, como os inibidores do sistema renina-angiotensina-aldosterona, betabloqueadores e estatinas. ^86^ Considerar o uso do dexrazoxano em pacientes com câncer de mama metastático com dose cumulativa elevada de antraciclina (doxorrubicina acima de 250 mg/m ^2^ ). ^86^

O exercício aeróbico é considerado como estratégia não farmacológica promissora para prevenir e/ou tratar cardiotoxicidade induzida por quimioterapia. Se houver redução da FEVE que atenda à definição de cardiotoxicidade, o tratamento da IC baseado em diretrizes deve ser considerado.

**Figura 4.5:**
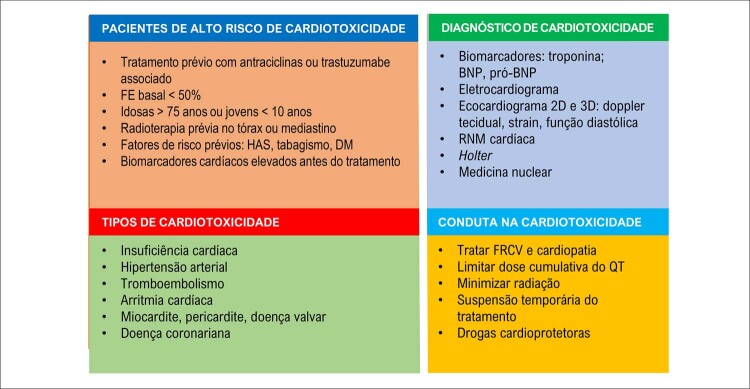
Sumário dos principais aspectos referentes à cardiotoxicidade. 2D: bidimensional; 3D: tridimensional; DM: diabetes mellitus; FE: fração de ejeção; FRCV: fatores de risco cardiovasculares; HAS: hipertensão arterial sistêmica; QT: quimioterápico; RNM: ressonância nuclear magnética.

A Figura 4.5 resume os principais aspectos relacionados à cardiotoxicidade.

Os Quadros 4.2 , 4.3 e 4.4 demonstram as recomendações para o manejo da doença isquêmica coronariana, insuficiência cardíaca e arritmia nas mulheres.

**Quadro 4.2:** Recomendações para o manejo da doença isquêmica do coração nas mulheres.

Recomendações para o manejo da doença isquêmica do coração nas mulheres			

Recomendação	CR	NE	Referências
Na doença coronariana obstrutiva e não obstrutiva, devem-se conhecer as diferenças no quadro clínico das SCA/SCC, aperfeiçoar diagnóstico e tratamento e identificar e tratar FRCV clássicos e FR específicos da mulher		**B**	^69–73^
Na doença coronariana obstrutiva e não obstrutiva, devem-se identificar os fatores potencializadores de risco (fatores psicossociais e determinantes sociais de saúde)		**B**	^69–73^
Na dissecção espontânea, minimizar os gatilhos emocionais, evitar terapia hormonal (isto é, estrogênio, progesterona e gonadotrofina coriônica β-humana), evitar gravidezes futuras, reabilitação cardíaca, preferencialmente com protocolo modificado evitando exercícios isométricos pesados e atividades aeróbicas intensas		**B**	^69–71,73^
No vasoespasmo, evitar fatores agravantes, como o uso de drogas ilícitas, anfetaminas, gás butano, álcool e medicamentos para enxaqueca		**B**	^69,71–73^
Na doença microvascular, importante a modificação dos FRCV (por exemplo, perda de peso e controle do estresse)		**B**	^69,72^
Na trombose e embolia coronariana, diagnóstico precoce das causas subjacentes e profilaxia da trombose/infecção		**B**	^69,73^

CR: classe de recomendação; NE: nível de evidência; FR: fatores de risco; FRCV: fatores de risco cardiovascular; SCA/SCC: síndrome coronariana aguda/síndrome coronariana crônica.

**Quadro 4.3:** Recomendações para o manejo da insuficiência cardíaca nas mulheres.

Recomendações para o manejo da insuficiência cardíaca nas mulheres			

Recomendação	CR	NE	Referências
A síndrome de Takotsubo está relacionada a menopausa (deficiência de estrogênio), porém a reposição hormonal não tem impacto na redução do risco de IC		**C**	^10,76^
Controlar fatores de risco que predispõem à CMPP: hipertensão, diabetes, obesidade, eclâmpsia		**B**	^10,76^
Na ICFEp e na ICFEr, controlar os fatores de risco, como peso e níveis glicêmicos, lipídicos e pressóricos, praticar atividade física regular, cessar tabagismo		**B**	^10,74–76^
Uso de estatina teria potencial benefício, porém existem poucas evidências específicas relacionadas ao sexo, não devendo ser recomendado	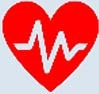	**C**	^10^
A identificação de potenciais fatores de risco para o desenvolvimento de IC em mulheres e a instituição de medidas para controlá-los ou até revertê-los podem ter impacto na prevenção		**B**	^10,70,76^

CR: classe de recomendação; NE: nível de evidência; CMPP: cardiomiopatia periparto; IC: insuficiência cardíaca; ICFEp: insuficiência cardíaca com fração de ejeção preservada; ICFEr: insuficiência cardíaca com fração de ejeção reduzida.

**Quadro 4.4:** Recomendações para o manejo das arritmias nas mulheres.

Recomendações para o manejo das arritmias nas mulheres			

Recomendação	CR	NE	Referências
A obesidade e a HAS são preditores de risco para FA nas mulheres; o controle de peso e níveis pressóricos é medida importante na prevenção		**B**	^14,15,81^
A prevalência de FA aumenta com a idade e nas mulheres após a menopausa; a terapia hormonal da menopausa não teria efeito benéfico	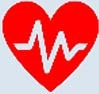	**C**	^80^
A atividade física vigorosa parece reduzir a incidência de FA nas mulheres		**B**	^80^
O consumo excessivo de álcool é preditor de FA nas mulheres; o controle do consumo de álcool para menos de 2 drinques/dia pode reduzir o risco		**B**	^80^

CR: classe de recomendação; NE: nível de evidência; FA: fibrilação atrial; HAS: hipertensão arterial sistêmica.

## 4.5. Acidente Vascular Cerebral

As mulheres enfrentam uma carga desproporcional de mortalidade e incapacidade por AVC. ^87^ Múltiplos FR específicos têm sido observados, como gravidez, PE, DG, uso de contraceptivos orais e de hormônios na pós-menopausa, além das variações hormonais. ^88^ Os FR para AVC mais fortes ou prevalentes nas mulheres que merecem consideração são: FA, enxaqueca com aura, DM, HAS, depressão e estresse psicossocial. Apesar da maior proporção de AVC ao longo da vida, não há diretrizes específicas de triagem e de tratamento para redução do risco de AVC nas mulheres. ^68^

É importante reforçar a conscientização e a educação sistemática nas faixas etárias mais jovens, incluindo mulheres na idade reprodutiva, alertando sobre a progressiva incidência de AVC com a idade e sua associação com complicações obstétricas, como HG, parto prematuro e DG, além de contracepção hormonal. O risco é progressivamente maior quando associado a FR clássicos, como obesidade, dislipidemias, HAS e DM, que incidem ao longo da vida, inclusive em idades mais jovens. ^87^

Embora as mulheres representem mais da metade da ocorrência de AVC na população geral, sua inclusão em ensaios clínicos de tratamento para AVC tem sido menor, o que requer uma melhor representação feminina nesses estudos. ^89 , 90^

## 4.6. Doença Arterial Periférica

**Figura 4.6:**
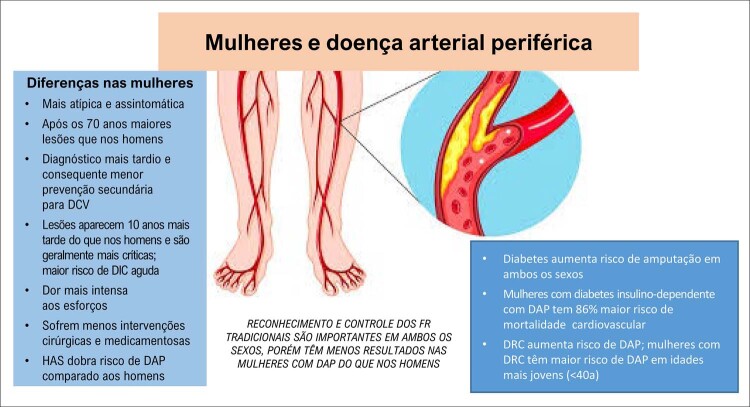
Diferenças na apresentação da doença arterial periférica em mulheres. 7 DAP: doença arterial periférica; DCV: doença cardiovascular; DIC: doença isquêmica do coração; DRC: doença renal crônica; FR: fatores de risco; HAS: hipertensão arterial sistêmica.

A DAP é uma condição prevalente que confere morbidade e mortalidade substanciais, sendo pouco diagnosticada e pouco tratada na população geral. ^91^ Sua frequência em mulheres é igual ou maior do que em homens. ^92^ Diferenças na fisiopatologia e FR podem contribuir para a apresentação tardia e muitas vezes atípica da DAP em mulheres ( Figura 4.6 ).

A inadvertência no atendimento da DAP prejudica os resultados em todos os pacientes, mas esses desafios são maiores nas mulheres, que têm dor mais intensa, pior qualidade de vida e maior risco de DCV e seus eventos concomitantes do que os homens. ^93^ Além disso, há dados demonstrando o menor uso da terapêutica baseada em evidências em mulheres em comparação a homens. ^97^

Embora o reconhecimento e o controle dos FR tradicionais sejam importantes para ambos os sexos, parecem ter um efeito diferencial e menor significância geral para mulheres com DAP do que para homens. ^98 , 99^ Como o tratamento dos FR tradicionais confere proteção para ambos os sexos, esforços devem ser feitos para assegurar os cuidados ideais para todos. ^100^ O reconhecimento de FR específicos do sexo (ex. gravidez) ou de fatores predominantemente femininos (ex. depressão) pode permitir uma adequada estratificação de risco e a adoção de medidas de prevenção precoces. ^101 , 102^

## 4.7. Demência

Apesar de não se saber claramente quais os impactos da privação estrogênica da menopausa sobre a função cognitiva da mulher, dados da literatura mostram que as mulheres parecem ter maior risco de desenvolver demência e alterações de memória relacionadas à idade do que os homens e pouco se sabe sobre essas diferenças em idades mais precoces. ^16^ Estudos mostram que mulheres na perimenopausa respondem melhor a tarefas relacionadas à memória, o que se atenua anos após a menopausa. ^16 , 103^

Há evidências epidemiológicas e biológicas de que a concentração sérica de estrogênio e, consequentemente, a atividade ovariana têm relação com a melhor *performance* em relação à memória e à cognição. ^16 , 103^ Sintomas como mente embotada, esquecimento e dificuldade de achar palavras são comuns nas mulheres na perimenopausa. ^16^ Além disso, como observado no estudo SWAN, a redução da função cognitiva nas mulheres parece não ter relação com ansiedade e depressão, fatores sabidamente aumentados nessa fase da vida feminina. ^65^

A DA, uma das causas de demência na população geral, tem como uma de suas principais características patológicas o depósito excessivo de placas de amiloide no sistema nervoso central. Dados de literatura sugerem que o estrogênio possa ter importante papel na prevenção do depósito de amiloide, além de atuar na função cognitiva. ^104^ Investigadores têm sugerido que a concentração reduzida de hormônios esteroides sexuais após a menopausa possa ser responsável pela maior prevalência e maior gravidade da DA em mulheres. ^105^ Muitos estudos têm sido realizados para definir se a THM seria uma medida eficaz na prevenção desses distúrbios neurológicos. ^105 , 106^

Apesar das evidências epidemiológicas conflitantes em relação ao uso de THM como medida preventiva na disfunção cognitiva e na redução da DA na menopausa, uma meta-análise de ensaios clínicos mostrou a ausência desses benefícios. Mulheres com mais de 65 anos sem demência que usaram estrogênio isolado ou associado a progesterona não tiveram evidências consistentes em relação a benefícios, nem mesmo as mais jovens (menopausa precoce e perimenopausa). ^106^

Assim, sugerimos que a THM não deva ser prescrita para preservação da função cognitiva em mulheres mais velhas na pós-menopausa. Também faltam fortes evidências de benefícios cognitivos para mulheres que usam THM em idades mais jovens (por exemplo, perto da menopausa). Portanto, a THM também não deve ser prescrita para preservação da função cognitiva em mulheres mais jovens. Mesmo resultados do estudo WHI não mostraram que o início precoce da THM seria necessário para obter benefícios cognitivos posteriores nas mulheres, nem mesmo prevenir DIC (“hipótese da janela crítica”). ^107^

Um estudo de coorte mostrou as principais causas de alterações cognitivas e demência em homens e mulheres mais jovens (menos de 55 anos): DA (34%), demência vascular (18%), demência frontoparietal (12%), demência relacionada a alcoolismo (10%), além de outras, como doença de Parkinson e esclerose múltipla. ^108^ Na DA, 10% dos casos se relacionaram a mutação genética e a prevalência de demência dobrou a cada 5 anos após os 35 anos de idade.

Existem FR vasculares relacionados a demência precoce como AVC, ataque isquêmico transitório, doença renal, DCV, HAS, alcoolismo crônico e intoxicação por drogas. ^109^

## 4.8. Doenças Valvares

A doença reumática é a principal causa de doença valvar adquirida em crianças e adultos jovens com idade inferior a 40 anos nos países emergentes. ^110^

### 4.8.1. Estenose Aórtica

Em mulheres jovens, a etiologia da estenose aórtica isolada, na sua totalidade, é congênita e a lesão estrutural é frequentemente a valva bicúspide. ^17^ A estenose aórtica isolada de etiologia reumática é rara, geralmente concomitante a outras lesões valvares, como insuficiência aórtica ou lesões mitrais. Com o envelhecimento, a calcificação degenerativa valvar é o principal mecanismo da doença. Quando comparadas aos homens, considerando o mesmo grau de calcificação valvar, as mulheres apresentam tendência à maior gravidade da estenose aórtica em razão da fibrose do aparelho valvar, que é mais pronunciada do que a calcificação.

**Figura 4.7:**
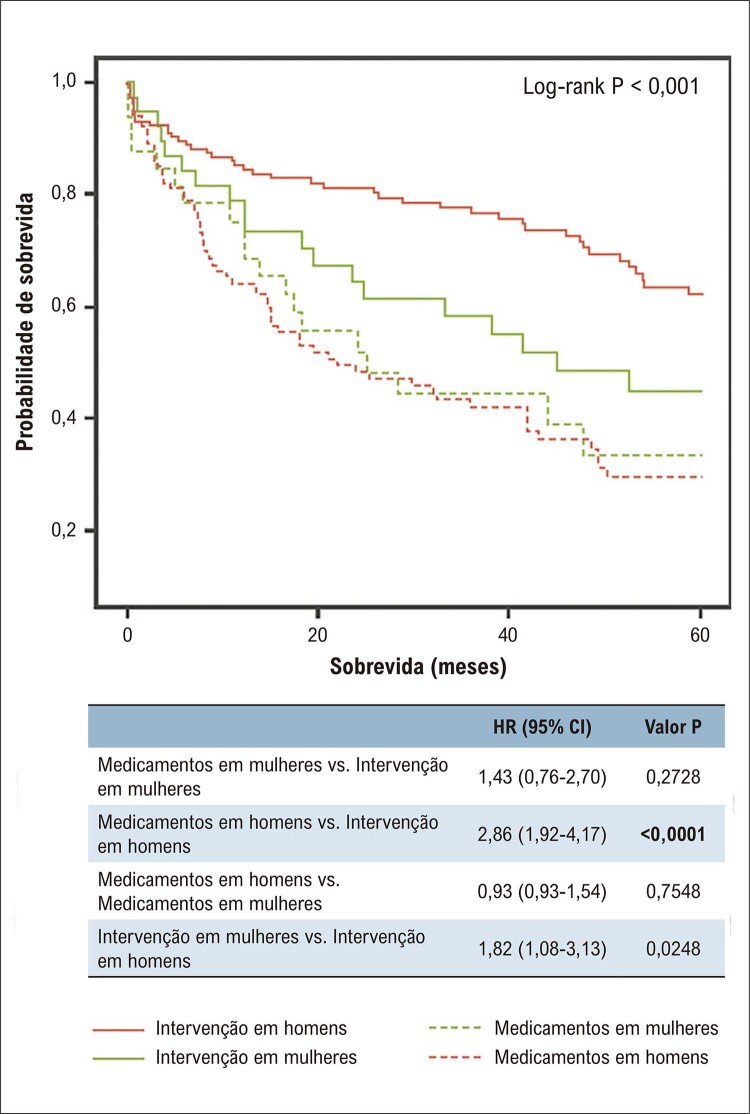
Diferenças na evolução clínica relacionadas ao sexo na estenose aórtica de baixo gradiente e baixa fração de ejeção. Adaptado de Bartko et al. 110

Mulheres portadoras de estenose aórtica de baixo gradiente e baixa fração de ejeção não valorizam os sintomas e apresentam-se em estágios mais avançados da doença, com pior capacidade funcional, quadro de síncope mais frequente e remodelamento mais excêntrico do ventrículo esquerdo. Essas manifestações podem justificar sua maior mortalidade em comparação aos homens ( Figura 4.7 ). ^17^

Mulheres encaminhadas para substituição cirúrgica valvar aórtica apresentam dispneia de esforço mais acentuada, escores de fragilidade mais elevados e grau de obstrução valvar mais grave para o mesmo padrão anatômico de área valvar e gradiente médio de pressão transvalvar aórtico dos homens. ^18^ A menor superfície corpórea das mulheres acarreta intervenções cirúrgicas tecnicamente mais minuciosas, razão da pior sobrevida pós-operatória.

Nas substituições valvares aórticas transcateter, mulheres são mais idosas, apresentam melhor função ventricular esquerda e menor prevalência de DAC, mas têm morbidades associadas como DM e FA. Características anatômicas do sexo feminino, tais como menor distância entre os óstios coronarianos e o anel valvar e maior prevalência de calcificação valvar e da aorta, são responsáveis pela maior incidência de obstrução coronária durante o procedimento. O menor diâmetro dos vasos periféricos também causa maiores complicações vasculares e sangramento. ^111^

Atualmente não há terapêutica para prevenção da progressão da estenose aórtica. ^8^ Contudo, ensaios clínicos têm formulado estratégias para retardar o processo de progressão da doença, como os inibidores da DPP-4, ^18^ testados em idosos e diabéticos, mas sem estudos referenciados para mulheres. A falta de controle de tabagismo, dislipidemia, níveis séricos de creatinina e cálcio plasmático parece propiciar uma redução absoluta e porcentual da área valvar aórtica anual. ^111 , 112^

### 4.8.2. Doença Valvar Mitral

A insuficiência mitral tem história natural dependente da etiologia e o acompanhamento clínico exige medidas preventivas fundamentadas na estratificação do grau anatômico da doença. A avaliação periódica exige atenção quanto ao desenvolvimento ou não de alterações anatômicas e/ou funcionais secundárias à doença valvar e ao surgimento de fatores complicadores, uma vez que a insuficiência mitral tem evolução muitas vezes insidiosa. A insuficiência mitral decorrente do prolapso valvar é prevalente entre as mulheres e suas características anatômicas peculiares mostram predomínio de válvulas mixomatosas com prolapso anterior e de folhetos bilaterais, além de espessamento extenso dos folhetos, que são menos flácidos que nos homens. ^113^

**Figura 4.8:**
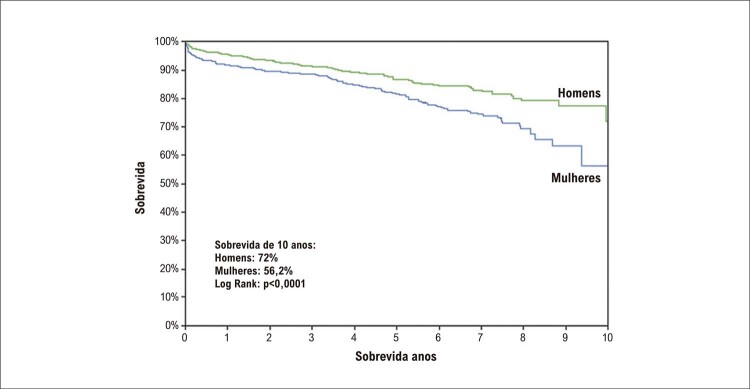
Evolução comparativa após cirurgia minimamente invasiva da valva mitral. Adaptado de Seeburger et al. 113

As mulheres apresentam menores volumes de regurgitação e dimensões atriais quando indexadas à superfície corporal; por conseguinte, muitas não alcançam critérios cirúrgicos baseados no aumento das cavidades ventriculares na insuficiência mitral, ^113^ o que pode resultar em piores resultados após a cirurgia. ^114^ Contudo, esses dados são controversos porque se fundamentam em estudos retrospectivos e com uma coorte muito limitada ( Figura 4.8 ).

No Brasil, a estenose mitral reumática é a principal causa de valvopatia adquirida em mulheres jovens; contudo, a calcificação do aparelho valvar mitral pode surgir com o progredir da idade. Em países industrializados, a erradicação da doença reumática cedeu espaço para as causas degenerativas, ^115^ em que predominam a calcificação com envolvimento da base dos folhetos e a ausência de fusão comissural, características estruturais que restringem a intervenção percutânea. Por essas razões, a substituição cirúrgica da valva mitral tem sido o tratamento preferível à comissurotomia percutânea/cirúrgica, exceto na presença de associação com morbidades que aumentem o risco operatório.

### 4.8.3. Doença Reumática

A prevenção primária da febre reumática aguda envolve o diagnóstico imediato e o tratamento antibiótico da infecção por estreptococos do grupo A com penicilina benzatina e outras alternativas. ^116^ Para ambos os sexos, medidas higiênicas são fundamentais para evitar a disseminação da doença e devem ser reforçadas. Pacientes com história de febre reumática aguda apresentam alto risco de recorrência e acometimento cardíaco com qualquer infecção subsequente por estreptococos do grupo A. Nas pacientes com risco de recorrência, a profilaxia antibiótica secundária deve ser a longo prazo em ambos os sexos, conforme preconizado pelas diretrizes atuais.

**Figura 4.9:**
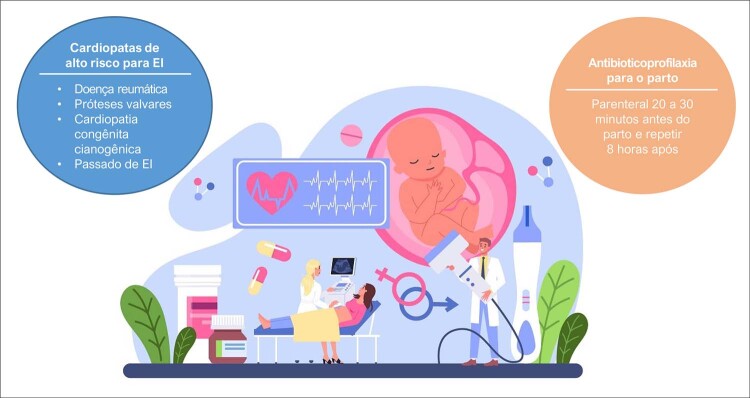
Orientações para a profilaxia de endocardite infecciosa na gravidez. EI: endocardite infecciosa.

Dentre as medidas preventivas em doença valvar nas mulheres, merece destaque a profilaxia da endocardite infecciosa na ocasião do parto. Embora controversa, o *Posicionamento sobre Cardiopatia e Gravidez da Sociedade Brasileira de Cardiologia* recomenda essa profilaxia em situações de presumível alto risco, indicando antibióticos específicos para o aparelho geniturinário, como ampicilina e gentamicina ou vancomicina, quando houver alergia à penicilina ( Figura 4.9 ). ^11^

## 4.9. Diabetes Mellitus, Pré-eclâmpsia e Doenças Hipertensivas na Gravidez

### 4.9.1. Diabetes Mellitus

A DM manifesta-se na gravidez como DM tipo 1, DM tipo 2 ou DG, estando associada a complicações materno-fetais, como PE, prematuridade e morte perinatal. A conduta para DM na gravidez deve ser individualizada de acordo com a presença dos fatores determinantes de prognóstico da gravidez, como nível glicêmico, tempo da doença, presença de comorbidades, lesões em órgãos-alvo, polidrâmnio, macrossomia fetal e outras malformações fetais. ^19^

A prevenção dessas complicações envolve mudança de estilo de vida, destacando-se controle do peso e dieta adequada antes e durante a gravidez. Uma meta-análise com 44 estudos avaliou a influência da atividade física e nutrição para o controle do peso corporal em 7.278 gestantes, mostrando redução média de 1,42 kg no ganho de peso e do risco de PE (RR 0,74, IC 0,60 - 0,92), sem comprometimento fetal. ^117^

A prática regular de exercício físico melhora a capacidade funcional, reduz o risco de depressão, previne ganho de peso excessivo e auxilia no controle dos distúrbios metabólicos e cardiovasculares, destacando-se PE e outras formas de HAS e de DM desenvolvidas na gravidez. Quando a atividade física é moderada ou alta, há redução do risco de DG em torno de 50% e da ocorrência de PE em 22% e 35%, respectivamente. O risco é ainda menor se a atividade física for iniciada antes e mantida desde o início da gravidez. Estudos mostram que 5 a 6 horas/semana de exercícios reduzem em 40% o risco de PE. Portanto, mulheres sem contraindicações obstétricas ou cardiovasculares devem ser fisicamente ativas durante toda a gravidez. Recomenda-se realizar 150 minutos de exercícios moderados, distribuídos em pelo menos três dias da semana, variando entre aeróbico, de resistência, alongamento e ioga. ^118^

### 4.9.2. Doenças Hipertensivas na Gravidez

As doenças hipertensivas na gravidez, que abrangem diferentes formas de HAS, estão entre as principais causas de complicações e mortalidade materna e perinatal em todo o mundo, sendo consideradas um marcador indiscutível de DCV no futuro. ^119^

Várias estratégias têm sido propostas para a prevenção da PE. Contudo, nenhuma é inequivocamente eficaz. Intervenções nutricionais, como vitaminas C e E, óleo de peixe, suplementação de alho, vitamina D, ácido fólico ou restrição de sódio, ^120^ não têm eficácia suficientemente comprovada. A reposição de cálcio é uma das poucas estratégias que mostraram benefício nas pacientes de alto risco de HAS ou PE, principalmente nas que consomem quantidade insuficiente de cálcio em sua dieta diária. Uma revisão sistemática de 13 estudos incluindo 15.730 pacientes com baixa ingesta diária de cálcio (< 600 mg/dia) mostrou que a reposição igual ou maior que 1,0 g/dia reduziu o risco relativo em 35% de HAS e em 55% de PE, sendo que doses mais elevadas (≥ 1g) tiveram maior benefício nesses desfechos. ^121^

**Figura 4.10:**
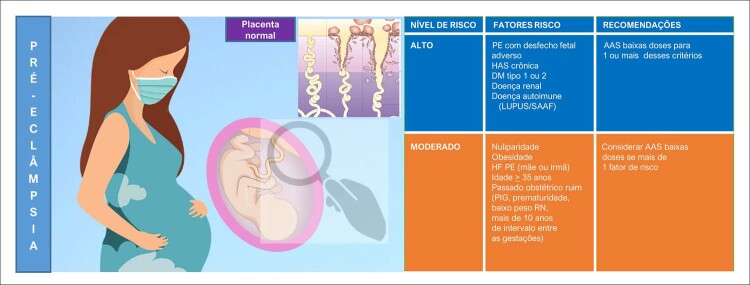
Recomendações para uso de ácido acetilsalicílico na profilaxia de pré-eclâmpsia. Adaptado do Posicionamento da Sociedade Brasileira de Cardiologia para Gravidez e Planejamento Familiar na Mulher Portadora de Cardiopatia 2020. 11 AAS: ácido acetilsalicílico; DM: diabetes mellitus; HAS: hipertensão arterial sistêmica; HF: história familiar; IMC: índice de massa corpórea; PE: pré-eclâmpsia; PIG: pequeno para idade gestacional; RN: recém-nascido; SAAF: síndrome do anticorpo antifosfolípide.

O uso do AAS entre 12 e 16 semanas de gravidez nas doses entre 75 mg e 150 mg diárias é considerado moderadamente eficaz em reduzir o risco de PE em pacientes de alto risco ( Figura 4.10 ). ^11^

O estudo ASPRE ( *Performance of Screening for Preterm Pre-Eclampsia),* utilizando um modelo de predição de PE (ultrassom Doppler no primeiro trimestre, medidas da pressão arterial média e marcadores inflamatórios), mostrou redução do risco relativo de 62% de PE com 150 mg de AAS à noite, com início entre a 11ª e a 13ª semana gestacional e término na 36ª semana gestacional. ^122^ Recente revisão corroborou redução do risco relativo de proteinúria na PE em 18% e do número necessário para tratar para 61, com baixo risco fetal, neonatal e de sangramento no pós-parto. ^123^

Em relação à HG como fator de risco para DCV no futuro, uma análise sistemática ^20^ de estudos mostrou que HG em primigestas foi associada a maior risco de DCV (RR, 1,45; IC 95%, 1,17-1,80) e DIC (RR, 1,46; IC 95%, 1,23-1,73), o que não ocorreu para AVC (RR, 1,26; IC 95%, 0,96-1,65) ou eventos tromboembólicos (RR, 0,88; IC 95%, 0,73–1,07). Mulheres com uma ou mais gestações com HG apresentaram maior risco de DCV (RR, 1,81; IC 95%, 1,42-2,31), DIC (RR, 1,83; IC 95%, 1,33-2,51) e insuficiência cardíaca (RR, 1,77; IC 95%, 1,47-2,13), mas não de AVC (RR, 1,50; IC 95%, 0,75-2,99). Pesquisas adicionais são necessárias para avaliar a correlação entre HG e DCV subsequente.

## 4.10. Gravidez na Adolescência

**Figura 4.11:**
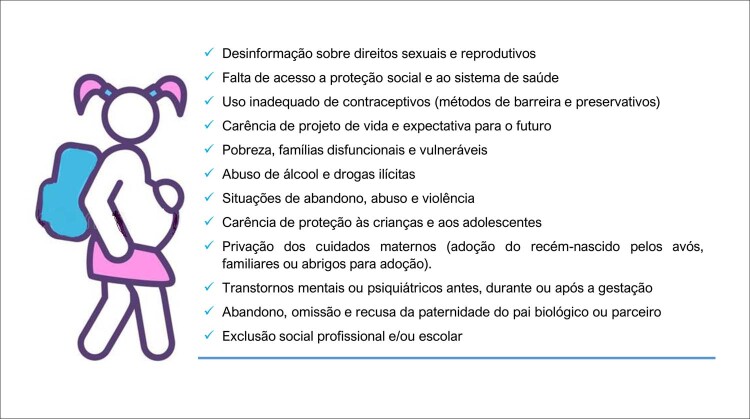
Fatores marcantes da gravidez na adolescência. 125

Dentre os problemas de saúde na adolescência (indivíduos entre 10 anos e 20 anos incompletos), a gravidez representa 400 mil casos por ano e as crianças nascidas de mães adolescentes representaram 18% dos nascidos vivos no Brasil em 2015, com predominância demográfica na região nordeste. ^124^ Entre os fatores que contribuem para o aumento da gravidez na adolescência, destacam-se: início precoce da atividade sexual, uso inadequado dos contraceptivos, dificuldades de acesso a programas de planejamento familiar e sobretudo desinformação sobre direitos sexuais e reprodutivos ( Figura 4.11 ). ^125^

**Figura 4.12:**
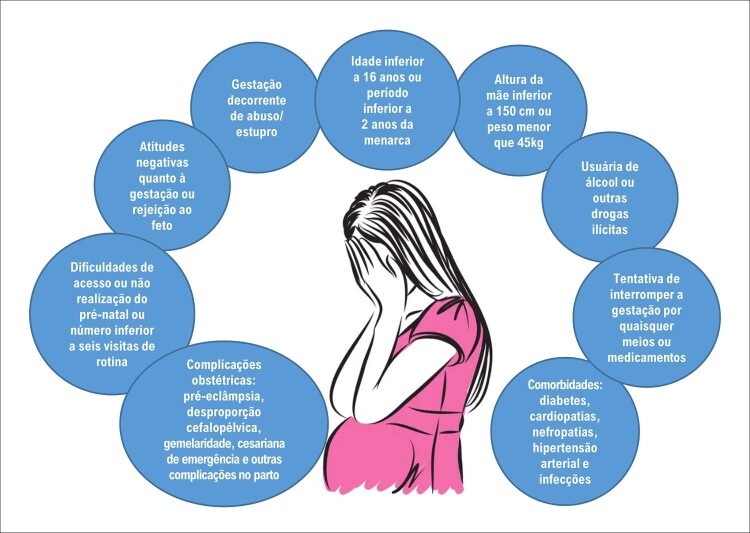
Fatores que contribuem para as complicações maternas da gravidez na adolescência. 125

De acordo com a OMS, a gravidez na adolescência aumenta complicações maternas, fetais e neonatais, além de agravar problemas socioeconômicos previamente existentes e influenciar o futuro de gerações. ^126^ Os fatores que contribuem para as complicações maternas da gravidez na adolescência estão demonstrados na Figura 4.12 . ^125^

De acordo com a Sociedade Brasileira de Pediatria, a abstinência sexual isoladamente não é uma estratégia para reduzir as taxas de gravidez na adolescência e estudos têm demonstrado que a abstinência não contribui para retardar o início da vida sexual nem reduzir o número de parceiros entre adolescentes. Segundo os estudos, adolescentes que receberam apenas educação sexual para abstinência não apresentaram efeito significativo na redução de gravidez (OR 0,7; IC 95%, 0,38-1,45; p = 0,38) em comparação àquelas que receberam educação sexual mais abrangente, que foi mais eficaz (OR 0,4; IC 95%, 0,22-0,69; p = 0,001). ^21^

O *Guia Prático sobre Prevenção da Gravidez na Adolescência da Sociedade Brasileira de Pediatria* considera que um dos mais importantes fatores de prevenção seja a educação sobre sexualidade e saúde reprodutiva apoiada em evidências científicas e em programas de promoção à saúde. ^125^ A instrução deve ser direcionada a aspectos biológicos, respeito recíproco, atividades sexuais com responsabilidade, além de uso de métodos contraceptivos seguros e eficazes na prevenção da gravidez e proteção contra infecções sexualmente transmissíveis. ^127^

A prescrição da contracepção para adolescentes é trabalhosa, pois requer tempo para construir um relacionamento de confiança. Entretanto, provedores pediátricos são os que mais merecem a confiança de adolescentes e familiares, sendo muitas vezes o único vínculo para o aconselhamento reprodutivo. ^128^ Estratégias para maior comprometimento e sucesso no aconselhamento reprodutivo são destacadas no Quadro 4.5 .

**Quadro 4.5:** Estratégias para a prevenção da gravidez na adolescência. 125

Reservar espaço da clínica para adolescentes com informações sobre saúde sexual e métodos de contracepção
Assegurar o sigilo das discussões sobre sua sexualidade
Apresentar os contraceptivos reforçando a eficácia, tolerância, efeitos colaterais, vantagens na contracepção e benefícios clínicos dos métodos.
Manter seguimento após a prescrição para identificar efeitos colaterais, adesão e satisfação
Regulamentar os anticoncepcionais de longa duração (LARCS) e os contraceptivos hormonais na prática pediátrica

Diretrizes atuais e melhores práticas para o fornecimento de contracepção incluem os Critérios de Elegibilidade Médica (MEC) ^132^ e as Recomendações de Práticas Selecionadas disponíveis na OMS e no Centro de Controle das Doenças dos Estados Unidos. ^129^ A Federação Brasileira de Ginecologia e Obstetrícia reforça o documento MEC-OMS em sua última versão, que aponta que somente a idade não é razão para atrasar o uso de qualquer método reversível e que questões sociais e comportamentais devem ser consideradas de modo individualizado. ^131^

**Figura 4.13:**
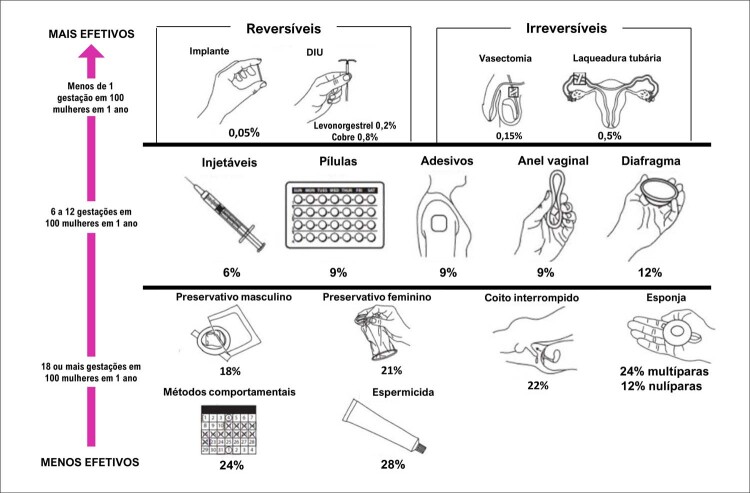
Métodos de contracepção e índice de Pearl (gestações/100 mulheres/ano).

Os contraceptivos podem ser divididos em hormonais e não hormonais (métodos comportamentais, mecânicos e de barreira) e apresentam falha variável, podendo sua eficácia ser calculada pelo índice de Pearl, que considera o número de gestações/100mulheres/ano ( Figura 4.13 ). ^128 , 132^

Fonte: Adaptado de U.S. Medical Eligibility Criteria for Contraceptive Use, 2016. ^132^

Os Quadros 4.6 , 4.7 e 4.8 demonstram as recomendações para o manejo de cardiotoxicidade, AVC, DAP, demência e outras doenças e situações específicas nas mulheres.

**Quadro 4.6:** Recomendações para o manejo de cardiotoxicidade nas mulheres.

Recomendações para o manejo da cardiotoxicidade nas mulheres			

Recomendação	CR	NE	Referências
Identificação e controle de fatores de risco para doenças cardiovasculares, como hipertensão, diabetes, obesidade, tabagismo e dislipidemia		**B**	^85,86^
Uso de drogas cardioprotetoras: drogas anti-remodelamento podem ser consideradas para pacientes com sinais de cardiotoxicidade subclínica ou pacientes de alto risco cardiovascular	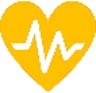 a	**B**	^85,86^
Pacientes do FE entre 40-50% devem iniciar tratamento com IECA/AT1 e betabloqueador antes do tratamento		**A**	^85,86^
Considerar o uso do dexrazoxano em pacientes com câncer de mama metastático com dose cumulativa elevada de antraciclina (doxorrubicina acima de 250 mg/m ^2^ )		**A**	^85,86^
Estratégias de vigilância com biomarcadores e imagens		**C**	^85^
O exercício aeróbico é considerado como estratégia não farmacológica promissora para prevenir cardiotoxicidade induzida por quimioterapia		**B**	^85,86^


CR: classe de recomendação; NE: nível de evidência; AT1: receptor de angiotensina tipo 1; IECA: inibidor da enzima de conversão da angiotensina; FE: fração de ejeção.

**Quadro 4.7:** Recomendações para o manejo de AVC, DAP e demência nas mulheres.

Recomendações para o manejo de AVC, DAP e demência nas mulheres			

Recomendação	CR	NE	Referências
Reforçar a conscientização das mulheres sobre a progressiva incidência de AVC e sua associação com complicações obstétricas (HG, partos prematuros, DG) e com contracepção hormonal		**A**	^87^
Alertar as mulheres sobre o risco progressivamente maior de AVC na presença de fatores de risco clássicos, como obesidade, dislipidemias, HAS e DM		**B**	^87^
O tratamento dos fatores de risco tradicionais confere proteção para ambos os sexos em relação à DAP		**B**	^100^
Na DAP, o reconhecimento de fatores de risco específicos do sexo, como complicações da gravidez, ou fatores predominantemente femininos, como depressão, permite adequada estratificação de risco e adoção de medidas de prevenção precoces		**B**	^101,102^
Sugere-se que o estrogênio possa ter importante papel na prevenção do depósito de amiloide na doença de Alzheimer, além de atuar na função cognitiva	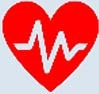	**A**	^107^

*CR: classe de recomendação; NE: nível de evidência; AVC: acidente vascular cerebral; HG: hipertensão gestacional; DG: diabetes gestacional; HAS: hipertensão arterial sistêmica; DM: diabetes mellitus; DAP: doença arterial periférica.*

**Quadro 4.8:** Recomendações para o manejo de situações específicas da gravidez.

Recomendações para o manejo de situações específicas da gravidez			

Recomendação	CR	NE	Referências
Na prevenção de endocardite infecciosa na ocasião do parto, a associação ampicilina 2,0g e gentamicina 1,5mg/kg deve ser feita uma hora antes do parto em pacientes de alto risco para endocardite infecciosa		**C**	^11^
Mudanças de estilo de vida, destacando-se controle do peso e dieta adequada, antes e durante a gravidez são importantes na prevenção de diabetes gestacional		**B**	^117^
Exercício físico regular durante a gravidez para mulheres sem contraindicação obstétrica: reduz risco de depressão, previne ganho de peso excessivo, auxilia no controle dos distúrbios metabólicos e cardiovasculares, destacando-se PE e outras formas de HAS e DM		**A**	^118^
Intervenções nutricionais, como vitaminas C e E, óleo de peixe, vitamina D, ácido fólico, restrição de sódio são medidas de prevenção para PE	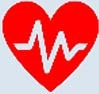	**C**	^119^
O uso do AAS antes da 16ª semana de gravidez reduz o risco de PE precoce (antes da 34ª semana) em pacientes de alto risco		**A**	^122^
A reposição de cálcio pode ter benefício na prevenção de PE em pacientes consideradas de alto risco que consomem quantidade diária insuficiente de cálcio (< 600 mg/dia)		**B**	^121^
A abstinência sexual como medida preventiva da gravidez na adolescência não contribui para retardar o início da vida sexual e nem tampouco reduzir a ocorrência de gravidez		**B**	^126^
A prevenção de gravidez na adolescência requer o esclarecimento sobre a eficácia, segurança e tolerância dos contraceptivos disponíveis e demanda supervisão até a idade adulta		**B**	^129^
A prescrição de contraceptivo deve ser apoiada nos critérios de elegibilidade da OMS		**B**	^132^

*CR: classe de recomendação; NE: nível de evidência; PE: pré-eclâmpsia; HAS: hipertensão arterial; DM: diabetes mellitus; AAS: ácido acetilsalicílico; OMS: Organização Mundial da Saúde.*

## 5. Peculiaridades dos Métodos Propedêuticos nas Mulheres

Na prática clínica, utilizamos escores de risco e características dos sintomas para estimar a probabilidade de RCV e identificar quem se beneficiaria de exames. A maioria dos modelos de predição superestima o risco, sendo os exames de imagem geralmente utilizados quando a probabilidade pré-teste é intermediária. Baseado no teorema de Bayes, a proporção de testes falso-positivos diminui quanto mais prevalente é a doença na população estudada, enquanto a de falso-negativos se reduz quanto menos prevalente é a doença na população. Nas mulheres, as lesões obstrutivas são menos comuns e a fisiopatologia da DIC tem suas peculiaridades, como acometimento de microcirculação, vasos menos calibrosos e maior reatividade vascular com consequente vasoespasmo. Assim, a sensibilidade e a especificidade dos testes nas mulheres podem ser diferentes daquelas nos homens. Este capítulo tem por objetivo apontar diferenças de interpretação, acurácia e indicação, quando houver, dos métodos de auxílio diagnóstico da DIC na mulher.

### 5.1. Eletrocardiograma

O ECG apresenta influências do sexo, relacionadas principalmente à magnitude dos sinais elétricos. A amplitude do QRS é menor nas mulheres, sobretudo nas derivações precordiais, como a onda S de V2 e R de V5, interferindo na acurácia do diagnóstico de HVE. As justificativas seriam: menor massa do ventrículo esquerdo e presença de mamas volumosas. O critério de Cornell para avaliação de HVE (soma da amplitude da onda R em aVL com onda S de V3) melhora a precisão ao considerar o sexo, sendo anormal > 28 mm em homens e > 20 mm em mulheres.

A amplitude do ponto J e da onda T é menor em mulheres. Em contrapartida, o intervalo QT corrigido é maior nelas, sendo anormal quando > 470 ms em mulheres e > 450 ms em homens. ^133^

### 5.2. Teste Ergométrico

O TE é seguro e fisiológico, indicado na investigação de isquemia miocárdica, de arritmias induzidas por esforço e dos preditores de prognóstico da DIC. Nas mulheres, os níveis menores de hemoglobina, o tamanho menor das coronárias, o aumento inapropriado de catecolaminas ao esforço e o estrogênio, por similaridades com a molécula do digital, podem provocar depressão do segmento ST falso-positiva.

Devido à alta prevalência de DAC não obstrutiva, lesões uniarteriais e doença microvascular, a acurácia varia com a probabilidade pré-teste da DIC. Segundo as diretrizes de ACC/AHA, o TE deve ser escolhido para avaliação de DIC em mulheres com probabilidade pré-teste intermediária, ECG normal e naquelas capazes de atingir o exercício máximo. O TE tem valor preditivo negativo similar em mulheres e homens (78% e 81%, respectivamente), sendo seu valor preditivo positivo em torno de 47%. A baixa amplitude eletrocardiográfica e o menor desempenho ao exercício prejudicam a avaliação da DIC em mulheres. ^25 , 134^

### 5.3. Ultrassonografia de Carótidas

A medida da EMI e a detecção de placa aterosclerótica são ferramentas auxiliares na avaliação do RCV. A EMI parece refletir a presença de FRCV e é diferente em homens e mulheres. A placa ateromatosa carotídea reflete a carga aterosclerótica, parece ser um preditor de RCV mais forte do que a EMI e não tem diferença entre os sexos. A medida da EMI para reclassificação de risco pode ser utilizada em alguns grupos específicos, nos quais a classificação por critérios habituais é difícil ou incompleta (hipercolesterolemia familiar, portadoras de doenças autoimunes, uso de medicações que induzem a elevação do colesterol) ou em mulheres com pelo menos dois FRCV. Essa medida foi estudada e publicada pelo projeto ELSA (Estudo Longitudinal da Saúde do Adulto), demonstrando não só diferença entre os sexos, mas entre etnias e, como sabido, entre faixas etárias de uma população genuinamente brasileira. Assim, podemos estratificar o risco em situações específicas com dados de nossa população. ^135^

A presença de placa ateromatosa é recomendada pelas diretrizes brasileiras e internacionais como fator agravante em pacientes de risco intermediário, situação em que muitas mulheres se enquadram. ^136^

### 5.4. Ecocardiografia

A ecocardiografia é a ferramenta mais utilizada na investigação diagnóstica e prognóstica CV em homens e mulheres, sem diferenças específicas do método entre os sexos. Além de diagnosticar alterações da contratilidade regional, identifica outras causas de dor torácica, como valvopatias, miocardiopatias, aortopatias e pericardiopatias. ^137^

A ecocardiografia de estresse é uma técnica atrativa, principalmente para mulheres jovens ou com risco de câncer de mama, pela ausência de exposição à radiação. Não existem muitos estudos avaliando diferenças no desempenho da ecocardiografia de estresse entre os sexos. Entretanto, fornece especificidade e precisão significativamente maiores do que o TE isolado, sem diferenças significativas entre homens e mulheres. A ecocardiografia de estresse com dobutamina *versus* TE para a detecção de DIC foi mais acurada em estenose de coronária >50% em mulheres com dor torácica, com sensibilidade de 70,4% vs 53,7% e especificidade de 94,6% vs 73,6%. A maior acurácia foi mantida após a exclusão de pacientes que não conseguiram atingir mais de 85% da frequência cardíaca prevista para a idade antes da indução de isquemia. A AHA recomenda a adição de imagens ao exercício na avaliação de mulheres de risco intermediário que têm um ECG basal anormal. ^25 , 138^

### 5.5. Cintilografia Miocárdica

A acurácia da cintilografia miocárdica de estresse na mulher é semelhante à da ecocardiografia de estresse, porém é menor se comparada à dos homens, possivelmente pelo menor diâmetro das câmaras cardíacas, levando a baixa resolução de imagens em gama-câmaras convencionais. Outro desafio é a atenuação causada pela mama, podendo levar a resultados falso-positivos. ^25^ Em meta-análise realizada comparando TE, cintilografia miocárdica de estresse e ecocardiografia de estresse, a sensibilidade e a especificidade de cada método foram 61% e 70%, 78% e 64%, e 86% e 79%, respectivamente, e foram similares às dos homens, exceto para o TE, que foi inferior nas mulheres. ^139^

O estudo WOMEN comparou a eficácia do TE com ou sem cintilografia miocárdica em mulheres sintomáticas com risco baixo/intermediário para DAC e capacidade para o exercício. Os autores mostraram que, apenas naquelas com risco pré-teste intermediário/alto, houve uma melhora na acurácia diagnóstica quando se combinou o TE com cintilografia miocárdica, sendo a acurácia maior para DAC obstrutiva: a cintilografia miocárdica de estresse apresentou sensibilidade de 78% (95% IC, 72%-83%) e o TE mostrou sensibilidade de 61% (95% IC, 54%-68%). A cintilografia miocárdica de estresse negativa mostrou excelente valor prognóstico em mulheres, com 99% de sobrevida sem eventos, incluindo idosas e diferentes etnias, sendo similar à dos homens. Portanto, a avaliação de probabilidade pré-teste para DAC é importante na decisão de qual método utilizar. ^140 , 141^

A exposição à radiação pela cintilografia em mulheres parece ser pouco nociva e, apesar do tema ser controverso, deve-se evitá-la nas jovens, sendo preferível a ecocardiografia de estresse.

### 5.6. Escore de Cálcio e Angiotomografia de Coronárias

Os escores populacionais preditores de RCV apresentam menor acurácia nas mulheres, em especial nas mais jovens. Atualmente, o CAC apresenta dose de radiação semelhante à mamografia (< 1 mSv) e tem igual acurácia em homens e mulheres para estratificação de risco e predição de eventos. Mulheres com CAC maior que zero apresentam risco de eventos maior do que homens. De forma semelhante, a calcificação arterial mamária, visualizada através da mamografia, guarda correlação com a presença de aterosclerose coronária. Se presente, deve indicar avaliação cardiológica.

O CAC tem valor prognóstico em mulheres assintomáticas com risco intermediário. É relatado que um CAC positivo em mulheres assintomáticas de risco intermediário está associado a uma taxa de eventos significativamente maior, incluindo morte, infarto agudo do miocárdio, cirurgia de RVM e intervenção coronária percutânea (3,3% naquelas com qualquer CAC *versus* 1,0% naquelas com CAC=0, após 37 meses de acompanhamento). ^27^

A Angio-TC é um teste anatômico não invasivo de DIC que fornece informações quanto à gravidade das obstruções, carga de placa e risco dessas placas se instabilizarem. Considerando as características da DIC em mulheres, a Angio-TC se torna um método capaz de evidenciar o padrão não obstrutivo mais prevalente, informação não avaliada pelos métodos funcionais. De fato, quando comparada a esses últimos, a Angio-TC gerou maior probabilidade de cateterismo com DAC obstrutiva, além de melhor correlação com eventos futuros. No contexto de dor torácica aguda, pelas características atípicas dessa queixa em mulheres no pronto-atendimento, a Angio-TC gerou maior impacto na redução do tempo de permanência hospitalar nas mulheres em relação aos homens. ^142 , 143^

### 5.7. Ressonância Magnética Cardíaca

A RMC é um método de alta acurácia na propedêutica de doenças cardiovasculares, sem necessidade de radiação. Isso a torna uma excelente opção em mulheres, especialmente naquelas em idade fértil, grávidas e em tratamento de câncer de mama. Em relação ao rastreamento de cardiotoxicidade, é sabido que cerca de 25% das pacientes com fração de ejeção ventricular < 50% na RNM não apresenta alterações ao ecocardiograma quando em propedêutica para cardiotoxicidade. ^144^

Na avaliação funcional da DIC, é conhecido o impacto das mamas e da menor massa miocárdica em estudos de imagem em mulheres. Tal limitação não se aplica à RMC, que demonstrou semelhante grau de acurácia em homens e mulheres. Considerando as características do padrão de DIC na população feminina, ou seja, a disparidade entre sintomas e achados anatômicos, a RMC tornou-se excelente opção para avaliação de alterações da perfusão miocárdica pela sua alta resolução espacial e pela sua igual performance, independentemente do sexo. Além disso, a RMC é capaz de evidenciar alterações perfusionais e/ou miocárdicas capazes de estender de forma mais abrangente o diagnóstico diferencial da dor torácica (ex: infartos por tromboembolismo ou dissecção coronariana) e ser particularmente útil na abordagem desse sintoma em mulheres. ^142 , 144^

### 5.8. Coronariografia

Há evidências de que o número de coronariografias realizadas em mulheres é menor do que em homens. Porém, quando se faz estratificação de risco para as mulheres, a indicação de coronariografia é semelhante à dos homens. ^144^ O estudo CURE mostra que a indicação de coronariografia, angioplastia e RVM foi inferior para mulheres (48% x 61%). ^145^ O *status* socioeconômico coloca a mulher de baixa renda em maior vulnerabilidade para acesso, o que poderia acarretar maior mortalidade a curto prazo após quadro de SCA. ^146^

Estudo transversal com dados do Registro VICTIM avaliou pacientes com diagnóstico de IMCSST oriundos de quatro hospitais (um público e três privados) com disponibilidade para realizar angioplastia primária. Foram incluídos 878 pacientes com IMCSST, 33,4% de mulheres. As mulheres apresentaram menores taxas de fibrinólise (2,3% no total, 1,7% nas mulheres e 2,6% nos homens) e de angioplastia primária (44% nas mulheres e 54,5% nos homens), resultando em maior mortalidade hospitalar (16,1% *versus* 6,7%). ^146^

Coronárias normais na coronariografia são mais comuns entre mulheres, inclusive na SCA, podendo representar doença microvascular, vasoespasmo ou trombólise espontânea. ^28^

O risco de complicações vasculares do procedimento pode ser maior entre as mulheres com artérias femorais mais finas, mesmo com compressão manual ou uso de *plug* com colágeno. As mulheres apresentam ainda maior tendência para desenvolver lesão renal aguda pós-contraste, por terem menor número de glomérulos, além de maior tendência a anemia. ^28^

As diferenças na abordagem diagnóstica dos métodos complementares usados para DIC entre os sexos, além de suas acurácias, vantagens e desvantagens nas mulheres são descritas no Quadro 5.1 .

**Quadro 5.1:** Métodos complementares diagnósticos: diferenças entre os sexos na interpretação, acurácia, vantagens e desvantagens quando empregados nas mulheres.

EXAME COMPLEMENTAR	Diferenças entre sexos na interpretação	Acurácia em mulheres	VANTAGENS	DESVANTAGENS
**ELETROCARDIOGRAMA**	Amplitude do QRS menor: onda S em V2 e R em V5 Intervalo QT é maior em mulheres (VR >470ms) Amplitude do ponto J e ST menor em mulheres	Sem diferenças entre os sexos	Utilizar critério de Cornell para diagnóstico de HVE em mulheres (VR > 20mm)	Interfere na acurácia do diagnóstico de HVE pelo critério de Sokolow
**TESTE ERGOMÉTRICO**	Depressão do ST como falso-positivo	Acurácia 47% Valor preditivo negativo semelhante ao dos homens: 78%	Baixo custo Bom valor preditivo negativo, quando realizado em pacientes que conseguem atingir o esforço máximo	Baixa acurácia
**US CARÓTIDAS**	EMI em mulheres é menor	Semelhante	Não tem irradiação, acessível e relativo baixo custo Placa aterosclerótica é maior preditora de risco que EMI Pode ser útil em subgrupos especiais onde os escores de risco são de pior aplicabilidade	Menos acurado que o CAC
**ECOCARDIOGRAFIA**	Sem diferenças entre os sexos	Eco de repouso: Sem diferença entre os sexos Parece semelhante para Eco de estresse	Eco de estresse é mais acurado que TE, especialmente quando o ECG de base for anormal Identifica outras doenças que podem causar dor precordial Sem radiação	Eco é examinador-dependente
**CINTILOGRAFIA**	Atenuação da imagem pela mama Pior resolução da imagem em câmeras convencionais	Sensibilidade 78% Especificidade 64%	Mais acurado do que o TE em mulheres com PPT intermediária/alta Valor prognóstico excelente, quando normal	Alguma radiação nas mamas
**ESCORE DE CÁLCIO CORONARIANO**	CAC maior do que zero em mulheres aponta para maior risco do que em homens	Acurácia semelhante para estratificação de risco e predição de eventos	Radiação semelhante à da mamografia (<1mSv)	Acessibilidade ao método
**ANGIOTOMOGRAFIA**	Sem diferença entre os sexos	Acurácia semelhante	Identifica DAC de padrão não obstrutivo mais prevalente em mulheres e melhor correlação com eventos futuros Redução do tempo de permanência hospitalar no pronto-atendimento	Acessibilidade ao método Radiação
**RESSONÂNCIA CARDÍACA**	Sem diferença entre os sexos	Acurácia semelhante	Sem radiação Evidencia alterações de perfusão e/ou miocárdicas, melhorando diagnóstico diferencial da dor torácica	Acessibilidade
**CORONARIOGRAFIA**	Sem diferença entre os sexos Mulheres apresentam mais coronárias normais na coronariografia, quando essa é solicitada	Sem diferenças entre os sexos	Pode diagnosticar angina vasoespástica, quando realizado para esse fim	Maior risco de complicações do procedimento: sangramento e lesão renal aguda Não avalia isquemia por doença microvascular

*CAC: escore de cálcio coronariano; DAC: doença arterial coronariana; Eco: ecocardiografia; EMI: espessura médio-intimal; HVE: hipertrofia ventricular esquerda; PPT: probabilidade pré-teste; TE: teste ergométrico; US: ultrassom; QT: quimioterápico; ECG: eletrocardiograma ; VR: valor de referência.*

O Quadro 5.2 apresenta as recomendações e níveis de evidência dos métodos diagnósticos de DIC nas mulheres.

**Quadro 5.2:** Recomendações dos métodos diagnósticos de doença isquêmica do coração nas mulheres.

Recomendações dos métodos diagnósticos de doença isquêmica do coração nas mulheres			

Recomendação	CR	NE	Ref.
O ECG na dor torácica mantém os graus de recomendação usuais, especialmente em mulheres de risco baixo e intermediário, atentando para as alterações relacionadas ao sexo		A	^25,133^
O teste de esforço está recomendado nas mulheres sintomáticas de risco intermediário para DIC, com ECG basal normal e capazes de atingir o exercício máximo		B	^25^
O teste de esforço com imagem está recomendado nas mulheres com alteração do segmento ST-T em repouso e incapazes de se exercitarem adequadamente		B	^25,140^
CAC: mulheres de risco baixo/intermediário ou risco inconsistente; incrementa a avaliação de risco. Alto valor preditivo negativo. Exame "ponte" para Angio-TC quando CAC positivo em pacientes de baixo risco	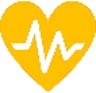 a	A	^143^
Angio-TC: estratificador de risco em mulheres; tão efetiva quanto teste de esforço	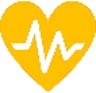 a	A	^143^
Angio-TC: alta acurácia preditiva para identificação de DAC obstrutiva e quando presente em mulheres de baixo risco	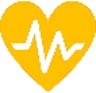 a	A	^143^
RNM indicada em mulheres na suspeita de DIC não obstrutiva (doença microvascular); vantagem por não ter radiação ionizante	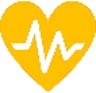 b	B	^144^
RNM em mulheres com probabilidade pré-teste intermediária e ECG com alteração do segmento ST-T em repouso e incapazes de se exercitarem adequadamente	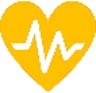 a	B	^144^

*Angio-TC: angiotomografia computadorizada de coronárias; CAC: escore de cálcio coronariano; CR: classe de recomendação; DIC: doença isquêmica do coração; ECG: eletrocardiograma; NE: nível de evidência; RNM: ressonância nuclear magnética; DAC: Doença Arterial Coronariana.*

## 6. Representação de Mulheres nos Estudos Clínicos sobre Fatores de Risco e Doença Cardiovascular

Sexo e gênero associam-se a riscos ambientais e ocupacionais, a comportamentos de risco, a cuidados em saúde e à percepção desses cuidados pelos seres humanos, influenciando a prevalência das doenças e o resultado do seu tratamento de forma diferente em homens e mulheres. ^147 , 148^ Como a farmacocinética e a farmacodinâmica dos medicamentos diferem entre os sexos, ocorrem diferenças no perfil de eventos adversos, bem como no resultado dos tratamentos entre homens e mulheres. ^148^ Esse conhecimento determina a necessidade de que os estudos clínicos sobre diagnóstico e tratamento mantenham em suas amostras a representatividade de homens e mulheres observada na população humana e proporcional à prevalência da doença objeto do estudo.

Entretanto, a análise de estudos clínicos realizados nas últimas décadas no campo da DCV e dos seus fatores de risco demonstra que, apesar de alguns avanços, mulheres ainda são sub-representadas nesses estudos. ^34 , 67 , 149^

Em 121 estudos clínicos financiados pelo *National Heart, Lung and Blood Institute (NHLBI)* entre 1965 e 1998, excluídos os estudos realizados com apenas um dos sexos, mulheres representaram em média 38% da amostra, não havendo mudanças significativas nesse perfil ao longo do tempo. ^67^ A análise demonstrou que a representação das mulheres nos estudos de DAC e de HAS foi proporcional à prevalência dessas doenças no sexo feminino; entretanto, nos estudos de IC e de arritmias, a representação das mulheres foi abaixo da prevalência dessas doenças no sexo feminino. ^87^

A revisão sistemática de 135 estudos clínicos utilizados para apoiar as recomendações da atualização da diretriz de prevenção cardiovascular em mulheres da *American Heart Association* de 2007 *(Evidence-based guidelines for cardiovascular disease prevention in women: 2007 update)* demonstrou que a representação de mulheres nos estudos publicados entre 1970 e 2006 aumentou ao longo do tempo (18% em 1970 e 34% em 2006), sendo maior na prevenção primária do que na secundária (43% *versus* 27%) e nos estudos de HAS, DM e AVC, mas menor naqueles de IC, DAC e dislipidemia. ^150^ Apesar desse aumento, os autores concluíram que ainda havia sub-representação das mulheres nos estudos clínicos de DCV e de seus fatores de risco e chamaram a atenção para o fato de que em apenas 31% dos estudos houve discussão dos resultados à luz das diferenças entre os sexos. ^150^

A análise da participação das mulheres e da segurança e eficácia relatadas por sexo em estudos clínicos de DCV que apoiou 36 aprovações de medicamentos de 2005 a 2015 pelo *Food and Drug Administration* dos EUA demonstrou que mulheres representaram em média 46% da amostra desses estudos. ^34^ Os autores concluíram que houve representação adequada das mulheres nos estudos clínicos sobre HAS, FA e hipertensão pulmonar e sub-representação nos estudos sobre IC, DAC e SCA. Foram registradas diferenças mínimas entre os sexos nos perfis de eficácia e segurança dos medicamentos avaliados e aprovados. ^34^

A participação das mulheres em 740 estudos clínicos de DCV registrados no *ClinicalTrials.gov* (https://clinicaltrials.gov) entre 2010 e 2017 foi, em média, 38,2% do total de 862.652 adultos. As mulheres eram mais jovens (≤ 55 anos) e tinham participação em estudos de intervenção no estilo de vida e sobre HAS, FA e hipertensão pulmonar, apesar de esses estudos serem realizados com menor duração. Entretanto, em estudos sobre procedimentos de alta complexidade, DAC, SCA e IC, as mulheres foram menos representadas. ^151^

**Figura 6.1:**
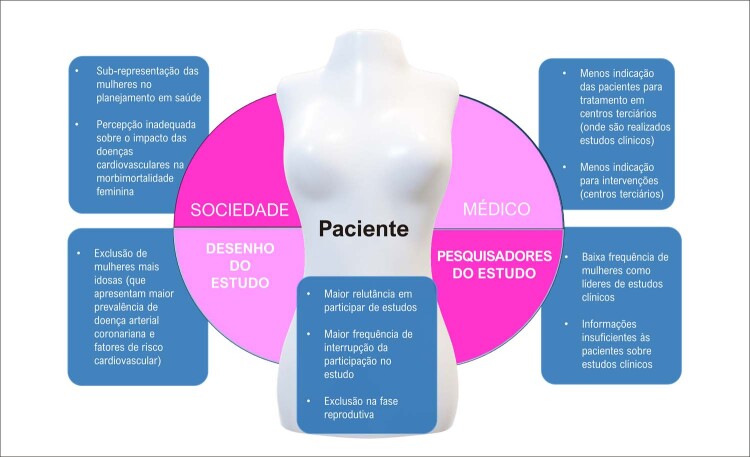
Barreiras que contribuem para a baixa representação de mulheres em estudos clínicos sobre doenças cardiovasculares e seus fatores de risco. 152,153

A análise desse cenário evidencia a existência de inúmeras barreiras a serem transpostas para que se obtenha equidade no cenário da representação dos sujeitos nos estudos clínicos, de forma a contemplar adequadamente as mulheres em geral, bem como as mulheres de populações minoritárias (étnicas e raciais). ^152 , 153^ Essas barreiras parecem relacionadas às pacientes, aos médicos assistentes, à equipe de pesquisa, ao desenho do estudo e à sociedade ^152^ e estão apresentadas na Figura 6.1 .

Recomenda-se que: A) os jornais científicos solicitem dos autores de estudos clínicos cardiovasculares análise de diferenças sexo/gênero-específicas; B) ocorra integração da equidade em saúde no desenho dos estudos clínicos, utilizando uma estrutura como *PROGRESS Plus (* ***P*** *lace of residence;* ***R*** *ace;* ***O*** *ccupation;* ***G*** *ender;* ***R*** *eligion;* ***E*** *ducation;* ***S*** *ocioeconomic status;* ***S*** *ocial capital; Plus=others)* ; C) mais mulheres passem a constituir os grupos de investigadores dos estudos clínicos; D) seja promovida a educação médica à luz das particularidades de sexo e gênero; E) ocorra facilitação do acesso da população aos centros que realizam pesquisa clínica. ^152 , 153^

## 7. Medidas de Prevenção Primária nas Mulheres

O cuidado da mulher na APS se faz através das Redes de Atenção à Saúde, seguindo as diretrizes da Política Nacional de Atenção Integral à Saúde da Mulher do Ministério da Saúde. ^154 , 155^ Através das Unidades Básicas de Saúde, são implementadas ações de saúde pública para a redução da morbimortalidade da mulher ao longo de seu ciclo de vida, extensivas à família e à comunidade em que está inserida. ^154 , 155^

O planejamento reprodutivo, conjunto de ações de regulação da fecundidade, e as práticas anticoncepcionais que são predominantemente assumidas pelas mulheres, incluindo lésbicas e bissexuais, cujo desejo ou direito à maternidade precisa ser garantido, devem ser acolhidos pelas equipes de APS, que precisam iniciar as medidas necessárias para sua implementação. ^154 , 155^

No pré-natal, as Equipes de Saúde da Família devem estratificar o risco materno-fetal, considerando a história reprodutiva anterior, as características individuais e as condições sociodemográficas desfavoráveis. A captação de mulheres em idade fértil (10-49 anos) é essencial para estratificar a gravidez com risco de complicações como: extremos de idade (menor que 15 anos e maior que 35 anos), baixo peso e sobrepeso ou obesidade, situação familiar insegura, não aceitação da gravidez (principalmente em adolescentes), baixa escolaridade, portadoras de cardiopatias, hipertensas crônicas e/ou história prévia de doença hipertensiva da gravidez e diabetes mellitus gestacional. No puerpério, deve-se dar especial atenção às condições psíquicas e sociais da mulher, proteção e apoio ao aleitamento materno, com amamentação exclusiva nos primeiros seis meses de vida da criança. ^154 , 155^

A transição da menopausa está associada com risco aumentado de DCV, atribuída principalmente a dislipidemia aterogênica, obesidade central e resistência à insulina, além de aumento do risco de hipertensão arterial. ^156^ A THM deve ser individualizada, considerando os riscos pessoais e familiares para neoplasia ginecológica e a estratificação do risco cardiovascular. A THM deve ser acompanhada sistematicamente na APS. A reposição estrogênica pode reduzir os níveis séricos do colesterol total e LDL-c pelo aumento da síntese de receptores de LDL-c. Benefícios da THM foram observados quando introduzida antes dos 60 anos e em mulheres que cessaram a menstruação há menos de 10 anos (preferencialmente nos primeiros 5 anos), com malefícios fora da janela de oportunidade, aumentando o risco cardiovascular. ^49^

Em 2019, por meio do Programa Previne Brasil, os indicadores de saúde da mulher pactuados foram: gestantes cadastradas pelas APS, média de atendimentos de pré-natal, pré-natal iniciado no 1º trimestre, gestantes com vacina em dia, visitas domiciliares para gestantes, gravidez na adolescência, proporção de parto normal, óbitos maternos, exames citopatológicos do colo do útero, rastreamento com mamografia em mulheres de 50-69 anos, entre outros. Observa-se que nenhum indicador de saúde cardiovascular foi pactuado naquele programa. ^157^ Para além disso, a OCDE divulgou recentemente um relatório sobre a APS no Brasil, onde não há menção a DCV em mulheres. Para essas, a OCDE apenas faz sugestões para abordagem das neoplasias de mama e ovário; no entanto, em termos porcentuais, as DCV causam duas vezes mais mortes do que todas as neoplasias no Brasil. ^158^

**Figura 7.1:**
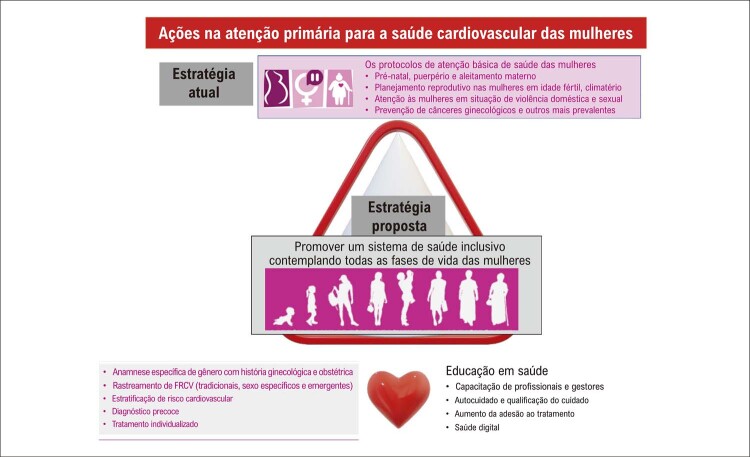
Ações estratégicas na Atenção Primária para a saúde cardiovascular das mulheres. FRCV: fatores de risco cardiovascular.

Faz-se necessária uma mudança de paradigma nas políticas públicas voltadas para a saúde integral das mulheres, especialmente quando se considera que as DCV são a principal causa de morte das mulheres na maior parte de seu ciclo de vida ( Figura 7.1 ).

Terapias não farmacológicas que incorporam modificação do estilo de vida (exercícios, perda de peso, cessação do tabagismo e dieta saudável) devem ser recomendadas como estratégia de primeira linha na APS. A eficácia dessas medidas está diretamente associada à melhor compreensão, sensibilização e motivação construídas durante o acompanhamento pela Equipe de Saúde da Família. A abordagem sobre o comportamento alimentar saudável, a prática de atividade física regular e a saúde mental deve ser individualizada. ^26^ Exemplos de ferramentas validadas para avaliação das pacientes, adicionadas à sua história clínica e ao exame físico, são o Guia Alimentar para a População Brasileira, as Diretrizes da Organização Mundial da Saúde para Atividade Física e Comportamento Sedentário, as Escalas de Estresse e Depressão e os Questionários de Espiritualidade. ^26^

De todas as intervenções possíveis na APS, a mais custo-efetiva é a cessação do tabagismo. Os efeitos nocivos do cigarro, comum e eletrônico, são maiores nas mulheres, especificamente pela perda do efeito protetor do estrogênio no endotélio dos vasos. ^159^

No Brasil e no mundo, a violência contra as mulheres constitui um sério problema de saúde pública, por ser uma das principais causas de morbidade e mortalidade femininas. Na APS, devem ser desenvolvidas ações que possam garantir os direitos sexuais na perspectiva da autonomia das mulheres sobre seu corpo. ^160^

Diante dos desafios para o controle das DCV no país e reconhecendo a APS como importante estratégia para as ações de promoção da saúde, de prevenção dos fatores de risco, de diagnóstico precoce e de cuidado às pessoas com DCV, sugere-se uma revisão crítica para aprimoramento das ações de saúde voltadas para as mulheres nas Equipes de Saúde da Família e na APS ( Figura 7.1 ). ^160^

## 8. Burnout, Qualidade de Vida e Espiritualidade nas Mulheres

### 8.1. Burnout

*Burnout* é uma síndrome psicológica resultante do estresse crônico no trabalho, caracterizada por exaustão emocional, sentimentos de cinismo/despersonalização e falta de eficácia profissional. ^161^ As dimensões de *burnout* foram significativamente associadas a um risco aumentado para doenças, independentemente de fatores sociodemográficos e sintomas depressivos. Em um estudo com 5.671 participantes [predominantemente médicos, idade média de 44,1 anos (variação, 18 anos a 70 anos), 62,4% mulheres], um aplicativo digital de saúde móvel foi usado para uma pesquisa *online* de *burnout* profissional medido com o *Maslach Burnout Inventory General Survey* . Por meio de análise de rede e regressão logística, o estudo mostrou a associação de alta exaustão emocional com hipertensão arterial e outras doenças crônicas após ajuste para idade, sexo, escolaridade e sintomas depressivos. ^162^

**Figura 8.1:**
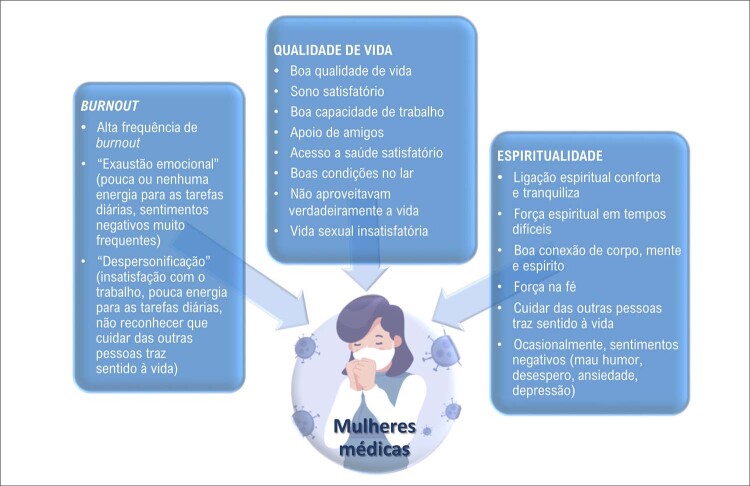
Estudo realizado com médicas brasileiras durante a pandemia pela COVID-19. Achados ressaltaram a maior susceptibilidade das mulheres aos fatores psicossociais, com significativo impacto na qualidade de vida, que pode gerar burnout, minimizado pela espiritualidade, especialmente em tempos de pandemia de COVID-19. 162

As condições de trabalho têm impacto conhecido na saúde dos trabalhadores e as mulheres, por estarem mais inseridas no mercado de trabalho e sobrecarregadas com a atividade laboral dupla, apresentam altas taxas de *burnout* . Trabalho realizado com médicas brasileiras, durante a pandemia pela COVID-19, demonstrou que 61,6% apresentaram sinais de *burnout* , com exaustão emocional, sentimentos negativos frequentes e insatisfação com a sua capacidade para o trabalho ( Figura 8.1 ). ^162^

Importante mencionar que as mulheres são mais expostas ao estresse e adversidades psicossociais do que os homens, além de serem mais vulneráveis aos efeitos dessas exposições. Depressão, fatores socioeconômicos desfavoráveis e transtorno de estresse pós-traumático são mais prevalentes em mulheres do que em homens e tendem a mostrar associações mais robustas com risco cardiometabólico nas mulheres. ^67^

### 8.2. Qualidade de Vida

Segundo a Organização Mundial da Saúde, a QV é a “percepção de um indivíduo de sua posição na vida no contexto da cultura e dos sistemas de valores em que vive e em relação aos seus objetivos, expectativas, padrões e preocupações”. A QV está diretamente relacionada com a maior incidência de doenças crônicas, em particular as DCV. Os indicadores-padrão da QV incluem emprego, riqueza, meio ambiente, saúde física e mental, educação, recreação e lazer, socialização, crenças religiosas, segurança, proteção e liberdade. ^163^

Estudo que avaliou a QV em médicas brasileiras reportou que elas consideraram ter boa QV (71,7%) e estar satisfeitas com sua saúde (55%), porém 64,8% não aproveitaram verdadeiramente a vida. Consideraram ainda satisfatórios os seguintes aspectos de suas vidas: sono, 62,9%; capacidade de realizar tarefas diárias, 54,7%; capacidade para o trabalho, 64,4%; relações pessoais, 57,7%; apoio de amigos, 61%; condições do lar, 84%; e acesso à saúde, 81,4%. Apenas 36,6% consideraram a sua vida sexual satisfatória e cerca de 94% tiveram, pelo menos ocasionalmente, sentimentos negativos ( Figura 8.1 ). ^162^

Estudo realizado com 1.387 mulheres de Uberaba, MG, avaliou a QV por meio do WHOQOL-Brief, segundo os quatro domínios. O estudo identificou que as mulheres com DCV tinham idade maior que 50 anos, baixa escolaridade e apresentavam valores mais baixos, estatisticamente significativos, para todos os quatro domínios, quando comparadas com mulheres sem doença ou com doença respiratória crônica. O domínio que obteve menor pontuação para todos os grupos foi o meio ambiente, que se relaciona com a condição socioeconômica por conter questões ligadas aos recursos financeiros, oportunidades de lazer, segurança, entre outros. Esses achados ressaltam a maior susceptibilidade das mulheres com DCV aos fatores psicossociais, com significativo impacto na QV. ^164^

### 8.3 Espiritualidade

“Espiritualidade é um conjunto de valores morais, mentais e emocionais que norteiam pensamentos, comportamentos e atitudes nas circunstâncias da vida de relacionamento intra- e interpessoal, com aspecto de ser motivado pela vontade, passível de observação e de mensuração”. Há de se diferenciar de religiosidade, já que “religião é um sistema organizado de crenças, práticas e símbolos destinados a facilitar a proximidade com o transcendente ou o Divino e a fomentar a compreensão do relacionamento e das responsabilidades de uma pessoa com os outros que vivem em comunidade”. ^26^

Além dos aspectos comportamentais, está demonstrada a relação benéfica entre espiritualidade, religiosidade, variáveis fisiológicas e fisiopatológicas de muitas entidades clínicas, incluindo-se as mulheres com DCV. ^165^ Anamnese espiritual vem sendo introduzida cada vez mais nos consultórios e hospitais, fazendo parte da história clínica, especialmente nas doenças graves, crônicas, progressivas, debilitantes e terminais. Existem ferramentas validadas para avaliação, baseadas em escalas de saúde (FICA, FAITH, SPIRIT e HOPE). ^26 , 163 - 173^

Estudo com médicas brasileiras, utilizando um formulário com questões baseadas no instrumento de teste de campo de Qualidade de Vida da Organização Mundial da Saúde – módulo Espiritualidade, Religiosidade e Crenças Pessoais, relatou que as respondentes acreditavam que a espiritualidade lhes trazia conforto e segurança (73,2%) e força espiritual em tempos difíceis (70,6%), com boa conexão de corpo, mente e espírito (67,8%), embora apenas 53,4% relatassem paz interior e 50,7% relatassem serem otimistas. O estudo reportou ainda que 72,7% das participantes encontravam força na fé e 44,3% encontravam apoio em comunidades religiosas ou espirituais ( Figura 8.1 ). ^162^

Em conclusão, a saúde necessita ser entendida no contexto físico, social, psicoemocional e espiritual, respeitando-se a individualidade e a singularidade do sexo feminino. ^44^

O Quadro 8.1 demonstra as recomendações para o manejo da espiritualidade e da saúde das mulheres.

**Quadro 8.1:** Recomendações para o manejo da espiritualidade e saúde das mulheres.

Recomendações para práticas em espiritualidade e saúde da mulher			

Recomendação	CR	NE	Ref
Rastreamento breve de espiritualidade e religiosidade		**B**	^26,163-173^
Anamnese espiritual de pacientes com doenças crônicas ou de prognóstico reservado		**B**	^26,163-173^
Respeitar e apoiar religiões, crenças e rituais pessoais do paciente que não sejam prejudiciais ao tratamento		**C**	^26,163-173^
Suporte por profissional capacitado aos pacientes em sofrimento ou com demandas espirituais		**C**	^26,163-173^
Religiosidade organizacional associa-se a redução de mortalidade		**B**	^26,163-173^
Anamnese espiritual de pacientes estáveis ou ambulatoriais	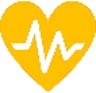 Ia	**B**	^26,163-173^
Questionários DUREL, FICA, HOPE ou FAITH para avaliar espiritualidade	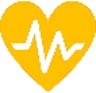 a	**B**	^26,163-173^
Meditação, técnicas de relaxamento e combate ao estresse	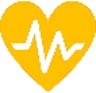 a	**B**	^26,163-173^
Técnicas de fortalecimento espiritual como perdão, gratidão e resiliência	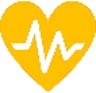 b	**C**	^26,163-173^

CR: classe de recomendação; NE: nível de evidência.

## 9. Implicações Cardiovasculares da COVID-19 na Gestação

**Figura 9.1:**
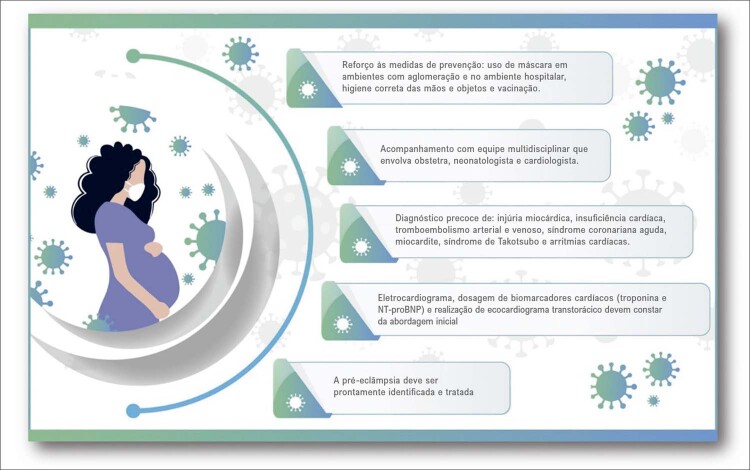
Medidas de prevenção para a gestante com COVID-19. NT-proBNP: Fragmento N-terminal do peptídeo natriurético tipo B.

As principais complicações descritas nas pacientes com COVID-19 são: injúria miocárdica, insuficiência cardíaca, tromboembolismo arterial e venoso, síndrome coronariana aguda, miocardite, síndrome de Takotsubo e arritmias cardíacas. ^191^ Essas complicações são mais frequentes nas idosas, com fatores de risco para doenças cardiovasculares e com comorbidades. ^191^ Grande preocupação ocorre com as grávidas ou puérperas, que também são mais susceptíveis às formas graves da COVID-19, com parto prematuro ou cesariano de emergência, elevando o risco de morte neonatal e materna ( Figura 9.1 ). ^192^

Durante a gravidez, a resposta imune predomina através das células *T-helper* 2 (Th2), que protegem o feto, mas tornam a mãe mais vulnerável a infecções virais, que são mais eficazmente combatidas pelas células Th1. ^193^ A transmissão vertical do SARS-CoV-2 pode ocorrer por via transplacentária e durante o parto e a amamentação. A capacidade de transmissão do SARS-CoV-2 pelo sangue ainda é incerta. ^162^ As alterações fisiológicas da gravidez cursam com aumento do volume plasmático, do volume sistólico e do débito cardíaco na primeira metade da gravidez e um aumento gradual da frequência cardíaca, além de diminuição das resistências vasculares sistêmica e pulmonar. Além disso, a gravidez é um estado de hipercoagulabilidade associado com risco aumentado de tromboembolismo venoso e pulmonar. ^194^

O quadro clínico da COVID-19 apresenta algumas particularidades nas grávidas, como persistência de sintomas por tempo prolongado e menor frequência de febre e mialgia, em comparação às não grávidas. Os principais fatores de risco para infecção grave são: aumento da idade materna, índice de massa corporal elevado e comorbidades preexistentes, como hipertensão, pré-eclâmpsia e diabetes. ^194^ A gravidez foi associada a infecção grave em 10%, admissão em unidade de terapia intensiva em 4%, ventilação mecânica em 3% e utilização de membrana extracorpórea em 0,2%. ^194 , 195^

As complicações perinatais também foram mais prevalentes. Quando comparadas a grávidas sem infecção, as com COVID-19 tiveram maior risco de parto prematuro e natimorto. No geral, 33% dos recém-nascidos de mulheres com COVID-19 foram admitidos em unidade de terapia intensiva neonatal. ^192 , 194^ Mesmo após ajuste para raça, comorbidades e idade, as gestantes foram mais propensas a evoluírem para óbito em comparação às mulheres não grávidas. ^196^

As complicações cardiovasculares graves na COVID-19 são a lesão miocárdica aguda, miocardite, arritmia, insuficiência cardíaca fulminante com choque cardiogênico e dissecções espontâneas das artérias coronárias e vertebrais, que apresentam mortalidade aumentada. ^192^

Os potenciais contribuintes para lesão cardíaca aguda no cenário de COVID-19 incluem: ^191^ alterações agudas na demanda e oferta do miocárdio devido a taquicardia, hipotensão e hipoxemia, resultando em infarto do miocárdio tipo 2; ^192^ síndrome coronariana aguda por aterotrombose aguda em meio trombótico e inflamatório induzido por vírus; ^193^ disfunção microvascular devido a microtrombos difusos ou lesão vascular; ^194^ cardiomiopatia relacionada ao estresse (síndrome de Takotsubo); ^195^ lesão miocárdica não isquêmica devido a tempestade de citocinas hiperinflamatória; ^196^ ou toxicidade viral direta de cardiomiócitos e miocardite. O tratamento medicamentoso da insuficiência cardíaca na gravidez tem particularidades que devem ser seguidas para evitar teratogenicidade. ^193^

Nos casos de síndrome coronariana aguda com supra de ST, a coronariografia deve ser preferencialmente realizada, seguida de tratamento percutâneo. Em 15 pacientes grávidas com COVID-19 e injúria miocárdica, 13,3% apresentaram fibrilação atrial, 2 apresentaram taquicardia supraventricular e 2 evoluíram para *torsades de pointes.* ^197^ Recomenda-se cautela com o uso de medicações que possam prolongar o intervalo QT.

A COVID-19 pode tanto predispor à ocorrência de pré-eclâmpsia quanto agravar sua evolução. O vírus gera no organismo um estado fisiopatológico semelhante ao da pré-eclâmpsia, caracterizado por hiperinflamação sistêmica, lesão endotelial direta, trombogênese e desregulação imunológica, e afeta o sistema renina-angiotensina-aldosterona, que eleva a incidência de pré-eclâmpsia nas pacientes infectadas. ^198^

A decisão sobre interrupção da gravidez deve basear-se nas diretrizes habituais, que levam em consideração a idade gestacional, as condições hemodinâmicas, o sofrimento fetal e o risco materno. A COVID-19 não complicada não deve ser indicação de interrupção de gravidez. O tipo de parto também deve seguir as recomendações obstétricas. ^199^

## 10. Perspectivas Futuras para a Melhoria do Cuidado Cardiovascular das Mulheres

Atendimento por uma equipe multidisciplinar é sempre desejável para ações preventivas e de tratamento no acompanhamento da mulher com DCV. Aumentar a conscientização passa pela criação de programas integrativos, envolvendo líderes e agentes comunitários, com novos centros especializados na saúde cardiovascular das mulheres. Importante informar especificamente sobre o autocuidado para a prevenção das DCV, o uso apropriado de contraceptivos orais, a realização do pré-natal, o acompanhamento a longo prazo dos FRCV e os aspectos psicossociais e socioeconômicos que envolvem as DCV no sexo feminino. ^200^

A prevenção primária na mulher deve se concentrar no tratamento eficaz dos fatores de risco tradicionais, como HAS, riscos dietéticos, dislipidemia, diabetes, obesidade e inatividade física. A ausência de escores de risco específicos para as mulheres sinaliza para a estratificação de risco de longo prazo, considerando o curso de vida e os fatores específicos para o sexo feminino, como a presença de depressão, e os fatores psicossociais. ^200^ Devemos ressaltar que a frequência de ICFEp nas mulheres é maior do que nos homens; além disso, as mulheres apresentam mais ICFEp do que ICFEr, devendo o tratamento ser instituído com o diagnóstico clínico de IC, independentemente da fração de ejeção, devido à maior relevância do déficit cognitivo associado a IC no sexo feminino. ^201^

A HAS é o maior fator de risco atribuível para o desenvolvimento de desfechos cardiovasculares em ambos os sexos. O controle da PA é fundamental para a redução da incidência de ICFEp e de ICFEr. ^202 , 203^ Entretanto, as mulheres apresentam menores taxas de controle que os homens, principalmente em faixas etárias mais avançadas. ^204^ Estratégias que contemplem a medida da PA fora do consultório, tais como a auto-aferição da PA, a monitorização residencial e ambulatorial da

PA com o uso de equipamentos semiautomáticos, são desejáveis. Além disso, o monitoramento à distância por meio de plataformas digitais e aplicativos, com a monitorização contínua da PA sem *cuffs* e em sincronia com *smartphones,* representa o emprego da tecnologia e *wearables* de fácil acesso em favor dos resultados clínicos e provavelmente auxiliarão no melhor controle da PA. ^205 , 206^

Devido às evoluções médica, cirúrgica e tecnológica nas últimas décadas, mais de 90% dos indivíduos com CC que nascem atualmente sobrevivem até a idade adulta. A atenção especial às mulheres com CC requer estratégias de planejamento para atender às necessidades dessa população. ^11^ Recomenda-se, portanto, a criação de centros especializados em CC com equipes multidisciplinares que incluam médicos, enfermeiros, psicólogos e assistentes sociais para o aconselhamento à contracepção ou para o planejamento de uma gravidez. Os riscos para a paciente adulta com CC, tratada ou não na infância, especialmente no que diz respeito à anticoncepção e gravidez, devem sempre ser considerados na medida em que 3% a 10% dos filhos desse grupo de mães podem apresentar lesões cardíacas congênitas. ^207^ Nas mulheres cardiopatas em idade fértil, a abordagem tem que ser realizada por uma equipe multidisciplinar, um “time de cardio-obstetrícia”, para aconselhamento pré-concepcional, definir planejamento da gravidez, parto e puerpério, incluindo atendimento especializado cardiológico, e planejamento familiar após o parto. ^208^

A prevenção e o tratamento de DCV em mulheres necessitam de sistemas de saúde robustos apoiados por profissionais com conhecimento das especificidades das DCV em mulheres, além de esforços coordenados com parcerias produtivas entre a sociedade política, médicos, pesquisadores e a comunidade. ^70^

**Figura 10.1:**
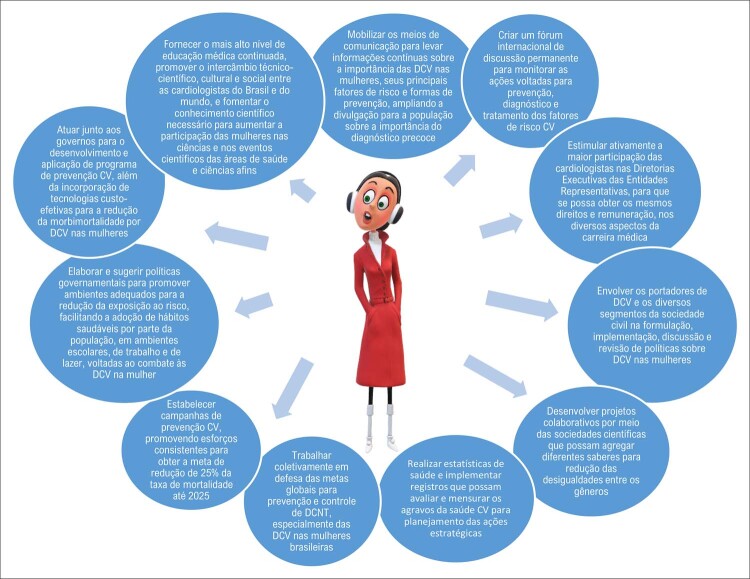
Perspectivas futuras para a abordagem das doenças cardiovasculares nas mulheres de acordo com a Carta das Mulheres. 4 CV: cardiovascular; DCNT: doenças crônicas não transmissíveis; DCV: doença cardiovascular

A Carta das Mulheres, publicada em 2019, sugere a criação de grupo permanente para a promoção e implementação de políticas voltadas para a saúde cardiovascular das mulheres. Esse grupo deverá exercer um papel de liderança nas políticas brasileiras para a saúde, fornecendo aos gestores uma visão geral da relevância das DCV no sexo feminino, para que se possam traçar ações estratégicas para reduzir a prevalência de fatores de risco, melhorar o diagnóstico e a abordagem terapêutica, diminuindo assim a mortalidade e a morbidade das DCV ( Figura 10.1 ). ^4^
