# Supplementary material for: Posicionamento sobre a Saúde Cardiovascular nas Mulheres – 2022
Source: Arq Bras Cardiol. 2022 Nov 7;119(5):815–82. [Article in Portuguese] doi: 10.36660/abc.20220734 (PMC10473826; doi:10.36660/abc.20220734)
Supplement: Translation

# Translation: Position Statement on Women’s Cardiovascular Health – 2022

**Authors:** Glaucia Maria Moraes de Oliveira, Maria Cristina Costa de Almeida, Celi Marques-Santos, Maria Elizabeth Navegantes Caetano Costa, Regina Coeli Marques de Carvalho, Cláudia Maria Vilas Freire, Lucelia Batista Neves Cunha Magalhães, Ludhmila Abrahão Hajjar, Maria Alayde Mendonça Rivera, Marildes Luiza de Castro, Walkiria Samuel Avila, Alexandre Jorge Gomes de Lucena, Andréa Araujo Brandão, Ariane Vieira Scarlatelli Macedo, Carla Janice Baister Lantieri, Carisi Anne Polanczyk, Carlos Japhet da Matta Albuquerque, Daniel Born, Eduardo Belisário Falcheto, Érika Olivier Vilela Bragança, Fabiana Goulart Marcondes Braga, Fernanda M. Consolim Colombo, Ieda Biscegli Jatene, Isabela Bispo Santos da Silva Costa, Ivan Romero Rivera, Jaqueline Ribeiro Scholz, José Xavier de Melo, Magaly Arrais dos Santos, Maria Cristina de Oliveira Izar, Maria Fátima Azevedo, Maria Sanali Moura, Milena dos Santos Barros Campos, Olga Ferreira de Souza, Orlando Otávio de Medeiros, Sheyla Cristina Tonheiro Ferro da Silva, Stéphanie Itala Rizk, Thais de Carvalho Vieira Rodrigues, Thaís Rocha Salim, Viviana de Mello Guzzo Lemke, Elizabeth Regina Giunco Alexandre

**Affiliations:** 1 Brasil Universidade Federal do Rio de Janeiro, Rio de Janeiro RJ – Brasil; 2 Brasil Centro Universitário de Belo Horizonte, Belo Horizonte MG – Brasil; 3 Brasil Universidade Tiradentes (UNIT), Aracaju SE – Brasil; 4 Brasil Centro Universitário do Estado Pará (CESUPA), Belém PA – Brasil; 5 Brasil Secretaria de Saúde do Estado do Ceará, Fortaleza CE – Brasil; 6 Brasil Hospital das Clínicas UFMG, Belo Horizonte MG – Brasil; 7 Brasil Faculdade de Medicina da UFBA, Salvador BA – Brasil; 8 Brasil Instituto do Coração (Incor) do Hospital das Clínicas FMUSP, São Paulo SP – Brasil; 9 Brasil Hospital Universitário Professor Alberto Antunes/Universidade Federal de Alagoas, Maceió AL – Brasil; 10 Brasil Faculdade IPEMED de Ciências Médicas, Belo Horizonte MG – Brasil; 11 Brasil Hospital Agamenom Magalhães, Recife PE – Brasil; 12 Brasil Universidade do Estado do Rio de Janeiro, Rio de Janeiro RJ – Brasil; 13 Brasil Santa Casa de Misericórdia de São Paulo, São Paulo SP – Brasil; 14 Brasil Universidade Municipal de São Caetano do Sul, Caetano do Sul SP – Brasil; 15 Brasil Hospital de Clínicas da UFRS, Porto Alegre RS – Brasil; 16 Brasil Instituto de Medicina Integral Professor Fernando Figueira (IMIP), Recife PE – Brasil; 17 Brasil Escola Paulista de Medicina, São Paulo SP – Brasil; 18 Brasil Hospital Felício Rocho, Belo Horizonte MG – Brasil; 19 Brasil Hospital Pio XII, São José dos Campos SP – Brasil; 20 Brasil Hospital do Coração (HCor), São Paulo SP – Brasil; 21 Brasil UDI Hospital, Rede D’Or, São Luís MA – Brasil; 22 Brasil Universidade Federal de São Paulo (UNIFESP), São Paulo SP – Brasil; 23 Brasil Ministério da Saúde, Brasília DF – Brasil; 24 Brasil Hospital Federal do Rio Grande do Norte, Natal RN – Brasil; 25 Brasil Hospital Universitário de Sergipe, Aracajú, SE – Brasil; 26 Brasil Rede D’Or Hospitais, Rio de Janeiro RJ – Brasil; 27 Brasil CEMISE, Aracajú SE – Brasil; 28 Brasil Hospital São Lucas D’Or, Aracaju SE – Brasil; 29 Brasil Cardiocare Clínica Cardiológica, Curitiba PR – Brasil

**Table:** 

Position Statement on Women’s Cardiovascular Health – 2022	

The report below lists declarations of interest as reported to the SBC by the experts during the period of the development of these statement, 2022.	

Expert	Type of relationship with industry
Alexandra Oliveira de Mesquita	Nothing to be declared
Alexandre Jorge Gomes de Lucena	Nothing to be declared
Andréa Araujo Brandão	Financial declaration A - Economically relevant payments of any kind made to (i) you, (ii) your spouse/partner or any other person living with you, (iii) any legal person in which any of these is either a direct or indirect controlling owner, business partner, shareholder or participant; any payments received for lectures, lessons, training instruction, compensation, fees paid for participation in advisory boards, investigative boards or other committees, etc. From the brazilian or international pharmaceutical, orthosis, prosthesis, equipment and implants industry: - Servier: Acertil, Acertalix, Acertanlo, Triplixan. Other relationships Funding of continuing medical education activities, including travel, accommodation and registration in conferences and courses, from the brazilian or international pharmaceutical, orthosis, prosthesis, equipment and implants industry: - Servier: Acertil, Acertalix, Acertanlo, Triplixan.
Ariane Vieira Scarlatelli Macedo	Financial declaration A - Economically relevant payments of any kind made to (i) you, (ii) your spouse/partner or any other person living with you, (iii) any legal person in which any of these is either a direct or indirect controlling owner, business partner, shareholder or participant; any payments received for lectures, lessons, training instruction, compensation, fees paid for participation in advisory boards, investigative boards or other committees, etc. From the brazilian or international pharmaceutical, orthosis, prosthesis, equipment and implants industry: - Bayer: Anticoagulação e insuficiência cardíaca; Pfizer: Anticoagulação e amiloidose; Jannsen: Leucemia. Other relationships Funding of continuing medical education activities, including travel, accommodation and registration in conferences and courses, from the brazilian or international pharmaceutical, orthosis, prosthesis, equipment and implants industry: - Bayer: Insuficiência cardíaca.
Carisi Anne Polanczyk	Nothing to be declared
Carla Janice Baister Lantieri	Nothing to be declared
Carlos Japhet da Matta Albuquerque	Nothing to be declared
Celi Marques Santos	Nothing to be declared
Cláudia Maria Vilas Freire	Nothing to be declared
Daniel Born	Nothing to be declared
Eduardo Belisario Falchetto	Nothing to be declared
Elizabeth Regina Giunco Alexandre	Financial declaration A - Economically relevant payments of any kind made to (i) you, (ii) your spouse/partner or any other person living with you, (iii) any legal person in which any of these is either a direct or indirect controlling owner, business partner, shareholder or participant; any payments received for lectures, lessons, training instruction, compensation, fees paid for participation in advisory boards, investigative boards or other committees, etc. From the brazilian or international pharmaceutical, orthosis, prosthesis, equipment and implants industry: - Lilly: Trulicity, Jardiance, Glyxambi. Other relationships Funding of continuing medical education activities, including travel, accommodation and registration in conferences and courses, from the brazilian or international pharmaceutical, orthosis, prosthesis, equipment and implants industry: - Novo Nordisk: Ozempic.
Érika Olivier Vilela Bragança	Financial declaration A - Economically relevant payments of any kind made to (i) you, (ii) your spouse/partner or any other person living with you, (iii) any legal person in which any of these is either a direct or indirect controlling owner, business partner, shareholder or participant; any payments received for lectures, lessons, training instruction, compensation, fees paid for participation in advisory boards, investigative boards or other committees, etc. From the brazilian or international pharmaceutical, orthosis, prosthesis, equipment and implants industry: - Bayer: Xarelto; Pfizer: Eliquis; Biocath: dispositivos cardíacos eletrônicos implantáveis e arritmias cardíacas. Other relationships Funding of continuing medical education activities, including travel, accommodation and registration in conferences and courses, from the brazilian or international pharmaceutical, orthosis, prosthesis, equipment and implants industry: - Bayer: Xarelto; Pfizer: Eliquis; Daiichi Sankyo: Lixiana; Boehringer Ingelheim: Pradaxa. Any economically relevant equity interest in companies in the healthcare or education industry or in any companies competing with or supplying to SBC: - Área de Saúde.
Fabiana Goulart Marcondes Braga	Nothing to be declared
Fernanda M. Consolim Colombo	Other relationships Funding of continuing medical education activities, including travel, accommodation and registration in conferences and courses, from the brazilian or international pharmaceutical, orthosis, prosthesis, equipment and implants industry: - Daiichi; Ache; Servier; AstraZeneca; Merck: Anti-hipertensivos.
Gláucia Maria Moraes de Oliveira	Nothing to be declared
Ieda Biscegli Jatene	Financial declaration A - Economically relevant payments of any kind made to (i) you, (ii) your spouse/partner or any other person living with you, (iii) any legal person in which any of these is either a direct or indirect controlling owner, business partner, shareholder or participant; any payments received for lectures, lessons, training instruction, compensation, fees paid for participation in advisory boards, investigative boards or other committees, etc. From the brazilian or international pharmaceutical, orthosis, prosthesis, equipment and implants industry: - AstraZeneca: Palivizumabe. Other relationships Funding of continuing medical education activities, including travel, accommodation and registration in conferences and courses, from the brazilian or international pharmaceutical, orthosis, prosthesis, equipment and implants industry: - AstraZeneca: Palivizumabe.
Isabela Bispo Santos da Silva Costa	Nothing to be declared
Ivan Romero Rivera	Nothing to be declared
Jaqueline Ribeiro Scholz	Nothing to be declared
José Xavier de Melo Filho	Nothing to be declared
Lucelia Batista Neves Cunha Magalhães	Nothing to be declared
Ludhmila Abrahão Hajjar	Nothing to be declared
Magaly Arrais dos Santos	Financial declaration A - Economically relevant payments of any kind made to (i) you, (ii) your spouse/partner or any other person living with you, (iii) any legal person in which any of these is either a direct or indirect controlling owner, business partner, shareholder or participant; any payments received for lectures, lessons, training instruction, compensation, fees paid for participation in advisory boards, investigative boards or other committees, etc. From the brazilian or international pharmaceutical, orthosis, prosthesis, equipment and implants industry: - Edwards: Implante transcateter valvar; Boston: Implante transcateter valvar; Medtronic: Implante.
Marcia de Melo Barbosa	Nothing to be declared
Maria Alayde Mendonça Rivera	Nothing to be declared
Maria Cristina Costa de Almeida	Nothing to be declared
Maria Cristina de Oliveira Izar	Financial declaration A - Economically relevant payments of any kind made to (i) you, (ii) your spouse/partner or any other person living with you, (iii) any legal person in which any of these is either a direct or indirect controlling owner, business partner, shareholder or participant; any payments received for lectures, lessons, training instruction, compensation, fees paid for participation in advisory boards, investigative boards or other committees, etc. From the brazilian or international pharmaceutical, orthosis, prosthesis, equipment and implants industry: - Bayer/Xarelto; Daiichi Sankyo/Lixiana; Libbs/Propafenona e Amiodarona; Pfizer/Eliquis.
Maria Elizabeth Navegantes Caetano Costa	Financial declaration A - Economically relevant payments of any kind made to (i) you, (ii) your spouse/partner or any other person living with you, (iii) any legal person in which any of these is either a direct or indirect controlling owner, business partner, shareholder or participant; any payments received for lectures, lessons, training instruction, compensation, fees paid for participation in advisory boards, investigative boards or other committees, etc. From the brazilian or international pharmaceutical, orthosis, prosthesis, equipment and implants industry: - Libbs: Plenance Enze; Servier: Vastarel. Other relationships Funding of continuing medical education activities, including travel, accommodation and registration in conferences and courses, from the brazilian or international pharmaceutical, orthosis, prosthesis, equipment and implants industry: - Libbs; Servier: participação em congresso.
Maria Fátima de Azevedo	Other relationships Funding of continuing medical education activities, including travel, accommodation and registration in conferences and courses, from the brazilian or international pharmaceutical, orthosis, prosthesis, equipment and implants industry: - Novo Nordisk: Diabetes; Boehinger: Diabetes; Biolab: Hipertensão e anticoagulante. Employment relationship with the brazilian or international pharmaceutical, orthosis, prosthesis, equipment and implants industry, as well as any employment relationship with health insurance companies or medical audit companies (including part-time jobs) in the year to which your declaration refers: - Unimed Natal.
Maria Sanali Moura de Oliveira Paiva	Nothing to be declared
Marildes Luiza de Castro	Financial declaration A - Economically relevant payments of any kind made to (i) you, (ii) your spouse/partner or any other person living with you, (iii) any legal person in which any of these is either a direct or indirect controlling owner, business partner, shareholder or participant; any payments received for lectures, lessons, training instruction, compensation, fees paid for participation in advisory boards, investigative boards or other committees, etc. From the brazilian or international pharmaceutical, orthosis, prosthesis, equipment and implants industry: - AstraZeneca: Forxiga/Insuficiência cardíaca; Servier: Acertil/Hipertensão arterial.
Milena dos Santos Barros Campos	Nothing to be declared
Olga Ferreira de Souza	Nothing to be declared
Orlando Otávio de Medeiros	Nothing to be declared
Regina Coeli Marques de Carvalho	Nothing to be declared
Sheyla Cristina Tonheiro Ferro da Silva	Financial declaration A - Economically relevant payments of any kind made to (i) you, (ii) your spouse/partner or any other person living with you, (iii) any legal person in which any of these is either a direct or indirect controlling owner, business partner, shareholder or participant; any payments received for lectures, lessons, training instruction, compensation, fees paid for participation in advisory boards, investigative boards or other committees, etc. From the brazilian or international pharmaceutical, orthosis, prosthesis, equipment and implants industry: - Boehringer Ingelheim, Novartis Farmacêutica, AstraZeneca, Servier, Libbs. Other relationships Funding of continuing medical education activities, including travel, accommodation and registration in conferences and courses, from the brazilian or international pharmaceutical, orthosis, prosthesis, equipment and implants industry: - Boehringer Ingelheim, Novartis Farmacêutica, Novo Nordisk, AstraZeneca.
Stéphanie Itala Rizk|	Nothing to be declared
Thais de Carvalho Vieira Rodrigues	Other relationships Funding of continuing medical education activities, including travel, accommodation and registration in conferences and courses, from the brazilian or international pharmaceutical, orthosis, prosthesis, equipment and implants industry: - AstraZeneca.
Thaís Rocha Salim	Nothing to be declared
Viviana de Mello Guzzo Lemke	Nothing to be declared
Walkiria Samuel Avila	Nothing to be declared

**Table:** 

List of Abbreviations and Acronyms	
ACS	Acute Coronary Syndrome
AD	Alzheimer’s Disease
AF	Atrial Fibrillation
AMI	Acute Myocardial Infarction
ARNI	Angiotensin-Receptor Neprilysin Inhibitor
ASA	Acetylsalicylic Acid
BMI	Body Mass Index
BP	Blood Pressure
CABG	Coronary Artery Bypass Grafting
CAD	Coronary Artery Disease
CC	Congenital Cardiopathy
CCaS	Coronary Calcium Score
CKD	Chronic Kidney Disease
CMRI	Cardiac Magnetic Resonance Imaging
CSD	Cardiac Sudden Death
CT	Cardiac Transplantation
CTA	Computed Tomography Angiography
CTX	Chemotherapy
CV	Cardiovascular
CVD	Cardiovascular Disease
CVP	Coronary Vasospasm
CVR	Cardiovascular Risk
CVRF	Cardiovascular Risk Factor
DALYs	Disability-Adjusted Life Years (1 DALY represents the loss of 1 year of healthy life)
DCS	Diseases of the Circulatory System
DM	Diabetes mellitus
DOACs	Direct Oral Anticoagulants
DPP-4	Dipeptidyl Peptidase-4
ECG	Electrocardiogram
ET	Exercise Test
GBD	Global Burden of Disease
GD	Gestational Diabetes
GH	Gestational Hypertension
GLS	Global Longitudinal Strain
HF	Heart Failure
HFpEF	Heart failure with preserved ejection fraction
HFrEF	Heart failure with reduced ejection fraction
ICD	Implantable Cardioverter Defibrillator
IHD	Ischemic Heart Disease
IMR	Index of Microcirculatory Resistance
IMT	Intimal-Media Thickness
INOCA	Ischemia with non-obstructive coronary arteries
LDL-c	Low-density-lipoprotein cholesterol
LVEF	Left Ventricular Ejection Fraction
LVH	Left Ventricular Hypertrophy
MCS	Malformations of the Circulatory System
MHT	Menopausal Hormone Therapy
MINOCA	Myocardial infarction with non-obstructive coronary arteries
MVD	Microvascular Disease
NSTEMI	Non-ST Elevation Myocardial Infarction
NT-proBNP	N-terminal fragment of pro-B-type natriuretic peptide
OECD	Organization for Economic Cooperation and Development
PAD	Peripheral Arterial Disease
PE	Preeclampsia
PH	Pulmonary Hypertension
PHC	Primary Health Care
PNS	National Health Survey (in Portuguese, *Pesquisa Nacional de Saúde* )
POS	Polycystic Ovary Syndrome
QoL	Quality of Life
REF	Risk-Enhancing Factor
RF	Risk Factor
RHD	Rheumatic Heart Disease
SAH	Systemic Arterial Hypertension
SBC	Brazilian Society of Cardiology (in Portuguese, *Sociedade Brasileira de Cardiologia* )
SCAD	Spontaneous Coronary Artery Dissection
SDH	Social Determinants of Health
SGLT2	Sodium-Glucose Cotransporter-2
STEMI	ST-Elevation Myocardial Infarction
UI	Uncertainty Interval
WHO	World Health Organization

Legends for the recommendation and level of evidence tables:

**Table:** 

Recommendations:	
	**I –** Conclusive evidence, or, failing that, general consensus that the procedure is safe and useful/effective.
	**II –** Conflicting evidence and/or divergence of opinion about the procedure’s safety and usefulness/effectiveness.
	**IIA –** Evidence/opinion is in favor of the procedure. Most approve of it.
	**IIB –** The procedure’s safety and utility/effectiveness is less well established, opinion is not predominantly in favor of it.
	**III –** Evidence and/or consensus that the procedure is not useful/effective and, in some cases, is harmful.
**Evidence:**	
**Level A**	Data obtained from multiple large randomized studies, concordant and/or robust meta-analysis of randomized clinical studies.
**Level B**	Data obtained from less robust meta-analysis, from a single randomized study or from non-randomized (observational) studies.
**Level C**	Data obtained from expert consensus.

## Contents

Introduction 822

1. Highlights 823

2. Epidemiology of Women’s Cardiovascular Diseases 827

2.1. Diseases of the Circulatory System in Children and Adolescents of the Female Sex in Brazil 835

3. Cardiovascular Risk Factors 837

3.1. Introduction 837

4. Cardiovascular Diseases in Women 844

4.1. Ischemic Heart Disease 844

**4.1.1. Coronary Artery Disease** 844

**4.1.2. Ischemia with Non-obstructive Coronary Arteries** 844

**4.1.3. Myocardial Infarction with Non-obstructive Coronary Arteries** 844

**4.1.4. Spontaneous Coronary Artery Dissection** 846

**4.1.5. Microvascular Disease** 846

**4.1.6. Coronary Vasospasm** 846

**4.1.7. Coronary Thrombosis/Embolism** 846

4.2. Heart Failure 846

**4.2.1. Pharmacological and Nonpharmacological Treatment of HFrEF and HFpEF** 847

4.3. Arrhythmias 849

**4.3.1. Ventricular Tachycardia and Cardiac Sudden Death** 850

**4.3.2. Atrial Fibrillation** 851

4.4. Cardiovascular Disease and Cancer 851

4.5. Stroke 854

4.6. Peripheral Artery Disease 855

4.7. Dementia 855

4.8. Valvular Heart Disease 856

**4.8.1. Aortic Stenosis** 856

**4.8.2. Mitral Valve Disease** 857

**4.8.3. Rheumatic Disease** 857

4.9. Diabetes Mellitus, Preeclampsia, and Pregnancy Hypertensive Disorders 857

**4.9.1. Diabetes Mellitus** 857

**4.9.2. Pregnancy Hypertensive Disorders** 858

4.10. Adolescent Pregnancy 859

5. Cardiac Investigative Methods: Peculiarities in Women 862

5.1. Electrocardiogram 862

5.2. Exercise Test 863

5.3. Carotid Ultrasound 863

5.4. Echocardiography 864

5.5. Myocardial Perfusion Scintigraphy 864

5.6. Coronary Calcium Score and Coronary Computed Tomography Angiography 864

5.7. Cardiac Magnetic Resonance Imaging 864

5.8. Coronary Angiography 866

6. Women’s Representation in Clinical Studies on Cardiovascular Disease and Risk Factors 867

7. Primary Prevention Measures in Women 868

8. Women’s Burnout, Quality of Life and Spirituality 869

8.1. Burnout 869

8.2. Quality of Life 870

8.3. Spirituality 870

9. Cardiovascular Implications of COVID-19

in Pregnancy 871

10. Future Perspectives on Women’s Cardiovascular

Healthcare 873

References 874

## Introduction

Of the noncommunicable chronic diseases, CVDs are the leading cause of death around the world and in Brazil, which has one of the highest mortality rates in South America.^1^The CVDs account for one third of the deaths from all causes, affect men and women at all ages, and represent more than twice the number of deaths from all neoplasms associated.^1^Among women, there is an increase in the prevalence of CVD and death from CVD after menopause, aggravating the perspectives in the near future due to aging and illness of the female population in Brazil.

Currently, IHD accounts for most deaths in all Brazilian Federative Units, followed by cerebrovascular diseases.^1^One aspect is the difference in the involvement of the regions and in the access to diagnosis and treatment, according to the peculiarities determined by the social and economic indicators in the macroregions, states, and cities of different sizes in Brazil. Half of the mortality from CVD before the age of 65 years can be attributed to poverty and social inequalities.^2^Inappropriate diets, low level of physical activity, and alcohol and tobacco consumption are important RFs for CVD in women, more prevalent among the less favored social classes of the population, including Brazilian children and adolescents.^3^Thus, primary and secondary prevention programs, as well as easier access to diagnosis, for those populations might have an even greater impact on CVD morbidity and mortality.

Most of the times, CVD can be prevented with public health interventions that involve RF control and optimized clinical management of patients. Reducing women’s CVDs in Brazil and worldwide is a complex task, which depends on several agents and continuous effort.

Most Brazilian cardiologists, one third of which are women, are members of the SBC, which has been developing continuous actions to reduce CVD morbidity and mortality through the Department of Women’s Cardiology.

The *Arquivos Brasileiros de Cardiologia* , the official scientific publication of the SBC, has published the “Women’s Letter”,^4^which represented an advance in establishing concrete actions to reduce CVD morbidity and mortality in women, such as: to work collectively towards the defense of the global goals to prevent and control noncommunicable chronic diseases, mainly Brazilian women’s CVDs; to establish campaigns of cardiovascular prevention, promoting consistent efforts to reach the goal of 30% reduction in mortality rate by 2030; to elaborate and suggest governmental policies to promote favorable environments to reduce exposure to risk, facilitating the adoption of healthy habits at school, work and leisure environments to fight women’s CVD; to work alongside governments in the development and application of a cardiovascular prevention program, in addition to the incorporation of cost-effective technologies for the reduction of CVD morbidity and mortality in women; engage with communication media to deliver continuous information on the importance of CVDs in women, their major RFs and preventive measures, ensuring the importance of early diagnosis; to provide the highest level of continuous medical education; to promote technical-scientific, cultural, and social exchange among cardiologists in Brazil and around the world; and to foster scientific knowledge necessary to increase the participation of women in the sciences and scientific events in the areas of health and related sciences.

Even considering the huge scientific and technological advances already achieved in cardiology, we need to change the health and disease paradigm, aiming at a population approach that enables the extension of the benefit of those achievements to the entire population. Therefore, a large agreement is required involving the civil society, the societies of medical specialties, the government, and the community to refurbish the education of physicians and other health professionals in parallel with the promotion of a wide discussion in society, contributing to raise awareness and improve the prevention, diagnosis, and treatment of the diseases accounting for most deaths of men and women in Brazil.

Brazil, by establishing in the Federal Constitution the inalienable commitment to the preservation of human dignity, defined health as a social right, ensuring universal, total, and free access to health for all Brazilians.

Even with the constitutional guarantees, inequities regarding women persist in the Brazilian society. Thus, it is necessary, through affirmative actions, to emphasize the need to ensure the essential equality between men and women, particularly regarding the awareness of women’s CVDs, which are neglected in Brazil.^2^

Thus, the “National Day of Awareness of Women’s Cardiovascular Diseases” has been proposed by the authors of the “Women’s Letter” and approved by the Law 14.320 of 2022, sanctioned by the President of Brazil and published in the *Diário Oficial da União* of April, the 1st, originated from the bill 1.136/2019, from the federal representative and cardiologist Mariana Carvalho. That day will be celebrated on May 14th, in honor of the birth of the physician Bettina Ferro de Souza, the first female president of the SBC.

It is fundamental to promote initiatives to enhance knowledge on the importance of cardiovascular health throughout women’s lives. In addition, it is essential to better understand the local differences in women’s cardiovascular health to define public policies and health care, reduce the gap, and promote sex equity regarding Brazilian health care.

Therefore, the SBC’s Department of Women’s Cardiology presents its **Position Statement on Women’s Cardiovascular Health** focused on primary prevention. This document was aimed to help reach the objective stated in the “Women’s Letter” of playing a leadership role in Brazilian health policies, providing managers with an overview of the relevance of women’s CVDs. This view will enable them to elaborate strategies to reduce the prevalence of RFs and improve the diagnosis and therapeutic approach, thus reducing cardiovascular outcomes, with impact on the Brazilian women’s physical, mental, and spiritual health.

## 1. Highlights

In this chapter 1, the highlights of chapters 2 to 10 are presented.

### Epidemiology of the cardiovascular diseases in women

- In the past 30 years, there was an increase in the CVD prevalence among individuals of both sexes aged 15-49 years. In addition, the CVD prevalence was higher in women until 2011; since then, the CVD prevalence in men has been higher.^1^
- The CVDs, mainly IHD and cerebrovascular disease, are the major cause of death in adults of both sexes. The percent of deaths due to IHD was similar in women and men, while the percent of deaths due to stroke was higher in women than in men.^5^
- In the other age groups, from birth to the beginning of adulthood, cardiac malformations, cardiac complications from rheumatic fever, and cardiomyopathies play a key role in mortality of both sexes.^6^
- Of the traditional RFs, overweight, obesity and diabetes mellitus were the most frequent among women. It is worth emphasizing that the prevalence of self-reported SAH in Brazil was higher in the female than in the male sex.

### Cardiovascular risk factors in women

- Recognizing women’s RFs, either specific, most prevalent, or similar to those of men, as well as knowing the 10-year risk for atherosclerotic CVD, is of great importance for CVD risk stratification in the female sex.^2^
- In women, the most impacting traditional RFs for CVD include diabetes mellitus, SAH, dyslipidemia, tobacco use, obesity, and sedentary lifestyle.
- The prevalence of those traditional RFs has increased even among young women and, when associated with sex-specific RFs, contribute to increase morbidity and mortality; however, they are not usually considered in CVR stratification.^7 , 8^
- Sex-specific RFs, such as polycystic ovary syndrome, use of hormonal contraception, gestational hypertension, adverse events of pregnancy, MHT, risks related to inflammatory and autoimmune diseases (rheumatoid arthritis, systemic lupus erythematosus), and depressive disorders are considered REFs.^8^
- The assessment of CVR in women should consider, in addition to risk stratifiers, the aggravating factors of the diabetes mellitus-associated risks, the REFs, and the sex-specific RFs to establish changes in lifestyle and recommend measures for primary prevention of CVD, aimed at identifying and treating earlier a larger number of women at risk.^7 , 8^

### Cardiovascular diseases in women

#### 1 – Ischemic heart disease

- Currently, additional mechanisms of coronary ischemia are well defined, and it is more appropriate to refer to it as IHD because it comprises several coronary affections that cause ischemia, such as myocardial infarction with non-obstructive coronary arteries, ischemia with non-obstructive coronary arteries, spontaneous coronary artery dissection, microvascular disease, coronary vasospasm, and coronary embolism/thrombosis. In women, several pathophysiological mechanisms of IHD are related to one or more of those coronary affections, which can be present even in younger and pregnant women.

#### 2 – Heart failure

- Considering HF pathophysiology, because of hormonal responses, women, as compared to men, differ regarding the HF epidemiology, clinical presentation, outcomes, and treatment.^9 , 10^Regarding HF phenotypes, some are more prevalent in women, such as Takotsubo syndrome, and others are specific to women, such as peripartum cardiomyopathy. However, in both chronic and acute HF, clinical trials/registries targeting women are scarce and the evidence derives from subanalysis of large studies in which women are underrepresented. Multicenter prospective registries could provide more precise evidence about the female population, including a larger number of women in HF with both reduced and preserved ejection fraction.

#### 3 – Arrhythmias

- Women more often have sinus tachycardia and nodal reentry tachycardia, and pregnancy increases the risk for supraventricular tachycardia.^11^
- In long QT syndrome type 2 among women, there is a greater risk of sudden death and pro-arrhythmia. In the puerperium, long QT increases the risk for *torsades de pointes.* ^12^
- In women, sudden death of cardiac etiology more often results from non-ischemic causes, and men are more often resuscitated and treated with a defibrillator. The use of an implantable cardioverter defibrillator for both primary and secondary prevention is more common in men; however, the risk of complications related to the procedure is higher in women.^13^
- Although the incidence of AF is higher among men, older women more often have AF, more symptoms, and worse quality of life. Strong risk predictors of AF in women are SAH and obesity. Women more often have paroxysmal AF, strokes and thromboembolism, HF, higher CHA_2_DS_2_-VASc score, more hospitalizations, and a higher risk of cardiovascular and all-cause mortality.^14 , 15^

#### 4 – Cardiovascular disease and cancer

- Cardiotoxicity is a challenge in the treatment of female cancer, especially breast cancer, which is one of the most frequent neoplasms, and can lead to an increased incidence of cardiovascular events and of cardiovascular and all-cause mortality as compared to women without neoplasm. The risks vary according to the cancer treatment, which can harm the cardiovascular system leading to the following: ventricular dysfunction, from asymptomatic and reversible to symptomatic and irreversible HF; acute coronary syndrome; pericarditis; myocarditis; ventricular arrhythmias; SAH; peripheral vascular disease. Genetic variants are factors related to the risk of cardiomyopathy, explaining why some patients with the same profile develop cardiotoxicity and others do not.
- The prognosis of such patients is affected by some issues, such as the analysis of cardiovascular RFs before, during, and after the diagnosis and oncological treatment, the effective intervention on modifiable RFs, in addition to the early diagnosis and treatment of the chemotherapy-related cardiotoxicity.

#### 5 – Stroke, peripheral artery disease, and dementia

- Future research on the recognition of female sex-specific RFs for stroke and a score to identify the group at higher risk are urgently needed to elaborate stroke prevention strategies for women.
- Peripheral artery disease correlates with IHD, and the treatment of traditional RFs is a preventive measure in both sexes. The recognition of women-specific RFs, such as pregnancy and its complications, or of predominantly female RFs may allow proper risk stratification and the adoption of early preventive measures.
- Women, as compared to men, seem to be at higher risk for developing dementia and memory disorders related to age. Although some studies have shown that estrogen plays a significant role in women’s cognitive functioning, the MHT showed no beneficial effect on reducing those changes even in younger women.^16^
- In patients under the age of 55 years, regardless of sex, the major causes of cognitive changes and dementia are Alzheimer’s disease, vascular dementia, frontoparietal dementia, and alcohol-related dementia.
- There are vascular RFs related to early dementia, such as stroke, transient ischemic attack, kidney disease, CVD, SAH, chronic alcoholism, and drug intoxication. The control of cardiovascular RFs plays a key role in preventing dementia and cognitive diseases, mainly in younger women.

#### 6 – Valvular heart diseases

- The follow-up of women with aortic valve stenosis or mitral valve disease requires careful and continuous reasoning about the best treatment model. The decisions regarding clinical or interventional management depend on the disease’s anatomical and functional diagnosis and careful patient’s assessment.
- When compared to men and considering the same degree of valve calcification, women have a tendency towards higher severity of aortic stenosis because of fibrosis of the valve apparatus, which is more pronounced than calcification. Men and women differ regarding clinical presentation, response to treatment, and results after valvular intervention.^17^
- In transcatheter aortic valve replacement, women are older and have better left ventricular function and lower prevalence of coronary artery disease; however, they have associated comorbidities, such as diabetes mellitus and AF. Anatomical characteristics of the female sex, such as shorter distance between the coronary artery ostia and the valvular ring, as well as the higher prevalence of valvular and aortic calcification, account for higher coronary artery obstruction during the procedure. In addition, the smaller diameter of the peripheral vessels causes greater vascular complications and bleeding.^18^
- Of the preventive measures in valvular heart diseases, the following stand out: primary and secondary prevention of rheumatic disease and antibiotic prophylaxis of infective endocarditis, mainly during childbirth. Valvular heart disease in women have peculiar characteristics with significant impact on treatment results and disease prognosis.
- The indications of infective endocarditis prophylaxis during pregnancy are women with rheumatic valvular heart disease, valvular prostheses, cyanogenic congenital heart disease, and history of infective endocarditis.

#### 7 – Diabetes mellitus, preeclampsia, and pregnancy hypertensive disorders

- Gestational diabetes is associated with maternal and fetal complications, such as PE, premature birth, and perinatal death. Lifestyle changes, such as regular physical activity during pregnancy, in the absence of obstetric or cardiovascular contraindications, substantially reduce the risks of those complications.^19^
- There is a wealth of literature supporting gestational hypertension as a RF for future CVD; primigravida who develop GH are at higher risk for future CVD, notably after menopause. Thus, the best recommendation is to initiate the prevention of PE and other forms of GH prior to pregnancy, which include proper body weight, healthy diet, and regular practice of physical exercises. In women at high risk, the use of acetylsalicylic acid in the first trimester and calcium replacement for pregnant women with low daily calcium intake should be indicated.^20^

#### 8 – Adolescent pregnancy

- Adolescent pregnancy increases maternal, fetal, and neonatal complications, worsens preexisting socioeconomic problems, and affects the future of generations. Thus, it should be managed efficiently and continuously in all socioeconomic levels.
- One of the most important preventive factors of adolescent pregnancy is education on sexuality and reproductive health, supported by scientific evidence and health promotion programs.^21^It should include information on biological aspects, mutual respect, responsible sexual activities, and use of safe and effective contraceptive methods to prevent pregnancy and protect against sexually transmissible infections.^22^
- Adolescents instructed by providers determined to face sensitive subjects, such as pregnancy prevention and contraception, are more engaged in their well-being. The qualified instruction about adolescent sexuality is an investment for a healthier and profitable future with high self-esteem.

## Cardiac investigative methods: peculiarities in women

- Women’s electrocardiographic findings differ from men’s regarding the magnitude of the electric signals, with smaller amplitude of the QRS complex, of the J point, and of the T wave, and a longer corrected QT interval. Anterior T-wave inversion, that is, beyond V1, occurs in 2.3% of the population, being more common in women, regardless of their physical activity *status* . That is a benign pattern if it occurs in asymptomatic and low-risk women, because CMRI studies suggest it might reflect lateral displacement of the right ventricle.^23^Electrocardiographic changes that may lead to misinterpretations are observed in 45% of women with breast implants: T-wave inversion, inferolateral ST depression, poor R-wave progression from V1 to V4, prolonged QT interval, and left ventricular hypertrophy.^24^
- On ET, women more often show false-positive depression of the ST segment and the test’s accuracy varies with the pretest probability of IHD. In women, the association of stress/rest myocardial perfusion scintigraphy with ET has better diagnostic accuracy than ET alone. That association has excellent negative predictive value in women with pretest intermediate/high risk.^25^
- The measure of intimal-media thickness to reclassify risk can be used in women with at least two cardiovascular RFs.^3 , 4^The presence of plaque as an aggravating factor for risk stratification can be used in women at intermediate risk.^26^
- Women with coronary artery calcium score over zero and breast arterial calcification have higher risk of ischemic events as compared to men. Coronary computed tomography angiography evidences the non-obstructive coronary pattern, more prevalent in women.^26 , 27^
- Normal coronary arteries on coronary angiography are more common in women. The risk of vascular complications from coronary angiography is higher among women, who have a higher tendency to develop acute kidney lesion due to contrast media.^28^
- The CMRI is an excellent investigative option for women, especially those of childbearing age, pregnant women, and those undergoing treatment for breast cancer. In addition, the CMRI can evidence perfusion and/or myocardial changes, helping the differential diagnosis of chest pain and being particularly useful in the management of IHD in women.

## Women’s representation in clinical studies on cardiovascular disease and risk factors

- The major cause of death worldwide is CVD, which is more and more recognized as having sex-specific characteristics regarding the illness processes, clinical manifestations, and results of treatments. Therefore, the identification of sex differences in CVD expression determines that women should be equally represented in cardiovascular clinical trials.
- Although women’s representation has increased in recent clinical studies, this has not occurred in all areas of CVD investigation. Women remain underrepresented in studies on arrhythmias, acute and chronic coronary artery disease, and HF, especially those involving interventions with implantable devices and highly complex procedures.
- The identification of barriers to equity in the representation of clinical trial subjects offers several opportunities to solve the problem, enabling equity in selection and maintenance of women as subjects of clinical studies on CVDs and their RFs.
- That equity is necessary for the accurate understanding of CVD expression and their RFs in women, as well as of the impact of treatment on women’s cardiovascular prognosis.

## Primary prevention measures for women

- Primary health care is usually the first contact of women with the health sector, occurring electively or through active search by community health agents. Primary health care should cover the needs of all women’s life stages, with actions directed to total health promotion, with emphasis on physical, mental, and spiritual well-being.
- Over time, there was a decentralization of public services initiated with the Women’s Total Health Care Program and consolidated with the Women’s Health Care Policy. The guarantee of women’s access to all levels of health care and the integration of actions and services remain a challenge, as well as the local planning and monitoring of indicators to promote harm reduction and death prevention.
- A paradigm change is necessary in the public policies on women’s total health, especially when considering that CVDs are the major cause of death among women in most phases of their life cycle.

## Women’s *burnout* , quality of life, and spirituality

- Burnout associates positively with alcohol consumption, sleep disorders, depression, sedentary lifestyle, obesity, and musculoskeletal pain, being a significant predictor of hypercholesterolemia and diabetes type 2, thus relating to a higher incidence of IHD and hospitalizations from CVD.^29^
- Work conditions have a well-known impact on the health of workers. Women’s labor force participation has been increasing, resulting in women’s overload due to double journeys, with consequent high burnout rates among them.
- Women’s life course is permeated by experiences of loss, stress, anxiety, and fear, which increase their psychological vulnerability, facilitating the appearance of symptoms of anxiety-depression. However, resilience, spirituality and personal beliefs seem to play a mediating role in some of those psychological variables, associating with better quality of life and lower frequency of CVD in women.

## Cardiovascular implications of COVID-19 in pregnancy

- The severity of SARS-CoV-2 infection is higher in pregnant as compared to non-pregnant women, with more frequent intensive care unit admissions, mechanical ventilation use, and increased mortality and morbidity, including myocardial infarction, venous and other thromboembolic events, PE, and preterm labor and delivery.^30^In addition, COVID-19 has been associated with a higher rate (and combined proportions) of premature delivery, PE, cesarean delivery, and perinatal death.^31^
- he management of cardiac complications during pregnancy should comprise the multidisciplinary follow-up with a cardiologist, an obstetrician, and a neonatologist.
•SARS-CoV-2 infection during pregnancy should be included in the differential diagnosis with other complications, such as coronary artery dissection and peripartum cardiomyopathy.^32^
- The beginning and duration of prophylactic anticoagulation in pregnancy associated with SARS-CoV-2 infection should consider disease severity, need for hospitalization, temporal relation between disease occurrence and time of delivery, as well as the prothrombotic risk conferred by additional comorbidities.^31^
- In this population, vaccination should be emphasized, because it is the best way to prevent COVID-19-related complications. Preventive measures, such as face mask use, hand hygiene, and avoiding crowded areas, should be maintained.

## Future perspectives on the improvement of women’s cardiovascular care

- Women’s mortality rates from CVD remain elevated and stagnant in most regions of the world, with little or no reduction in recent years. Women’s CVR is still underestimated by the general population, and, especially, by women themselves and health care professionals. The non-implementation of the guidelines on women’s CVD prevention delays the diagnosis of several CVDs, especially that of IHD, which is often neglected in women.^33^
- Understanding the sex/gender-related differences in premature mortality from CVDs is essential for the development of preventive and control measures for those diseases. The scarcity of robust clinical studies and the female sex underrepresentation in clinical trials contribute to the scarce knowledge on women’s CVD.^34^Further research on the role played by sexual hormones in the female global CVR is mandatory.
- To reduce the CVD load in women by 2030 is an ambitious, but imperative, goal, especially because several cardiovascular RFs can be modified and mitigated.

## 2. Epidemiology of Women’s Cardiovascular Diseases

According to data from the GBD 2019 Study, the prevalence of CVD was 6.1% of the population in 2019, 12 946 932 (95% UI, 11 899 752 – 13 617 524) individuals, 51% of the male sex. Men had a higher age-standardized prevalence rate as compared to women in 2019 ( Figure 2.1 ). From 1990 to 2019, there was an 8.7% reduction in the CVD prevalence rate in men, smaller than that in women, 12.8%. There was an increase in the CVD prevalence in that period among individuals of both sexes aged 15-49 years. In addition, the CVD prevalence was higher in women until 2011; since then, the CVD prevalence in men has been higher ( Table 2.1 and Figure 2.2 ).^1 , 5^

**Figure 2.1:** Age-standardized prevalence rates of cardiovascular disease, per 100 000 inhabitants, according to sex, in Brazil, 1990-2019.

Source: Global Burden of Disease Study (GBD) 2019.^1^

**Table 2.1:** Number of cases and age-standardized prevalence, incidence, death, and DALYs rates due to cardiovascular disease in women, per 100 000 inhabitants, and percent change of the rates, according to age group, in Brazil, in 1990 and 2019.

Age groups	1990		2019		Percent change (95% UI)

	Number (95% UI)	Rate (95% UI)	Number (95% UI)	Rate (95% UI)	
**PREVALENCE**					
15-49 years	81840.1 (71524.9;92783.6)	210.2 (183.7;238.3)	105700 (92430.3;120336.2)	180.7 (158.1;205.8)	-14 (-16.8;-10.9)
50-69 years	102496.1 (91526.7;114514.7)	1255.7 (1121.3;1402.9)	208399.3 (186607.1;232143.7)	973.2 (871.4;1084)	-22.5 (-25;-19.8)
5-14 years	26514.1 (17557.2;37666.8)	151.7 (100.4;215.5)	24476.1 (16014.6;34617.6)	154.6 (101.1;218.6)	1.9 (-1.4;5.7)
70+ years	77895.9 (69702.8;86822.5)	3321.7 (2972.3;3702.4)	200343.1 (180509.1;222408)	2653.3 (2390.6;2945.5)	-20.1 (-22.9;-17.2)
Age-standardized	294962.9 (275518.3;317426.8)	557.5 (523.8;597.3)	544515.2 (512491.4;581529.1)	437.4 (411;468.6)	-21.5 (-23.3;-20)
All ages	294962.9 (275518.3;317426.8)	391.9 (366;421.7)	544515.2 (512491.4;581529.1)	491.1 (462.3;524.5)	25.3 (21.7;29.1)
Under 5 years	6216.7 (4434;8521.4)	74.5 (53.1;102.1)	5596.8 (3974;7644)	73.8 (52.4;100.8)	-1 (-4;2.2)
**INCIDENCE**					
15-49 years	81840.1 (71524.9;92783.6)	210.2 (183.7;238.3)	105700 (92430.3;120336.2)	180.7 (158.1;205.8)	-14 (-16.8;-10.9)
50-69 years	102496.1 (91526.7;114514.7)	1255.7 (1121.3;1402.9)	208399.3 (186607.1;232143.7)	973.2 (871.4;1084)	-22.5 (-25;-19.8)
5-14 years	26514.1 (17557.2;37666.8)	151.7 (100.4;215.5)	24476.1 (16014.6;34617.6)	154.6 (101.1;218.6)	1.9 (-1.4;5.7)
70+ years	77895.9 (69702.8;86822.5)	3321.7 (2972.3;3702.4)	200343.1 (180509.1;222408)	2653.3 (2390.6;2945.5)	-20.1 (-22.9;-17.2)
Age-standardized	294962.9 (275518.3;317426.8)	557.5 (523.8;597.3)	544515.2 (512491.4;581529.1)	437.4 (411;468.6)	-21.5 (-23.3;-20)
All ages	294962.9 (275518.3;317426.8)	391.9 (366;421.7)	544515.2 (512491.4;581529.1)	491.1 (462.3;524.5)	25.3 (21.7;29.1)
Under 5 years	6216.7 (4434;8521.4)	74.5 (53.1;102.1)	5596.8 (3974;7644)	73.8 (52.4;100.8)	-1 (-4;2.2)
**DEATHS**					
15-49 years	769476.9 (739810.4;801490.1)	1976.3 (1900.1;2058.5)	679263.9 (631272.6;728617.3)	1161.5 (1079.5;1245.9)	-41.2 (-44.5;-37.8)
50-69 years	1154563.6 (1117881.8;1194713.4)	14144.8 (13695.4;14636.7)	1485239.2 (1407973.4;1568445)	6935.6 (6574.8;7324.2)	-51 (-53.4;-48.4)
5-14 years	38226.4 (34169.5;42955.7)	218.7 (195.5;245.7)	22398 (18744.9;26627.2)	141.4 (118.4;168.2)	-35.3 (-42.9;-28.7)
70+ years	976778.1 (898018.1;1019807.9)	41652.6 (38294.1;43487.5)	1661643.2 (1472104.5;1785160.2)	22006.3 (19496.1;23642.2)	-47.2 (-50.2;-44.7)
Age-standardized	3017512.3 (2897630.7;3117428)	6191.2 (5895.3;6408.3)	3875201.6 (3604407.9;4099252.4)	3019.5 (2810.8;3195.6)	-51.2 (-53.4;-49.1)
All ages	3017512.3 (2897630.7;3117428)	4008.9 (3849.7;4141.7)	3875201.6 (3604407.9;4099252.4)	3495.4 (3251.1;3697.5)	-12.8 (-17;-8.6)
Under 5 years	78467.3 (65203.6;97481.4)	940.3 (781.4;1168.2)	26657.3 (21657.1;32912.1)	351.4 (285.5;433.9)	-62.6 (-73.6;-49.5)
**DALYs**					
15-49 years	769476.9 (739810.4;801490.1)	1976.3 (1900.1;2058.5)	679263.9 (631272.6;728617.3)	1161.5 (1079.5;1245.9)	-41.2 (-44.5;-37.8)
50-69 years	1154563.6 (1117881.8;1194713.4)	14144.8 (13695.4;14636.7)	1485239.2 (1407973.4;1568445)	6935.6 (6574.8;7324.2)	-51 (-53.4;-48.4)
5-14 years	38226.4 (34169.5;42955.7)	218.7 (195.5;245.7)	22398 (18744.9;26627.2)	141.4 (118.4;168.2)	-35.3 (-42.9;-28.7)
70+ years	976778.1 (898018.1;1019807.9)	41652.6 (38294.1;43487.5)	1661643.2 (1472104.5;1785160.2)	22006.3 (19496.1;23642.2)	-47.2 (-50.2;-44.7)
Age-standardized	3017512.3 (2897630.7;3117428)	6191.2 (5895.3;6408.3)	3875201.6 (3604407.9;4099252.4)	3019.5 (2810.8;3195.6)	-51.2 (-53.4;-49.1)
All ages	3017512.3 (2897630.7;3117428)	4008.9 (3849.7;4141.7)	3875201.6 (3604407.9;4099252.4)	3495.4 (3251.1;3697.5)	-12.8 (-17;-8.6)
Under 5 years	78467.3 (65203.6;97481.4)	940.3 (781.4;1168.2)	26657.3 (21657.1;32912.1)	351.4 (285.5;433.9)	-62.6 (-73.6;-49.5)

Source: Global Burden of Disease Study (GBD) 2019. Rate/100 000 inhabitants.^1^UI: uncertainty interval.

**Figure 2.2:** Prevalence of cardiovascular disease, according to sex, in Brazil, 1990-2019.

Source: Global Burden of Disease Study (GBD) 2019.^1^

In 2019, in Brazil, the age-standardized IHD incidence rates, mainly of myocardial infarction, were 78 and 148 per 100 000 inhabitants for women and men, respectively. Regarding chronic IHD (previous myocardial infarction, stable angina, or ischemic HF), the age-standardized prevalence rates were 1046 and 2534 per 100 000 inhabitants for women and men, respectively ( Table 2.1 ).

Analysis of data from the GBD 2019 study showed a reduction in the age-standardized CVD mortality rate for women. In the beginning of the period, 1990, the mortality rates for women differed significantly between the Brazilian geographic regions, and that difference decreased by the end of the period. This can be explained by a more pronounced reduction in the Southeast and South, regions that concentrate the largest populations and incomes, and more modest in the North and Northeast ( Figures 2.3 and 2.4 ).^1 , 5^

**Figure 2.3:** Proportional mortality due to cardiovascular disease, according to sex, in Brazil, 1990-2019.

Source: Global Burden of Disease Study (GBD) 2019.^1^

**Figure 2.4:** Age-standardized mortality rates due to cardiovascular disease, per 100 000 inhabitants, in women, in Brazil and its regions, 1990-2019.

Source: Global Burden of Disease Study (GBD) 2019.^1^

In Brazil, CVDs are the major cause of death among women and men, despite their 50.6% decrease from 1990 to 2019 ( Table 2.1 ). Although the age-standardized CVD mortality rates were higher in men during the entire period, the percent reduction was similar for both sexes, 48% for men and 52% for women. The proportional CVD mortality was higher in women during the entire period (1990 to 2019) ( Figure 2.3 ).^1 , 5^

According to GBD 2019 study estimates, of the CVDs, IHD was the first cause of death in Brazil, followed by stroke. The IHD accounted for 12.03% and 12.2% of the deaths of women and men, respectively, while the percent of deaths due to stroke was higher in women than in men, 10.39% and 8.41%, respectively. The estimated DALYs due to IHD were 1276.6 (1165.2;1359) and 2179.4 (2054.4;2296.3) and those due to stroke were 1235.6 (1133.8;1322.5) and 1410.1 (1323.5;1487.9) in women and men, respectively ( Figures 2.5 and 2.6 ).^1 , 5^

**Figure 2.6:** DALYs (%) due to cardiovascular disease in women (A) and men (B), according to age group, in Brazil, 2019.

Source: Global Burden of Disease Study (GBD) 2019.^1^

In 2019, the prevalence of AF and atrial flutter was higher in men than in women, although the age-standardized mortality and DALYs rates were higher in women. AF occurs in association with advanced RHD, especially mitral stenosis, more frequent in women, at the ratio of 3 to 2.

The age-standardized prevalence of RHD showed a slight 2.1% increase, being higher among women (3.5%). However, there was a reduction in the age-standardized mortality rates attributable to RHD, the percent reduction being similar in both sexes in the past 30 years ( Figures 2.5 and 2.6 ). In addition, the proportional mortality from aortic stenosis was higher among women in Brazil in 2019, and the DALYs rates decreased from 1990 to 2019 similarly for men and women ( Figures 2.5 and 2.6 ).^1 , 5^

**Figure 2.5:** Proportional mortality from cardiovascular disease in women (A) and men (B), according to age group, in Brazil, 2019.

Source: Global Burden of Disease Study (GBD) 2019.^1^

According to data from the Brazilian Ministry of Health Mortality Information System, from 1980 to 2018, HF was the underlying cause of death of 1 185 120 individuals (584.155 men [49.3%]).^35^The ratios between the crude mortality rates from HF in both sexes, according to age groups and geographic regions, are shown in Table 2.2 . Ratios equal to or higher than 1 were observed in almost the entire period, all age groups, and geographic regions, with higher mortality in men, except for the North (1985-1989), Northeast (1980-1984) and South regions in the age group of 60 years and over, in which women’s mortality was higher.^35 , 36^

**Table 2.2:** Ratio between the crude mortality rates in the male and female sexes, according to age groups and geographic regions, in 5-year periods.

Age group	Number Region / Period	1980-1984	1985-1989	1990-1994	1995-1999	2000-2004	2005-2009	2010-2014	2015-2018
**0-29**	North	1.0	0.9	1.2	1.0	1.2	1.3	1.3	1.8
	Northeast	0.9	1.0	1.0	1.1	1.1	1.4	1.4	1.5
	Southeast	1.1	1.2	1.3	1.3	1.2	1.6	1.5	1.8
	South	1.1	1.2	1.2	1.6	1.5	1.3	1.5	1.1
	West-Central	1.1	1.1	1.1	1.1	1.9	1.5	2.7	1.0
**30-59**	North	1.5	1.4	1.5	1.4	1.6	1.9	1.8	1.5
	Northeast	1.2	1.3	1.4	1.3	1.3	1.4	1.5	1.6
	Southeast	1.4	1.5	1.6	1.5	1.6	1.7	1.6	1.5
	South	1.4	1.5	1.5	1.4	1.4	1.5	1.4	1.2
	West-Central	1.2	1.5	1.6	1.6	1.8	1.9	1.7	1.7
**60+**	North	1.1	1.1	1.1	1.1	1.2	1.3	1.3	1.2
	Northeast	1.2	1.2	1.2	1.1	1.2	1.2	1.2	1.2
	Southeast	1.1	1.1	1.0	1.0	1.0	1.0	1.0	1.0
	South	1.1	1.0	1.0	0.9	0.9	0.9	0.9	0.9
	West-Central	1.1	1.1	1.0	1.0	1.1	1.1	1.1	1.2

Source: Brazilian Health Information System - Datasus.^35,36^

Of the RFs for CVDs in Brazilian women, the following stand out: SAH, dietary risks, obesity, increased serum cholesterol levels, and elevated fasting glycemia ( Figure 2.7 ).^7^From 1990 to 2019, elevated BMI was the RF that increased most in Brazil.^1 , 7^The specific RF in women with stroke include pregnancy, preeclampsia, gestational diabetes, oral contraception use, hormone use during menopause, and changes in the hormone status.^7^

**Figure 2.7:** Ranking of the age-standardized mortality and DALYs rates due to cardiovascular diseases attributable to risk factors, in 2019, in Brazil, women (A) and men (B). DALY: disability-adjusted life years; CVD: cardiovascular disease; GBD: Global Burden of Disease; LDL: low-density lipoprotein. Source: Global Burden of Disease Study (GBD) 2019. 1,5

The prevalence of self-reported SAH in Brazil in 2019 was 23.9%, higher in the female than in the male sex (26.4% *versus* 21.1%, respectively).^37^Cardiovascular mortality attributable to SAH was higher in women aged 65-79 years and in men aged 50-79 years.^5^Dietary risks were the second most important RF for CVD in 2019, accounting for 5.0% and 5.7% of the deaths due to IHD and for 2.6% and 2.4% of the deaths due to stroke in women and men, respectively.^2^Physical inactivity, another behavioral RF, increased from 1990 to 2019 in Brazil, mainly among women, 4.7%, as compared to men, 3.1%.^2^According to data from the Brazilian Institute of Geography and Statistics (IBGE), in 2019, in Brazil, the percentages of overweight and obese adults (age ≥18 years) were 62.6% and 29.5% for women, and 57.5% and 21.8% for men, respectively. There was a progressive increase of obesity with age increase, with a higher prevalence of overweight and obesity in women of all age groups.^5^The prevalence of diabetes increases as the prevalence of obesity increases.^5^Data from the 2014-2015 PNS in Brazil showed a higher prevalence of diabetes in women, individuals over the age of 30 years, and those with overweight or obesity.^37^Diabetes is a RF for CAD more important to women than to men, even to premenopausal women.^7 , 37^

Anxiety, depression, and violence victimization were studied in 31 847 women from the 2013 PNS. The major depressive episodes and suicidal ideation were assessed with the *Patient Health Questionnaire,* while violence victimization was self-reported. Women had a higher prevalence of depressive episodes (OR = 2.36; 95% CI, 2.03-2.74), suicidal ideation (OR = 2.02; 95% CI, 1.73-2.36), and violence victimization (OR = 1.73; 95% CI, 1.45-2.06).^38^

Sex-specific factors are of fundamental importance and affect the occurrence of CVD throughout a woman’s life. A cross-sectional multicenter study with 27 referral maternity hospitals from all Brazilian regions and 82 388 women giving birth, from July 2009 to June 2010, identified 9555 cases of severe maternal morbidity, 140 deaths, and 770 maternal near misses. The major determinant cause of maternal complications was hypertensive disease.^39^Preeclampsia, gestational diabetes, pregnancy-induced hypertension, preterm delivery, and small for gestational age newborn are early indicators of maternal cardiovascular risk. According to data from the GBD 2019 study, hypertensive diseases of pregnancy were the second cause of mortality and DALYs in women of childbearing age ( Figure 2.8 ).^1^

**Figure 2.8:** Mortality and DALYs rates due to maternal causes in the federative units, per 100 000 inhabitants, in women, Brazil, 2019.

Source: Global Burden of Disease Study (GBD) 2019.^1^

### 2.1. Diseases of the Circulatory System in Children and Adolescents of the Female Sex in Brazil

The etiologies of the DCS in individuals under the age of 20 years in Brazil vary according to age group, sex, and place of residence. The MCS are the major cause of death up to the age of 4 years, and cardiomyopathies are the major cause for ages 5 to 19 years.^40^

In Brazil, the deaths due to MCS from 2000 to 2015 were more frequent under the age of 4 years, mainly in the first year of life and in the male sex ( Table 2.3 ).^6^In the female sex, the following MCS had the highest incidence: persistent truncus arteriosus, Ebstein’s anomaly (tricuspid valve), and ostium secundum atrial septal defect. The DCS more often occurred at the age of 5-19 years, predominantly in the female sex, probably because of competition of external causes of death, more prevalent in the male sex.^6^

**Table 2.3:** Proportional mortality and mortality rate per group of causes of death in children, according to sex and age group, Brazil, 2000-2015. 6

Causes of death		<20 years	Male						Female					

		Total	Total	<1year	1-4 years	5-9 years	10-14 years	15-19 years	Total	<1year	1-4 years	5-9 years	10-14 years	15-19 years
**MCS**	Deaths	57.892	31.077	26.144	2.901	766	625	641	26.815	22.016	2.972	728	593	506
	PM(%)	4.2	3.7	6.2	4.1	1.8	1.1	0.3	5.15	6.6	5.0	2.4	1.7	0.8
	Mort	5.3	5.62	107.0_(1)_	2.7_(2)_	0.6	0.4	0.5	5.0	94.7_(1)_	2.9_(2)_	0.5	0.4	0.4
**Other CM**	Deaths	86.165	45.237	39.715	3.133	956	725	708	40.928	35.729	3.009	870	720	600
	PM(%)	6.3	5.4	9.3	4.4	2.3	1.3	0.3	7.78	10.8	5.1	2.9	2.0	0.9
	Mort	7.9	8.2	162.6_(1)_	2.9_(2)_	0.7	0.5	0.5	7.7	153.7_(1)_	2.9_(2)_	0.7	0.5	0.4
**DCS**	Deaths	29.904	16.706	3.735	2.084	1.493	2.749	6.645	13.198	3.280	2.045	1.376	2.197	4.300
	PM(%)	2.2	2.0	0.9	2.9	3.6	4.8	2.6	2.54	1.0	3.4	4.5	6.2	6.6
	Mort	2.8	3.0	15.3_(1)_	1.9_(2)_	1.1	2.0	4.7	2.5	14.1_(1)_	2.0_(2)_	1.0	1.6	3.1
**Ill defined**	Deaths	85.458	49.940	25.907	7.692	3.019	3.679	9.643	35.518	19.414	6.431	2.408	2.700	4.565
	PM(%)	6.2	5.9	6.1	10.9	7.2	6.5	3.8	6.82	5.9	10.8	7.9	7.7	7.0
	Mort	7.9	9.0	106.0_(1)_	7.0_(2)_	2.2	2.6	6.8	6.7	83.5_(1)_	6.2_(2)_	1.8	2.0	3.3
**External**	Deaths	340.974	274.627	10.816	16.304	16.384	29.287	201.836	66.347	7.431	10.328	8.801	11.992	27.795
	PM(%)	24.9	32.5	2.5	23.0	39.1	51.6	80.4	12.75	2.2	17.4	28.8	34.0	42.5
	Mort	31.4	49.7	44.3_(1)_	15.0_(2)_	11.9	20.9	142.5	12.5	32.0_(1)_	9.9_(2)_	6.6	8.9	20.2
**All causes**	Deaths	1.367.355	845.481	424.932	70.854	41.904	56.775	251.016	521.874	331.269	59.430	30.518	35.293	65.364
	PM(%)	100.0	100.0	100.0	100.0	100.0	100.0	100.0	100.0	100.0	100.0	100.0	100.0	100.0
	Mort	126.0	153.0	1.739.3_(1)_	65.2_(2)_	30.4	40.5	177.3	98.0	1424.7_(1)_	57.0_(2)_	23.0	26.1	47.6

MCS: malformations of the circulatory system; Other CM: other congenital malformations excluding MCS; DCS: diseases of the circulatory system; PM(%): proportional mortality in percentage; Mort: mortality rate per 100 000 (1) Mortality per 100 000 live births (2) Mortality per 100 000 in the population aged 0 – 4, live births excluded.

The importance of deaths from DCS increases with age progression ( Figure 2.9 ).^6 , 40^One should consider that children with MCS, even repaired ones, and who did not die in their first year of life can have complications and sequelae, such as HF, arrhythmias, and endocarditis, with death in adolescence. Another possible explanation is the higher diagnostic negligence of MCS and DCS in the female sex at all age groups in Brazil, resulting from the poorer access to the health system and diagnostic resources in the female sex as compared to that in the male sex.^3 , 6 , 40^

**Figure 2.9:** Annual proportional mortality due to specific causes of the circulatory system in children and adolescents, per sex and age group, in the state of Rio de Janeiro, 1995 to 2012. 40 DCS: Diseases of the Circulatory System; CBVD - Cerebrovascular Diseases.

Table 2.4 shows the proportional mortality attributed to DCS and MCS in the first year of life due to specific causes and according to Brazilian regions.^13^More than 83% of the deaths due to DCS resulted from MCS in all regions, with emphasis to the South region, where that percentage was 93%. The non-specified MCS corresponded to half of the deaths under the age of 1 year in Brazil, with no difference between the sexes and predominance in the Southeast region (44%). The proportional mortality from DCS was 2.5 times higher in the North region than in the South region, predominating in the female sex. Cardiomyopathy accounted for 32% of the deaths from DCS, being the leading cause of death in all regions and both sexes.^41^

**Table 2.4:** Proportional mortality due to diseases of the circulatory system and malformations of the circulatory system, subdivided into specific causes, in the first year of life, female sex, according to Brazilian regions, 2000 to 2015. 41

CAUSES OF DEATH		NORTH	NORTHEAST	SOUTHEAST	SOUTH	WEST-CENTRAL	TOTAL
CARDIAC-PULMONARY AND PULMONARY CIRCULATION DISEASES	Deaths	77	131	244	39	41	532
	PM(%)	**3.09**	**1.99**	**2.33**	**1.15**	**1.75**	**2.10**
PERICARDITIS AND ENDOCARDITIS	Deaths	13	32	69	9	14	137
	PM(%)	**0.52**	**0.48**	**0.66**	**0.27**	**0.60**	**0.54**
MYOCARDITIS	Deaths	3	14	49	23	4	93
	PM(%)	**0.12**	**0.21**	**0.47**	**0.68**	**0.17**	**0.37**
CARDIOMYOPATHIES	Deaths	130	360	469	61	71	1091
	PM(%)	**5.22**	**5.46**	**4.47**	**1.80**	**3.02**	**4.31**
HEART FAILURE	Deaths	74	170	140	33	25	442
	PM(%)	**2.97**	**2.58**	**1.33**	**0.98**	**1.06**	**1.75**
CEREBROVASCULAR DISEASES AND OF OTHER VASSELS	Deaths	46	91	187	31	34	389
	PM(%)	**1.84**	**1.38**	**1.78**	**0.92**	**1.45**	**1.64**
OTHER DISEASES OF THE CIRCULATORY SYSTEM	Deaths	83	188	243	37	45	596
	PM(%)	**3.33**	**2.85**	**2.32**	**1.09**	**1.92**	**2.36**
**SUBTOTAL DCS**	Deaths	426	986	1401	233	234	3280
	PM(%)	**17.11**	**14.97**	**13.36**	**6.89**	**9.96**	**12.97**
CHAMBERS AND SEPTA	Deaths	300	715	1943	630	370	3985
	PM(%)	**12.05**	**10.75**	**18.52**	**18.64**	**15.75**	**15.75**
VALVES	Deaths	87	196	691	293	138	1405
	PM(%)	**3.49**	**2.97**	**6.59**	**8.67**	**5.87**	**5.55**
NON-SPECIFIED	Deaths	1284	3749	4666	1733	1324	12756
	PM(%)	**51.57**	**56.90**	**44.48**	**51.27**	**56.36**	**50.43**
OTHERS	Deaths	235	547	712	166	126	1786
	PM(%)	**9.57**	**8.30**	**6.79**	**4.91**	**5.36**	**7.06**
VESSELS	Deaths	158	395	1049	325	157	1727
	PM(%)	**6.34**	**5.99**	**10.00**	**9.62**	**6.68**	**8.24**
SUBTOTAL MCS	Deaths	2064	5602	7859	3147	2115	22016
	PM(%)	**82.89**	**85.04**	**74.93**	**93.11**	**90.04**	**87.03**
**TOTAL DCS+MCS**	Deaths	2490	6588	10489	3380	2349	25296
	PM(%)	**100.00**	**100.00**	**100.00**	**100.00**	**100.00**	**100.00**

DCS: Diseases of the Circulatory System; MCS: Malformations of the Circulatory System; PM(%): Proportional Mortality in percentage.

In the past decade, the increase in obesity, insulin resistance, and SAH in children and adolescents contributed to increase the cardiovascular risk in young individuals. An increase in the prevalence of DCS in that population is estimated to occur in coming years.^26 , 42^

More girls and female adolescents died from DCS than their male counterparts, indicating the need for public health strategies directed to the female sex, such as equity of access to health resources, early diagnosis, and institution of female sex-specific therapeutic measures.

## 3. Cardiovascular Risk Factors

### 3.1. Introduction

According to data from the GBD Study 2019, there was a global reduction in the CVD mortality rate in past decades, tending to stagnation in recent years.^1^In women aged 35-54 years, there was a growing increase in the IHD mortality rate in the United States of America,^8 , 43^attributed to the higher prevalence of traditional CVRFs.

According to data from the GBD Study 2019 and regarding Brazilian women ( Figure 2.7 ), in the ranking of age-standardized mortality and DALYs rates attributable to risk factors, the following risk factors stood out in decreasing order: elevated systolic blood pressure, elevated BMI, dietary risks, high LDL-cholesterol, tobacco use, and high fasting glycemia. It is worth emphasizing the increased CVD prevalence in young women aged 15-49 years, from 1990 to 2019.^7 , 44^

Regarding the risk of atherosclerotic CVD, women share some traditional CVRFs with men, while some other CVRFs are underrecognized in women ( Chart 3.1 and Figure 3.1 )^2^and others are female-sex/gender specific ( Figure 3.2 ).

**Chart 3.1:** Aggravating factors of the cardiovascular risk from diabetes mellitus in women. 50

•Women’s age > 56 years and diabetes diagnosis >10 years
•Family history: first-degree relative with premature CVD (men <55 years and women <65 years)
•Tobacco use
•Systemic arterial hypertension
•Metabolic syndrome
•Albuminuria: creatinine >30 mg/g
•Glomerular filtration rate <60 ml/min
•Non-proliferative retinopathy
•Incipient cardiovascular autonomic neuropathy

**Figure 3.1:** Risk factors for cardiovascular disease in women. The well-established risk factors common to both sexes are incorporated into the risk scores of atherosclerotic cardiovascular disease. However, the sex-specific and the underrecognized risk factors interact with the traditional ones, aggregating risk, especially in women.

**Figure 3.2:** Female sex-specific risk factors for cardiovascular diseases, with evidence and recommendations. 11,45–49 AH: arterial hypertension; BMI: body mass index; BP: blood pressure; CAD: coronary artery disease; CVD: cardiovascular disease; CVR: cardiovascular risk; CVRF: cardiovascular risk factor; DM: diabetes mellitus; IHD: ischemic heart disease; IUD: intrauterine device; MHT: menopausal hormone therapy; MI: myocardial infarction; MS: metabolic syndrome; PE: preeclampsia; POS: polycystic ovary syndrome.

In clinical trials, despite the important relationship between CVRFs and CVD, women are underrepresented, and the scores used for cardiovascular risk stratification, such as Framingham, SCORE ( *Systematic COronary Risk Evaluation* ), SBC/SBD/SBEM, do not contemplate female sex-specific CVRFs, such as ( Figure 3.2 ): POS, hormonal contraception use, GH, adverse events of pregnancy, menopausal hormone therapy, and risks associated with the socioeconomic, psychosocial and environmental *status* . In addition, the scores do not contemplate the association with inflammatory (HIV) and autoimmune (rheumatoid arthritis, systemic lupus erythematosus, psoriasis) diseases,^8^anxious-depressive disorders, and SDH, considered REFs in women.

When assessing cardiovascular risk, it is essential to consider, in addition to risk stratifiers ( Figure 3.3 ), the aggravating risk factors associated with diabetes mellitus ( Chart 3.2 ), the REFs, and the sex-specific RFs to establish changes in lifestyle and recommend measures for primary prevention of CVD, aimed at identifying and treating earlier a larger number of women at risk.^7 , 8 , 44^

**Figure 3.3:** Algorithm for the assessment of women’s cardiovascular risk and the recommendation of statin use. 53 ABI: ankle-brachial index; CAD: coronary artery disease; CCaS: coronary calcium score; CRD: chronic renal disease; CTA: computed tomography angiography; DM: diabetes mellitus; eGFR: estimated glomerular filtration rate; GRS: Global Risk Score.

**Chart 3.2:** Most prevalent risk factors for CVD in women, as well as the cardiovascular risk factors shared by men and women, with evidence. 11,43,45,53,55–58,65–67

Risk factor	> risk for CVD in women	Evidence
**Diabetes mellitus**	**X**	SAH and inadequate diet are associated with high risk of pre-diabetes. Women with DM have a 45% higher risk of IHD.^45,67^ DM2 is more common in women < 40 years and results in higher mortality. Relative risk of CVD mortality is higher in women than in men; the excessive risk of CVD mortality is higher in women.^45,67^ Atherosclerotic events cause more congestive HF in women.^67^
**Systemic arterial hypertension**	**X**	Higher risk of myocardial infarction (INTERHEART).^67^ Higher prevalence of pre-menopausal secondary hypertension. Post-menopausal women less often have nocturnal blood pressure decrease, but more events. Women more often develop ventricular hypertrophy, HFpEF, arterial stiffness, CRD, and DM. Women more often have adverse effects with anti-hypertensive drugs. SAH affects 80% of women > 75 years. Only 29% have proper control of SAH.^45^
Dyslipidemia (*)		In the SWAN study, the levels of TC, LDL-c, and triglycerides showed a peak in the transition from menopause to the early post-menopausal phase.^65^ Recent meta-analysis showed no significant difference in HDL-c levels between pre- and post-menopause.^61^
Obesity (*)		The Framingham Heart Study showed an increase in the risk of CAD: 64% in women *versus* 46% in men.^45^ More prevalent in Brazilian women than men. The most important RF for SAH in women. Associated with adverse outcomes in pregnancy.
Sedentary lifestyle (*)		Higher prevalence of inactivity among women. 25% of women do not practice physical activity regularly.^43,45^
Tobacco use (*)		Women have a 25% higher risk of CAD as compared to men, except in the younger age group (30-44 years).^43,67^
**Atrial fibrillation**	**X**	As compared to men with AF, women have a 20-30% higher risk of stroke, higher severity, and worse long-term result regarding permanent disability. The female sex was associated with age >65 years or >2 risk factors in the CHA2DS2-VASc regarding nonvalvular AF.^67^
Metabolic syndrome (*)		In the SWAN study, the risks of MS in pre-menopausal and menopausal women are 1.45x and 1.25x higher, respectively.^65^
**Rheumatoid arthritis**		50% increase in the risk of CVD mortality as compared to that of the general population.^67^
**Systemic lupus erythematosus**		The risk of IHD is three times higher.^45^ More prevalent in Asian, Afro-American, Afro-Caribbean, and Hispanic women as compared to Caucasian women. Black women are 2-4 times more likely to have SLE than white women. IHD is the major cause of mortality in SLE.^67^
Depression (**)	**X**	Common in women with IHD. Independent risk factor for worse outcomes.^56^ Women are more susceptible, with a 2-time increase in the risk of IHD.^45^
**Mental stress (anxiety, psychosocial stress) Vital exhaustion**	**X**	Studies have shown the association of mental stress, microvascular dysfunction, and coronary vasospasm in patients with INOCA,^58^mainly women.
**Social Determinants of Health**		SDH have negative outcomes that, added to the classical and sex/gender-specific CVRFs, cause vascular inflammation and endothelial dysfunction, culminating in CVD.^59^

(*) risk factors with attributed risk similar in both sexes. (**) Basic questions: How do you think your health will be in the future? How often do you feel pleasure or happiness? Have you ever been grateful for your health or any other thing in your life? - Analyzes optimism, positive affection, and gratitude, respectively,56 and might help recognition and specific approach. ACEI: angiotensin-converting-enzyme inhibitor; AF: atrial fibrillation; BMI: body mass index; BRA: angiotensin-receptor blocker; CAD: coronary artery disease; CVD: cardiovascular disease; CRD: chronic renal disease; SDH: social determinants of health; RF: risk factor; CVRF: cardiovascular risk factor; DM: diabetes mellitus; DM2: diabetes mellitus type 2; DOAC: direct oral anticoagulants; HDL-c: high-density lipoprotein cholesterol; HF: heart failure; HFpEF: heart failure with preserved ejection fraction; IHD: ischemic heart disease; INOCA: ischemia with non-obstructive coronary arteries; LDL-c: low-density lipoprotein cholesterol; MS: metabolic syndrome; SAH: systemic arterial hypertension; SLE: systemic lupus erythematosus; TC: total cholesterol.

The risk estimation of cardiovascular events in women aimed at primary prevention is limited. The ACC/AHA Guideline on the Primary Prevention of Cardiovascular Disease identifies situations involving apparently healthy women with REFs, which require a shared decision-making with a cardiologist, an obstetrician gynecologist, and the patient about the use of mid-potency statin for low or intermediate risk. There is no evidence about the use of statins, and the assessment of the CCaS is necessary in selected women. If the CCaS is zero, statin should not be initiated, except in the presence of: DM, family history of premature CAD, or tobacco use. A CCaS between 1 and 99 favors the use of statins, mainly in women > 55 years. If CCaS > 100 or above the 75th percentile for age and sex, statins should be introduced ( Figure 3.3 ).^51 , 52^

The 2017 Brazilian Guideline on Dyslipidemias and Atherosclerosis Prevention^53^and the 2019 SBC Update of the Guideline on Cardiovascular Prevention^26^recommend the use of the Global Risk Score,^54^which estimates the 10-year risk of fatal or nonfatal myocardial infarction and stroke, heart failure, and peripheral vascular failure. Figure 3.3 suggests an algorithm for the assessment of women’s cardiovascular risk and the recommendation of statin use.

The anxiety-depression disorders, such as mood, behavior, and affection changes, accompanied or not by somatic changes or cognitive deficit, are common causes of disability in developed countries and considered REFs by the AHA/ACC and the European Society of Cardiology. Women are more susceptible to anxiety-depression disorders, which double the risk of IHD. Behavioral, hormonal, genetic, and psychosocial changes overlap and, from the pathophysiological viewpoint, promote endothelial dysfunction, atherothrombosis, and immune and hemostasis dysfunctions from changes in the hypothalamus-hypophysis-adrenal axis and autonomous nervous system ( Figure 3.1 ). In addition, the estrogens stimulate T and B lymphocytes, propitiating a higher immune and inflammatory response. Such overlaps justify the relationship between depression and CVD, mainly IHD. In this context, physical exercise has been recognized as effective for the treatment of depression, similarly to psychotherapy and the use of serotonin inhibitors. Aerobic exercises seem to promote better benefit.^55 - 59^

Regarding the SDH, the diversity of race and ethnicity, educational level, racism, and discrimination, inaccessibility to health care systems, absence of social support, economic instability, sexual orientation, and violence are factors that lead to disparities, affect women, and generate negative outcomes, which, added to the classic CVRFs and sex/gender-specific risk factors, promote vascular inflammation and endothelial dysfunction, culminating in CVD ( Figure 3.1 ).^26 , 59 - 63^

A cross-sectional study to calculate the cardiovascular risk, using laboratory data from a subsample of the PNS with 3584 women, has found 58.4% at low cardiovascular risk, 32.9% at intermediate risk, and 8.7% at elevated risk. The risk increased with age, being high in the population with low educational level. The proportion of the components of the Framingham model, according to risk groups and sex, shows that, for women at high risk, the major contributors to cardiovascular risk were: systolic blood pressure, total cholesterol, HDL, DM, and tobacco use.^64^

Charts 3.3 , 3.4 , 3.5 , 3.6 , 3.7 and 3.8 show recommendations for the management of dyslipidemia, diabetes, metabolic syndrome, tobacco use, overweight and obesity, arterial hypertension, and physical activity in women.

**Chart 3.3:** Recommendations for the management of dyslipidemia in women.

Recommendations for the management of dyslipidemia in women			

Recommendation	CR	LE	References
Nutritional therapy, body weight loss, and physical activity should be recommended for all patients.		**A**	^26,43,51,67^
Reduce sodium intake and maintain adequate consumption of fresh fruits, vegetables, and low-fat dairy products. Maintain body weight and waist circumference within normal range.		**A**	^26,51,53,67^
At least 30 minutes of moderate dynamic aerobic exercise (walking, running, biking, or swimming) 5 to 7 days per week. Gradually increase aerobic physical activity to 300 minutes per week of moderate intensity or 150 minutes per week of vigorous aerobic physical activity, or an equivalent combination of both, ideally with daily supervised exercise.		**A**	^26,43,51,67^
Women at very high and high cardiovascular risk: LDL-c should be reduced to < 50 mg/dL and < 70 mg/dL, and non-HDL-c to < 80 mg/dL and < 100 mg/dL, respectively.		**B**	^51,52,53,^
Women at intermediate cardiovascular risk: LDL-c should be reduced to < 100 mg/dL, and non-HDL-c to < 130 mg/Dl.		**A**	^51,52,53^
Women at low cardiovascular risk: the goal is LDL-c < 130 mg/dL and non-HDL-c < 160 mg/dL.		**A**	^51,52,53^
Medicamentous treatment is not recommended for pregnant women.		**A**	^11,43,47,67^
Bile acid sequestrants are considered safe for use while breastfeeding.		**B**	^11,43,47,67^
Rosuvastatin and pravastatin at low doses have been studied and their use can be considered while breastfeeding if the benefits exceed the potential risks.		**B**	^11,43,47,67^

CR: class of recommendation; LE: level of evidence; HDL-c: high-density lipoprotein cholesterol; LDL-c: low-density lipoprotein cholesterol. The minimum reassessment time after medicamentous treatment should be of one month. Adapted from Update of the Guideline on Dyslipidemias and Atherosclerosis Prevention.^53^Similarly to the recommendation on the use of medicines during pregnancy, the authors of this article recommend a shared decision-making between patient and clinician to determine the ideal clinical management for individual patients.

**Chart 3.4:** Recommendations for the management of diabetes and metabolic syndrome in women.

Recommendations for the management of diabetes and metabolic syndrome in women			

Recommendation	CR	LE	References
The strategies of body weight control, physical activity, dietary guidance, and smoking cessation should be provided to all women with glucose intolerance, MS, or DM to reduce CV risk.		**A**	^26,43,50,51,52,67^
The risk of coronary events should not be stratified by use of anatomic or functional methods in asymptomatic women with MS or DM.		**A**	^26,43,50,51,52,67^
The CCaS should be used in women with DM or MS and intermediate CV risk (GRS 5-10% in 10 years). If CCaS = 0, statins should not be initiated.		**B**	^26,43,50,51,52,53,67^
In primary prevention, women with DM or MS for whom statin therapy is indicated should receive high-potency doses and/or ezetimibe, and the goal should be LDL-c < 70 mg/dL.		**A**	^26,43,50, 51,52,67^
Alternatively, in women with DM or MS at high or very high risk, the goal should be LDL-c < 50 mg/dL.		**B**	
The use of ASA is not recommended for primary prevention in women with MS or DM, regardless of their CV risk.		**A**	^26,43,50,51,53,67^
The use of ASA is recommended for prevention in women with MS or DM, at high or very high risk, in the absence of contraindication or risk of bleeding.	a	**B**	^26,43,50,51,52,67^
Regarding pharmacological treatment, there is no gender difference in the recommendations, except for those related to pregnancy.		**A**	^11,26,43,50,51,52,67^

CR: class of recommendation; LE: level of evidence; ASA: acetylsalicylic acid; CCaS: coronary calcium score; CV: cardiovascular; DM: diabetes mellitus; GRS: global risk score; LDL-c: low-density-lipoprotein cholesterol; MS: metabolic syndrome.

**Chart 3.5:** Recommendations for the management of tobacco use in women.

Recommendations for the management of tobacco use in women			

Recommendation	CR	LE	References
Women’s tobacco use and exposure should be routinely assessed at all consultations with healthcare professionals and written down in the medical chart.		**A**	^26,43,51,52,67^
Systematic counseling of all women for tobacco use cessation.		**A**	^26,43,51,52,67^
A combination of cognitive-behavioral and pharmacological interventions is recommended to all women to maximize the cessation rates.		**A**	^26,43,51,52,67^
Smoking abstinence is recommended for all women to reduce the cardiovascular risks.		**A**	^26,43,51,52,67^
Multidisciplinary teams should be assigned to facilitate smoking cessation in all healthcare systems.	a	**B**	^26,43,51,52,67^

CR: class of recommendation; LE: level of evidence.

**Chart 3.6:** Recommendations for the management of overweight and obesity in women.

Recommendations for the management of overweight and obesity in women			

Recommendation	CR	LE	References
For overweight and obese women, body weight loss is recommended to improve the cardiovascular risk profile.		**B**	^26,43,51,52,67^
Counselling and comprehensive interventions in lifestyle, including caloric restriction, are recommended to achieve and maintain weight loss in overweight and obese women. Emphasize the relationship between overweight and obesity with increased cardiovascular risk.		**B**	^26,43,51,52,67^
Calculate the body mass index and take anthropometric measures in medical visits to identify overweight and obese women aiming at intervening.		**C**	^26,43,51,52,67^
The treatment should involve a multidisciplinary team, and, when proper weight reduction is not achieved, pharmacological treatment and bariatric surgery should be considered. Medication should be used if BMI > 30 or >27 in the presence of comorbidities.		**B**	^26,43,51,52,67^
Assess waist circumference to identify women at higher cardiometabolic risk.	a	**B**	^26,43,51,52,67^
The use of pharmacological drugs to reduce body weight during pregnancy and while breastfeeding is not recommended. Special attention should be paid to women at childbearing age.		**B**	^26,43,51,52,67^

CR: class of recommendation; LE: level of evidence; BMI: body mass index.

**Chart 3.7:** Recommendations for the management of arterial hypertension in women.

Recommendations for the management of arterial hypertension in women			

Recommendation	CR	LE	References
For all women with high BP or hypertension, nonpharmacological measures are indicated to reduce BP: body weight loss, healthy dietary pattern, sodium reduction, dietary potassium supplementation, increase in physical activity with a structured exercise program, and limited alcohol intake.		**A**	^26,43,51,52,67^
Anti-hypertensive medication is recommended for women with an estimated risk ≥ 5% in 10 years and mean systolic BP ≥ 130 mm Hg or mean diastolic BP ≥ 80 mm Hg, for primary prevention of CVD.		**A**	^26,43,51,52,67^
For women with confirmed hypertension and CV risk ≥ 10%, target BP < 130/80 mm Hg is recommended.		**B**	^26,43,51,52,67^
For women with arterial hypertension and chronic renal disease, target BP < 130/80 mm Hg is recommended.		**B**	^26,43,51,52,67^
For women with arterial hypertension and type 2 diabetes, target BP < 130/80 mm Hg is recommended, and anti-hypertensive medication should be initiated if BP ≥ 130/80 mm Hg.		**B**	^26,43,51,52,67^
For women with estimated risk < 10% in 10 years and BP ≥ 140/90 mm Hg, anti-hypertensive medication is recommended for primary prevention of CVD.		**C**	^26,43,51,52,67^
For women with confirmed hypertension and no additional markers of increased CV risk, target BP < 130/80 mm Hg is recommended.	b	**B**	^26,43,51,52,67^
Use neither ACEI nor ARB for women at childbearing age and with perspective of pregnancy because of their possible teratogenic effects.		**A**	^26,43,51,52,67^
Secondary hypertension is common in young and older women, and hypertensive adolescents and young adults should be screened to prevent long-term CV complications and specific treatment should be initiated		**A**	^26,43,51,52,67^

CR: class of recommendation; LE: level of evidence; ARB: angiotensin-receptor blocker; CV: cardiovascular; CVD: cardiovascular disease; ACEI: angiotensin-converting-enzyme inhibitor; BP: blood pressure.

**Chart 3.8:** Recommendations for the management of physical activity in women.

Recommendations for the management of physical activity in women			

Recommendation	CR	LE	References
During medical visits, physicians should advise their patients to practice physical activity.		**B**	^26,43,51,52,67^
Physical activity: ≥ 150 minutes/week of moderate intensity exercise or 75 minutes/week of vigorous exercise reduce cardiovascular risk.		**A**	^26,43,51,52,67^
Physical activity: < 150 minutes/week of moderate intensity exercise or < 75 minutes/week of vigorous exercise reduce cardiovascular risk.	a	**B**	^26,43,51,52,67^
Physical activity: > 150 minutes/week of moderate intensity exercise throughout pregnancy without complications.		**A**	^26,43,51,52,67^

CR: class of recommendation; LE: level of evidence.

## 4. Cardiovascular Diseases in Women

### 4.1. Ischemic Heart Disease

In women, IHD accounts for 47% of the deaths from CVD, while stroke, to 36%.^1^The IHD pathophysiology, clinical presentation, diagnosis, treatment, and prevention are shown in Table 4.1 .

**Table 4.1:** General characteristics of ischemic heart disease.

	CAD^**70**^	INOCA^**69**^	MINOCA^**71**^	SCAD	Vasospasm^**72**^	Microvascular disease^**70**^	Coronary thrombosis/embolism^**73**^
**Pathophysiology**	- Smaller atherosclerotic plaque - Plaque erosion with distal embolization - Diffuse atherosclerosis associated with microvascular and endothelial dysfunction	Microvascular dysfunction and/or epicardial and microvascular vasospasm	Coronary vasospasm, microvascular dysfunction, spontaneous coronary thrombosis and rechanneled emboli, plaque rupture, and spontaneous coronary artery dissection	- Nontraumatic, non-iatrogenic, and non-atherosclerotic separation of the coronary artery wall, due to spontaneous rupture of the intima or vasa vasorum within the arterial wall - Intramural hematoma within the false lumen, compressing the true lumen, causing ischemia or myocardial infarction - Increased shear stress - Emotional/physical triggers	- Epicardial or microvascular vasoconstriction related to endothelial dysfunction - Multiple mechanisms: autonomous nervous system (circadian variation with morning predominance), endothelial dysfunction, chronic inflammation, oxidative stress, and smooth muscle hypercontractility	- Structural remodeling - Microvascular spam - External compression (myocardial hypertrophy) - Reduced microcirculatory conductance and/or dynamic arteriolar obstruction	Thrombophilia, atrial fibrillation, valvular disease, patent *foramen ovale* , infective endocarditis, and non-bacterial thrombotic endocarditis
**Clinical presentation**	- Older women - Chest pain (precordial/retrosternal) ;jaw/neck pain - Fatigue, nausea - Stable/unstable angina - AMI	Wide spectrum: from clinical manifestations, such as chest discomfort, to angina and anginal equivalents	AMI 2/3 of the cases are NSTEMI (exclude myocarditis, Takotsubo, PPCM and PE) - Rule out: sepsis, PE, non-cardiac causes of troponin elevation	- Wide spectrum: from mild chest pain, ACS, AMI to sudden cardiac death - Most common cause of AMI in pregnancy and puerperium	Wide spectrum: from silent disease to sudden cardiac death Angina/AMI	Angina Dyspnea on exertion	AMI
**Clinical diagnosis / complementary tests**	- Similar in women and men	Non-invasive tests: CMRI associated with adenosine; PET Invasive tests: intracoronary acetylcholine and hyperemia with intracoronary adenosine (CFR and IMR)	- Transient until the cause is established - Clinical findings of AMI - Troponin - Coronary angiography without obstructive CAD, CCT, ICUS - CMRI	- No traditional CVRF - Knowledge about the predisposing RF: fibromuscular dysplasia (50-86%), connective tissue disorders (5%), systemic inflammatory diseases (5-12%), hormone therapy (estrogen, progesterone, gonadotrophin, clomiphene, or infertility treatment) - Previous multiple pregnancies - Coronary CTA - Coronary angiography	- Coronary angiography associated with intracoronary acetylcholine provocation test	- Association with inflammatory markers (systemic lupus erythematosus and rheumatoid arthritis) - Non-invasive tests: reversible defects - Invasive tests: FFR > 0.8; CFR < 2.0; IMR ≥ 25	- Coronary angiography / aspiration thrombectomy
**Prevention**	- Knowledge about the differences in ACS/CCS clinical findings - Improve diagnosis and treatment - Identify and treat classical CVRFs and women-specific RFs	- Identify and aggressively treat classical CVRFs and women-specific RFs - Identify risk enhancing factors (psychosocial factors and social determinants of health)	- Identify and treat classical CVRFs and women-specific RFs - Identify risk enhancing factors (psychosocial factors and social determinants of health)	- Reduce emotional triggers, avoid hormone therapy (estrogen, progesterone, chorionic gonadotropin) - Prevent pregnancy - Cardiac rehabilitation, preferably with modified protocol, avoiding heavy isometric exercises and intense aerobic activities	- Avoid aggravating factors: use of illegal drugs, amphetamines, butane gas, alcohol, and medications for migraines	- Modification of CVRFs (weight loss and stress control)	- Early diagnosis of the underlying causes and thrombosis/infection prophylaxis
**Outcomes**	Woman: higher in-hospital and 30-day mortality - Higher mortality in women under 50 years of age	- Recurring angina - Frequent hospitalizations - Repeated coronary angiographies - High rates of major CV events	- Prognosis depends on the underlying cause - Mortality:similar to that of CAD - Disability: similar to that from CAD	- Frequent recurrence, mainly in the postpartum period and in women with connective tissue diseases	- Recurrence rate from 3.9% to 18.6% - Arrhythmia and sudden death	- Recurring angina - Frequent hospitalizations -Repeated coronary angiographies - High rates of major CV events	- Good prognosis in most cases undergoing early thrombectomy

ACS: acute coronary syndrome; AMI: acute myocardial infarction; CAD: coronary artery disease; CTA: computed tomography angiography; CCS: chronic coronary syndrome; CFR: coronary flow reserve; CMRI: cardiac magnetic resonance imaging; CV: cardiovascular; CVRF: cardiovascular risk factor; FFR: fractional flow reserve; IMR: index of microcirculatory resistance; NSTEMI: non-ST elevation myocardial infarction; PE: pulmonary embolism; PET: PET scan; PPCM: peripartum cardiomyopathy; RF: risk factor; CCT: coronary computed tomography; ICUS: intracoronary ultrasound.

Currently, the term ‘IHD’ is more suitable to refer to the coronary disorders causing ischemia, previously referred to as CAD. Ischemic heart disease includes CAD, INOCA, MINOCA, SCAD, MVD, CVP, and coronary embolism/thrombosis.

#### 4.1.1. Coronary Artery Disease

CAD is the major cause of myocardial infarction in women. Sex/gender-related differences are especially pronounced in CAD and its peculiarities are shown in Table 4.1 .

#### 4.1.2. Ischemia with Non-obstructive Coronary Arteries

INOCA is more common in women than in men, with especially high prevalence among women aged 45-65 years. It is not a benign condition, being associated with an increased risk of adverse cardiovascular events.^69^The strategies for the management of INOCA have not been well defined, mainly because there is not enough evidence on the treatment of the microvascular dysfunction associated with INOCA.^70^

#### 4.1.3. Myocardial Infarction with Non-obstructive Coronary Arteries

MINOCA is related to several pathophysiological mechanisms of coronary disorders ( Table 4.1 ). Its prevalence is 5-10%, considering all AMI, and two-thirds of the patients have NSTEMI.^69^

It is more common in younger women than men (10.5% vs 3.4%; p<0.0001). Cardiovascular RFs can be present, although less prevalent than in patients with CAD.^69^

MINOCA is a transient diagnosis that requires confirmation of its causes. First, AMI due to epicardial coronary obstruction should be excluded. Then, it is paramount to search for the underlying cause; failure in identifying that cause can result in inadequate treatment and information.^70^

#### 4.1.4. Spontaneous Coronary Artery Dissection

SCAD is a rare cause of myocardial infarction, representing 1-4% of all ACS. Corresponds to the spontaneous rupture of the intima or of the *vasa vasorum* within the arterial wall, resulting in accumulation of intramural hematoma in the false lumen, which can compress the true lumen, causing ischemia or myocardial infarction.

SCAD is increasingly recognized as an important cause of myocardial infarction in women under the age of 50 years. According to some studies, 25-35% of the cases of SCAD occur in women under the age of 50 years and 25% in women over the age of 60 years. In addition, it is the most common cause of myocardial infarction associated with pregnancy (up to 43% of the cases), occurring mainly in the third trimester or postpartum period. The risk of recurring events is substantial.^70^

#### 4.1.5. Microvascular Disease

Its pathophysiology results from structural remodeling with consequent conductance reduction or arteriolar vasomotor disorders, or both. The diagnostic confirmation of MVD should meet the following criteria: presence of symptoms (angina and/or dyspnea on exertion); absence of obstructive disease; objective evidence of ischemia; and changes in the microvascular function (reversible defects, abnormalities on the invasive functional tests – IMR > 25).^69^

MVD associates with pro-inflammatory markers in women with INOCA; thus, there is increased risk in diseases such as systemic lupus erythematosus and rheumatoid arthritis. After menopause, these diseases are more frequent in women, which can contribute to the sexual differences of MVD.^69^

#### 4.1.6. Coronary Vasospasm

CVP is defined by reversible diffuse or focal vasoconstriction, common in patients with IHD and present in the mechanisms of INOCA, MINOCA and MVD, regardless of racial, genetic, and geographic variations. It is more prevalent in women aged 40-70 years.^69^

The intracoronary acetylcholine provocation test remains the fundamental tool for the diagnosis of CVP, and women have a higher response to smaller doses. This test allows the reproduction of CVP and the assessment of reactivity to nitrates.

#### 4.1.7. Coronary Thrombosis/Embolism

Coronary artery embolism in an underdiagnosed cause of ACS, and can be classified into three types: direct, paradoxical (thrombus originated from deep venous thrombosis that transposes the *foramen ovale* ), and iatrogenic. In the third type, percutaneous coronary intervention is the most common cause of the embolism, and the risk is increased with rotation techniques, valvuloplasty, and inadequate anticoagulation of the procedure.

## 4.2. Heart Failure

Epidemiological studies have shown similar incidence of HF in men and women. However, hormonal changes, mainly postmenopausal, are responsible for peculiar characteristics of HF in women, which contribute to the higher prevalence of HFpEF (EF≥50%) as compared to HFrEF (FE≤40%).^9 , 74 , 75^ Figure 4.1 shows the pathophysiological mechanisms related to HF in women due to the hormone cycle.^10^

**Figure 4.1:** Pathophysiological mechanisms related to heart failure in women.

LV: left ventricular; HF: heart failure

There are sex/gender-related differences not only regarding HF epidemiology, but also regarding HF clinical presentation, outcomes, and treatment ( Figure 4.2 ).^74 , 75^Those differences are even more evident when the different phenotypes of HF are analyzed in isolation: HFrEF (EF ≤ 40%) and HFpEF (EF ≥ 50%).^9^

**Figure 4.2:** Risk factors, characteristics, prognosis, and measures to prevent HFrEF and HFpEF in women. HF: heart failure; HFpEF: heart failure with preserved ejection fraction; HFrEF: heart failure with reduced ejection fraction; SAH: systemic arterial hypertension; RF: risk factor; CV: cardiovascular.

Two clinical conditions that can evolve to HF in women are worth noting: Takotsubo syndrome, a reversible acute left ventricular dysfunction, in which 90% of the patients are women, especially after menopause;^10^and peripartum cardiomyopathy, defined as left ventricular dysfunction at the end of pregnancy or in the first months after delivery, without any other evident cause.^76^ Table 4.2 shows the major characteristics of those pathologies.

**Table 4.2:** Characteristics of the Takotsubo syndrome and peripartum cardiomyopathy.

Clinical condition	Takotsubo Syndrome	Peripartum Cardiomyopathy
**Women (%)**	90%	100%
**Mean age**	Elderly – frequently postmenopausal (90% aged 58-75 years)	Childbearing age
**Definition**	Reversible acute left ventricular dysfunction	Left ventricular dysfunction at the end of pregnancy or first postpartum months
**Risk factors**	Typical emotional or physical trigger, but not always present More common in postmenopausal women	✓ Afro-American ethnicity ✓ Advanced maternal age ✓ SAH or preeclampsia ✓ Multiple pregnancies
**Characteristics**	✓ Acute chest pain suggestive of ACS ✓ Electrocardiographic changes suggestive of ACS ✓ Dyspneia ✓ Palpitation ✓ Pre-syncope / syncope due to ventricular arrhythmia or ✓ Cardiogenic shock	✓ Dyspnea, orthopnea, lower limb edema, palpitation ✓ Signs of congestion at the end of pregnancy ✓ Thromboembolic events ✓ Ventricular arrhythmias ✓ Cardiogenic shock
**Diagnosis**	✓ ECG: ST elevation or T inversion + prolonged QTc ✓ ECHO: segmental alteration that extends beyond a single artery territory ✓ High natriuretic peptides ✓ Mild troponin elevation (occurs in more than 90%) ✓ Disparity between the degree of troponin elevation and the severity of LV dysfunction ✓ MRI: LV dysfunction; RV assessment; thrombus; no late enhancement (different from myocarditis) ✓ Coronary angiography: absence of coronary lesions with apical and middle-apical hypokinesia on ventriculography	✓ Diagnosis can be delayed due to confusion with usual symptoms of pregnancy ✓ Differential diagnosis with myocarditis, Takotsubo, hypertrophic cardiomyopathy, etc ✓ ECHO: left ventricular dysfunction; presence of thrombus ✓ High natriuretic peptide levels ✓ Holter: might reveal ventricular arrhythmias
**Prevention**	✓ Related to menopause (estrogen deficiency, but hormone replacement does not reduce the risk for HF)	✓ Control risk factors that predispose to PPCM: hypertension, diabetes, obesity, eclampsia
**Treatment**	✓ Beta-blocker if LVEF < 45%, AF, ventricular arrhythmia ✓ ACEI if LVEF < 45% ✓ Anticoagulation if apical thrombus ✓ MCS if cardiogenic shock	BOARD ✓ B: Bromocriptine is indicated (increasing doses according to PPCM severity) ✓ O: Oral treatment for HF according to guidelines ✓ A: Anticoagulation (at least prophylactic) ✓ R: Relaxant (vasodilators) IV and inotropic drugs for severe PPCM ✓ D: Diuretics if congestion For the most severe cases: ✓ IAB, ECMO for cases of rapid deterioration despite inotropic drugs ✓ Transplant or VAD if symptoms persist despite optimal therapy
**Prognosis**	Usually good prognosis with early recovery of LV function, but severe complications can occur before recovery Cardiogenic shock (6-20%) In-hospital mortality (<5%)	Usually good prognosis (40% increase LVEF to > 50%) Persistent dysfunction or rapid deterioration can occur LVEF < 35% at presentation confers worse prognosis
**Follow-up**	Imaging test to confirm LV function recovery: ECHO or MRI Recurrence in up to 22%	Improvement from 6 months to 5 years in general New pregnancy is not advised because of the risk of recurrence

ACS: acute coronary syndrome; AF: atrial fibrillation; ACEI: angiotensin-converting-enzyme inhibitor; CATH: cardiac catheterization; ECG: electrocardiogram; ECHO: echocardiogram; ECMO: extracorporeal membrane oxygenation; HF: heart failure; IAB: intra-aortic balloon; IV: intravenous; LV: left ventricular; LVEF: left ventricular ejection fraction; MCS: mechanical circulatory support; MRI: magnetic resonance imaging; PPCM: peripartum cardiomyopathy; RV: right ventricular; SAH: systemic arterial hypertension; VAD: ventricular assistance device.

Data from acute HF registries have shown that women and men are equally affected (50%), with no evidence of differences in mortality between men and women ( Table 4.3 ),^77^although women have a higher risk profile (older age and higher number of comorbidities). However, in clinical studies/registries of cardiogenic shock/ mechanical circulatory support, women are underrepresented.

**Table 4.3:** Differences in characteristics and outcomes between men and women with decompensated heart failure and/or cardiogenic shock.

CLINICAL STUDY	ADHERE Decompensated HF	IABP-SHOCK II Cardiogenic shock – IAB	cVAD Registry Cardiogenic shock – IMPELLA
**Women (%)**	52%	31%	27%
**Year**	2001-2004	2009-2012	2007-2013
**Characteristics**	Older women (75 vs 70 years)	Older women (74 x 68 years)	Older women (71 x 64 years)
	Higher % of comorbidities: ✓ SAH ✓ Thyroid disease Smaller % of ischemic etiology (19% vs 32%)	Higher % of comorbidities: ✓ SAH (76% vs 66%) ✓ DM (40% vs 29%) Smaller % of ischemic etiology (16% vs 25%)	Higher % of comorbidities: ✓ SAH (80% vs 70%) ✓ DM (54% vs 41%) Smaller % of ischemic etiology (33% vs 35%)
	Higher LVEF(42% vs 33%)	Higher LVEF (40% vs 35%)	Higher LVEF (30% vs 24%)
**Mortality**	No difference in adjusted in-hospital mortality	No difference in adjusted 30-day, 6-month, and 1-year mortality	No difference in mortality on hospital discharge

DM: diabetes mellitus; HF: heart failure; IAB: intra-aortic balloon; LVEF: left ventricular ejection fraction; SAH: systemic arterial hypertension.

The best way to prevent progression to advanced HF is the institution of adequate treatment for HF. However, when not sufficient, the gold-standard treatment is CT for both sexes. The mean long-term survival is significantly higher in the female sex, and sex-mismatch transplant (female donor/male receptor) has worse prognosis.^70^Regarding the use of long-term ventricular assistance devices, the results have improved. One indication for ventricular assistance device use is contraindication to CT, such as hypersensitization, which is frequent in multiparous women. Women tend to have a higher risk for stroke and right ventricular dysfunction, in addition to be underrepresented in most registries.^77^

### 4.2.1. Pharmacological and Nonpharmacological Treatment of HFrEF and HFpEF

Considering that ischemic cardiomyopathy is the most frequent etiology of HFrEF in women, establishing measures to prevent CAD, such as dyslipidemia and hypertension control, tobacco use cessation, and physical activity encouragement, is essential ( Figure 4.2 ). The pharmacological treatment of HFrEF with angiotensin-converting-enzyme inhibitors, beta-blockers, mineralocorticoid antagonists, ARNI, and SGLT2 inhibitors has shown to unequivocally reduce general death or cardiovascular mortality and hospitalization due to HF.^9^Those benefits are similarly for men and women, and, thus, the pharmacological treatment prevents sudden death and disease progression to advanced HF that requires specific therapies. However, women are underrepresented in most studies on HFrEF (20-30%); therefore, studies involving a higher number of women with HFrEF are required ( Table 4.4 ).^10^

**Table 4.4:** Differences in outcomes between men and women in studies on HFrEF.

CLINICAL STUDY	% Women	OUTCOME	GENERAL RESULT	MEN / WOMEN	P interaction
**SOLVD (Enalapril)**	20%	General death	HR 0.84 (0.74 – 0.95)	-----	-----
**CIBIS II (Bisoprolol)**	20%	General death	HR 0.66 (0.54 – 0.81)	H: HR 0.53 (0.42 – 0.67) M: HR 0.37 (0.19 – 0.89)	NS
**RALES (Spironolactone)**	27%	General death	HR 0.69 (0.58 – 0.82)	H: HR 0.71 (0.60 – 0.82) M: HR 0.72 (0.57 – 0.97)	NS
**PARADIGM-HF (Sacubitril-Valsartan)**	21%	CV death or hospitalization due to HF	HR 0.80 (0.73 – 0.87)	H: HR 0.80 (0.73 – 0.87) M: HR 0.79 (0.66 – 0.94)	NS
**DAPA-HF (Dapagliflozin)**	23%	CV death or hospitalization due to HF	HR 0.75 (0.65-0.85)	H: HR 0.73 (0.63 – 0.85) M: HR 0.79 (0.59 – 1.06)	NS
**EMPEROR-Reduced (Empagliflozin)**	24%	CV death or hospitalization due to HF	HR 0.75 (0.65– 0.86)	H: HR 0.80 (0.68 – 0.93) M: HR 0.59 (0.44 – 0.80)	NS

CV: cardiovascular; M: men; HR: hazard ratio; HF: heart failure; W: women; NS: nonsignificant.

The pathophysiology of HFpEF is directly related to obesity, increased insulin resistance and metabolic syndrome. Thus, weight reduction and physical activity encouragement are essential to prevent HFpEF, especially in women ( Figure 4.2 ). Regarding pharmacological treatment, the evidence is not so robust, and, even until recently, there was no medicamentous therapy recommended.^9^Randomized studies have shown a neutral effect of the major dugs used for HF.^77^Subanalyses of two of those studies, one involving spironolactone (TOPCAT) and another involving ARNI (PARAGON-HF), have suggested different responses to therapy according to sex. In addition, SGLT2 inhibitors have shown benefits in HFpEF with similar results in men and women ( Table 4.5 ).^10^Thus, current evidence points to benefit from iSGLT2 in both sexes and potential benefit from spironolactone and sacubitril-valsartan in women.^76^Prospective studies to assess differences between sexes are necessary.

**Table 4.5:** Differences in outcomes between men and women in studies on HFpEF.

CLINICAL STUDY	% Women	OUTCOME	GENERAL RESULT	MEN / WOMEN	DIFFERENCES between sexes	P interaction
TOPCAT (Spironolactone)	52%	CV death or hospitalization due to HF or CPA	HR 0.89 (0.77 – 1.04)	General death H: p=0.68 M: HR 0.66 (0.48 – 0.90)	Significant reduction of general death only in women	0.02
PARAGON-HF (Sacubitril-Valsartan)	52%	CV death or hospitalization due to HF	HR 0.87 (0.75 – 1.01)	H: HR 1.03 (0.85 – 1.25) M: HR 0.73 (0.59 – 0.90)	Significant reduction of CV death or hospitalization due to HF and total hospitalizations only in women	0.017
EMPEROR-Preserved (Empagliflozin)	45%	CV death or hospitalization due to HF	HR 0.79 (0.69 – 0.90)	H: HR 0.81 (0.69 – 0.96) M: HR 0.75 (0.61 – 0.92)	Significant reduction of CV death or hospitalization due to HF in men and women	NS
DELIVER (Dapagliflozin)	44%	CV death or hospitalization due to HF or visit to the emergency room	On going			

CV: cardiovascular; M: men; HR: hazard ratio; HF: heart failure; W: women; NS: nonsignificant; CPA: cardiopulmonary arrest.

Usually, in both chronic and acute HF, clinical trials especially directed to women are scarce and the evidence derive from subanalysis of large studies in which women are underrepresented. However, the identification of potential RFs for the development of HF in women and

the institution of measures for their control, or even their reversion, can impact prognosis. Prospective multicenter registries could provide more accurate evidence in the female population.

## 4.3. Arrhythmias

There are some electrophysiological differences between sexes regarding the occurrence, clinical symptoms, and prognosis of arrhythmias, and they can affect both depolarization and repolarization. Women more often have sinus tachycardia and nodal reentry tachycardia, while ventricular arrhythmias are less common. In the long QT syndrome type 2, women are known to have a higher risk of sudden death and of pro-arrhythmia from cardiovascular or non-cardiovascular drugs. Pregnancy increases the risk of supraventricular tachycardia and reduces the occurrence of *torsades de pointes,* whose risk is increased in the long QT syndrome during the puerperium.^70^

### 4.3.1. Ventricular Tachycardia and Cardiac Sudden Death

The CSD is an important public health problem of high global incidence. In the United States of America, in 2016, there were 366 494 CSD, and 178 823 (48.8%) in women. The most common cause of CSD is IHD; in women, however, CSD more often is due to non-ischemic causes. In women, ventricular tachycardia/fibrillation is less documented (19.4% women *vs* 26.7% men, p<0.001), which reduces the probability of survival as compared to men, who are resuscitated and treated with defibrillator.^70^

The ICD is the therapy of choice for the primary or secondary prophylaxis of CSD. However, women are underrepresented in clinical trials (inclusion of women varies from 16% to 29%) and the sex-based analyses of subgroups are limited.^78^One registry of 236 084 Medicare beneficiaries from 1991 to 2015 evidenced that men received more ICD than women in both primary (3.2 times more) and secondary (2.4 times more) prevention. In addition, women are at a higher risk for complications related to the ICD procedure (7.2% *vs* 4.8%, p<0.001), particularly pneumothorax requiring intervention, cardiac tamponade, and ICD mechanical complications that need revision.^13^

### 4.3.2. Atrial Fibrillation

Around the world, 29.4 million women are estimated to have AF. Although the AF incidence is higher among men, older women more often have AF, considering their life expectancy is longer.^1^Women with arrhythmia have more symptoms and report poorer quality of life as compared to men ( Figure 4.3 ),^79^in addition to having peculiar predictors of AF. A study with 34 221 women has reported that SAH was a risk predictor, obesity was another important risk marker, and the dynamic weight changes were deleterious. Women who practice vigorous physical activity have a 28% reduction in the incidence of AF. Regarding middle-aged healthy women, an increased incidence of AF was observed in those who had two or more drinks per day, the excessive consumption of alcohol being a predictor of AF among them. A large prospective study has shown the association of multiparity and higher risk. Another prospective study, assessing 30 034 menopausal women, has shown no increase in the risk in addition to that related to age, while monotherapy with estrogen associated with an increased risk of arrhythmia.^80^

**Figure 4.3:** Differences of symptoms between women and men with atrial fibrillation. 72

Women more often have paroxysmal AF and higher CHA_2_DS_2_-VASc score, but are less often submitted to both cardioversion and AF and flutter ablation. In addition, they more often undergo ablation of the atrioventricular node and pacemaker implantation, more often have strokes and thromboembolism, and are more often hospitalized due to all causes.^14^

Few studies on heart rate and rhythm control have assessed the differences between sexes. Women less often undergo catheter ablation with isolation of the pulmonary veins and usually have worse results, because of later indication, more comorbidities, or higher presence of atrial fibrosis and foci outside the pulmonary veins.^15^A study with 2789 patients with recent-onset AF and large women’s representativity (46%) has shown that early rhythm control associated with a smaller risk of adverse cardiovascular events, without affecting the length of hospital stay.^81^

Women with AF are at higher risk for stroke, which is even more severe and extensive at ages over 65 years.^15 , 70^Studies with DOACs (dabigatran, apixaban, rivaroxaban, and edoxaban) have shown a smaller risk of major bleeding in women, but a higher risk of stroke/systemic embolism with the use of warfarin, although women represented only 35% to 40% of those studies’ samples.^15^There was a reduction in all-cause mortality and a significant reduction in the risk of intracranial hemorrhage with the use of DOACs as compared to warfarin in women. In studies assessing the left atrial appendage occlusion as an alternative to anticoagulation for stroke prevention in AF, women were underrepresented and the primary outcome of efficacy composed by stroke, systemic embolism, and cardiovascular death did not vary between sexes.^15 , 70^Women with AF have a higher risk of cardiovascular and all-cause mortality, in addition to more cardiac events, and a higher risk of stroke and HF.^15^The major differences of symptoms between sexes in AF are shown in Chart 4.1 .

**Chart 4.1:** Summary of the differences of symptoms between sexes in individuals with atrial fibrillation. 15

WOMEN		MEN
**EPIDEMIOLOGY AND RISK**		
Higher than in the total population Higher risk of death and stroke due to AF	Increase of incidence and prevalence with time	Age-adjusted prevalence
**SYMPTOMS AND QUALITY OF LIFE**		
Longer symptom duration Worse quality of life in the scores		
**PREVENTION AND RISK OF STROKE**		
Stroke due to AF is more severe and disabling		Receive more anticoagulation for prevention Longer time in the therapeutic range with warfarin
**HEART RATE AND RHYTHM CONTROL**		
Higher risk of CV events and pro-arrhythmia with AAD Higher risk of adverse events with catheter ablation		Higher indication for cardioversion and AF ablation

AAD: antiarrhythmic drugs; AF: atrial fibrillation; CV: cardiovascular.

## 4.4. Cardiovascular Disease and Cancer

Cardiovascular diseases and cancer are the major causes of death worldwide, and they share RFs, such as age, obesity, tobacco use, family history, and diet.

Women’s most common malignant neoplasms are breast, lung, and colorectal cancers, responsible for 50% of the new diagnoses, of which breast cancer represents 30%.^82^

Advances in the early diagnosis and treatment of cancer caused an important reduction in mortality, mainly due to the perception of acute and chronic adverse cardiovascular effects resulting from the oncological treatment, which affect the quality and expectancy of life of the survivors, who require lifelong follow-up.

The different forms of treatment, such as chemotherapy and radiotherapy, can lead to cardiotoxicity through different mechanisms, the risk being directly related to detection of the underlying cardiac disease, cardiovascular RFs, and previous cardiotoxic oncological treatment.

Although there is no risk score validated, age and LVEF previous to treatment are two strong risk predictors suggested.^83^

The diagnosis of cardiotoxicity can be done through the confirmation of new cardiovascular change during or even years after treatment, such as a clinical change and/or alteration in cardiovascular biomarkers and/or imaging, and it is an exclusion diagnosis done after investigating other etiologies.

According to the Brazilian Guidelines on Cardio-Oncology, ventricular dysfunction related to cancer therapy is defined as a reduction ≥ 10% in LVEF to a value below the lower limit of normality (LVEF < 50%), and cardiovascular imaging should be repeated in 2 to 3 weks.^84^

Ventricular dysfunction can occur during treatment or even years after its end, and that is the reason long-term surveillance is important, mainly in those who develop cardiotoxicity during treatment or are at high risk for that.

Echocardiography is the method of choice to detect myocardial dysfunction before, during, and after cancer treatment. Three-dimensional echocardiogram has better accuracy to assess ventricular function. In its absence, two-dimensional Simpson is recommended to assess volumes and LVEF. The SLG is a tool to predict with high sensitivity the later reduction in LVEF. A reduction ≥ 15% in SLG as compared to baseline is considered abnormal and an early ventricular dysfunction marker. Magnetic resonance imaging is gold-standard for the assessment of cardiac function, indicated in cases with limitation of echocardiography.^84^

It is fundamental to detect subclinical cardiac abnormalities that can influence the clinical decisions regarding the choice of treatment, indication for cardioprotection, or increase of surveillance, such as asymptomatic ventricular dysfunction.

Anthracycline is the most used CTX drug for breast cancer treatment and has a histologically proven cardiotoxic effect via necrosis of cardiomyocytes, which leads to irreversible lesions. The association of cumulative anthracycline doses and the risk of HF is exponential: 5% incidence of HF with a cumulative dose of 400 mg/m^2^, and 48% incidence of HF with a cumulative dose of 700 mg/m^2^.^85^

Another CTX drug used for breast cancer treatment, trastuzumab, can cause cardiotoxicity, and, because it is not associated with necrosis of cardiomyocytes, the lesions caused are partially or totally reversible after treatment interruption.

Fluoropyrimidine, used to treat colorectal cancer, can cause vasospasm and consequent myocardial ischemia, with or without electrocardiographic changes. The symptoms can appear at any time during treatment, but bolus injection can be less cardiotoxic, because the vasospasm can be related to accumulation of metabolites rather than to the peak effect of the drugs.^86^

If the patient develops HF and LVEF < 40% during treatment, the CTX drug should be temporarily suspended according to a decision shared by the cardiologist and the oncologist, and the HF therapy should be initiated based on guidelines and consensus.

Cardiotoxicity prevention should be performed in all patients undergoing cancer treatment. General measures, such as proper control of RFs, specific cardioprotective pharmacological interventions, and imaging- and biomarker-based surveillance strategies have an overall beneficial effect, but there is heterogeneity in results and no consensus on the recommendations for cardioprotective pharmacotherapy ( Figure 4.4 ).^85^

**Figure 4.4:** Measures for the prevention of cardiotoxicity in women. CVRF: cardiovascular risk factor

Although the recommendations are controversial, cardioprotective drugs have been tested, such as dexrazoxane, an iron chelator with proven cardioprotective action, and neurohormonal inhibitors that have a cardiac anti-remodeling action, such as renin-angiotensin-aldosterone system inhibitors, beta-blockers, and statins.^86^The use of dexrazoxane in patients with metastatic breast cancer with high cumulative dose of anthracycline (doxorubicin dose > 250 mg/m^2^) should be considered.^86^

Aerobic exercise is considered a promising nonpharmacological strategy to prevent and/or treat CTX-induced cardiotoxicity. If there is a reduction in LVEF that meets the definition of cardiotoxicity, the guideline-based treatment of HF should be considered.

Figure 4.5 summarizes the major aspects related to cardiotoxicity.

**Figure 4.5:** Summary of the major aspects related to cardiotoxicity. 2D: two-dimensional; 3D: three-dimensional; CVRF: cardiovascular risk factor; CTX: chemotherapy; DM: diabetes mellitus; EF: ejection fraction; MRI: magnetic resonance imaging; SAH: systemic arterial hypertension.

Charts 4.2 , 4.3 , 4.4 show the recommendations for the management of ischemic heart disease, heart failure, and arrhythmia in women.

**Chart 4.2:** Recommendations for the management of ischemic heart disease in women.

Recommendations for the management of ischemic heart disease in women			

Recommendation	CR	LE	References
In obstructive and non-obstructive coronary disease, the differences in clinical findings of ACS/CCS should be known, diagnosis and treatment should be improved, and the classical CVRFs and female-sex-specific RFs should be identified and treated.		**B**	^69–73^
In obstructive and non-obstructive coronary disease, the risk enhancing factors should be identified (psychosocial factors and social determinants of health).		**B**	^69–73^
In spontaneous dissection, minimize the emotional triggers, avoid hormone therapy (estrogen, progesterone, β-human chorionic gonadotropin), avoid future pregnancies, cardiac rehabilitation, preferably with modified protocol, avoiding heavy isometric exercises and intense aerobic activities.		**B**	^69–71,73^
In vasospasm, avoid aggravating factors: use of illegal drugs, amphetamines, butane gas, alcohol, and medications for migraine.		**B**	^69,71–73^
In microvascular disease, it is important to modify CVRFs (body weight loss and stress control).		**B**	^69,72^
In coronary thrombosis and embolism, early diagnosis of the underlying causes and thrombosis/infection prophylaxis are important.		**B**	^69,73^

CR: class of recommendation; LE: level of evidence; ACS/CCS: acute coronary syndrome/chronic coronary syndrome; CVRF: cardiovascular risk factor; RF: risk factor.

**Chart 4.3:** Recommendations for the management of heart failure in women.

Recommendations for the management of ischemic heart failurein women			

Recommendation	CR	LE	References
Takotsubo syndrome is related to menopause (estrogen deficiency), but hormone replacement has no impact on reducing the risk of HF.		**C**	^10,76^
Control risk factors that predispose to PPCM: hypertension, diabetes, obesity, eclampsia.		**B**	^10,76^
In HFpEF and HFrEF, control risk factors, such as body weight and glucose, lipid and blood pressure levels, practice regular physical activity, and cease tobacco use.		**B**	^10,74–76^
Use of statin would have a potential benefit, but there is scarce specific sex-related evidence, thus, it should not be recommended.		**C**	^10^
The identification of potential risk factors for the development of HF in women and the institution of measures to control them or even reverse them could impact prevention.		**B**	^10,70,76^

CCR: class of recommendation; LE: level of evidence; HF: heart failure; HFpEF: heart failure with preserved ejection fraction; HFrEF: heart failure with reduced ejection fraction; PPCM: peripartum cardiomyopathy.

**Chart 4.4:** Recommendations for the management of arrhythmias in women.

Recommendations for the management of arrhythmias in women			

Recommendation	CR	LE	References
Obesity and SAH are predictors of risk for AF in women; the control of body weight and blood pressure levels is an important preventive measure.		**B**	^14,15,81^
The prevalence of AF increases with age and in women after menopause; menopausal hormone therapy has no beneficial effect.		**C**	^80^
Vigorous physical activity seems to reduce the incidence of AF in women.		**B**	^80^
Excessive alcohol consumption is a predictor of AF in women; alcohol consumption control to less than 2 drinks/day can reduce the risk.		**B**	^80^

CR: class of recommendation; LE: level of evidence; AF: atrial fibrillation; SAH: systemic arterial hypertension.

## 4.5. Stroke

Women face a disproportionately high burden of mortality and disability from stroke.^87^Multiple specific RF have been observed, such as pregnancy, PE, GD, use of oral contraceptives, postmenopausal use of hormones, and women’s hormonal variations.^88^The strongest or most prevalent RF for stroke in women are: AF, migraine with aura, DM, SAH, depression, and psychosocial stress. Despite the higher proportion of stroke throughout life, there are no specific guidelines on screening and treatment to reduce the risk of stroke in women.^68^

It is worth emphasizing the need for awareness and systematic education of the youngest age groups, including women at childbearing age, alerting to the increasing incidence of stroke with age and its association with obstetric complications, such as GH, premature births, and GD, in addition to hormonal contraception. The risk is progressively higher in the presence of classical RF, such as obesity, dyslipidemias, SAH, and DM, which can appear throughout life, including at younger ages.^87^

Although more than half of the cases of stroke occur in women, the inclusion of women in clinical trials on stroke treatment has been smaller than that of men. A better representation of the female sex in those studies is necessary.^89 , 90^

## 4.6. Peripheral Artery Disease

Peripheral artery disease is a prevalent condition with substantial morbidity and mortality, rarely diagnosed and treated in the general population.^91^Its frequency in women is equal to that in men.^92^Differences in pathophysiology and RFs can contribute to the late and often atypical presentation of PAD in women ( Figure 4.6 ).

**Figure 4.6:** Differences in peripheral artery disease presentation in women. 7 CVD: cardiovascular disease; CRD: chronic renal disease; IHD: ischemic heart disease; PAD: peripheral artery disease; RF: risk factor; SAH: systemic arterial hypertension.

Unawareness about PAD hinders the outcomes of all patients, but the challenges are even bigger for women, who, as compared to men, suffer from more severe pain, have poorer quality of life, and are at higher risk for CVD

and their concomitant events.^93^In addition, the use of evidence-based therapy is less frequent in women as compared to men.^97^

Although the recognition and control of traditional RFs are important in both sexes, they seem to have a different effect and smaller general significance for women with PAD than for men.^98 , 99^Because the treatment of traditional RFs offers protection for both sexes, ideal care should be ensured to everybody.^100^Recognizing sex-specific RFs (ex. pregnancy) or predominantly female factors (ex. depression) can allow adequate risk stratification and adoption of early preventive measures.^101 , 102^

## 4.7. Dementia

Although the impacts of menopausal estrogen deprivation on women’s cognitive function are not clearly known, literature data have shown that women seem to be at higher risk of developing dementia and age-related memory changes than men, and little is known about such differences at younger ages.^16^Studies have shown that perimenopausal women respond better to memory-related tasks, which attenuates years after menopause.^16 , 103^

There is epidemiological and biological evidence that serum estrogen concentration and, thus, ovarian activity relate to better memory and cognition performance.^16 , 103^Symptoms, such as dullness of mind, forgetfulness, and difficulty to find the words are common in perimenopausal women.^16^In addition, as shown in the SWAN study, the reduced cognitive function in women seems to be related to neither anxiety nor depression, factors known to be increased in that phase of women’s life.^65^

One of the major pathologic characteristics of AD, a cause of dementia in the general population, is the accumulation of amyloid plaques in the central nervous system. Data from the literature have suggested that estrogen can have an important role in preventing amyloid buildup, in addition to acting on the cognitive function.^104^Researchers have suggested that the reduced concentration of sexual steroid hormones after menopause can account for the higher prevalence and severity of AD in women.^105^Several studies have been performed to define whether MHT would be effective to prevent those neurological disorders.^105 , 106^

Despite the conflicting epidemiological evidence regarding the use of MHT as a preventive measure against cognitive dysfunction and AD reduction in menopause, a meta-analysis of clinical trials has shown the absence of those benefits. Women over the age of 65 years without dementia who used estrogen isolated or associated with progesterone showed no consistent evidence regarding those benefits, not even the younger ones (early menopause and perimenopause).^106^

Thus, we suggest that MHT should not be prescribed to preserve cognitive function in postmenopausal women. In addition, there is lack of robust evidence of cognitive benefits for women using MHT at younger ages (for example, perimenopausal). Thus, MHT should not be prescribed to preserve cognitive function in younger women. Even results of the WHI study have not shown that the early initiation of MHT would be necessary to provide later cognitive benefits in women, not even prevent IHD (“the critical window hypothesis”).^107^

A cohort study has shown the major causes of cognitive changes and dementia in younger men and women (under the age of 55 years): AD (34%), vascular dementia (18%), frontoparietal dementia (12%), alcoholism-related dementia (10%), and others, such as Parkinson’s disease and multiple sclerosis.^108^In AD, 10% of the cases related to a genetic mutation and the prevalence of dementia doubled every 5 years after the age of 35 years.

There are vascular RFs related to early dementia, such as stroke, transient ischemic attack, renal disease, CVD, SAH, chronic alcoholism, and drug intoxication.^109^

## 4.8. Valvular Heart Disease

Rheumatic disease is the major cause of acquired valvular heart disease in children and young adults aged less than 40 years in emerging countries.^110^

### 4.8.1. Aortic Stenosis

In young women, the etiology of aortic stenosis isolated is congenital and often the structural lesion is bicuspid valve.^17^Aortic stenosis isolated of rheumatic etiology is rare, usually concomitant with other valvular lesions, such as aortic insufficiency or mitral lesions. With aging, degenerative valvular calcification is the major mechanism of disease. When compared to men, considering the same degree of valvular calcification, women tend to higher severity of aortic stenosis due to the valvular apparatus fibrosis, which is more pronounced than calcification.

Women with low-gradient aortic stenosis and low ejection fraction do not value their symptoms and present at more advanced disease stages, with worse functional capacity, more frequent syncope, and more eccentric left ventricular remodeling. Such manifestations can justify their higher mortality as compared to men ( Figure 4.7 ).^17^

**Figure 4.7:** Sex-related differences in clinical course of low-gradient aortic stenosis with low ejection fraction. Adapted from Bartko et al. 110

Women referred for aortic valve surgical replacement have more severe dyspnea on exertion, higher frailty scores, and more severe valvular obstruction degree for the same anatomic pattern of valvular area and mean transvalvular aortic pressure gradient.^18^Women’s smaller body surface implicates in technically more delicate surgical interventions, explaining the worse postoperative survival.

Of the patients submitted to transcatheter aortic valve implantation, women are older, have better left ventricular function, lower prevalence of CAD, but have comorbidities associated with DM and AF. Anatomical characteristics of the female sex, such as the shorter distance between coronary ostia and valvular ring and higher prevalence of valvular and aortic calcification, account for the higher incidence of coronary obstruction during the procedure. The smaller diameter of peripheral vessels causes more vascular complications and bleeding.^111^

Currently, there is no therapy to prevent the progression of aortic stenosis.^18^However, clinical trials have formulated strategies to delay disease progression with DPP-4 inhibitors,^18^tested in elderly and individuals with DM, but with no specific reference to women. The lack of control of tobacco use, dyslipidemia, creatinine serum levels and plasma calcium levels seems to cause an annual absolute and percent reduction in the aortic valve area.^111 , 112^

### 4.8.2. Mitral Valve Disease

Mitral insufficiency has an etiology-dependent natural history, and its clinical follow-up requires preventive measures based on the stratification of the disease’s anatomical degree. Mitral insufficiency can have an insidious course, thus it requires the periodic assessment of the development of anatomical and/or functional changes secondary to valvular disease and the appearance of complicating factors. Mitral insufficiency resulting from valvular prolapse is prevalent among women and its peculiar anatomic characteristics show predominance of myxomatous valves with anterior and bilateral leaflet prolapse, and extensive thickening of leaflets, which are less flaccid than in men.^113^

Women have smaller regurgitation volumes and atrial dimensions when indexed to body surface; therefore, many do not meet surgical criteria based on the increase of ventricular cavities in mitral insufficiency,^113^which can result in worse results after surgery.^114^However, those data are controversial because they are based on retrospective studies with a very limited cohort ( Figure 4.8 ).

**Figure 4.8:** Comparative evolution after minimally invasive surgery of the mitral valve between women and men. Adapted from Seeburger et al. 113

In Brazil, rheumatic mitral stenosis is the major cause of acquired valvular disease in young women; however, calcification of the mitral valve apparatus can occur as age advances. In industrialized countries, with the eradication of rheumatic disease, the degenerative causes predominate,^115^with calcification and involvement of the base of leaflets and absence of commissural fusion, structural characteristics that limit percutaneous intervention. Therefore, surgical replacement of the mitral valve has been preferred to percutaneous/surgical commissurotomy, except in the presence of comorbidities that increase the surgical risk.

### 4.8.3. Rheumatic Disease

Primary prevention of acute rheumatic fever involves the prompt diagnosis and antibiotic treatment of the group A streptococcal infection with benzathine penicillin or other alternatives.^116^For both sexes, hygiene measures are essential to prevent disease spread and should be reinforced. Patients with history of acute rheumatic fever are at a high risk of recurrence and cardiac involvement in the case of subsequent group A streptococcal infection. For patients at risk of recurrence, long-lasting secondary antibiotic prophylaxis should be done, as recommended by the current guidelines in both sexes.

Of the preventive measures against valvular heart disease in women, prophylaxis of infective endocarditis during delivery should be emphasized. Although controversial, the Brazilian Society of Cardiology Statement for Management of Pregnancy and Family Planning in Women with Heart Disease recommends prophylaxis in high-risk situations, indicating specific antibiotics for the genitourinary tract, such as ampicillin and gentamicin or vancomycin, in case of allergy to penicillin ( Figure 4.9 ).^11^

**Figure 4.9:** Guidance for prophylaxis against infective endocarditis in pregnancy. IE: infective endocarditis.

## 4.9. Diabetes Mellitus, Preeclampsia, and Pregnancy Hypertensive Disorders

### 4.9.1. Diabetes Mellitus

Diabetes mellitus manifests during pregnancy as type 1 DM, type 2 DM, or GD, and associates with maternal and fetal complications, such as PE, prematurity, and perinatal death. Management of DM during pregnancy should be individualized according to the presence of prognostic determinants of pregnancy, such as blood glucose level, disease duration, presence of comorbidities, target-organ lesions, polyhydramnios, fetal macrosomia, and other fetal malformations.^19^

The prevention of those complications involves lifestyle changes, mainly body weight control and healthy diet before and during pregnancy. A meta-analysis with 44 studies, assessing the influence of physical activity and nutrition on body weight control in 7278 pregnant women, has shown a mean reduction of 1.42 kg in weight gain and a reduction in the risk of PE (RR 0.74; CI 0.60 – 0.92), with no fetal impairment.^117^

Regular physical exercise practice improves functional capacity, reduces the risk of depression, prevents excessive weight gain, helps control metabolic and cardiovascular disorders, mainly PE, other forms of SAH, and DM developed during pregnancy. When physical activity is moderate or vigorous, there is a reduction in the GD risk by 50% and in the PE occurrence by 22% and 35%, respectively. The risk is even lower if the physical activity is initiated before and maintained since the beginning of pregnancy. Studies have shown that 5 to 6 hours/week of physical exercise reduce the risk of PE by 40%. Thus, women without obstetric or cardiovascular contraindications should be physically active throughout the entire pregnancy. The recommendation is 150 minutes of moderate exercise, comprising aerobic and resistance training, stretching, and yoga, distributed in at least three days per week.^118^

### 4.9.2. Pregnancy Hypertensive Disorders

Pregnancy hypertensive disorders, comprising different forms of SAH, are among the major causes of maternal and perinatal complications and mortality around the world, being considered indisputable markers of future CVD.^119^

Several strategies have been proposed to prevent PE; however, none is unequivocally effective. Nutritional interventions, such as vitamins C and E, fish oil, garlic supplementation, vitamin D, folic acid, or sodium restriction,^120^have no proven efficacy. Calcium replacement is one of the few strategies that showed benefit for patients at high risk for SAH or PE, mainly those whose daily dietary calcium intake is insufficient. A systematic review of 13 studies including 15 730 patients with low daily dietary calcium intake (< 600 mg/day) has shown that a supplementation equal to or higher than 1.0 g/day reduced the relative risk of SAH by 35% and that of PE by 55%, and higher doses (≥ 1g) were more beneficial regarding those outcomes.^121^

The use of ASA between the 12^th^and 16^th^weeks of pregnancy at daily doses between 75 mg and 150 mg is considered moderately effective to reduce the risk of PE in high-risk patients ( Figure 4.10 ).^11^

**Figure 4.10:** Recommendations for the use of acetylsalicylic acid in preeclampsia prophylaxis. Adapted from the Brazilian Society of Cardiology Statement for Management of Pregnancy and Family Planning in Women with Heart Disease – 2020. 11 APS: antiphospholipid antibody syndrome; ASA: acetylsalicylic acid; BMI: body mass index; DM: diabetes mellitus; FH: family history; NB: newborn; PE: preeclampsia; SAH: systemic arterial hypertension; SGA: small for gestational age.

The ASPRE trial ( *Performance of Screening for Preterm Preeclampsia)* , using a PE prediction model (Doppler ultrasound in the first trimester, mean blood pressure measurement, and inflammatory markers), has shown a 62% reduction in the relative risk of PE with 150 mg of ASA at night, initiating between the 11^th^and 13^th^gestational week and ending at the 36^th^gestational week.^122^A recent review corroborated the 18% reduction in the relative risk of proteinuria in PE, as well as a reduction in the number needed to treat to 61, with low fetal, neonatal, and postpartum bleeding risk.^123^

Regarding GH as a RF for future CVD, a systematic analysis^20^has shown that GH in primigravida was associated with a higher risk of CVD (RR, 1.45; 95% CI, 1.17-1.80) and IHD (RR, 1.46; 95% CI, 1.23-1.73), but of neither stroke (RR, 1.26; 95% CI, 0.96-1.65) nor thromboembolic events (RR, 0.88; 95% CI, 0.73–1.07). Women with one or more pregnancies with GH had a higher risk of CVD (RR, 1.81; 95% CI, 1.42-2.31), IHD (RR, 1.83; 95% CI, 1.33-2.51) and HF (RR, 1.77; 95% CI, 1.47-2.13), but not of stroke (RR, 1.50; 95% CI, 0.75-2.99). Additional studies are necessary to assess the correlation between GH and subsequent CVD.

## 4.10. Adolescent Pregnancy

Of the health problems in adolescence (individuals between 10 years and 20 years incomplete), pregnancy represents 400 000 cases per year, and children from adolescent mothers represented 18% of the live births in Brazil in 2015, with demographic predominance in the Northeastern region.^46^Of the factors contributing to increase the occurrence of adolescent pregnancy, the following stand out: early sexual activity start, inadequate use of contraceptives, difficulty in accessing family planning programs, and mainly misinformation on sexual and reproductive rights ( Figure 4.11 ).^125^

**Figure 4.11:** Significant factors of teenage pregnancy. 125

According to the WHO, adolescent pregnancy increases the likelihood of maternal, fetal, and neonatal complications, in addition to worsening previous socioeconomic problems and influencing the future of generations.^126^The factors contributing to maternal complications in adolescent pregnancy are shown in Figure 4.12 .^125^

**Figure 4.12:** Factors contributing to maternal complications of adolescent pregnancy. 125

According to the Brazilian Society of Pediatrics, sexual abstinence isolated is not a strategy to reduce the adolescent pregnancy rates. In addition, studies have shown that abstinence neither contributes to delay the start of sexual life nor reduces the number of partners among adolescents. According to those studies, sexual education alone for abstinence has no significant effect on reducing adolescent pregnancy (OR 0.7; 95% CI, 0.38-1.45; p = 0.38) as compared to broader sexual education, which proved more effective (OR 0.4; 95% CI, 0.22-0.69; p = 0.001).^21^

The Brazilian Society of Pediatrics’ Practical Guide to Prevent Adolescent Pregnancy considers that one of the most important prevention factors is education on sexuality and reproductive health supported by scientific evidence and health promotion programs.^125^The guidance should focus on biological aspects, mutual respect, responsible sexual activities, and use of safe and effective contraceptive methods to prevent pregnancy and protect against sexually transmitted infections.^127^

Prescribing contraception to adolescents is a hard task, because building a trustful relationship requires time. However, pediatric healthcare providers are considered the most reliable professionals by adolescents and their families, and usually the only source of reproductive counselling.^128^Strategies for successful reproductive counselling are shown in Chart 4.5 .

**Chart 4.5:** Strategies for pregnancy prevention. 125

In the clinic for adolescents, have an exclusive space to provide information on sexual health and contraceptive methods
Ensure confidentiality about the discussions on one’s sexuality
Present contraceptives emphasizing efficacy, tolerance, side effects, contraception advantages, and clinical benefits of the methods
After prescription, follow up to identify side effects, adhesion, and satisfaction
Regulate long-acting reversible contraceptive methods and hormonal contraceptives in the pediatric practice

Current guidelines and best practices for contraception include the U.S. Medical Eligibility Criteria for Contraceptive Use (MEC),^132^the WHO Selected Practice Recommendations for Contraceptive Use, and the Recommendations and Reports of the U.S. Center for Disease Control.^129^The Brazilian Federation of Gynecology and Obstetrics reinforces the last version of the MEC-WHO document, which indicates that age alone is not the reason to delay the use of any reversible method, and that social and behavioral issues should be considered individually.^131^

Contraceptives can be divided into hormonal and non-hormonal (behavioral, mechanical and barrier methods) and have variable contraceptive failure. Their efficacy can be calculated by use of the Pearl index, which considers the number of pregnancies/100 women/year ( Figure 4.13 ).^128 , 132^

**Figure 4.13:** Methods of contraception and Pearl index (pregnancies/100 women/year).

Source: Adapted from U.S. Medical Eligibility Criteria for Contraceptive Use, 2016.^132^IUD: intrauterine device.

Charts 4.6 , 4.7 , and 4.8 show the recommendations for the management of cardiotoxicity, stroke, PAD, dementia and other female-sex-specific diseases and situations.

**Chart 4.6:** Recommendations for the management of cardiotoxicity in women.

Recommendations for the management of cardiotoxicity in women			

Recommendation	CR	LE	References
Identification and control of risk factors for cardiovascular diseases, such as hypertension, diabetes, obesity, tobacco use, and dyslipidemia.	>	**B**	^85,86^
Use of cardioprotective drugs: anti-remodeling drugs can be considered for patients with signs of subclinical cardiotoxicity or patients at high cardiovascular risk.	a	**B**	^85,86^
For patients with EF of 40-50%, ACEI/AT1 and beta-blocker should be initiated before treatment.		**A**	^85,86^
Consider the use of dexrazoxane for patients with metastatic breast cancer with elevated cumulative dose of anthracycline (doxorubicin > 250 mg/m^2^).		**A**	^85,86^
Surveillance strategies with biomarkers and imaging.		**C**	^85^
Aerobic exercise is considered a promising nonpharmacological strategy to prevent chemotherapy-induced cardiotoxicity.		**B**	^85,86^

CR: class of recommendation; LE: level of evidence; ACEI: angiotensin-converting-enzyme inhibitor; AT1: angiotensin receptor type 1; EF: ejection fraction.

**Chart 4.7:** Recommendations for the management of stroke, PAD, and dementia in women.

Recommendations for the management of stroke, PAD, and dementia in women			

Recommendation	CR	LE	References
Strengthen women’s awareness of the progressive incidence of stroke and its association with obstetric complications (GH, premature birth, GD) and hormonal contraception.		**A**	^87^
Alert women about the progressively higher risk of stroke in the presence of classical risk factors, such as obesity, dyslipidemias, SAH, and DM.		**B**	^87^
Treatment of traditional risk factors protects against PAD in both sexes.		**B**	^100^
In PAD, the recognition of sex-specific risk factors, such as pregnancy complications, or predominantly female factors, such as depression, allows proper risk stratification and adoption of early preventive measures.		**B**	^101,102^
Estrogen is suggested to have an important role in preventing amyloid buildup in Alzheimer’s disease, in addition to acting in the cognitive function.		**A**	^107^

CR: class of recommendation; LE: level of evidence; DM: diabetes mellitus; GD: gestational diabetes; GH: gestational hypertension; PAD: peripheral artery disease; SAH: systemic arterial hypertension.

**Chart 4.8:** Recommendations for the management of pregnancy-specific situations.

Recommendations for the management of pregnancy-specific in women			

Recommendation	CR	LE	References
For infective endocarditis prophylaxis during delivery, the association of ampicillin (2.0g) and gentamicin (1.5mg/kg) should be administered one hour before delivery in patients at high risk for infective endocarditis.		**C**	^11^
Changes in lifestyle, mainly body weight control and healthy diet, before and throughout pregnancy are important to prevent gestational diabetes.		**B**	^117^
Regular physical exercise practice during pregnancy for women without obstetrical contraindication reduces the risk of depression, prevents excessive weight gain, helps control metabolic and cardiovascular disorders, mainly PE, other forms of SAH, and DM.		**A**	^118^
Nutritional interventions, such as vitamins C and E, fish oil, vitamin D, folic acid, and sodium restriction are measures to prevent PE.		**C**	^119^
The use of ASA before the 16th gestational week reduces the risk of early PE (before the 34th week) in high-risk patients.		**A**	^122^
Calcium replacement can be beneficial to prevent PE in patients considered to be at high risk and whose daily dietary calcium intake is insufficient (< 600 mg/day).		**B**	^121^
Sexual abstinence as a measure to prevent adolescent pregnancy contributes neither to delay the beginning of sexual life nor to reduce the occurrence of pregnancy.		**B**	^126^
The prevention of adolescent pregnancy requires education on the efficacy, safety, and tolerance of the contraceptive measures available, as well as supervision until adulthood.		**B**	^129^
The prescription of contraceptives should be based on the WHO eligibility criteria.		**B**	^132^

CR: class of recommendation; LE: level of evidence; ASA: acetylsalicylic acid; DM: diabetes mellitus; PE: preeclampsia; SAH: systemic arterial hypertension; WHO: World Health Organization.

## 5. Cardiac Investigative Methods: Peculiarities in Women

In clinical practice, risk scores and symptoms are used to estimate the probability of CVR and identify individuals who would benefit from tests. Most prediction models overestimate risk, and imaging tests are usually used when the pretest probability is intermediate. Based on Bayes theorem, the proportion of false-positive tests reduces as the disease prevalence in a population increases, while the proportion of false-negative decreases as the disease prevalence in a population decreases. In women, coronary obstructive lesions are less common and the pathophysiology of IHD have peculiarities, such as involvement of the microcirculation, thinner vessels, and higher vascular reactivity with consequent vasospasm. Thus, sensitivity and specificity of the tests in women can be different from those in men. This chapter aimed to report the differences in interpretation, accuracy, and indication of the tests that help diagnose IHD in women.

### 5.1. Electrocardiogram

The ECG is influenced by sex, mainly regarding the magnitude of the electrical signals. The QRS amplitude is smaller in women, mainly in the precordial leads, such as S wave in V2 and R wave in V5, interfering with the accuracy of the LVH diagnosis. Smaller left ventricular mass and presence of large breasts are the reasons for that. The Cornell criteria to assess LVH (addition of the R wave amplitude in aVL and the S wave in V3) improve precision by considering sex, being abnormal when > 28 mm in men and > 20 mm in women.

The J point and T wave amplitude is smaller in women. However, the corrected QT interval is longer in women, being abnormal when > 470 ms in women and > 450 ms in men.^133^

### 5.2. Exercise Test

The ET is safe and physiological, indicated to investigate myocardial ischemia, exertion-induced arrhythmias, and prognosis predictors in IHD. In women, the lower levels of hemoglobin, the smaller size of the coronary arteries, the inappropriate increase in catecholamines on exertion, and estrogen, because of its similarities with the digitalis molecule, can cause a false-positive depression of the ST segment.

Because of the high prevalence in women of non-obstructive CAD, single-vessel lesions, and microvascular disease, the accuracy of ET varies with the pretest probability of IHD. According to the ACC/AHA guidelines, ET should be chosen to assess IHD in women with intermediate pretest probability, normal ECG, and who can reach maximum exercise. The ET has similar negative predictive value in women and men (78% and 81%, respectively), its positive predictive value being around 47%. Small electrocardiographic amplitude and poorer exercise performance hinder IHD assessment in women.^25 , 134^

### 5.3. Carotid Ultrasound

Measurement of IMT and atherosclerotic plaque detection are auxiliary tools to assess CVR. The IMT seems to reflect the presence of CVRFs and differs between sexes. Carotid atheromatous plaque reflects atherosclerotic burden, seems to be a stronger predictor of CVR than IMT, and does not differ between sexes. The IMT measure for risk reclassification can be used in some specific groups, in which classification by usual criteria is difficult or incomplete (family hypercholesterolemia, autoimmune disease, use of medications that induce increase in cholesterol levels) or women with at least two CVRFs. The IMT measure was studied and published in the ELSA project (Brazilian Longitudinal Study of Adult Health), which showed differences not only between sexes, but between ethnicities, and, as well known, between age ranges of a Brazilian population. Thus, the risk can be stratified in specific situations using data from the Brazilian population.^135^

The Brazilian and international guidelines recommend that the presence of atheromatous plaque be considered an aggravating factor in patients at intermediate risk, a situation in which several women can be.^136^

### 5.4. Echocardiography

Echocardiography is the most used tool in the CV diagnostic and prognostic investigation in men and women, without specific differences between the sexes. In addition to the diagnosis of regional contractility changes, it identifies other causes of chest pain, such as valvular heart diseases, cardiomyopathies, aortopathies, and pericardial diseases.^137^

Stress echocardiography is an appealing technique, mainly for young women or those at risk for breast cancer, because it does not rely on radiation. There are few studies assessing differences in stress echocardiography performance between the sexes. However, its specificity and precision are significantly higher than those of ET isolated, without significant differences between men and women. Stress echocardiography with dobutamine *versus* ET for the detection of IHD was more accurate in coronary stenosis >50% in women with chest pain, with sensitivity of 70.4% vs 53.7% and specificity of 94.6% vs 73.6%. Higher accuracy was maintained after the exclusion of patients who did not achieve more than 85% of the heart rate predicted for age before ischemia induction. The AHA recommends adding images to exercise when assessing women at intermediate risk with an abnormal baseline ECG.^25 , 138^

### 5.5. Myocardial Perfusion Scintigraphy

The accuracy of stress myocardial perfusion scintigraphy in women is similar to that of stress echocardiography, but lower as compared to that in men, possibly because of the smaller diameter of the cardiac chambers, which leads to lower image resolution in conventional gamma cameras. Another challenge is the attenuation caused by the breasts, which can lead to false-positive results.^25^In a meta-analysis comparing ET, stress myocardial perfusion scintigraphy, and stress echocardiography, the sensitivity and specificity of each method were 61% and 70%, 78% and 64%, and 86% and 79%, respectively, and were similar to those in men, except for ET, whose values were lower in women.^139^

The WOMEN Trial has compared the effectiveness of ET with or without myocardial scintigraphy in symptomatic women at low/intermediate risk for CAD with ability to exercise. The authors showed that, only in women with intermediate/high pre-test risk, diagnostic accuracy improved when ET was combined with myocardial scintigraphy, and accuracy was higher for obstructive CAD: stress myocardial perfusion scintigraphy had sensitivity of 78% (95% CI, 72%-83%) and ET had sensitivity of 61% (95% CI, 54%-68%). A negative stress myocardial perfusion scintigraphy showed an excellent prognostic value in women, with 99% survival without events, including older women and different ethnicities, and similar to that in men. Thus, the pretest probability assessment for CAD is important in deciding which method use.^140 , 141^

Exposure to radiation on scintigraphy in women seems little harmful; however, although controversial, it should be avoided in young women, for whom stress echocardiography should be preferred.

### 5.6. Coronary Calcium Score and Coronary Computed Tomography Angiography

Population scores to predict CVR have lower accuracy in women, especially in the younger ones. Currently, the radiation dose of CCaS is similar to that of mammography (< 1 mSv), and the CCaS has equal accuracy in men and women for risk stratification and prediction of events. For women with a CCaS greater than zero, the risk of events is higher than for men. Similarly, breast arterial calcification visualized on mammography correlates with the presence of coronary atherosclerosis and indicates the need for cardiological assessment.

The CCaS has prognostic value in asymptomatic women at intermediate risk. A positive CCaS in asymptomatic women at intermediate risk is associated with a significantly higher rate of events, including death, acute myocardial infarction, CABG, and percutaneous coronary intervention (3.3% in those with CCaS > 0 *versus* 1.0% in those with CCaS = 0, after a 37-month follow-up).^27^

Coronary CTA is a non-invasive anatomical test for IHD that provides information on the occlusion severity, plaque burden, and plaque instability risk. Considering the characteristics of IHD in women, coronary CTA can evidence the most prevalent non-obstructive pattern, which cannot be assessed on functional modalities. When compared to the latter, coronary CTA resulted in higher probability of coronary angiography in a obstructive IHD, in addition to a better correlation with future events. In the context of acute chest pain, because of the atypical characteristics of that complaint in women at the emergency unit, coronary CTA had a higher impact in the reducing the length of hospital stay in women than in men.^142 , 143^

### 5.7. Cardiac Magnetic Resonance Imaging

Cardiac magnetic resonance imaging is a highly accurate method to investigate CV diseases without using radiation. This makes it an excellent option for women, especially those at childbearing age, pregnant, and undergoing breast cancer treatment. It is well known that a considerable part of female patients (25%) with ventricular ejection fraction < 50% na CMRI do not have echocardiographic changes when investigating cardiotoxicity.^144^

In the functional assessment of women’s IHD, the impact of the breasts and of the smaller myocardial mass in imaging tests is well known. This limitation does not apply to CMRI, which has similar accuracy in men and women. Considering the characteristics of the IHD pattern in the female population, that is the disparity between symptoms and anatomical findings, the CMRI became an excellent option for myocardial perfusion assessment because of its high spatial resolution and identical performance, regardless of sex. In addition, CMRI can evidence perfusion and/or myocardial changes, widening the differential diagnosis scope of chest pain (ex: infarctions due to thromboembolism or coronary dissection), and be particularly useful in approaching that symptom in women.^142 , 144^

### 5.8. Coronary Angiography

There is evidence that the number of coronary angiographies performed in women is smaller than in men. However, when stratifying risk for women, the indication of coronary angiography is similar to that for men.^144^The CURE study shows that the indication of coronary angiography, angioplasty, and CABG was lower for women (48% x 61%).^145^The socioeconomic level results in higher vulnerability of access for low-income women, which could cause higher short-term mortality in ACS.^146^

A cross-sectional study with data from the VICTIM Registry has assessed patients diagnosed with STEMI from four hospitals (one public and three private hospitals) that offered primary angioplasty. The study included 878 patients with STEMI, 33.4% of whom were women. Women had smaller rates of fibrinolysis (total: 2.3%; women: 1.7%; and men: 2.6%) and of primary angioplasty (44% in women; 54.5% in men), resulting in higher in-hospital mortality (16.1% *versus* 6.7%).^146^

Normal coronary arteries on coronary angiography are more common in women, including in ACS, and can represent microvascular disease, vasospasm, or spontaneous thrombolysis.^28^

The risk of vascular complications from coronary angiography can be higher in women with thinner femoral arteries, even with manual compression or use of collagen plug. In addition, women have a higher tendency to develop contrast-induced acute kidney injury, because they have more glomeruli, and to develop anemia.^28^

Chart 5.1 shows the differences in the interpretation of the complementary tests for IHD between sexes, and their accuracy, advantages, and disadvantages in women.

**Chart 5.1:** Diagnostic complementary tests for ischemic heart disease: differences between sexes in the interpretation of the tests, accuracy, advantages, and disadvantages when used in women.

COMPLEMENTARY TEST	Test interpretation differences between sexes	Accuracy in women	ADVANTAGES	DISADVANTAGES
**ELECTROCARDIOGRAM**	Smaller QRS amplitude: S wave in V2 and R wave in V5 QT interval is longer in women (RV >470ms) J point and ST amplitude smaller in women	No difference between sexes	Uses Cornell criteria to diagnose LVH in women (RV > 20mm)	Interferes with the accuracy of LVH diagnosis by use of the Sokolow criteria
**EXERCISE TEST**	ST-segment depression as false-positive	Accuracy: 47% Negative predictive value similar to that in men: 78%	Low cost Good negative predictive value when performed in patients who reach maximum exertion	Low accuracy
**CAROTID ULTRASOUND**	IMT is smaller in women	Similar	No radiation, good availability, and relatively low cost Atherosclerotic plaque is better risk predictor than IMT May be useful in special subgroups in which the applicability of risk scores is worse	Less accurate than CCaS
**ECHOCARDIOGRAPHY**	No difference between sexes	Rest echo: No difference between sexes Stress echo: similar accuracy in both sexes	Stress echo is more accurate than ET, especially when baseline ECG is abnormal Identifies other diseases that can cause precordial pain No radiation exposure	Echo is examiner-dependent
**SCINTIGRAPHY**	Image attenuation due to the breasts Worse image resolution in conventional gamma cameras	Sensitivity 78% Specificity 64%	More accurate than ET in women with intermediate/high PTP Excellent prognostic value when normal	Breast exposure to radiation
**CORONARY CALCIUM SCORE**	CCaS > 0 in women indicates higher risk than in men	Similar accuracy for risk stratification and event prediction	Radiation similar to that of mammography (<1mSv)	Limited availability of the test
**COMPUTED TOMOGRAPHY ANGIOGRAPHY**	No difference between sexes	Similar accuracy	Identifies non-obstructive CAD, which is more prevalent in women, and better correlation with future events Reduction in the emergency department length of stay	Limited availability of the test Radiation exposure
**CARDIAC MAGNETIC RESONANCE IMAGING**	No difference between sexes	Similar accuracy	No radiation exposure Evidences perfusion and/or myocardial changes, improving the differential diagnosis scope of chest pain	Limited availability of the test
**CORONARY ANGIOGRAPHY**	No difference between sexes Women have a higher frequency of normal coronary arteries on coronary angiography when that is requested	No difference between sexes	Can diagnose vasospastic angina, when performed for that purpose	Higher risk of procedural complications: bleeding and acute renal lesion Does not assess ischemia due to microvascular disease

CCaS: coronary calcium score; CAD: coronary artery disease; Echo: echocardiography; ET: exercise test; IMT: intimal-media thickness; LVH: left ventricular hypertrophy; PTP: pretest probability; RV: reference value.

Chart 5.2 shows the recommendations and levels of evidence of the diagnostic tests for IHD in women.

**Chart 5.2:** Recommendations of diagnostic tests for ischemic heart disease in women.

Recommendations for the management of ischemic heart disease in women			

Recommendation	CR	LE	References
In chest pain, ECG maintains the usual classes of recommendation, especially in low/intermediate-risk women, considering the sex-related changes.		A	^25,133^
Exercise test is recommended in symptomatic women at intermediate risk for IHD, with normal baseline ECG and who can achieve maximum exercise.		B	^25^
Exercise test with imaging is recommended in women with ST segment-T changes at rest and who cannot exercise properly.		B	^25,140^
CCaS: women at low/intermediate or inconsistent risk: improves risk assessment. High negative predictive value. It is a "bridge" test for coronary CTA when CCaS is positive in low-risk patients.	a	A	^143^
Coronary CTA: risk stratifier in women; as effective as exercise test.	a	A	^143^
Coronary CTA: high predictive accuracy to identify obstructive CAD and when present in low-risk women.	a	A	^143^
MRI indicated in women with suspected non-obstructive IHD (microvascular disease); advantage: it involves no ionizing radiation.	b	B	^144^
MRI in women with intermediate pretest probability, ST segment-T changes at rest on ECG and who cannot exercise properly.	a	B	^144^

CR: class of recommendation; LE: level of evidence; CCaS: coronary calcium score; CTA: computed tomography angiography; ECG: electrocardiogram; IHD: ischemic heart disease; MRI: magnetic resonance imaging.

## 6. Women’s Representation in Clinical Studies on Cardiovascular Disease and Risk Factors

Sex and gender associate with environmental and occupational risks, risky behaviors, health care, and its perception by human beings, influencing the prevalence of diseases and the results of their treatments differently in men and women.^147 , 148^Considering that the pharmacokinetics and pharmacodynamics of medications differ between sexes, men and women differ in the profile of adverse events and the results of treatments.^148^Knowing this, clinical trials on diagnosis and treatment need to maintain in their samples the representativeness of men and women observed in the human population and proportional to the prevalence of the disease studied.

However, the analysis of clinical studies performed in recent decades about CVDs and their risk factors has shown that, despite some advances, women are still underrepresented in those studies.^34 , 67 , 149^

In 121 clinical trials funded by the National Heart, Lung, and Blood Institute (NHLBI) between 1965 and 1998, excluding the studies performed with only one sex, women represented on average 38% of the samples, without significant profile changes over time.^67^The analysis showed that the women’s representation in studies on CAD and SAH was proportional to the prevalence of those diseases in the female sex; however, in studies on HF and arrhythmias, the women’s representation was lower than the prevalence of those diseases in the female sex.^87^

The systematic review of 135 clinical trials used to support the recommendations of the American Heart Association’s Evidence-based guidelines for cardiovascular disease prevention in women: 2007 update has shown that the women’s representation in studies published between 1970 and 2006 increased over time (from 18% in 1970 to 34% in 2006), being higher in primary than in secondary prevention (43% *versus* 27%) and in studies on SAH, DM, and stroke, but lower in studies on HF, CAD, and dyslipidemia.^150^Despite that increase, the authors concluded that women were still underrepresented in clinical studies on CVDs and their risk factors, emphasizing that in only 31% of those studies the results were discussed based on differences between sexes.^150^

The analysis of women’s participation and of the safety and efficacy reported according to sex in clinical studies on CVD that supported 36 approvals of medications from 2005 to 2015 by the USA Food and Drug Administration has shown that women represented, on average, 46% of those studies’ samples.^34^The authors concluded that there was proper representation of women in clinical studies on SAH, AF, and PH, but underrepresentation in studies on HF, CAD, and ACS. Minimal differences between sexes were reported in the efficacy and safety profiles of the medications assessed and approved.^34^

The participation of women in 740 clinical studies on CVD registered in ClinicalTrials.gov (https://clinicaltrials.gov) between 2010 and 2017 was, on average, 38.2% of the total of 862 652 adults. Women were younger (≤ 55 years) and participated in studies of lifestyle intervention and on SAH, AF, and PH, although those studies were of shorter duration. However, in studies on high-complexity procedures, CAD, ACS, and HF, women were underrepresented.^151^

The analysis of this scenario evidences several barriers to be overcome to reach equity in representation of the subjects in clinical studies, to properly contemplate women in general as well as women from minority populations (ethnical and racial).^152 , 153^Those barriers seem to relate to patients, assistant physicians, research team, study’s design, and society,^152^and are shown in Figure 6.1 .

**Figure 6.1:** Barriers that contribute to the low representation of women in clinical studies on cardiovascular diseases and their risk factors. 152,153

Recommendations: A) scientific journals should ask authors of cardiovascular clinical studies to analyze sex/gender-specific differences; B) health equity should be integrated in the design of clinical studies, using a structure such as *PROGRESS Plus (* ***P*** *lace of residence;* ***R*** *ace;* ***O*** *ccupation;* ***G*** *ender;* ***R*** *eligion;* ***E*** *ducation;* ***S*** *ocioeconomic status;* ***S*** *ocial capital; Plus=others)* ; C) more women should integrate the research teams of clinical studies; D) medical education should comprise sex and gender particularities; E) population access to clinical trial sites should be facilitated.^152 , 153^

## 7. Primary Prevention Measures in Women

Women’s PHC occurs via the Health Care Network, according to the guidelines of the National Policy of Women’s Total Health Care from the Brazilian Ministry of Health.^154 , 155^Through the Basic Health Units, public health actions are implemented to reduce women’s morbidity and mortality throughout their life cycle, extending to their family and community.^154 , 155^

Reproductive life planning, the set of actions to regulate fertility, and the contraceptive practices that are mainly adopted by women, including lesbians and bisexuals, whose desire or right to maternity need to be ensured, should be contemplated by the PHC teams, which need to initiate the necessary measures for their implementation.^154 , 155^

In prenatal care, the Family Health Teams should stratify maternal and fetal risks, considering reproductive history, individual characteristics, and unfavorable sociodemographic conditions. The recruitment of women of reproductive age (10-49 years) is essential to stratify pregnancies at risk for complications, such as: extremes of age (under 15 years and over 35 years), low body weight and overweight or obesity, unsafe family situation, non-acceptance of pregnancy (mainly in adolescents), low schooling, women with heart disease, chronic hypertension and/or history of gestational hypertensive disorder, and gestational diabetes mellitus. In puerperium, special attention should be given to women’s psychic and social conditions, as well as protection and support to breastfeeding, which should be exclusive in the first six months of life.^154 , 155^

Menopausal transition is associated with an increased risk for CVD, mainly attributed to atherogenic dyslipidemia, central obesity, and insulin resistance, and with an increased risk for arterial hypertension.^156^The MHT should be individualized, considering personal and family risks for gynecological neoplasia, in addition to cardiovascular risk stratification. The MHT should be systematically accompanied in PHC. Estrogen replacement can reduce the serum levels of total cholesterol and LDL-c because of the increased synthesis of LDL-c receptors. Benefits from MHT were observed when introduced before the age of 60 years and in women whose menstrual cycles ceased less than 10 years before (preferably in the first 5 years); however, it can be harmful if outside the window of opportunity, increasing cardiovascular risk.^49^

In 2019, some of the indicators of woman’s health agreed upon in the Brazilian prevention program, *Programa Previne Brasil,* were as follows: pregnant women registered in the PHC, mean number of prenatal visits, prenatal follow-up initiated in the first trimester, pregnant women with up-to-date vaccines, home visits for pregnant women, adolescent pregnancy, proportion of normal deliveries, maternal deaths, cytopathology of the uterine cervix, screening with mammography in women from 50 to 69 years. It is worth noting the lack of any cardiovascular health indicator agreed upon in that prevention program.^157^In addition, the OECD has recently released a report on the PHC in Brazil, in which women’s CVDs have not been mentioned. Regarding women, that report only made suggestions for the management of breast and ovary neoplasms; however, in percentage terms, CVDs cause twice more deaths than all other neoplasms in Brazil.^158^

A paradigm change in the basic assumptions of the public policies on women’s total health is necessary, especially considering that CVDs are the major cause of death of women in most of their life cycle ( Figure 7.1 ).

**Figure 7.1:** Primary Health Care strategies for women’s cardiovascular health. CVRF: cardiovascular risk factor

Nonpharmacological therapies incorporating lifestyle changes (exercises, body weight loss, smoking cessation, and healthy diet) should be recommended as first-line strategy in PHC. The efficacy of such measures is directly associated with better understanding, sensitization, and motivation built during the Family Health Team follow-up. The management of healthy dietary habits, regular physical activity practice, and mental health should be individualized.^26^Examples of validated tools for patients’ assessment, in addition to clinical history and physical exam, are the food guide for the Brazilian population ( *Guia Alimentar para a População Brasileira* ), the World Health Organization guidelines on physical activity and sedentary behavior, the stress and depression scales, and the questionnaires of spirituality.^26^

Of all possible interventions in PHC, the most cost-effective is smoking cessation. The harmful effects of regular and electronic cigarette smoking are greater in women, specifically because of the loss of estrogen protection on the vascular endothelium.^159^

In Brazil and around the world, violence against women is a serious public health problem, because it is one of the major causes of female morbidity and mortality. In PHC, actions that can ensure sexual rights in the perspective of women’s autonomy regarding their bodies should be developed.^160^

Considering the challenges to control CVD in Brazil and recognizing PHC as an important strategy for health promotion, risk factor prevention, early diagnosis, and care to individuals with CVD, a critical review to improve health actions for women in the Family Health Teams and PHC is suggested ( Figure 7.1 ).^160^

## 8. Women’s Burnout, Quality of Life and Spirituality

### 8.1. Burnout

Burnout is a psychological syndrome resulting from chronic stress at work, characterized by emotional exhaustion, cynicism/disengagement, and reduced professional efficacy.^161^The dimensions of burnout were significantly associated with an increased risk for diseases, regardless of sociodemographic factors and depressive symptoms. In a study with 5671 participants [predominantly physicians, mean age of 44.1 years (range, 18 to 70 years), 62.4% women], a digital health app was used for online research about professional burnout measured with the Maslach Burnout Inventory General Survey. By use of network analysis and logistic regression, the study has shown the association of high emotional exhaustion with arterial hypertension and other chronic diseases after adjusting for age, sex, educational level, and depressive symptoms.^162^

Work conditions have a well-known impact on the health of workers, and women, because they make up a great proportion of the global workforce and are overloaded with double journey, show high rates of burnout. A study conducted with Brazilian female physicians during the COVID-19 pandemic has reported that 61.6% of the participants had signs of burnout, such as emotional exhaustion, frequent negative feelings, and dissatisfaction with their capability for work ( Figure 8.1 ).^162^

**Figure 8.1:** Study performed with Brazilian female physicians during the COVID-19 pandemic. The findings emphasized the higher susceptibility of women to psychosocial factors, with a significant impact on quality of life, which can cause burnout, minimized by spirituality, especially in the COVID-19 pandemic. 162

It is worth noting that women are more exposed to stress and psychosocial adversities than men are, in addition to being more vulnerable to the effects of such exposures. Depression, unfavorable socioeconomic factors, and post-traumatic stress disorder are more prevalent in women than in men and tend to show more robust associations with cardiometabolic risk in women.^67^

### 8.2. Quality of Life

According to the World Health Organization, QoL is “an individual’s perception of their position in life in the context of the culture and value systems in which they live and in regard to their goals, expectations, standards and concerns”. The QoL is directly related to the higher incidence of chronic diseases, mainly CVD. The standard indicators of QoL include job, wealth, environment, physical and mental health, education, recreation and leisure, socialization, religious beliefs, safety, protection, and liberty.^163^

In a study assessing the QoL of Brazilian female physicians, most participants reported having good QoL (71.7%) and being satisfied with their health (55%), but 64.8% reported not really enjoying their lives. In addition, they considered the following aspects of their lives satisfactory: sleep, 62.9%; ability to perform daily chores, 54.7%; capability for work, 64.4%; personal relationships, 57.7%; support from friends, 61%; conditions of the household, 84%; and access to healthcare, 81.4%. Only 36.6% considered their sexual life satisfactory, and around 94% had, at least occasionally, negative feelings ( Figure 8.1 ).^162^

A study conducted with 1387 women in the city of Uberaba, Minas Gerais state, has assessed the QoL by using the WHOQOL-Brief and according to four domains. The authors reported that women with CVD were older than 50 years and had low educational level and statistically significant lower values for all four domains as compared to women without disease or those with chronic respiratory disease. The domain with the lowest score was environment, which relates to socioeconomic condition because it contains questions related to financial resources, leisure opportunities, safety, etc. These findings emphasize the higher susceptibility of women with CVD to psychosocial factors, with significant impact on QoL.^164^

### 8.3. Spirituality

“Spirituality is a set of moral, mental, and emotional values that guide thoughts, behaviors, and attitudes in the circumstances of intra- and interpersonal relationships, motivated by the will, and capable of being observed and measured”. Spirituality must be differentiated from religiousness. “Religion is an organized system of beliefs, practices, and symbols destined to facilitate the proximity with the Divine and promote the understanding of one’s relationships and responsibilities with the others in the community”.^26^

In addition to the behavioral aspects, the beneficial relationship of spirituality, religiousness, and the physiological and pathophysiological variables of several clinical entities, including women’s CVDs, has been shown.^165^Spiritual anamnesis has been more and more introduced in medical offices and hospitals, being part of the clinical history, especially in severe, chronic, progressive, debilitating, and terminal diseases. There are validated tools for assessment, based on health scales (FICA, FAITH, SPIRIT e HOPE).^26 , 163 - 173^

A study with Brazilian female physicians, using a survey with questions based on the World Health Organization Quality of Life Spirituality, Religiousness and Personal Beliefs field-test instrument (WHOQOL-SRPB), has reported that the respondents believed that spirituality comforted and reassured them (73.2%) and that they found spiritual force in hard times (70.6%), with good connection of body, mind, and spirit (67.8%), even though only 53.4% reported inner peace and 50.7% reported being optimistic. In addition, 72.7% of the respondents reported finding strength in faith, and 44.3% found support in religious or spiritual communities ( Figure 8.1 ).^162^

In conclusion, health needs to be understood in the physical, social, psychoemotional, and spiritual context, respecting the individuality and singularity of the female sex.^44^

Chart 8.1 shows the recommendations for the management of women’s spirituality and health.

**Chart 8.1:** Recommendations for the management of women’s spirituality and health.

Recommendations for the management of women’s spirituality and health			

Recommendation	CR	LE	References
Brief screening on spirituality and religiousness.		**B**	^26,163-173^
Spiritual anamnesis of patients with chronic diseases or of poor prognosis.		**B**	^26,163-173^
Respect and support patient’s religion, beliefs, and personal rituals that do not jeopardize treatment.		**C**	^26,163-173^
Offer professional support to patients in pain or with spiritual demands.		**C**	^26,163-173^
Organizational religiousness associates with mortality reduction.		**B**	^26,163-173^
Spiritual anamnesis of stable or ambulatory patients.	Ia	**B**	^26,163-173^
Questionnaires (DUREL, FICA, HOPE, or FAITH) to assess spirituality.	a	**B**	^26,163-173^
Meditation, techniques for relaxation and fighting stress.	a	**B**	^26,163-173^
Techniques for spiritual strengthening, such as forgiveness, gratitude, and resilience.	b	**C**	^26,163-173^

CR: class of recommendation; LE: level of evidence.

## 9. Cardiovascular Implications of COVID-19 in Pregnancy

The major complications reported in patients with COVID-19 are: myocardial injury, heart failure, arterial and venous thromboembolism, acute coronary syndrome, myocarditis, Takotsubo syndrome, and cardiac arrhythmias.^191^Those complications are more frequent in older women, with risk factors for cardiovascular diseases and with comorbidities.^191^There is great concern about women during pregnancy and puerperium, who are more susceptible to severe forms of COVID-19, which can result in premature or emergency cesarean delivery, elevating the risk of neonatal and maternal death ( Figure 9.1 ).^192^

**Figure 9.1:** Preventive measures for pregnant women with COVID-19. NT-proBNP: N-terminal fragment of pro-B-type natriuretic peptide.

During pregnancy, the predominant immune response is via T-helper 2 (Th2) cells, which protects the fetus, but increases mother’s vulnerability to viral infections, which are more effectively fought by Th1 cells.^193^The vertical transmission of SARS-CoV-2 can occur through the transplacental route, during delivery and while breastfeeding. The SARS-CoV-2 transmission via blood is still uncertain.^162^The physiological changes of pregnancy comprise the increase in plasma and systolic volume, and in cardiac output in the first half of pregnancy, with a gradual increase in heart rate, in addition to a decrease in systemic and pulmonary vascular resistance. Furthermore, pregnancy is a hypercoagulable state associated with increased risk of venous and pulmonary thromboembolism.^194^

The COVID-19 clinical findings have some particularities in pregnancy, such as longer persistence of symptoms and lower frequency of fever and myalgia, as compared to non-pregnancy. The major risk factors for severe infection are older maternal age, high body mass index, and preexisting comorbidities, such as hypertension, preeclampsia, and diabetes.^194^Pregnancy was associated with severe infection in 10%, admission to the intensive care unit in 4%, mechanical ventilation in 3%, and use of extracorporeal membrane oxygenation in 0.2%.^194 , 195^

In addition, perinatal complications were also more prevalent. When compared to pregnant women without infection, those with COVID-19 had a higher risk of premature delivery and stillbirth. Overall, 33% of the newborns from women with COVID-19 were admitted to the neonatal intensive care unit.^192 , 194^Even after adjusting to race, comorbidities, and age, pregnant women had a high likelihood to die as compared to their non-pregnant counterparts.^196^

The severe cardiovascular complications in COVID-19 are acute myocardial injury, myocarditis, arrhythmia, fulminant heart failure, cardiogenic shock, and spontaneous dissections of the coronary and vertebral arteries, which have higher mortality.^192^

The potential contributors to acute cardiac injury in COVID-19 are:^191^acute changes in myocardial supply and demand due to tachycardia, hypotension, and hypoxemia, resulting in type 2 myocardial infarction;^192^acute coronary syndrome due to acute atherothrombosis in a virus-induced thrombotic and inflammatory condition;^193^microvascular dysfunction due to diffuse microthrombi or vascular lesion;^194^stress-related cardiomyopathy (Takotsubo syndrome);^195^non-ischemic myocardial injury due to hyperinflammatory cytokine storm;^196^or direct viral toxicity to cardiomyocytes and myocarditis. The medicamentous treatment of heart failure in pregnancy has particularities that should be considered to avoid teratogenicity.^193^

In cases of ST-elevation acute coronary syndrome, coronary angiography should be preferentially performed and followed by percutaneous treatment. In 15 pregnant patients with COVID-19 and myocardial injury, 13.3% had atrial fibrillation, 2 had supraventricular tachycardia, and 2 patients developed *torsades de pointes.* ^197^The use of medications that can prolong the QT interval should be carefully considered.

COVID-19 can both predispose to preeclampsia and worsen its course. The virus generates a pathophysiological state similar to that of preeclampsia, characterized by systemic hyperinflammation, direct endothelial injury, thrombogenesis, and immune dysregulation; in addition, the virus affects the renin-angiotensin-aldosterone system, increasing the incidence of preeclampsia in infected patients.^198^

The decision about interrupting pregnancy should be based on the usual guidelines, which consider gestational age, hemodynamic conditions, fetal suffering, and maternal risk. Non-complicated COVID-19 should not be an indication for pregnancy interruption. The type of delivery should follow the obstetrical recommendations.^199^

## 10. Future Perspectives on Women’s Cardiovascular Healthcare

A multidisciplinary team is always recommended for preventive care and treatment in the follow-up of women with CVD. To raise awareness includes the creation of integrative health programs, involving communitarian leaders and agents, with new centers specializing in women’s cardiovascular health. It is important to specifically educate on self-care to prevent CVD, proper use of oral contraception, prenatal follow-up, long-term follow-up of CVRFs, as well as the psychosocial and socioeconomic aspects involving CVDs in the female sex.^200^

Primary prevention in women should focus on the effective treatment of traditional risk factors, such as SAH, dietary risks, dyslipidemia, diabetes, obesity, and physical inactivity. The lack of specific risk scores for women indicates long-term risk stratification, considering life course and the female-sex-specific factors, such as the presence of depression, as well as the psychosocial factors.^200^It is worth emphasizing that the frequency of HFpEF in women is higher than in men; in addition, women more often have HFpEF than HFrEF, and treatment should

be initiated with the clinical diagnosis of HF, regardless of the ejection fraction, because of the greater relevance of the cognitive deficit associated with HF in the female sex.^201^

The major attributable risk factor for the development of cardiovascular outcomes in both sexes is SAH. The BP control is essential to reduce the incidence of HFpEF and HFrEF.^202 , 203^However, women have lower rates of BP control as compared to men, mainly at more advanced age groups.^204^Strategies that include BP measurement outside the medical office, such as BP self-measurement, home and ambulatory BP monitoring, using semi-automated equipment are desirable. In addition, remote monitoring using digital platforms and apps, with continuous BP monitoring without cuffs and synchronized with smartphones, represents the use of technology and wearables of easy access, favoring the clinical results, and might help better BP control.^205 , 206^

Because of the medical, surgical, and technological evolution in recent decades, more than 90% of individuals with CC born nowadays survive until adulthood. The especial attention to women with CC requires planning strategies to address their needs.^11^Thus, centers specialized in CC should be created with multidisciplinary teams that include physicians, nurses, psychologists, and social workers for contraception counselling or pregnancy planning. The risks of an adult patient with CC, treated or not during childhood, especially regarding contraception and pregnancy, should always be considered, because 3% to 10% of the babies from those mothers can have congenital cardiac lesions.^207^Women of reproductive age with cardiopathy should be approached by a multidisciplinary team, a “cardio-obstetrical team”, for pre-conceptional counselling to define pregnancy, delivery and puerperium planning, including specialized cardiological care and family planning after delivery.^208^

The prevention and treatment of CVDs in women require solid health systems supported by professionals knowing the specificities of CVDs in women, in addition to coordinated efforts with productive partnerships between society, physicians, researchers, and the community.^70^

The “Women’s Letter”, published in 2019, suggests the creation of a permanent group to promote and implement policies directed to women’s cardiovascular health. That group should play a leadership role in Brazilian health policies, providing managers with an overview of the relevance of CVD in the female sex, so that strategies can be developed to reduce the prevalence of risk factors and improve diagnosis and treatment, therefore reducing CVD mortality and morbidity ( Figure 10.1 ).^4^

**Figure 10.1:** Perspectives for management of cardiovascular diseases in women according to the ‘Women’s Letter’. 4 CV: cardiovascular; NCD: noncommunicable disease; CVD: cardiovascular disease.
